# Alkylative kinetic resolution of vicinal diols under phase-transfer conditions: a chiral ammonium borinate catalysis†
†Electronic supplementary information (ESI) available. See DOI: 10.1039/c7sc04854h


**DOI:** 10.1039/c7sc04854h

**Published:** 2017-12-12

**Authors:** Martin Pawliczek, Takuya Hashimoto, Keiji Maruoka

**Affiliations:** a Department of Chemistry , Graduate School of Science , Kyoto University , Sakyo , Kyoto , 606-8502 , Japan . Email: takuya@kuchem.kyoto-u.ac.jp ; Email: maruoka@kuchem.kyoto-u.ac.jp

## Abstract

Herein, we report the first alkylative kinetic resolution of vicinal alcohols realized by cooperative use of a chiral quaternary ammonium salt and an achiral borinic acid. In addition, a catalytic regioselective alkylation of a secondary alcohol in the presence of an unprotected primary one is presented, emphasizing the unique selectivity and potential of this ammonium borinate catalysis.

## Abstract 2

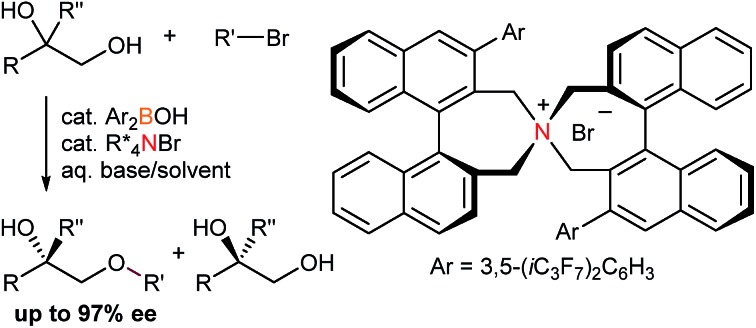
Alkylative kinetic resolution of vicinal alcohols is realized by a cooperative chiral ammonium borinate catalysis.

## Introduction

Chiral phase-transfer catalysis has been extensively studied in the past three decades due to its operational simplicity and benign reaction conditions. A large body of work focusing on the development of new chiral catalysts and reactions was reported during this period.^1^ Despite these continuing progresses, one simple reaction, namely the asymmetric functionalization of unprotected alcohols, has remained intractable in chiral phase-transfer catalysis as a way to provide enantioenriched molecules. It is in contrast with the fact that the functionalization of alcohols has been known to be accelerated in the presence of quaternary ammonium salts long before the emergence of chiral phase-transfer catalysis.^2^ From our experience in this field,1a,b we surmised that an obstacle to achieve this goal is the formation of a localized, hard alkoxide anion. Compared to a delocalized and flat anion like an enolate,^3^ an alkoxide is structurally more flexible, rendering the recognition by a chiral cation difficult. The strong basicity of an alkoxide is also a problem since such a strong base is known to degrade the phase-transfer catalyst and is incompatible with base-sensitive substrates.

**Fig. 1:**
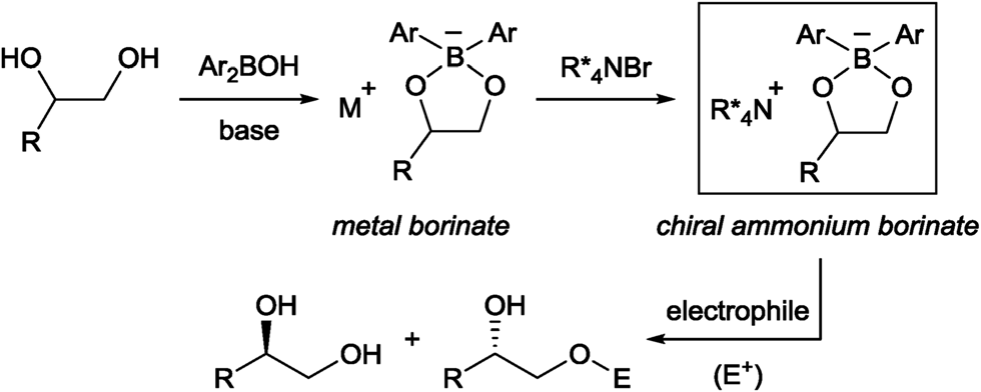
Cooperative catalysis for the asymmetric functionalization of vicinal diols.

Taking these facts into consideration, we planned to realize an enantioselective functionalization of alcohols by merging the chiral ammonium salt with an additional catalyst which is able to interact directly with an alcohol and modify its properties. In this regard, Taylor *et al.* recently reported the use of achiral borinic acids for the mono-functionalization of vicinal diols *via* an intermediary formed borinate (Fig. 1).^4^,^5^ In such an anionic species, the alkoxide is structurally fixed and its basicity is attenuated. Deploying a chiral quaternary ammonium salt to pair up with this anionic species is expected to create a chiral environment around the diol, facilitating asymmetric functionalization of the diol (Fig. 1).^6^

We report herein our primary investigations on this subject using an alkylative kinetic resolution of vicinal diols. Despite the fact that alkylation of alcohols directly forges ubiquitous ether moieties,^7^ such transformation has remained unexplored in asymmetric catalysis.^8^,^9^ High enantioselectivities were achieved in the kinetic resolution of secondary as well as tertiary diols by exploiting this chiral ammonium borinate catalysis. The availability of the optimal chiral quaternary ammonium salt, a persistent bottleneck in the binaphthyl-based chiral phase-transfer catalysts,1a,b was enhanced by applying a metal-catalyzed C–H arylation. To further highlight the unique reactivity and selectivity of this catalysis, we also demonstrate a catalyst-controlled, regioselective alkylation of an innately less reactive secondary alcohol of a terminal vicinal diol.

## Results and discussion

The first challenge to achieve our goal was the confirmation of reaction conditions to facilitate the alkylation only when both the chiral quaternary ammonium salt and the borinic acid are present. The optimization was implemented by use of (*rac*)-1-phenyl-1,2-ethanediol **1a** and benzyl bromide as reactant in combination with 2 mol% of a simple chiral ammonium salt **4** and 10 mol% of a nitrogen-tethered borinic acid **8** as catalyst pair (Table 1).^10^ Initially we found out that a biphasic system consisting of CH_2_Cl_2_ and aqueous base suppresses independent catalyst activities and ensures enantioselective product formation through a cooperative catalytic pathway (entries 1–3). Screening of a collection of chiral ammonium salts (see ESI†) revealed the effectiveness of catalyst **6** (entries 4 and 5),^11^ which was further optimized by attaching 3,5-bis(heptafluoroisopropyl)-phenyl substituents (**7**) (entries 6 and 7).^12^ A final optimization resulted in the use of 4 mol% of **7**, 5 mol% of **8**, a CH_2_Cl_2_–toluene co-organic solvent system, an excess amount of benzyl bromide, 2 equivalent of Cs_2_CO_3_ and NaI as additive. Under these conditions, product **2aa** was obtained in 44% yield with 91% ee (entry 8). Interestingly, a considerable amount of side-product **3aa**, derived from alkylation of the secondary alcohol, was also observed with the preferential uptake of (*R*)-**1a**, indicating the divergent nature of this catalysis.

**Table 1:** Optimization of the reaction conditions^*a*^ ^–^^*c*^

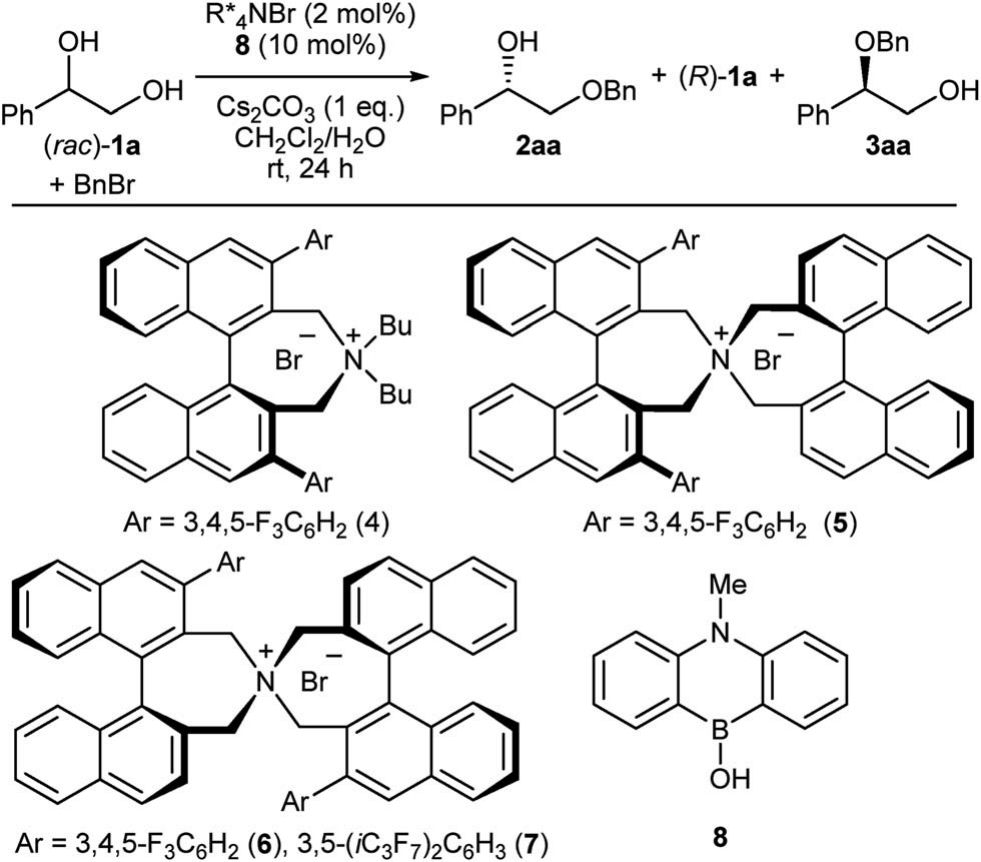							
Entry	PTC	**2aa** (% yield, % ee)		**1a** (% yield, % ee)		**3aa** (% yield, % ee)	
1	**4**	17	21	72	4	—	—
2^*d*^	**4**	—	—	97	0	—	—
3	—	—	—	97	0	—	—
4	**5**	14	3	78	1	—	—
5	**6**	15	45	75	8	—	—
6^*e*^	**6**	4	54	90	2	—	—
7^*e*^	**7**	7	87	86	4	—	—
8^*f*^ ^,^^*g*^	**7**	**44**	**91**	49	74	6	22

^*a*^Performed with (*rac*)-**1a** (0.10 mmol), benzyl bromide (0.06 mmol), phase-transfer catalyst (2 mol%) and **8** (10 mol%) in CH_2_Cl_2_/H_2_O (0.35 mL/0.65 mL) at rt.

^*b*^NMR yield.

^*c*^ee determined by chiral HPLC.

^*d*^Performed without **8**.

^*e*^Performed at 0 °C for 48 h.

^*f*^ **7** (4 mol%), **8** (5 mol%), Cs_2_CO_3_ (2 eq.) and NaI (5 eq.) in CH_2_Cl_2_/toluene/H_2_O (0.15 mL/0.20 mL/0.65 mL) at 0 °C for 96 h.

^*g*^Isolated yield.

**Scheme 1:**
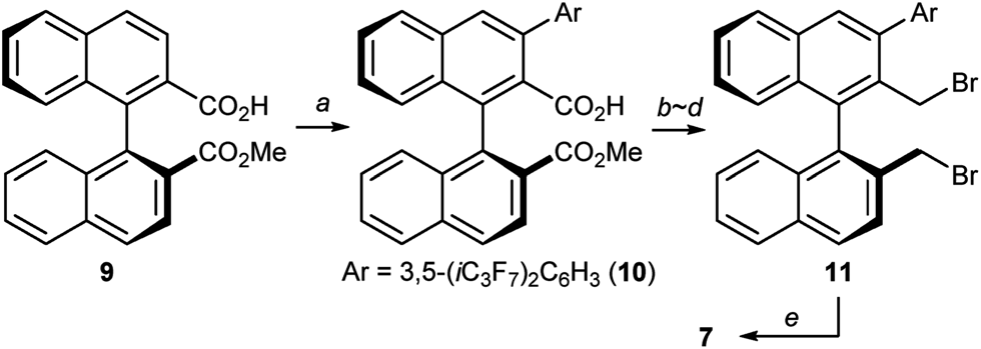
Preparation of catalyst **7**. Conditions: (a) [(*p*-cym)RuCl_2_]_2_, Bu_3_P, 3,5-(*i*C_3_F_7_)_2_C_6_H_3_I, K_2_CO_3_, NMP, 110 °C, 78% yield; (b) LiOH aq., THF, reflux; (c) BH_3_·Me_2_S, THF, reflux, 86% yield (2 steps); (d) PBr_3_, THF, rt, 88% yield; (e) NH_3_ aq., CH_3_CN, 80 °C, 69% yield.

At this stage, we decided to streamline the synthesis of the structurally complex phase-transfer catalyst **7** with the help of the state-of-the-art C–H arylation.^13^ Starting from readily available 1,1′-binaphthyl-2,2′-dicarboxylic acid,^14^ direct mono C–H arylation of the 3-position was investigated using several methods. It was revealed that the ruthenium-catalyzed arylation of mono ester **9** with 3,5-(*i*C_3_F_7_)_2_C_6_H_3_I is a robust and scalable procedure (Scheme 1).^15^,^16^ Arylated compound **10** was further transformed into dibromide **11** by conventional synthetic methods, which upon treatment with aqueous ammonia yielded the desired catalyst **7**.

With the optimized reaction conditions and the secure supply of the catalyst in hand, we investigated the substrate scope with various alkyl halides (Table 2, entries 1–13). As for benzylic bromides, regardless of the substituent pattern on the aromatic ring, the alkylation proceeded with good enantioselectivities at nearly half conversion of the diol (**2ab–2aj**). The reaction could be scaled up to 1.0 mmol without difficulty as shown in the preparation of **2ad** (entry 3). Efficient kinetic resolution took place with prenyl and cinnamyl bromides to afford products **2ak** and **2al** with high enantioselectivities (entries 10 and 11). The mild reaction conditions allowed the use of base-sensitive methyl 4-bromocrotonate, to give **2am** bearing an unsaturated ester (entry 12). The reaction using phenylpropargyl bromide was found to be slow, giving **2an** in 36% yield with 90% ee after 6 d (entry 13). In these experiments, we isolated some amount of side-products derived from the alkylation of the secondary alcohol (*e.g.***3aa** in Table 1) in a range from 10% to 30% yield in addition to the recovered (*R*)-diols **1** (see ESI† for detail).

**Table 2:** Asymmetric alkylation of terminal vicinal diols^*a*^ ^–^^*c*^

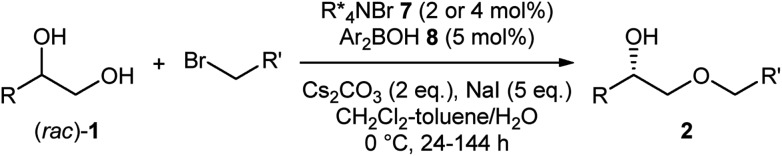					
Entry	R	R′	% Yield	% ee	
1^*d*^	Ph	2-MeC_6_H_4_	51	87	**2ab**
2	Ph	3-MeC_6_H_4_	46	82	**2ac**
3^*d*^ ^,^^*e*^	Ph	4-MeC_6_H_4_	48 (48)	87 (86)	**2ad**
4	Ph	2-FCC_6_H_4_	47	90	**2ae**
5	Ph	2-BrC_6_H_4_	45	85	**2af**
6	Ph	2-CNC_6_H_4_	49	87	**2ag**
7	Ph	4-PhC_6_H_4_	46	91	**2ah**
8	Ph	3,5-(CF_3_)_2_C_6_H_4_	36	89	**2ai**
9	Ph	2-Naphthyl	36	91	**2aj**
10	Ph	(Me)_2_C=CH	40	89	**2ak**
11	Ph	(*E*)-PhCH=CH	39	84	**2al**
12	Ph	(*E*)-MeO_2_CH=CH	39	80	**2am**
13	Ph	PhC≡C	36	90	**2an**
14	2-MeC_6_H_4_	Ph	45	83	**2ba**
15	3-MeC_6_H_4_	Ph	40	87	**2ca**
16	4-MeC_6_H_4_	Ph	34	97	**2da**
17	2-FC_6_H_4_	Ph	42	87	**2ea**
18	3-FC_6_H_4_	Ph	40	87	**2fa**
19	4-FC_6_H_4_	Ph	44	91	**2ga**
20	4-BrC_6_H_4_	Ph	33	82	**2ha**
21	4-MeOC_6_H_4_	Ph	46	88	**2ia**
22	Cy	Ph	38	48	**2ja**

^*a*^Performed with **7** (4 mol%), **8** (5 mol%), Cs_2_CO_3_ (2 eq.) and NaI (5 eq.).

^*b*^Isolated yield.

^*c*^ee determined by chiral HPLC.

^*d*^Performed with 2 mol% of **7**.

^*e*^Yield and ee in parentheses are the results of 1 mmol scale experiment.

We then turned our attention to the use of various terminal vicinal diols as nucleophile (Table 2, entries 14–22). A variety of aromatic diols were found to be applicable irrespective of the substituent pattern and the electronic property (**2ba–2ia**). The use of an aliphatic diol gave the benzylated product **2ja** with lower enantioselectivity, implying the necessity of catalyst re-optimizations for this class of substrates.

As more challenging substrates, terminal vicinal diols (*rac*)-**12** with a tertiary alcohol moiety were examined under our reaction conditions (Table 3).9*g* Gratifyingly, exactly the same co-catalytic system worked for substrates having an additional methyl group (entries 1–8). At nearly half conversion, benzylated products **13a–13h** were obtained with enantioselectivities ranging around 80% to 90%. Attachment of other alkyl substituents instead of the methyl group led to lower enantioselectivity as exemplified by **13i** (entry 9). A general requirement for tertiary alcohols is a higher borinic acid loading and longer reaction time due to the slow turnover of the catalysis.

**Table 3:** Alkylation of tertiary vicinal diols^*a*^ ^–^^*c*^

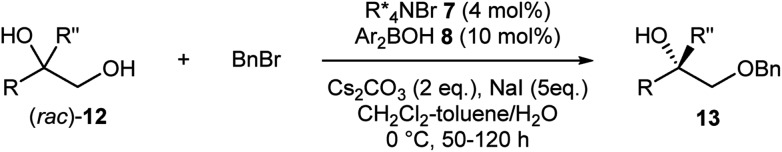					
Entry	R	R′′	% Yield	% ee	
1	Ph	Me	47	84	**13a**
2	2-MeC_6_H_4_	Me	42	82	**13b**
3	3-MeC_6_H_4_	Me	38	85	**13c**
4^*d*^	4-MeC_6_H_4_	Me	41	83	**13d**
5	4-FC_6_H_4_	Me	30	91	**13e**
6	4-MeOC_6_H_4_	Me	46	87	**13f**
7	2-Naphthyl	Me	33	78	**13g**
8	3-Thienyl	Me	44	72	**13h**
9	Ph	CH_2_CH=CH_2_	25	69	**13i**

^*a*^Performed with **7** (4 mol%), **8** (10 mol%), Cs_2_CO_3_ (2 eq.) and NaI (5 eq.).

^*b*^Isolated yield.

^*c*^ee determined by chiral HPLC.

^*d*^Performed with 15 mol% of **8**.

**Fig. 2:**
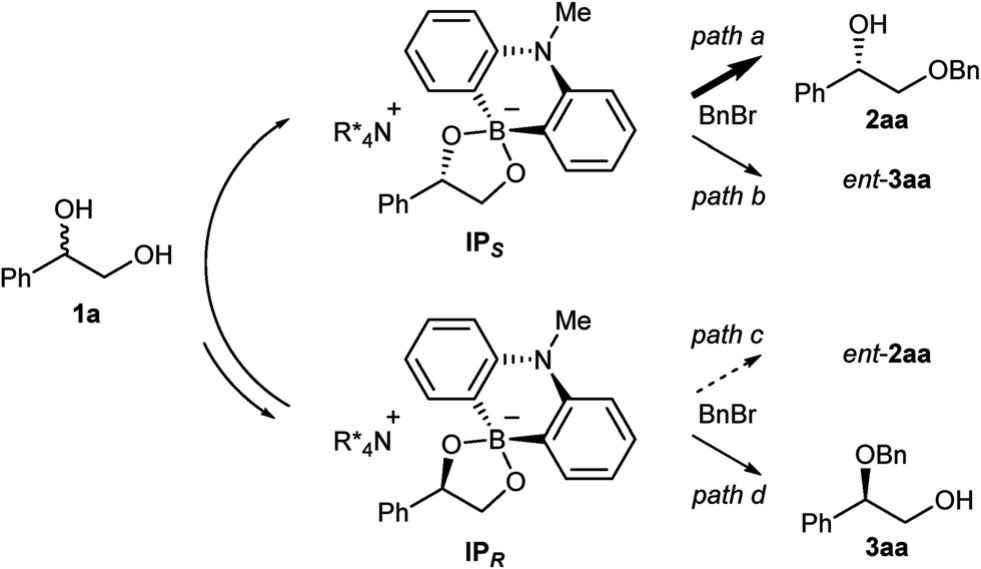
The reaction pathways of the ammonium borinate catalyzed alkylation.

We then carried out NMR experiments of the ammonium borinate prepared from 1 : 1 : 2 ratio of **7**, **8** and (*rac*)-**1a**, and Cs_2_CO_3_ in CDCl_3_ (see ESI† for detail). The analysis of the ^1^H and ^11^B NMRs confirmed the formation of two diastereomeric ammonium borinates favoring the ion pair **IP*_S_*** complexed with (*S*)-**1a** over **IP*_R_*** (Fig. 2). Addition of more of (*rac*)-**1a** to this solution shifted the ratio of ion pairs within minutes, indicating that these two diastereomeric pairs are in a rapid equilibrium relative to the reaction time frame. This observation suggested that the alkylation, not the ion pairing, is the enantio-determining step. Therefore, the enantioselectivity arguably stems from the reactivity difference of **IP*_S_*** and **IP*_R_*** for alkylation (path *a* > path *c*). As a result of deceleration of the reaction *via* path *c*, **IP*_R_*** is considered to react eventually at the innately less reactive secondary alcohol to give **3aa**.

**Scheme 2:**
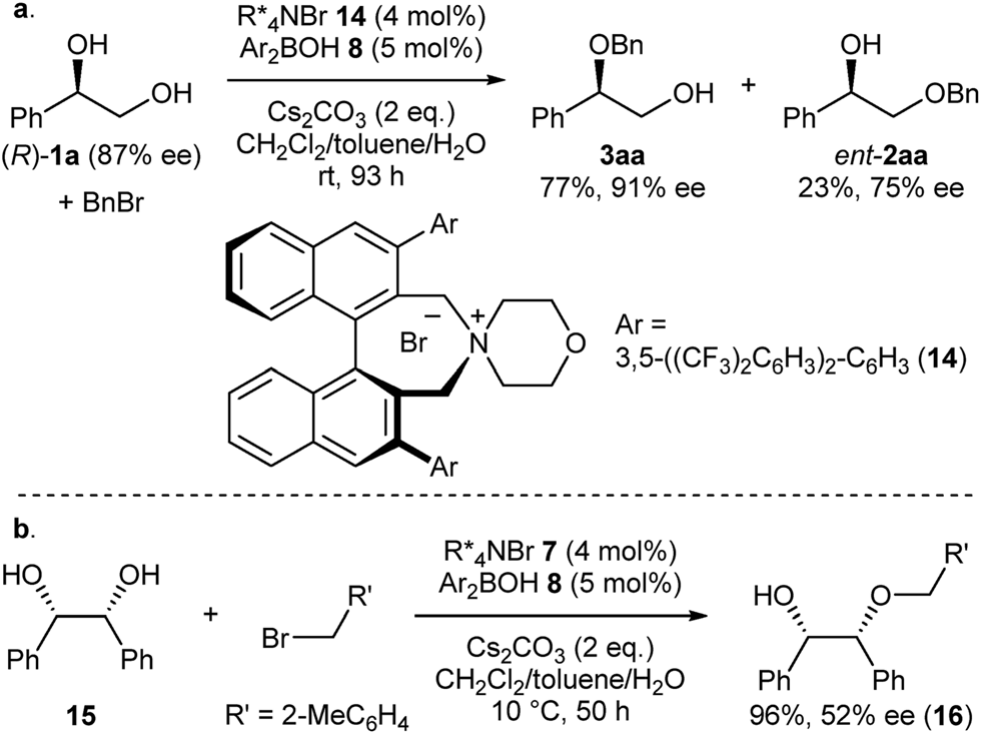
(a) Catalyst-controlled, regioselective alkylation of the secondary alcohol of (*R*)-**1a**. (b) Desymmetrization of *meso*-diol **12**.

Intrigued by the unique regioselectivity toward secondary alcohol observed with **IP*_R_***, we decided to take a closer look on this phenomenon (Scheme 2a).6b,^17^,^18^ Such a transformation is in contrast to the studies conducted by Taylor *et al.*, in which the borinic acid exclusively functionalized the primary alcohol of a terminal diol.4*b* It was thus surmised that the judicious choice of an ammonium salt would reveal a novel, catalytic regioselective alkylation of the secondary alcohol of terminal vicinal diols. By analyzing the results of the previous catalyst screening (Table 1 and ESI†), we could quickly identify the optimal catalyst **14** for this purpose. Starting from commercially available (*R*)-**1a** with 87% ee, the catalyst controlled, regioselective alkylation proceeded with a ratio of 3.4 : 1 in favor of the secondary alcohol. A slight increase in enantiomeric excess for the desired product **3aa** was observed as well.

Finally, we applied our catalyst system to the desymmetrization of a *meso*-diol (Scheme 2b). Treatment of *meso*-diol **15** with 2-methylbenzyl bromide gave the desired compound **16** in 96% yield with 52% ee paving the way for further optimizations in the future.

## Conclusions

In conclusion, we developed herein the cooperative catalytic system capable of performing asymmetric functionalization of alcohols under phase-transfer conditions as demonstrated by the first alkylative kinetic resolution of secondary and tertiary diols. In addition, this method allowed a catalyst-controlled, regioselective alkylation of the less accessible secondary alcohol in the presence of an unprotected primary one. Our ongoing research is focused on the application of this chiral ammonium borinate catalysis to other electrophiles and more complex diols like saccharides.

## Conflicts of interest

There are no conflicts to declare.

## Supplementary Material

Supplementary information
