# Supplementary material for: Alkylative kinetic resolution of vicinal diols under phase-transfer conditions: a chiral ammonium borinate catalysis
Source: Chem Sci. 2017 Dec 12;9(5):1231–5. doi: 10.1039/c7sc04854h (PMC5885781; doi:10.1039/c7sc04854h)
Supplement: Supplementary file 1 [file SC-009-C7SC04854H-s001.pdf]

Electronic Supplementary Material (ESI) for Chemical Science.
This journal is © The Royal Society of Chemistry 2017

Electronic Supplementary Information

Alkylative Kinetic Resolution of Vicinal Diols under Phase-Transfer Conditions: A Chiral Ammonium Borinate Catalysis

Martin Pawliczek, Takuya Hashimoto* and Keiji Maruoka*

Department of Chemistry, Graduate School of Science, Kyoto University, Sakyo, Kyoto, 606-8502, (Japan).

Table of Contents

General Information 2
Representative Examples from the PTC Screening 3
Catalyst Synthesis (Scheme 1) 4
General Procedure (Tables 2 and 3) 8
Product Scope (Tables 2 and 3) 9
Alkylation of Secondary Alcohol and Desymmetrization (Scheme 2) 29
NMR Experiment (Figure 2) 31
References 33
NMR Spectra 34
HPLC 83

General Information

Infrared (IR) spectra were recorded on a ThermoFischer Scientific NICOLET iS5 spectrometer. ^{1}H NMR spectra were measured on JEOL JNM-FX400 (400 MHz) and JNM-ECA500 (500 MHz) spectrometers. Data were reported as follows: chemical shifts in ppm from tetramethylsilane as an internal standard or from the residual solvent in in $CDCl_3$ or acetone-d_6, integration, multiplicity (s = singlet, d = doublet, t = triplet, q = quartet, quin = quintet, m = multiplet, br = broad, app = apparent), coupling constants (Hz), and assignment. ^{13}C NMR spectra were measured on JEOL JNM-FX400 (100 MHz) and JNM-ECA500 (125 MHz) spectrometers with complete proton decoupling. Chemical shifts were reported in ppm from the residual solvent as an internal standard. High performance liquid chromatography (HPLC) was performed on Shimadzu 10A instruments at 210 nm using 4.6 mm x 25 cm Daicel chiral columns. High-resolution mass spectra (HRMS) were performed on Thermo Scientific Exactive Plus Orbitrap LC-MS. Optical rotations were measured on a JASCO DIP-1000 digital polarimeter. For thin layer chromatography (TLC) analysis throughout this work, Merck precoated TLC plates (silica gel 60 GF_{254}, 0.25 mm) were used. The products were purified by flash column chromatography silica gel 60 (Merck, 230-400 mesh) or preparative thin layer chromatography silica gel (PLC 60 F_{254}. 0.5 mm).

In experiments requiring dry solvent, CH_2Cl_2, toluene and THF were purchased from Kanto Chemical Co. Inc. as "Dehydrated" and further purified by passing through neutral alumina under nitrogen atmosphere. Commercially obtained reagents were used as received.

Representative Examples from the PTC Screening

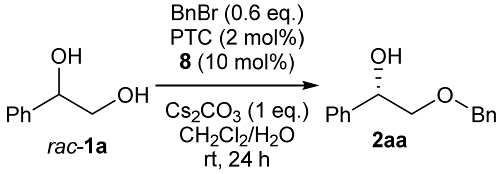

PTC	Yield[a] [%] (Product)	e.e.[b] [%]
1	8	-5
2	15	0
3	6	16
4	6	16
5	6	11
6	12	0
7	11	0
8	5 (12)[c]	24 (28)[c]
9	6	21

Performed with: **1a** (0.10 mmol), benzyl bromide (0.06 mmol), phase-transfer catalyst (2 mol%), **8** (10 mol%) and C_2CO_3 (0.10 mmol) in CH_2Cl_2/H_2O (0.35 mL/0.65 mL) at rt for 24 h. [a]Yields were determined by 1H-NMR with mesitylene as internal standard. [b]e.e. determined by chiral HPLC. (The e.e. of the substrate was not determined). [c] The yield and e.e of the side product **3aa**.

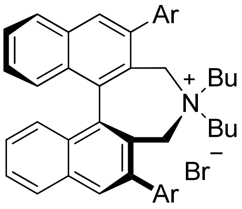

1) Ar = 3,5-$(Ph)_2$-C_6H_3
2) Ar = 3,5-$(^tBu)_2$-C_6H_3
3) Ar = 4-NO_2-C_6H_4
4) Ar = 4-CF_3-C_6H_4

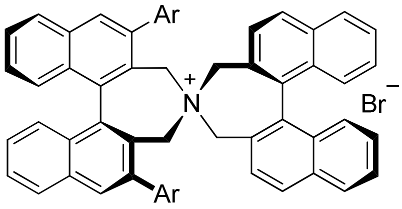

5) Ar = 3,5-$(CF_3)_2$-C_6H_3

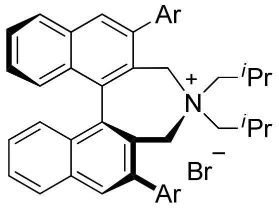

6) Ar = 3,5-(3,5-$(CF_3)_2$-$C_6H_3)_2$-C_6H_3

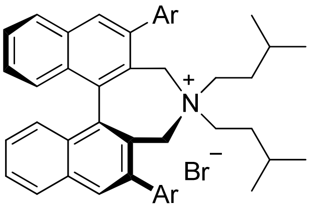

7) Ar = 3,5-$(CF_3)_2$-C_6H_3

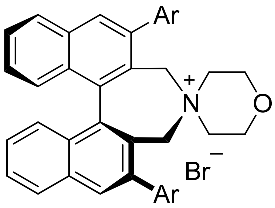

8) Ar = 3,5-(3,5-$(CF_3)_2$-$C_6H_3)_2$-C_6H_3
(14)

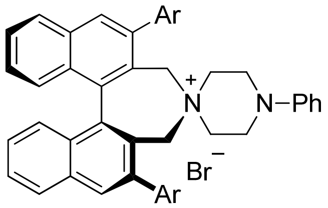

9) Ar = 3,5-(3,4,5-F_3-C_6H_2)-C_6H_3

Catalyst Synthesis (Scheme 1)

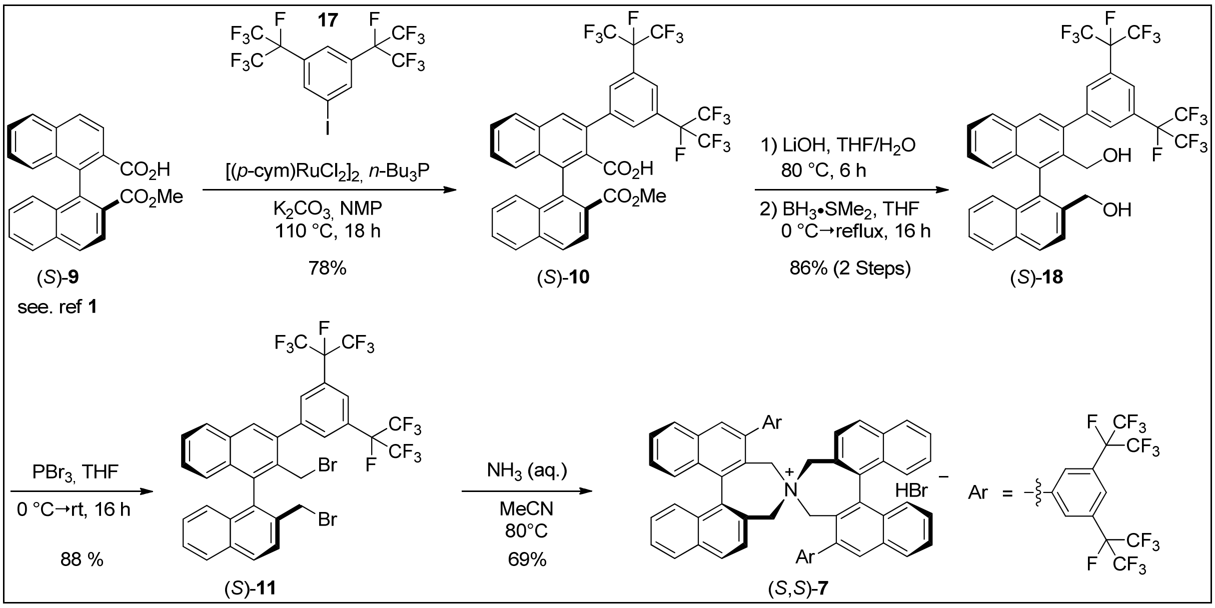

PTC (*S*)-Precursor **10**

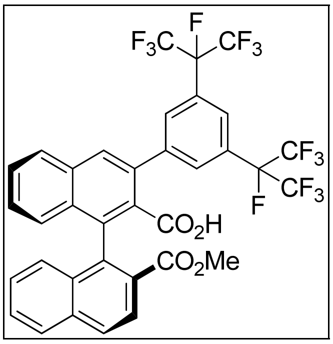

(*S*)-**9** (61 mg, 170 µmol), [(*p*-cym)RuCl$_2$]$_2$ (5.2 mg, 8.5 µmol) and K$_2$CO$_3$ (26 mg, 187 µmol) were dissolved in NMP under argon atmosphere. Afterwards NMP (1 mL), tributylphosphine (3.4 mg, 4.2 µL, 17 µmol) and aryl iodide **17** (119 mg, 221 µmol) were added and the mixture was pre-stirred at room temperature for 5 min before heated up to 110 °C for 18 h. After cooling down to room temperature HCl (1 N) was added, the phases were separated and the aqueous phase was extracted with EtOAc. The combined organic phases were washed with brine, dried over Na$_2$SO$_4$ and the solvent was removed *in vacuo*. The resulting residue was purified by column chromatography on silica gel (hexane:EtOAc = 4:1) to afford the title compound (*S*)-**10** (102 mg, 133 µmol, 78%) as a pale yellow solid.

^{1}H NMR (500 MHz, CDCl$_3$) δ = 8.08 (s, 2 H), 8.01 (t, *J* = 4.0, 2 H), 7.98 (s, 2 H), 7.94 (d, *J* = 8.2, 1 H), 7.88 (s, 1 H), 7.56 (ddd, *J* = 7.9, 6.7, 0.9, 2 H), 7.39 – 7.29 (m, 2 H), 7.23 (d, *J* = 8.5, 1 H), 6.98 (d, *J* = 8.5, 1 H), 3.64 (s, 3 H) ppm. ^{13}C NMR (125 MHz, CDCl$_3$) δ = 170.1, 168.5, 142.5 (t, *J* = 2.1), 136.7, 135.3, 135.1, 133.8, 133.2, 132.9, 132.2, 131.5, 130.0, 129.4, 129.1 (d, *J* = 10.3), 128.7, 128.5, 128.3, 128.3, 128.2 (d, *J* = 2.2), 128.1, 128.0, 127.7, 127.7, 126.5, 125.0, 122.3 (t, *J* = 11.7) 120.4 (qd, *J* = 286.1, 27.8), 91.3 (dq, *J* = 204.2, 33.3), 53.0 ppm. ^{19}F NMR (466 MHz, CDCl$_3$) δ = -75.26 – -75.38 (m), -181.68 – -181.82 (m) ppm. HRMS (ESI) exact mass calcd. for $C_{35}H_{17}F_{14}O_4$:

m/z 767.0909 ([M - H]⁻), found *m/z* 767.0757 ([M - H]⁻), IR (neat): 3065, 2360, 1730, 1280,1231, 1194 cm^{-1}. $[\alpha]_D^{27.1}$ = -17.9 (*c* = 1.0, $CHCl_3$).

PTC (*S*)-Precursor **18**

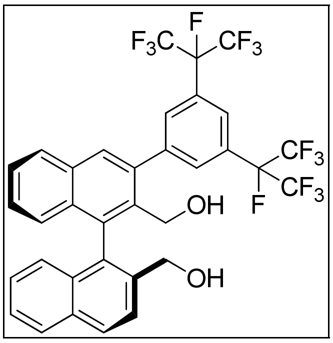

Ester **10** (102 mg, 133 µmol) was dissolved in THF/H_2O (1.5 mL/0.5 mL), lithium hydroxide monohydrate (27.9 mg, 665 µmol) was added and the reaction mixture was refluxed for 6 h. After cooling down to room temperature HCl (1 N) and CH_2Cl_2 were added, the phases were separated and the aqueous phase was extracted with CH_2Cl_2. The combined organic phases were dried over Na_2SO_4 and the solvent was removed *in vacuo*. The obtained dicarboxylic acid was dissolved in THF (2 mL) and BH_3•SMe_2 (60.6 mg, 76 µL, 798 µmol) was added dropwise at 0 °C under argon atmosphere and the resulting reaction mixture was refluxed for additional 16 h. After cooling down to 0 °C, water and HCl (1 N) were carefully added, the phases were separated and the aqueous phase was extracted with EtOAc. The combined organic phases were washed with brine, dried over Na_2SO_4 and the solvent was removed *in vacuo*. The resulting residue was purified by column chromatography on silica gel (hexane:EtOAc = 8:1) to afford the title compound (*S*)-**18** (83.1 mg, 115 µmol, 86%) as a white solid.

^{1}H NMR (500 MHz, $CDCl_3$) δ = 8.28 (s, 2 H), 8.05 – 7.86 (m, 5 H), 7.68 (d, *J* = 8.3, 1 H), 7.51 (dt, *J* = 28.5, 7.2, 2 H), 7.29 (dd, *J* = 13.2, 6.3, 2 H), 7.04 (t, *J* = 9.2, 2 H), 4.45 (d, *J* = 11.6, 1 H), 4.21 (d, *J* = 11.5, 1 H), 4.08 (dd, *J* = 27.7, 11.0, 2 H), 3.95 – 3.67 (m, 1 H) ppm. ^{13}C NMR (125 MHz, $CDCl_3$) δ = 143.1 (t, *J* = 2.1), 138.7, 137.6, 137.6, 134.4, 134.3, 133.2, 133.1, 133.0, 132.9, 130.3, 130.2 (d, *J* = 9.2), 129.1, 128.3, 128.3, 127.9 (dd, *J* = 20.8, 2.2), 127.8, 127.5, 127.3, 126.9, 126.6, 126.3, 126.1, 122.1 (t, *J* = 12.2), 120.5 (dq, *J* = 286.3, 27.6), 91.3 (ddt, *J* = 133.0, 66.8, 33.3), 63.1, 59.8 ppm. ^{19}F NMR (466 MHz, $CDCl_3$) δ = -75.30 (ddd, *J* = 22.0, 15.1, 7.7), -181.56 (dt, *J* = 14.4, 7.2) ppm. HRMS (ESI) exact mass calcd. for $C_{34}H_{20}F_{14}NaO_2$: *m/z* 749.1132 ([M + Na]⁺), found *m/z* 749.1143 ([M + Na]⁺), IR (neat): 3281, 1302, 1230, 1194, 985 cm^{-1}. $[\alpha]_D^{25.5}$ = -56.7 (*c* = 1.0, $CHCl_3$).

PTC (*S*)-Precursor **11**

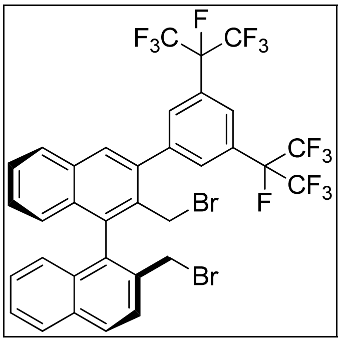

To a solution of (*S*)-**18** (44.1 mg, 60.7 µmol) in THF (1.5 mL) was added PBr_3 (36 mg, 13 µL, 134 µmol) at 0 °C and the resulting mixture was stirred at room temperature for additional 16 h. Afterwards aq. sat. $NaHCO_3$-sol was added, the phases were separated and the aqueous phase was extracted with EtOAc. The combined organic phases were washed with brine, dried over Na_2SO_4 and the solvent was removed *in vacuo*. The resulting residue was purified by column chromatography on silica gel (hexane:EtOAc = 100:1) to afford the title compound (*S*)-**11** (45.8 mg, 53.8 µmol, 88%) as colorless oil.

^{1}H NMR (500 MHz, $CDCl_3$) δ = 8.10 – 8.03 (m, 3 H), 7.98 – 7.91 (m, 4 H), 7.76 (d, *J* = 8.6, 1 H), 7.57 (dd, *J* = 8.1, 7.0, 1 H), 7.52 (dd, *J* = 8.1, 6.9, 1 H), 7.38 – 7.29 (m, 2 H), 7.16 (d, *J* = 8.5, 1 H), 7.12 (d, *J* = 8.6, 1 H), 4.29 (s, 2 H), 4.09 – 4.03 (m, 2 H) ppm. ^{13}C NMR (125 MHz, $CDCl_3$) δ = 142.6 (t, *J* = 2.1), 137.8, 136.6, 134.1, 134.1, 133.4, 133.0, 132.6, 132.3, 132.0, 130.7, 129.8 , 129.85 – 129.69 (m), 128.2 (dd, *J* = 20.8, 2.3), 128.2, 128.2, 127.9, 127.9, 127.7, 127.2, 127.0, 127.0, 127.0, 122.4 (d, *J* = 9.7), 120.5 (dq, *J* = 286.7, 27.8), 91.1 (dd, *J* = 204.3, 33.4), 33.0, 30.2 ppm. ^{19}F NMR (466 MHz, $CDCl_3$) δ = -75.23 (d, *J* = 7.2), -181.79 (dq, *J* = 14.2, 7.1) ppm. IR (neat): 1279, 1220, 1192, 984, 818 cm^{-1}. $[\alpha]_D^{26.7}$ = -51.5 (*c* = 0.9, $CHCl_3$).

PTC (*S*,*S*)-**7**

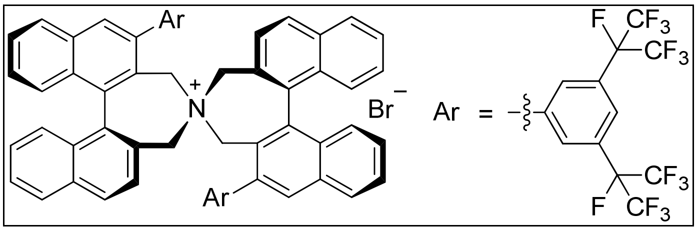

A solution of dibromide (*S*)-**11** (350 mg, 410 µmol) in CH_3CN (9 mL) was heated to 80 °C, aqueous ammonia (200 µL, 2.76 mmol) was added and the resulting mixture was stirred at 80 °C overnight. After cooling down to room temperature the solvent was removed *in vacuo* and the residue was purified by column chromatography on silica gel (CH_2Cl_2:MeOH = 10:1) to yield the title compound (*S,S*)-**7** (208 mg, 1.41 µmol, 69%) as white solid.

^{1}H NMR (500 MHz, $CDCl_3$) δ = 8.94 (s, 2 H), 8.20 (s, 4 H), 8.10 (d, *J* = 8.2, 2 H), 7.83 (d, *J* = 8.3, 2 H), 7.63 (t, *J* = 7.5, 2 H), 7.55 (t, *J* = 7.5, 2 H), 7.45 (s, 2 H), 7.35 – 7.26 (m, 4 H), 7.20 (d, *J* = 8.4, 2 H), 7.12 (d, *J* = 8.7, 2 H), 7.05 (d, *J* = 8.8, 2 H), 6.24 (d, *J* = 8.5, 2 H), 4.88 (d, *J* = 14.0, 2 H), 4.69 (d, *J* = 13.3, 2 H), 4.51 (d, *J* = 14.1, 2 H), 3.67 (d, *J* = 13.2, 2 H) ppm. ^{13}C NMR (125 MHz, $CDCl_3$) δ = 142.8 (t, *J* = 2.0), 140.5, 136.3, 135.9, 134.7, 134.0, 133.8, 131.7, 131.3, 130.8 – 130.5 (m), 130.8 – 130.5 (m), 129.3, 129.3, 128.8, 128.8, 128.6, 128.0, 127.8, 127.4, 127.1,

125.5, 125.3, 123.3 – 123.0 (m), 120.6, 62.4, 57.6 ppm. ^{19}F NMR (466 MHz, $CDCl_3$) δ = -74.36, -74.75, -74.94, -75.16, -180.60, -181.14 ppm. HRMS (ESI) exact mass calcd. for $C_{68}H_{36}F_{28}N$: *m/z* 1398.2395 ($[M + Na]^+$), found *m/z* 1398.2436 ($[M + Na]^+$). IR (neat): 1305, 1280, 1228, 1194, 985, 730 cm^{-1}. $[\alpha]_D^{28.4}$ = +61.0 (*c* = 1.05, $CHCl_3$).

General Procedure (Tables 2 and 3)

The racemic diols **1b**-**1o** where synthesized according to ref [3] from their corresponding styrene derivatives. The tertiary diols **12a**-**12j** were synthesized according to ref [4].

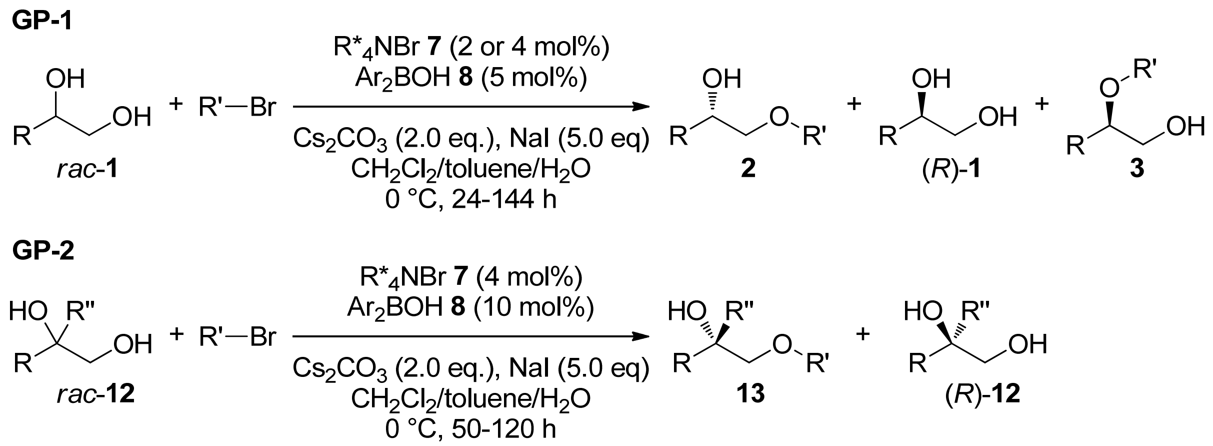

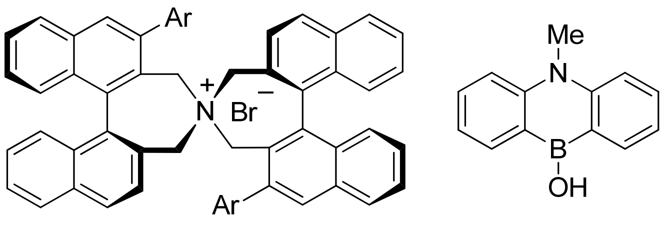

GP-1

To a mixture of diol (100 µmol, 1.0 equiv), borinic acid **8** (5 µmol, 5 mol%), PTC (*S,S*)-**7** (4 µmol, 4 mol%), NaI (500 µmol, 5.0 equiv) and Cs_2CO_3 (200 µmol, 2.0 equiv) in CH_2Cl_2:toluene:H_2O (0.15 mL : 0.20 mL : 0.65 mL) at 0 °C was added alkyl bromide (400 µmol, 4.0 equiv) and the resulting mixture was stirred for the stated period of time at that temperature. The reaction was quenched with EtOAc and aq. sat. NH_4Cl, the phases were separated and the aqueous phase was extracted with EtOAc. The combined organic phases were washed with brine, dried over Na_2SO_4 and the solvent was removed *in vacuo*. The resulting residue was purified by column chromatography on silica gel.

GP-2

To a mixture of diol (100 µmol, 1.0 equiv), borinic acid **8** (5 µmol, 5 mol%), PTC (*S,S*)-**7** (4 µmol, 4 mol%), NaI (500 µmol, 5.0 equiv) and Cs_2CO_3 (200 µmol, 2.0 equiv) in CH_2Cl_2:toluene:H_2O (0.15 mL : 0.20 mL : 0.65 mL) at 0 °C was added alkyl bromide (400 µmol, 4.0 equiv) and the resulting mixture was stirred for the stated period of time at that temperature. The reaction was quenched with EtOAc and aq. sat. NH_4Cl, the phases were separated and the aqueous phase was extracted with EtOAc. The combined organic phases were washed with brine, dried over Na_2SO_4 and the solvent was removed *in vacuo*. The resulting residue was dissolved in CH_2Cl_2 (1 mL) and H_2O_2 (15 µL, 35%) was added at room temperature and the resulting mixture was stirred for 1 h. Aq. sat. $Na_2S_2O_3$.-sol. was added, the phases were separated and the aqueous phase was extracted with CH_2Cl_2. The combined organic phases were dried over Na_2SO_4 and the solvent was removed *in vacuo*. The resulting residue was purified by column chromatography on silica gel.

Product Scope (Tables 2 and 3)

(*S*)-2-(Benzyloxy)-1-phenylethanol (**2aa**)

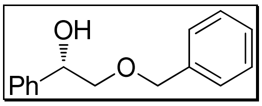

1-Phenylethane-1,2-diol (**1a**) (13.8 mg, 100 µmol), benzyl bromide (68 mg, 400 µmol), borinic acid **8** (1.1 mg, 5 µmol), PTC (*S,S*)-**7** (6.0 mg, 4 µmol), NaI (75 mg, 500 µmol) and Cs_2CO_3 (65 mg, 200 µmol) were reacted as described in **GP-1** for 96 h. The crude product mixture was purified by column chromatography on silica gel (hexane:EtOAc = 10:1 → 1:1) to obtain the title compound **2aa** (10.0 mg, 44%, 91% ee) as colorless oil along with the corresponding side-product **3aa** (1.4 mg, 6%) as colorless oil and substrate **1a** (6.7 mg, 49%, 74% ee) as white solid.

The enantiomeric purity of the product was determined by HPLC analysis (Daicel CHIRALPAK AD3, hexane/*i*PrOH = 60:1, flow rate = 0.5 mL/min, retention time; 63.1 min (major) and 66.6 min (minor)).

The enantiomeric purity of the substrate was determined by HPLC analysis (Daicel CHIRALPAK AD3, hexane/EtOH = 15:1, flow rate = 0.5 mL/min, retention time; 39.5 min (minor) and 43.8 min (major)).

^{1}H NMR (400 MHz, $CDCl_3$) δ = 7.40 – 7.24 (m, 10 H), 4.97 – 4.89 (m, 1 H), 4.66 – 4.55 (m, 2 H), 3.64 (dd, *J* = 9.8, 3.2, 1 H), 3.51 (t, *J* = 9.3, 1 H), 2.82 (d, *J* = 2.1, 1 H) ppm. ^{13}C NMR (100 MHz, $CDCl_3$) δ = 140.4, 138.0, 128.7, 128.6, 128.1, 128.0, 126.4, 76.0, 73.6, 73.0 ppm. HRMS (ESI) exact mass calcd. for $C_{15}H_{16}NaO_2$: *m/z* 251.1043 ([M + Na]$^+$), found *m/z* 251.1043 ([M + Na]$^+$). IR (neat): 3437, 2859, 1453, 1198, 1104, 1069 cm^{-1}. $[\alpha]_D^{28.6}$ = +23.0 (*c* = 1.0, $CHCl_3$).

The absolute configuration of the product was deduced by comparing the optical rotation of the recovered substrate **2a** with commercial (*R*)- and (*S*)-**2a**.

(*S*)-2-((2-Methylbenzyl)oxy)-1-phenylethanol (**2ab**)

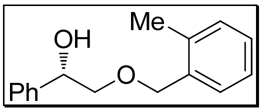

1-Phenylethane-1,2-diol (**1a**) (13.8 mg, 100 µmol), 1-(bromomethyl)-2-methylbenzene (74 mg, 400 µmol), borinic acid **8** (1.1 mg, 5 µmol), PTC (*S,S*)-**7** (3.0 mg, 2 µmol), NaI (75 mg, 500 µmol) and Cs_2CO_3 (65 mg, 200 µmol) were reacted as described in **GP-1** for 90 h. The crude product mixture was purified by column chromatography on silica gel (hexane:EtOAc = 10:1 → 1:1) to obtain the title compound **2ab** (12.4 mg, 51%, 87% ee) as white solid along with the corresponding side-product **3ab** (1.0 mg, 4%) as colorless oil and substrate **1a** in 40% yield (5.5 mg, 40%, 94% ee) as white solid.

The enantiomeric purity of the product was determined by HPLC analysis (Daicel CHIRALPAK AD3, hexane/*i*PrOH = 60:1, flow rate = 0.5 mL/min, retention time; 52.1 min (major) and 54.8 min (minor)).

The enantiomeric purity of the substrate was determined by HPLC analysis (Daicel CHIRALPAK ADH, hexane/EtOH = 20:1, flow rate = 1.0 mL/min, retention time; 27.0 min (minor) and 29.9 min (major)).

^{1}H NMR (400 MHz, $CDCl_3$) δ = 7.41 – 7.15 (m, 9 H), 4.93 (dt, *J* = 8.9, 2.5, 1 H), 4.64 – 4.56 (m, 2 H), 3.66 (dd, *J* = 9.7, 3.2, 1 H), 3.55 – 3.49 (m, 1 H), 2.78 (d, *J* = 2.3, 1 H), 2.34 (s, 3 H) ppm. ^{13}C NMR (100 MHz, $CDCl_3$) δ = 140.5, 137.0, 136.9, 135.9, 130.6, 129.0, 128.6, 128.3, 128.0, 126.4, 126.1, 76.0, 73.0, 72.0, 18.9 ppm. HRMS (ESI) exact mass calcd. for $C_{16}H_{18}NaO_2$: *m/z* 265.1199 ([M + Na]$^+$), found *m/z* 265.1201 ([M + Na]$^+$). IR (neat): 3446, 2915, 1494 1456, 1069 cm^{-1}. $[\alpha]_{D}^{28.6}$ = +4.5 (*c* = 1.24, $CHCl_3$).

(*S*)-2-((3-Methylbenzyl)oxy)-1-phenylethanol (**2ac**)

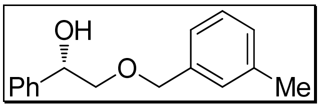

1-Phenylethane-1,2-diol (**1a**) (13.8 mg, 100 µmol), 1-(bromomethyl)-3-methylbenzene (74 mg, 400 µmol), borinic acid **8** (1.1 mg, 5 µmol), PTC (*S,S*)-**7** (6.0 mg, 4 µmol), NaI (75 mg, 500 µmol) and Cs_2CO_3 (65 mg, 200 µmol) were reacted as described in **GP-1** for 94 h. The crude product mixture was purified by column chromatography on silica gel (hexane:EtOAc = 10:1 → 1:1) to obtain the title compound **2ac** (11.1 mg, 46%, 82% ee) as colorless oil along with the corresponding side-product **3ac** (3.1 mg, 13%) as colorless oil and substrate **1a** (4.2 mg, 30%, 90% ee) as white solid.

The enantiomeric purity of the product was determined by HPLC analysis (Daicel CHIRALPAK IE, hexane/*i*PrOH = 60:1, flow rate = 0.5 mL/min, retention time; 46.3 min (major) and 51.1 min (minor)).

The enantiomeric purity of the substrate was determined by HPLC analysis (Daicel CHIRALPAK ADH, hexane/EtOH = 20:1, flow rate = 1.0 mL/min, retention time; 27.0 min (minor) and 29.9 min (major)).

^{1}H NMR (400 MHz, $CDCl_3$) δ = 7.41 – 7.31 (m, 4 H), 7.31 – 7.21 (m, 2 H), 7.17 – 7.09 (m, 3 H), 4.93 (dd, *J* = 8.9, 2.9, 1 H), 4.62 – 4.51 (m, 2 H), 3.64 (dd, *J* = 9.8, 3.2, 1 H), 3.50 (t, *J* = 9.4, 1 H), 2.83 (s, 1 H), 2.35 (s, 3 H) ppm. ^{13}C NMR (100 MHz, $CDCl_3$) δ = 140.4, 138.4, 137.9, 128.8, 128.6, 128.6, 128.0, 126.4, 125.1, 76.0, 73.6, 73.0, 21.5 ppm. HRMS (ESI) exact mass calcd. for $C_{16}H_{18}NaO_2$: *m/z* 265.1199 ([M + Na]$^+$), found *m/z* 265.1199 ([M + Na]$^+$). IR (neat): 3420, 2857, 1453, 1105, 1068, 698 cm^{-1}. $[\alpha]_{D}^{28.0}$ = +18.6 (*c* = 1.16, $CHCl_3$).

(*S*)-2-((4-Methylbenzyl)oxy)-1-phenylethanol (**2ad**)

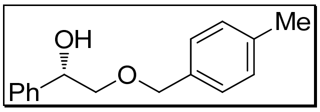

1-Phenylethane-1,2-diol (**1a**) (13.8 mg, 100 µmol), 1-(bromomethyl)-4-methylbenzene (74 mg, 400 µmol), borinic acid **8** (1.1 mg, 5 µmol), PTC (*S,S*)-**7** (3.0 mg, 2 µmol), NaI (75 mg, 500 µmol) and Cs_2CO_3 (65 mg, 200 µmol) were reacted as described in **GP-1** for 96 h. The crude product mixture was purified by column chromatography on silica gel (hexane:EtOAc = 10:1 → 1:1) to obtain the title compound **2ad** (11.5 mg, 48%, 87% ee) as white solid along with substrate **1a** (6.5 mg, 47%, 80% ee) as a white solid.

The enantiomeric purity of the product was determined by HPLC analysis (Daicel CHIRALPAK AD3, hexane/*i*PrOH = 60:1, flow rate = 0.5 mL/min, retention time; 60.0 min (major) and 63.6 min (minor)).

The enantiomeric purity of the substrate was determined by HPLC analysis (Daicel CHIRALPAK AD3, hexane/EtOH = 15:1, flow rate = 0.5 mL/min, retention time; 39.5 min (minor) and 43.8 min (major)).

^{1}H NMR (400 MHz, $CDCl_3$) δ = 7.39 – 7.31 (m, 4 H), 7.31 – 7.20 (m, 3 H), 7.16 (d, *J* = 7.9, 2 H), 4.91 (dt, *J* = 8.9, 2.5, 1 H), 4.60 – 4.51 (m, 2 H), 3.62 (dd, *J* = 9.7, 3.2, 1 H), 3.48 (t, *J* = 9.4, 1 H), 2.83 (d, *J* = 2.2, 1 H), 2.35 (s, 3 H) ppm. ^{13}C NMR (100 MHz, $CDCl_3$) δ = 145.4, 140.4, 137.8, 134.9, 129.4, 128.6, 128.1, 128.0, 126.4, 75.8, 73.4, 73.0, 21.3 ppm. HRMS (ESI) exact mass calcd. for $C_{16}H_{18}NaO_2$: *m/z* 265.1199 ([M + Na]$^+$), found *m/z* 265.1202 ([M + Na]$^+$). IR (neat): 3437, 2858, 1452, 1101, 803, 753 cm^{-1}. $[\alpha]_D^{28.4}$ = +22.8 (*c* = 1.50, $CHCl_3$).

(*S*)-2-((2-Fluorobenzyl)oxy)-1-phenylethanol (**2ae**)

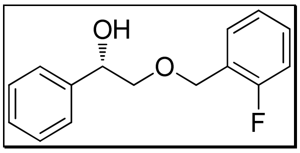

1-Phenylethane-1,2-diol (**1a**) (13.8 mg, 100 µmol), 1-(bromomethyl)-2-fluorobenzene (76 mg, 400 µmol), borinic acid **8** (1.1 mg, 5 µmol), PTC (*S,S*)-**7** (6.0 mg, 4 µmol), NaI (75 mg, 500 µmol) and Cs_2CO_3 (65 mg, 200 µmol) were reacted as described in **GP-1** for 120 h. The crude product mixture was purified by column chromatography on silica gel (hexane:EtOAc = 10:1 → 1:1) to obtain the title compound **2ae** (11.6 mg, 47%, 90% ee) as colorless oil along with the corresponding side-product **3ae** (1.0 mg, 4%) as colorless oil and substrate **1a** (6.2 mg, 45%, 73% ee) as white solid.
The enantiomeric purity of the product was determined by HPLC analysis (Daicel CHIRALPAK AD3, hexane/*i*PrOH = 60:1, flow rate = 0.5 mL/min, retention time; 65.4 min (major) and 69.1 min (minor)).
The enantiomeric purity of the substrate was determined by HPLC analysis (Daicel CHIRALPAK ADH, hexane/EtOH = 20:1, flow rate = 1.0 mL/min, retention time; 27.0 min (minor) and 29.9 min (major)).

^{1}H NMR (500 MHz, $CDCl_3$) δ = 7.42 – 7.32 (m, 5 H), 7.32 – 7.26 (m, 2 H), 7.17 – 7.11 (m, 1 H), 7.08 – 7.03 (m, 1 H), 4.93 (dt, *J* = 9.0, 2.6, 1 H), 4.67 (s, 2 H), 3.67 (dd, *J* = 9.8, 3.2, 1 H), 3.54 (t, *J* = 9.4, 1 H), 2.81 (d, *J* = 2.2, 1 H) ppm. ^{13}C NMR (125 MHz, $CDCl_3$) δ = 161.0 (d, *J* = 247.1), 140.1, 130.3 (d, *J* = 4.4), 129.8 (d, *J* = 8.3), 128.5, 120.0, 126.3, 124.9 (d, *J* = 14.8), 124.3 (d, *J* = 3.8), 115.5 (d, *J* = 21.5), 76.1, 72.9, 67.0 (d, *J* = 3.8) ppm. ^{19}F NMR (466 MHz, $CDCl_3$) δ = -118.7 (m) ppm. HRMS (ESI) exact mass calcd. for $C_{15}H_{15}FNaO_2$: *m/z* 269.0948 ([M + Na]$^+$), found *m/z* 269.0950 ([M + Na]$^+$). IR (neat): 3446, 2864, 1490, 1473, 1229, 1090, 756 cm^{-1}. $[\alpha]_D^{28.6}$ = –8.4 (*c* = 1.16, $CHCl_3$).

(*S*)-2-((2-Bromobenzyl)oxy)-1-phenylethanol (**2af**)

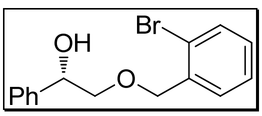

1-Phenylethane-1,2-diol (**1a**) (13.8 mg, 100 µmol), 1-bromo-2-(bromomethyl)benzene (100 mg, 400 µmol), borinic acid **8** (1.1 mg, 5 µmol), PTC (*S,S*)-**7** (6.0 mg, 4 µmol), NaI (75 mg, 500 µmol) and Cs_2CO_3 (65 mg, 200 µmol) were reacted as described in **GP-1** for 78 h. The crude product mixture was purified by column chromatography on silica gel (hexane:EtOAc = 10:1 → 1:1) to obtain the title compound **2af** (13.8 mg, 45%,

85% ee) as white solid along with the corresponding side-product **3af** (1.5 mg, 5%) as colorless oil and substrate **1a** (5.8 mg, 42%, 71% ee) as white solid.

The enantiomeric purity of the product was determined by HPLC analysis (Daicel CHIRALPAK IC3, hexane/*i*PrOH = 60:1, flow rate = 1.0 mL/min, retention time; 22.0 min (minor) and 26.5 min (major)).

The enantiomeric purity of the substrate was determined by HPLC analysis (Daicel CHIRALPAK ADH, hexane/EtOH = 20:1, flow rate = 1.0 mL/min, retention time; 27.0 min (minor) and 29.9 min (major)).

^{1}H NMR (400 MHz, $CDCl_3$) δ = 7.55 (d, *J* = 8.0, 1 H), 7.44 (d, *J* = 7.7, 1 H), 7.42 – 7.24 (m, 6 H), 7.17 (t, *J* = 7.6, 1 H), 4.97 (d, *J* = 8.7, 1 H), 4.71 – 4.62 (m, 2 H), 3.72 (ddd, *J* = 9.8, 3.2, 0.7, 1 H), 3.64 – 3.55 (m, 1 H), 2.87 (d, *J* = 1.6, 1 H) ppm. ^{13}C NMR (100 MHz, $CDCl_3$) δ = 140.3, 137.3, 132.9, 129.6, 129.5, 128.6, 128.1, 127.7, 126.4, 123.3, 76.4, 73.0, 72.8, 11.3 ppm. HRMS (ESI) exact mass calcd. for $C_{15}H_{15}BrNaO_2$: *m/z* 329.0148 ([M + Na]$^+$), found *m/z* 329.0160 ([M + Na]$^+$). IR (neat): 3420, 2859, 1439, 1104, 1025, 748 cm^{-1}. $[\alpha]_D^{27.7}$ = +24.0 (*c* = 1.38, $CHCl_3$).

(*S*)-2-((2-Hydroxy-2-phenylethoxy)methyl)benzonitrile (**2ag**)

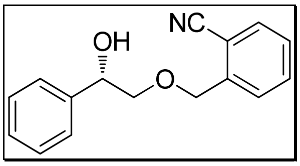

1-Phenylethane-1,2-diol (**1a**) (13.8 mg, 100 µmol), 2-(bromomethyl)benzonitrile (78 mg, 400 µmol), borinic acid **8** (1.1 mg, 5 µmol), PTC (*S,S*)-**7** (6.0 mg, 4 µmol), NaI (75 mg, 500 µmol) and Cs_2CO_3 (65 mg, 200 µmol) were reacted as described in **GP-1** for 115 h. The crude product mixture was purified by column chromatography on silica gel (hexane:EtOAc = 7:1 → 1:1) to obtain the title compound **2ag** (12.4 mg, 49%, 87% ee) as colorless oil along with the corresponding side-product **3ag** (1.3 mg, 5%) as colorless oil and substrate **1a** (6.0 mg, 43%, 57% ee) as white solid.

The enantiomeric purity of the product was determined by HPLC analysis (Daicel CHIRALPAK ADH, hexane/EtOH = 15:1, flow rate = 1.0 mL/min, retention time; 37.1 min (minor) and 41.5 min (major)).

The enantiomeric purity of the substrate was determined by HPLC analysis (Daicel CHIRALPAK AD3, hexane/EtOH = 15:1, flow rate = 1.0 mL/min, retention time; 19.9 min (minor) and 21.9 min (major)).

^{1}H NMR (400 MHz, $CDCl_3$) δ = 7.67 (d, *J* = 7.7, 1 H), 7.59 (t, *J* = 7.6, 1 H), 7.53 (d, *J* = 7.6, 1 H), 7.44 – 7.27 (m, 6 H), 4.99 (d, *J* = 8.7, 1 H), 4.78 (s, 2 H), 3.73 (dd, *J* = 9.7, 3.1, 1 H), 3.62 (t, *J* = 9.3, 1 H), 2.91 (d, *J* = 1.8, 1 H) ppm. ^{13}C NMR (100 MHz, $CDCl_3$) δ = 141.7, 140.2, 133.1, 133.1, 128.9, 128.6, 128.5, 128.1, 126.4, 117.7, 111.8, 76.7, 72.9, 71.1 ppm. HRMS (ESI) exact mass calcd. for $C_{16}H_{15}NNaO_2$: *m/z* 276.0995 ([M + Na]$^+$), found *m/z* 276.1000 ([M + Na]$^+$). IR (neat): 3459, 2862, 2225, 1451, 1087, 760 cm^{-1}. $[\alpha]_D^{27.8}$ = +26.1 (*c* = 1.10, $CHCl_3$).

(*S*)-2-([1,1'-Biphenyl]-4-ylmethoxy)-1-phenylethanol (**2ah**)

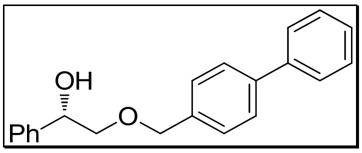

1-Phenylethane-1,2-diol (**1a**) (13.8 mg, 100 µmol), 4-(bromomethyl)-1,1'-biphenyl (99 mg, 400 µmol), borinic acid **8** (1.1 mg, 5 µmol), PTC (*S,S*)-7 (6.0 mg, 4 µmol), NaI (75 mg, 500 µmol) and Cs_2CO_3 (65 mg, 200 µmol) were reacted as described in **GP-1** for 91 h. The crude product mixture was purified by column chromatography on silica gel (hexane:EtOAc = 10:1 → 1:1) to obtain the title compound **2ah** (13.9 mg, 46%, 91% ee) as white solid along with the corresponding side-product **3ah** (4.6 mg, 15%) as colorless oil and substrate **1a** (5.1 mg, 37%, 79% ee) as white solid.

The enantiomeric purity of the product was determined by HPLC analysis (Daicel CHIRALPAK ADH, hexane/EtOH = 15:1, flow rate = 1.5 mL/min, retention time; 17.0 min (minor) and 19.5 min (major)).

The enantiomeric purity of the substrate was determined by HPLC analysis (Daicel CHIRALPAK AD3, hexane/EtOH = 15:1, flow rate = 0.5 mL/min, retention time; 39.5 min (minor) and 43.8 min (major)).

^{1}H NMR (400 MHz, $CDCl_3$) δ = 7.59 (m, 4 H), 7.47 – 7.26 (m, 11 H), 4.96 (d, *J* = 8.9, 1 H), 4.70 – 4.59 (m, 2 H), 3.68 (dd, *J* = 9.7, 3.2, 1 H), 3.54 (t, *J* = 9.3, 1 H), 2.81 (d, *J* = 2.0, 1 H) ppm. ^{13}C NMR (100 MHz, $CDCl_3$) δ = 141.1,

141.0, 140.4, 137.0, 129.0, 128.6, 128.5, 128.1, 127.6, 127.5, 127.3, 126.4, 76.0, 73.3, 73.0 ppm. HRMS (ESI) exact mass calcd. for $C_{21}H_{20}NaO_2$: *m/z* 327.1356 ([M + Na]$^+$), found *m/z* 327.1361 ([M + Na]$^+$). IR (neat): 3446, 2856, 1452, 1102, 698 cm^{-1}. $[\alpha]_D^{28.6}$ = +14.6 (*c* = 1.39, $CHCl_3$).

(*S*)-2-((3,5-Bis(trifluoromethyl)benzyl)oxy)-1-phenylethanol (**2ai**)

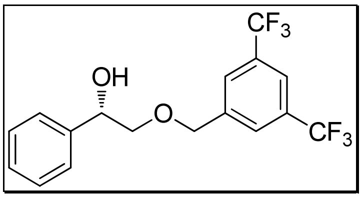

1-Phenylethane-1,2-diol (**1a**) (13.8 mg, 100 µmol), 1-(bromomethyl)-3,5-bis(trifluoromethyl)benzene (123 mg, 400 µmol), borinic acid **8** (1.1 mg, 5 µmol), PTC (*S,S*)-**7** (6.0 mg, 4 µmol), NaI (75 mg, 500 µmol) and Cs_2CO_3 (65 mg, 200 µmol) were reacted as described in **GP-1** for 120 h. The crude product mixture was purified by column chromatography on silica gel (hexane:EtOAc = 5:1 → 1:1) to obtain the title compound **2ai** (13.1 mg, 36%, 89% ee) as colorless oil along with the corresponding side-product **3ai** (9.5 mg, 26%) as colorless oil and substrate **1a** (4.8 mg, 35%, 70% ee) as white solid.
The enantiomeric purity of the product was determined by HPLC analysis (Daicel CHIRALPAK AD3, hexane/*i*PrOH = 60:1, flow rate = 1.0 mL/min, retention time; 18.6 min (major) and 35.4 min (minor)).
The enantiomeric purity of the substrate was determined by HPLC analysis (Daicel CHIRALPAK ADH, hexane/EtOH = 20:1, flow rate = 1.0 mL/min, retention time; 27.0 min (minor) and 29.9 min (major)).

^{1}H NMR (400 MHz, $CDCl_3$) δ = 7.80 (d, *J* = 11.6, 3 H), 7.42 – 7.29 (m, 5 H), 4.99 (dt, *J* = 8.3, 2.9, 1 H), 4.74 – 4.66 (m, 2 H), 3.71 (dd, *J* = 9.8, 3.4, 1 H), 3.63 (dd, *J* = 9.7, 8.4, 1 H), 2.62 (d, *J* = 2.7, 1 H) ppm. ^{13}C NMR (125 MHz, $CDCl_3$) δ = 140.7, 140.1, 131.9 (q, *J* = 33.4), 128.6, 128.2, 127.50 – 127.4 (m), 126.2, 123.4 (q, *J* = 272.7), 121.8 (dt, *J* = 7.7, 3.8), 76.5, 73.1, 72.0 ppm. ^{19}F NMR (466 MHz, $CDCl_3$) δ = -62.75 ppm. HRMS (ESI) exact mass calcd. for $C_{17}H_{14}F_6NaO_2$: *m/z* 387.0790 ([M + Na]$^+$), found *m/z* 387.0793 ([M + Na]$^+$). IR (neat): 3420, 2915, 1357, 1278, 1174, 1130 cm^{-1}. $[\alpha]_D^{28.4}$ = +12.5 (*c* = 1.18, $CHCl_3$).

(*S*)-2-(Naphthalen-2-ylmethoxy)-1-phenylethanol (**2aj**)

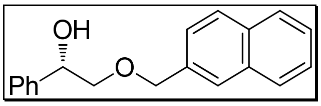

1-Phenylethane-1,2-diol (**1a**) (13.8 mg, 100 µmol), 2-(bromomethyl)naphthalene (88 mg, 400 µmol), borinic acid **8** (1.1 mg, 5 µmol), PTC (*S,S*)-**7** (6.0 mg, 4 µmol), NaI (75 mg, 500 µmol) and Cs_2CO_3 (65 mg, 200 µmol) were reacted as described in **GP-1** for 96 h. The crude product mixture was purified by column chromatography on silica gel (hexane:EtOAc = 10:1 → 1:1) to obtain the title compound **2aj** (10.0 mg, 36%, 91% ee) as white solid along with the corresponding side-product **3aj** (3.7 mg, 13%) as colorless oil and substrate **1a** (6.6 mg, 48%, 64% ee) as white solid.

The enantiomeric purity of the product was determined by HPLC analysis (Daicel CHIRALPAK ADH, hexane/EtOH = 50:1, flow rate = 1.5 mL/min, retention time; 49.5 min (minor) and 55.3 min (major)).
The enantiomeric purity of the substrate was determined by HPLC analysis (Daicel CHIRALPAK ADH, hexane/EtOH = 20:1, flow rate = 1.0 mL/min, retention time; 27.0 min (minor) and 29.9 min (major)).

^{1}H NMR (400 MHz, $CDCl_3$) δ = 7.82 (m, 3 H), 7.76 (s, 1 H), 7.51 – 7.43 (m, 3 H), 7.40 – 7.23 (m, 5 H), 4.95 (d, *J* = 8.8, 1 H), 4.80 – 4.71 (m, 2 H), 3.68 (dd, *J* = 9.7, 3.2, 1 H), 3.55 (t, *J* = 9.3, 1 H), 2.83 (s, 1 H) ppm. ^{13}C NMR (100 MHz, $CDCl_3$) δ = 140.4, 135.5, 133.5, 133.3, 128.6, 128.5, 128.1, 128.0, 127.9, 126.8, 126.4, 126.2, 125.9, 75.9. 73.7, 73.0 ppm. HRMS (ESI) exact mass calcd. for $C_{19}H_{18}NaO_2$: *m/z* 301.1199 ([M + Na]$^+$), found *m/z* 301.1206 ([M + Na]$^+$). IR (neat): 3426, 2857, 1452, 1071, 900, 751 cm^{-1}. $[\alpha]_D^{28.6}$ = +14.6 (*c* = 1.0, $CHCl_3$).

(*S*)-2-((3-Methylbut-2-en-1-yl)oxy)-1-phenylethanol (**2ak**)

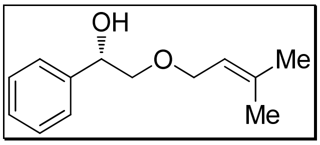

1-Phenylethane-1,2-diol (**1a**) (13.8 mg, 100 µmol), 1-bromo-3-methylbut-2-ene (60 mg, 400 µmol), borinic acid **8** (1.1 mg, 5 µmol), PTC (*S,S*)-**7** (6.0 mg, 4 µmol), NaI (75 mg, 500 µmol) and Cs_2CO_3 (65 mg, 200 µmol) were reacted as described in **GP-1** for 96 h. The crude product mixture was purified by column chromatography on silica gel (hexane:EtOAc = 10:1 → 1:1) to obtain the title compound **2ak** (8.3 mg, 40%, 89% ee) as colorless oil along with the corresponding side-product **3ak** (2.0 mg, 10%) as colorless oil and substrate **1a** (5.2 mg, 38%, 84%) as white solid.
The enantiomeric purity of the product was determined by HPLC analysis (Daicel CHIRALPAK AD3, hexane/*i*PrOH = 60:1, flow rate = 1.0 mL/min, retention time; 15.7 min (major) and 16.2 min (minor)).
The enantiomeric purity of the substrate was determined by HPLC analysis (Daicel CHIRALPAK ADH, hexane/EtOH = 20:1, flow rate = 1.0 mL/min, retention time; 27.0 min (minor) and 29.9 min (major)).

^{1}H NMR (400 MHz, $CDCl_3$) δ = 7.42 – 7.24 (m, 5 H), 5.37 (t, *J* = 6.8, 1 H), 4.89 (d, *J* = 9.1, 1 H), 4.06 (d, *J* = 6.9, 2 H), 3.60 (dd, *J* = 9.8, 3.1, 1 H), 3.42 (t, *J* = 9.5, 1 H), 2.84 (d, *J* = 2.0, 1 H), 1.76 (s, 3 H), 1.68 (s, 3 H) ppm. ^{13}C NMR (100 MHz, $CDCl_3$) δ = 140.6, 137.7, 128.6, 128.0, 126.4, 120.9, 75.7, 73.0, 67.8, 25.9, 18.1 ppm. HRMS (ESI) exact mass calcd. for $C_{13}H_{18}NaO_2$: *m/z* 229.1199 ([M + Na]$^+$), found *m/z* 229.1197 ([M + Na]$^+$). IR (neat): 3446, 2856, 1451, 1069, 1005, 699 cm^{-1}. $[\alpha]_D^{27.6}$ = +35.3 (*c* = 0.83, $CHCl_3$).

(*S*)-2-(Cinnamyloxy)-1-phenylethanol (**2al**)

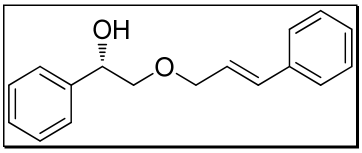

1-Phenylethane-1,2-diol (**1a**) (13.8 mg, 100 µmol), (*E*)-(3-bromoprop-1-en-1-yl)benzene (79 mg, 400 µmol), borinic acid **8** (1.1 mg, 5 µmol), PTC (*S,S*)-**7** (6.0 mg, 4 µmol), NaI (75 mg, 500 µmol) and Cs_2CO_3 (65 mg,

200 µmol) were reacted as described in **GP-1** for 24 h. The crude product mixture was purified by column chromatography on silica gel (hexane:EtOAc = 6:1 → 1:1) to obtain the title compound **2al** (10.0 mg, 39%, 84% ee) as pale yellow solid along with the corresponding side-product **3al** (8.2 mg, 32%) as colorless oil and substrate **1a** (3.8 mg, 27%, 84% ee) as white solid.

The enantiomeric purity of the product was determined by HPLC analysis (Daicel CHIRALPAK IC3, hexane/*i*PrOH = 60:1, flow rate = 1.0 mL/min, retention time; 37.3 min (minor) and 39.4 min (major)).

The enantiomeric purity of the substrate was determined by HPLC analysis (Daicel CHIRALPAK ADH, hexane/EtOH = 20:1, flow rate = 1.0 mL/min, retention time; 27.0 min (minor) and 29.9 min (major)).

^{1}H NMR (500 MHz, $CDCl_3$) δ = 7.42 – 7.23 (m, 10 H), 6.60 (d, *J* = 15.9, 1 H), 6.30 (dt, *J* = 15.9, 6.1, 1 H), 4.94 (d, *J* = 8.3, 1 H), 4.29 – 4.20 (m, 2 H), 3.66 (dd, *J* = 9.8, 3.2, 1 H), 3.51 (dd, *J* = 9.7, 9.1, 1 H), 2.80 (d, *J* = 1.9, 1 H) ppm. ^{13}C NMR (125 MHz, $CDCl_3$) δ = 140.3, 136.6, 133.1, 128.7, 128.5, 128.0, 127.9, 126.6, 126.3, 125.6, 75.8, 73.0, 72.0 ppm. HRMS (ESI) exact mass calcd. for $C_{17}H_{18}NaO_2$: *m/z* 277.1199 ([M + Na]$^+$), found *m/z* 277.1201 ([M + Na]$^+$). IR (neat): 3423, 2855, 1495, 1110, 968, 700 cm^{-1}. $[\alpha]_D^{27.5}$ = +17.7 (*c* = 1.10, $CHCl_3$).

(*S*,*E*)-Methyl 4-(2-hydroxy-2-phenylethoxy)but-2-enoate (**2am**)

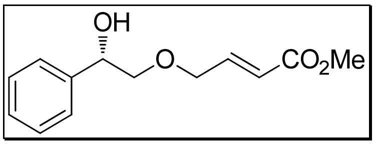

1-Phenylethane-1,2-diol (**1a**) (13.8 mg, 100 µmol), (E)-methyl 4-bromobut-2-enoate (72 mg, 400 µmol), borinic acid **8** (1.1 mg, 5 µmol), PTC (*S,S*)-**7** (6.0 mg, 4 µmol), NaI (75 mg, 500 µmol) and Cs_2CO_3 (65 mg, 200 µmol) were reacted as described in **GP-1** for 144 h. The crude product mixture was purified by column chromatography on silica gel (hexane:EtOAc = 5:1 → 1:1) to obtain the title compound **2am** (9.2 mg, 39%, 80% ee) as colorless oil along with the corresponding side-product **3am** (4.1 mg, 17%) as colorless oil and substrate **1a** (4.6 mg, 33%, 77% ee) as white solid.

The enantiomeric purity of the product was determined by HPLC analysis (Daicel CHIRALPAK OD3, hexane/*i*PrOH = 20:1, flow rate = 1.0 mL/min, retention time; 31.7 min (major) and 42.4 min (minor)).

The enantiomeric purity of the substrate was determined by HPLC analysis (Daicel CHIRALPAK ADH, hexane/EtOH = 20:1, flow rate = 1.0 mL/min, retention time; 27.0 min (minor) and 29.9 min (major)).

^{1}H NMR (400 MHz, $CDCl_3$) δ = 7.42 – 7.27 (m, 5 H), 6.96 (dt, *J* = 15.8, 4.4, 1 H), 6.09 (dt, *J* = 15.8, 1.9, 1 H), 4.93 (dt, *J* = 8.6, 2.6, 1 H), 4.23 (dd, *J* = 4.4, 1.9, 2 H), 3.75 (s, 3H), 3.65 (dd, *J* = 9.7, 3.3, 1 H), 3.58 – 3.48 (m, 1 H), 2.71 (d, *J* = 2.4, 1 H) ppm. ^{13}C NMR (100 MHz, $CDCl_3$) δ = 166.8, 144.2, 140.2, 128.7, 128.2, 126.3, 121.5, 76.5, 73.0, 67.0, 51.8 ppm. HRM*S* (ESI) exact mass calcd. for $C_{13}H_{16}NaO_4$: *m/z* 259.0941 ([M + Na]$^+$), found *m/z* 259.0943 ([M + Na]$^+$). IR (neat): 3446, 2852, 1718, 1304, 1132, 1026, 701 cm^{-1}. $[\alpha]_D^{28.5}$ = +31.6 (*c* = 0.33, $CHCl_3$).

(*S*)-1-Phenyl-2-((3-phenylprop-2-yn-1-yl)oxy)ethanol (**2an**)

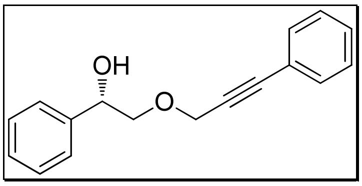

1-Phenylethane-1,2-diol (**1a**) (13.8 mg, 100 µmol), (3-bromoprop-1-yn-1-yl)benzene (78 mg, 400 µmol), borinic acid **8** (1.1 mg, 5 µmol), PTC (*S,S*)-**7** (6.0 mg, 4 µmol), NaI (75 mg, 500 µmol) and Cs_2CO_3 (65 mg, 200 µmol) were reacted as described in **GP-1** for 144 h. The crude product mixture was purified by column chromatography on silica gel (hexane:EtOAc = 10:1 → 1:1) to obtain the title compound **2an** (7.6 mg, 30%, 90% ee) as colorless oil along with the corresponding side-product **3an** (2.0 mg, 8%) as colorless oil and substrate **1a** (7.8 mg, 57%, 38% ee) as white solid.

The enantiomeric purity of the product was determined by HPLC analysis (Daicel CHIRALPAK AD3, hexane/*i*PrOH = 60:1, flow rate = 1.0 mL/min, retention time; 46.9 min (major) and 59.9 min (minor)).

The enantiomeric purity of the substrate was determined by HPLC analysis (Daicel CHIRALPAK ADH, hexane/EtOH = 20:1, flow rate = 1.0 mL/min, retention time; 27.0 min (minor) and 29.9 min (major)).

^{1}H NMR (400 MHz, $CDCl_3$) δ = 7.48 – 7.27 (m, 10 H), 4.97 (d, *J* = 9.0, 1 H), 4.56 – 4.41 (m, 2 H), 3.81 (dd, *J* = 9.7, 3.0, 1 H), 3.62 (t, *J* = 9.3, 1 H), 2.76 (s, 1 H) ppm. ^{13}C NMR (100 MHz, $CDCl_3$) δ = 132.0, 128.8, 128.7, 128.5, 128.1, 126.4, 86.9, 84.8, 77.5, 77.2, 76.8, 75.7, 73.0, 59.5 ppm. HRMS (ESI) exact mass calcd. for $C_{17}H_{16}NaO_2$: *m/z* 275.1043 ([M + Na]$^+$), found *m/z* 275.1045 ([M + Na]$^+$). IR (neat): 3421, 2916, 1703, 1490, 1071, 755 cm^{-1}. $[\alpha]_D^{27.4}$ = +6.0 (*c* = 0.76, $CHCl_3$).

(*S*)-2-(Benzyloxy)-1-(*o*-tolyl)ethanol (**2ba**)

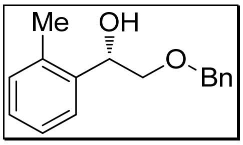

1-(*o*-Tolyl)ethane-1,2-diol (**1b**) (15.2 mg, 100 µmol), benzyl bromide (68 mg, 400 µmol), borinic acid **8** (1.1 mg, 5 µmol), PTC (*S,S*)-**7** (6.0 mg, 4 µmol), NaI (75 mg, 500 µmol) and Cs_2CO_3 (65 mg, 200 µmol) were reacted as described in **GP-1** for 120 h. The crude product mixture was purified by column chromatography on silica gel (hexane:EtOAc = 10:1 → 1:1) to obtain the title compound **2ba** (11.0 mg, 45%, 83% ee) as white solid along with the corresponding side-product **3ba** (5.6 mg, 23%) as colorless oil and substrate **1b** (4.4 mg, 29%, 75% ee) as white solid.

The enantiomeric purity of the product was determined by HPLC analysis (Daicel CHIRALPAK AD3, hexane/*i*PrOH = 60:1, flow rate = 1.0 mL/min, retention time; 27.5 min (major) and 34.5 min (minor)).

The enantiomeric purity of the substrate was determined by HPLC analysis (Daicel CHIRALPAK ADH, hexane/EtOH = 20:1, flow rate = 1.0 mL/min, retention time; 25.2 min (minor) and 26.7 min (major)).

^{1}H NMR (400 MHz, $CDCl_3$) δ = 7.51 (d, *J* = 7.4, 1 H), 7.40 – 7.27 (m, 5 H), 7.26 – 7.14 (m, 2 H), 7.12 (d, *J* = 7.3, 1 H), 5.16 (dd, *J* = 8.9, 1.9, 1H), 4.61 (q, *J* = 11.9, 2 H), 3.60 (dd, *J* = 10.0, 2.4, 1 H), 3.46 (t, *J* = 9.5, 1 H), 2.75 (s,

1 H), 2.30 (s, 3 H) ppm. ^{13}C NMR (100 MHz, $CDCl_3$) δ = 138.3, 138.1, 135.0, 130.5, 128.7, 128.1, 128.0, 127.8, 126.4, 126.1, 74.8, 73.5, 69.8, 19.1 ppm. HRMS (ESI) exact mass calcd. for $C_{16}H_{18}NaO_2$: *m/z* 265.1199 ([M + Na]$^+$), found *m/z* 265.1157 ([M + Na]$^+$). IR (neat): 3446, 2858, 1455, 1100, 1072, 754, 698 cm^{-1}. $[\alpha]_D^{23.1}$ = +34.0 (*c* = 1.00, $CHCl_3$).

(*S*)-2-(Benzyloxy)-1-(*m*-tolyl)ethanol (**2ca**)

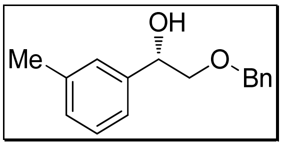

1-(*m*-Tolyl)ethane-1,2-diol (**1c**) (15.2 mg, 100 μmol), benzyl bromide (68 mg, 400 μmol), borinic acid **8** (1.1 mg, 5 μmol), PTC (*S,S*)-**7** (6.0 mg, 4 μmol), NaI (75 mg, 500 μmol) and Cs_2CO_3 (65 mg, 200 μmol) were reacted as described in **GP-1** for 120 h. The crude product mixture was purified by column chromatography on silica gel (hexane:EtOAc = 10:1 → 1:1) to obtain the title compound **2ca** (9.7 mg, 40%, 87% ee) as colorless oil along with the corresponding side-product **3ca** (1.8 mg, 7%) as colorless oil and substrate **1c** (5.8 mg, 38%, 60% ee) as white solid.

The enantiomeric purity of the product was determined by HPLC analysis (Daicel CHIRALPAK IE, hexane/*i*PrOH = 60:1, flow rate = 1.5 mL/min, retention time; 16.3 min (major) and 17.9 min (minor)).

The enantiomeric purity of the substrate was determined by HPLC analysis (Daicel CHIRALPAK ADH, hexane/EtOH = 20:1, flow rate = 1.0 mL/min, retention time; 25.2 min (minor) and 33.3 min (major)).

^{1}H NMR (400 MHz, $CDCl_3$) δ = 7.39 – 7.27 (m, 5 H), 7.24 (d, *J* = 8.7, 1 H), 7.19 (s, 1 H), 7.15 (d, *J* = 7.6, 1 H), 7.09 (d, *J* = 7.4, 1 H), 4.89 (dd, *J* = 9.0, 2.9, 1 H), 4.64 – 4.55 (m, 2 H), 3.63 (dd, *J* = 9.7, 3.2, 1 H), 3.50 (t, *J* = 9.4, 1H), 2.79 (s, 1 H), 2.34 (s, 3 H) ppm. ^{13}C NMR (100 MHz, $CDCl_3$) δ = 140.3, 138.3, 138.0, 128.8, 128.7, 128.5, 128.0, 128.0, 127.0, 123.5, 76.0, 73.6, 73.0, 21.5 ppm. HRMS (ESI) exact mass calcd. for $C_{16}H_{18}NaO_2$: *m/z* 265.1199 ([M + Na]$^+$), found *m/z* 265.1201 ([M + Na]$^+$). IR (neat): 3420, 2858, 1453, 1099, 1072, 735 cm^{-1}. $[\alpha]_D^{23.6}$ = +22.0 (*c* = 0.97, $CHCl_3$).

(*S*)-2-(Benzyloxy)-1-(*p*-tolyl)ethanol (**2da**)

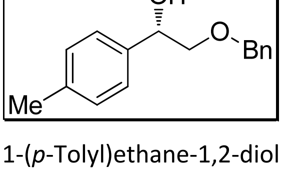

1-(*p*-Tolyl)ethane-1,2-diol (**1d**) (15.2 mg, 100 μmol), benzyl bromide (68 mg, 400 μmol), borinic acid **8** (1.1 mg, 5 μmol), PTC (*S,S*)-**7** (6.0 mg, 4 μmol), NaI (75 mg, 500 μmol) and Cs_2CO_3 (65 mg, 200 μmol) were reacted as described in **GP-1** for 120 h. The crude product mixture was purified by column chromatography on silica gel (hexane:EtOAc = 10:1 → 1:1) to obtain the title compound **2da** (8.2 mg, 34%, 97% ee) as colorless oil along with the corresponding side-product **3da** (7.7 mg, 32%) as colorless oil and substrate **1d** (5.2 mg, 34%, 74% ee) as white solid.

The enantiomeric purity of the product was determined by HPLC analysis (Daicel CHIRALPAK AD3, hexane/*i*PrOH = 60:1, flow rate = 0.5 mL/min, retention time; 60.0 min (major) and 64.1 min (minor)).
The enantiomeric purity of the substrate was determined by HPLC analysis (Daicel CHIRALPAK ADH, hexane/EtOH = 20:1, flow rate = 1.0 mL/min, retention time; 27.2 min (minor) and 28.6 min (major)).

^{1}H NMR (400 MHz, $CDCl_3$) δ = 7.39 – 7.28 (m, 5 H), 7.26 (d, *J* = 7.8, 2 H), 7.15 (d, *J* = 7.9, 2 H), 4.93 – 4.83 (m, 1 H), 4.65 – 4.52 (m, 2 H), 3.62 (dd, *J* = 9.7, 3.2, 1 H), 3.50 (t, *J* = 9.4, 1 H), 2.77 (d, *J* = 2.2, 1 H), 2.33 (s, 3 H) ppm. ^{13}C NMR (100 MHz, $CDCl_3$) δ = 138.1, 137.7, 137.4, 129.3, 128.7, 128.0, 126.3, 76.0, 73.6, 72.9, 21.2 ppm. HRMS (ESI) exact mass calcd. for $C_{16}H_{18}NaO_2$: *m/z* 265.1199 ([M + Na]$^+$), found *m/z* 265.1202 ([M + Na]$^+$). IR (neat): 3420, 2859, 1453, 1198, 1102, 1070, 735 cm^{-1}. $[\alpha]_D^{24.2}$ = +30.8 (*c* = 0.82, $CHCl_3$).

(*S*)-2-(Benzyloxy)-1-(2-fluorophenyl)ethanol (**2ea**)

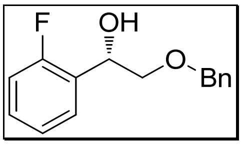

1-(2-Fluorophenyl)ethane-1,2-diol (**1e**) (15.6 mg, 100 µmol), benzyl bromide (68 mg, 400 µmol), borinic acid **8** (1.1 mg, 5 µmol), PTC (*S,S*)-**7** (6.0 mg, 4 µmol), NaI (75 mg, 500 µmol) and Cs_2CO_3 (65 mg, 200 µmol) were reacted as described in **GP-1** for 120 h. The crude product mixture was purified by column chromatography on silica gel (hexane:EtOAc = 8:1 → 1:1) to obtain the title compound **2ea** (10.4 mg, 42%, 87% ee) as white solid along with the corresponding side-product **3ea** (3.2 mg, 13%) as colorless oil and substrate **1e** (6.0 mg, 38%, 96% ee) as white solid.
The enantiomeric purity of the product was determined by HPLC analysis (Daicel CHIRALPAK AD3, hexane/*i*PrOH = 60:1, flow rate = 1.0 mL/min, retention time; 27.9 min (major) and 30.8 min (minor)).
The enantiomeric purity of the substrate was determined by HPLC analysis (Daicel CHIRALPAK OD3, hexane/EtOH = 20:1, flow rate = 1.0 mL/min, retention time; 10.0 min (major) and 12.0 min (minor)).

^{1}H NMR (500 MHz, $CDCl_3$) δ = 7.58 – 7.51 (m, 1 H), 7.38 – 7.23 (m, 6 H), 7.15 (td, *J* = 7.5, 1.1, 1 H), 7.01 (ddd, *J* = 10.5, 8.2, 1.1, 1 H), 5.26 (dt, *J* = 8.5, 2.8, 1 H), 4.66 – 4.55 (m, 2 H), 3.73 (ddd, *J* = 9.8, 3.1, 1.0, 1 H), 3.55 – 3.46 (m, 1 H) ppm. ^{13}C NMR (125 MHz, $CDCl_3$) δ = 159.9 (d, *J* = 245.9), 137.8, 129.2 (d, *J* = 8.3), 128.6, 128.0, 127.9, 127.8 (d, *J* = 4.2), 127.3 (d, *J* = 13.2), 124.4 (d, *J* = 3.5), 115.2 (d, *J* = 21.7), 74.4 (d, *J* = 1.2), 73.4, 67.0 (d, *J* = 2.8) ppm. ^{19}F NMR (466 MHz, $CDCl_3$) δ = -119.2 (ddd, *J* = 10.9, 6.9, 5.9) ppm. HRMS (ESI) exact mass calcd. for $C_{15}H_{15}FNaO_2$: *m/z* 269.0937 ([M + Na]$^+$), found *m/z* 269.0950 ([M + Na]$^+$). IR (neat): 3446, 2861, 1489, 1101, 1073, 757 cm^{-1}. $[\alpha]_D^{24.6}$ = +22.0 (*c* = 1.04, $CHCl_3$).

(*S*)-2-(Benzyloxy)-1-(3-fluorophenyl)ethanol (**2fa**)

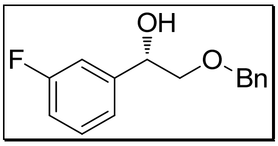

1-(3-Fluorophenyl)ethane-1,2-diol (**1f**) (15.6 mg, 100 µmol), benzyl bromide (68 mg, 400 µmol), borinic acid **8** (1.1 mg, 5 µmol), PTC (*S,S*)-**7** (6.0 mg, 4 µmol), NaI (75 mg, 500 µmol) and Cs_2CO_3 (65 mg, 200 µmol) were reacted as described in **GP-1** for 120 h. The crude product mixture was purified by column chromatography on silica gel (hexane:EtOAc = 8:1 → 1:1) to obtain the title compound **2fa** (9.9 mg, 40%, 87% ee) as colorless oil along with the corresponding side-product **3fa** (2.8 mg, 11%) as colorless oil and substrate **1f** (5.6 mg, 36%, 74% ee) as white solid.

The enantiomeric purity of the product was determined by HPLC analysis (Daicel CHIRALPAK AD3, hexane/*i*PrOH = 60:1, flow rate = 1.0 mL/min, retention time; 31.0 min (major) and 33.1 min (minor)).

The enantiomeric purity of the substrate was determined by HPLC analysis (Daicel CHIRALPAK OD3, hexane/EtOH = 20:1, flow rate = 1.0 mL/min, retention time; 11.7 min (major) and 13.6 min (minor)).

^{1}H NMR (500 MHz, $CDCl_3$) δ = 7.39 – 7.27 (m, 6 H), 7.16 – 7.08 (m, 2 H), 6.97 (tdd, *J* = 8.5, 2.6, 0.9, 1 H), 4.91 (dd, *J* = 8.7, 2.9, 1 H), 4.66 – 4.47 (m, 2 H), 3.64 (dd, *J* = 9.7, 3.3, 1 H), 3.47 (dd, *J* = 9.7, 8.8, 1 H), 2.85 (s, 1 H) ppm. ^{13}C NMR (125 MHz, $CDCl_3$) δ = 163.0 (d, *J* = 245.8), 142.9 (d, *J* = 7.2), 137.7, 129.9 (d, *J* = 8.2), 128.6, 128.1, 127.9, 121.8 (d, *J* = 2.9), 114.7 (d, *J* = 21.3), 113.3 (d, *J* = 22.1), 75.6, 73.6 (s, *J* = 1.7), 72.3 (d, *J* = 1.8) ppm. ^{19}F NMR (466 MHz, $CDCl_3$) δ = -112.9 (ddd, *J* = 10.1, 8.7, 5.8) ppm. HRMS (ESI) exact mass calcd. for $C_{15}H_{15}FNaO_2$: *m/z* 269.0948 ([M + Na]$^+$), found *m/z* 269.0948 ([M + Na]$^+$). IR (neat): 3429, 2858, 1592, 1451, 1246, 1105, 696 cm^{-1}. $[\alpha]_D^{24.9}$ = +16.8 (*c* = 0.99, $CHCl_3$).

(*S*)-2-(Benzyloxy)-1-(4-fluorophenyl)ethanol (**2ga**)

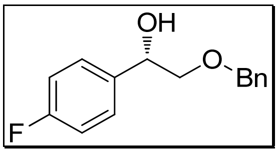

1-(4-Fluorophenyl)ethane-1,2-diol (**1g**) (15.6 mg, 100 µmol), benzyl bromide (68 mg, 400 µmol), borinic acid **8** (1.1 mg, 5 µmol), PTC (*S,S*)-**7** (6.0 mg, 4 µmol), NaI (75 mg, 500 µmol) and Cs_2CO_3 (65 mg, 200 µmol) were reacted as described in **GP-1** for 120 h. The crude product mixture was purified by column chromatography on silica gel (hexane:EtOAc = 8:1 → 1:1) to obtain the title compound **2ga** (10.8 mg, 44%, 91% ee) as colorless oil along with the corresponding side-product **3ga** (1.5 mg, 6%) as colorless oil and substrate **1g** (7.3 mg, 47%, 70% ee) as white solid.

The enantiomeric purity of the product was determined by HPLC analysis (Daicel CHIRALPAK AD3, hexane/*i*PrOH = 60:1, flow rate = 0.5 mL/min, retention time; 64.6 min (major) and 67.1 min (minor)).

The enantiomeric purity of the substrate was determined by HPLC analysis (Daicel CHIRALPAK OD3, hexane/EtOH = 20:1, flow rate = 1.0 mL/min, retention time; 12.3 min (major) and 14.0 min (minor)).

^{1}H NMR (500 MHz, $CDCl_3$) δ = 7.39 – 7.28 (m, 7 H), 7.06 – 6.99 (m, 2 H), 4.90 (dd, *J* = 8.8, 2.8, 1 H), 4.64 – 4.54 (m, 2 H), 3.61 (dd, *J* = 9.7, 3.3, 1 H), 3.47 (dd, *J* = 9.7, 8.9, 1 H), 2.85 (s, 1 H) ppm. ^{13}C NMR (125 MHz, $CDCl_3$) δ = 162.5 (d, *J* = 245.8), 137.7, 136.0 (d, *J* = 3.1), 128.6, 128.0 (d, *J* = 5.8), 127.9, 115.3 (d, *J* = 21.4), 75.8 (d, *J* = 1.4), 73.5, 72.3 ppm. ^{19}F NMR (466 MHz, $CDCl_3$) δ = -114.6 (tt, *J* = 8.7, 5.4) ppm. HRMS (ESI) exact mass calcd. for $C_{15}H_{15}FNaO_2$: *m/z* 269.0948 ([M + Na]$^+$), found *m/z* 269.0950 ([M + Na]$^+$). IR (neat): 3421, 2860, 1604, 1509, 1220, 1099, 836 cm^{-1}. $[\alpha]_{\mathrm{D}}^{25.3}$ = +17.1 (*c* = 1.08, $CHCl_3$).

(*S*)-2-(Benzyloxy)-1-(4-bromophenyl)ethanol (**2ha**)

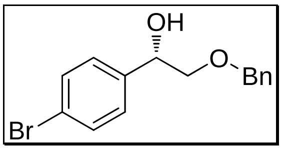

1-(4-Bromophenyl)ethane-1,2-diol (**1h**) (21.7 mg, 100 µmol), benzyl bromide (68 mg, 400 µmol), borinic acid **8** (1.1 mg, 5 µmol), PTC (*S,S*)-**7** (6.0 mg, 4 µmol), NaI (75 mg, 500 µmol) and Cs_2CO_3 (65 mg, 200 µmol) were reacted as described in **GP-1** for 120 h. The crude product mixture was purified by column chromatography on silica gel (hexane:EtOAc = 3:1 → 1:1) to obtain the title compound **2ha** (10.1 mg, 33%, 82% ee) as white solid along with the corresponding side-product **3ha** (4.3 mg, 14%) as white solid and substrate **2ha** (11.1 mg, 51%, 33% ee) as white solid.

The enantiomeric purity of the product was determined by HPLC analysis (Daicel CHIRALPAK AD3, hexane/*i*PrOH = 60:1, flow rate = 1.0 mL/min, retention time; 35.6 min (major) and 38.7 min (minor)).

The enantiomeric purity of the substrate was determined by HPLC analysis (Daicel CHIRALPAK OD3, hexane/EtOH = 20:1, flow rate = 1.0 mL/min, retention time; 14.0 min (major) and 15.9 min (minor)).

^{1}H NMR (400 MHz, $CDCl_3$) δ = 7.47 (d, *J* = 8.3, 2 H), 7.40 – 7.29 (m, 5 H), 7.25 (d, *J* = 8.2, 2 H), 4.87 (s, 1 H), 4.59 (s, 2 H), 3.62 (dd, *J* = 9.7, 3.2, 1 H), 3.45 (t, *J* = 9.3, 1 H), 2.82 (d, *J* = 2.3, 1 H) ppm. ^{13}C NMR (100 MHz, $CDCl_3$) δ = 139.4, 137.7, 131.6, 128.7, 128.1, 128.0, 121.8, 75.5, 73.6, 72.3 ppm. HRMS (ESI) exact mass calcd. for $C_{15}H_{15}BrNaO_2$: *m/z* 329.0148 ([M + Na]$^+$), found *m/z* 329.0103 ([M + Na]$^+$). IR (neat): 3419, 2859, 1488, 1103, 1028, 821, 698 cm^{-1}. $[\alpha]_{\mathrm{D}}^{26.3}$ = +11.7 (*c* = 1.01, $CHCl_3$).

(*S*)-2-(Benzyloxy)-1-(4-methoxyphenyl)ethanol (**2ia**)

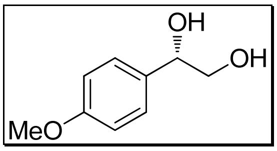

1-(4-Methoxyphenyl)ethane-1,2-diol (**1i**) (16.8 mg, 100 µmol), benzyl bromide (68 mg, 400 µmol), borinic acid **8** (1.1 mg, 5 µmol), PTC (*S,S*)-**7** (6.0 mg, 4 µmol), NaI (75 mg, 500 µmol) and Cs_2CO_3 (65 mg, 200 µmol) were reacted as described in **GP-1** for 110 h. The crude product mixture was purified by column chromatography on silica gel (hexane:EtOAc = 4:1 → 1:1) to obtain the title compound **2ia** (11.4 mg, 44%, 88% ee) as white solid along with the corresponding side-product **3ia** (3.4 mg, 13%) as colorless oil and substrate **1i** (5.8 mg, 35%, 86% ee) as white solid.

The enantiomeric purity of the product was determined by HPLC analysis (Daicel CHIRALPAK AD3, hexane/*i*PrOH = 60:1, flow rate = 1.0 mL/min, retention time; 52.8 min (major) and 57.5 min (minor)).
The enantiomeric purity of the substrate was determined by HPLC analysis (Daicel CHIRALPAK ADH, hexane/EtOH = 20:1, flow rate = 1.0 mL/min, retention time; 47.1 min (minor) and 50.4 min (major)).

^{1}H NMR (400 MHz, $CDCl_3$) δ = 7.39 – 7.24 (m, 7 H), 6.87 (d, *J* = 8.5, 2 H), 4.87 (d, *J* = 8.9, 1 H), 4.65 – 4.54 (m, 2 H), 3.79 (s, 3 H), 3.60 (dd, *J* = 9.7, 3.2, 1 H), 3.50 (t, *J* = 9.3, 1 H), 2.76 (d, *J* = 1.7, 1 H) ppm. ^{13}C NMR (100 MHz, $CDCl_3$) δ 159.6, 138.1, 132.6, 128.7, 128.0, 128.0, 127.6, 114.0, 76.0, 73.5, 72.6, 55.4 ppm. HRMS (ESI) exact mass calcd. for $C_{16}H_{18}NaO_3$: *m/z* 281.1148 ([M + Na]$^+$), found *m/z* 281.1152 ([M + Na]$^+$). IR (neat): 3421, 2855, 1512, 1245, 1029, 830, 697 cm^{-1}. $[\alpha]_D^{25.6}$ = +22.3 (*c* = 1.14, $CHCl_3$).

(*S*)-2-(Benzyloxy)-1-cyclohexylethanol (**2oa**)

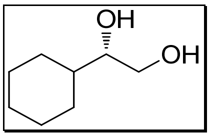

3,3-Dimethylbutane-1,2-diol (**1o**) (14.4 mg, 100 µmol), benzyl bromide (68 mg, 400 µmol), borinic acid **8** (2.2 mg, 10 µmol), PTC (*S,S*)-**7** (6.0 mg, 4 µmol), NaI (75 mg, 500 µmol) and Cs_2CO_3 (65 mg, 200 µmol) were reacted as described in **GP-1** for 120 h. The crude product mixture was purified by column chromatography on silica gel (hexane:EtOAc = 10:1 → 1:1) to obtain the title compound **2oa** (9.0 mg, 38%, 48 ee) as colorless oil along with substrate **1o** (5.8 mg, 40%, 17% ee) as white solid.
The enantiomeric purity of the product was determined by HPLC analysis (Daicel CHIRALPAK AD3, hexane/*i*PrOH = 60:1, flow rate = 1.0 mL/min, retention time; 16.9 min (major) and 20.9 min (minor)).
The enantiomeric purity of the substrate was determined by HPLC analysis using its benzylated version (Daicel CHIRALPAK AD3, hexane/*i*PrOH = 60:1, flow rate = 1.0 mL/min, retention time; 10.3 min (major) and 12.1 min (minor)).

^{1}H NMR (400 MHz, $CDCl_3$) δ = 7.41 – 7.27 (m, 5 H), 4.55 (s, 2 H), 3.57 (dd, *J* = 9.8, 2.7, 2 H), 3.41 (t, *J* = 9.3, 1 H), 2.32 (s, 1 H), 1.87 (d, *J* = 12.7, 1 H), 1.80 – 1.55 (m, 4 H), 1.51 – 1.35 (m, 1 H), 1.32 – 0.96 (m, 5 H) ppm. ^{13}C NMR (100 MHz, $CDCl_3$) δ = 138.3, 128.6, 127.9, 127.9, 74.6, 73.5, 72.9, 40.9, 29.1, 28.7, 26.6, 26.3, 26.2 ppm. HRMS (ESI) exact mass calcd. for $C_{15}H_{22}NaO_2$: *m/z* 257.1512 ([M + Na]$^+$), found *m/z* 257.1514 ([M + Na]$^+$). IR (neat): 3447, 2921, 1450, 1096, 735 cm^{-1}. $[\alpha]_D^{26.8}$ = +2.8 (*c* = 0.98, $CHCl_3$).

(*S*)-1-(Benzyloxy)-2-phenylpropan-2-ol (**13a**)

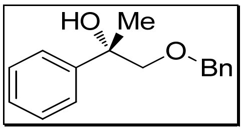

2-Phenylpropane-1,2-diol (**12a**) (15.2 mg, 100 µmol), benzyl bromide (68 mg, 400 µmol), borinic acid **8** (2.1 mg, 10 µmol), PTC (*S,S*)-**7** (6.0 mg, 4 µmol), NaI (75 mg, 500 µmol) and Cs_2CO_3 (65 mg, 200 µmol) were reacted as

described in **GP-2** for 120 h. The crude product mixture was purified by column chromatography on silica gel (hexane:EtOAc = 6:1 → 1:1) to obtain the title compound **13a** (11.4 mg, 47%, 84% ee) as colorless oil and substrate **12a** (6.8 mg, 46%, 81% ee) as white solid.

The enantiomeric purity of the product was determined by HPLC analysis (Daicel CHIRALPAK AD3, hexane/*i*PrOH = 60:1, flow rate = 1.0 mL/min, retention time; 15.1 min (major) and 17.1 min (minor)).

The enantiomeric purity of the substrate was determined by HPLC analysis (Daicel CHIRALPAK ADH, hexane/EtOH = 20:1, flow rate = 1.0 mL/min, retention time; 27.5 min (minor) and 41.9 min (major)).

^{1}H NMR (400 MHz, $CDCl_3$) δ = 7.48 – 7.42 (m, 2 H), 7.36 – 7.21 (m, 8 H), 4.53 (s, 2 H), 3.62 (d, *J* = 9.2, 1 H), 3.55 (d, *J* = 9.2, 1 H), 2.92 (s, 1 H), 1.52 (s, 3 H) ppm. ^{13}C NMR (100 MHz, $CDCl_3$) δ = 145.7, 138.1, 128.6, 128.3, 127.9, 127.8, 127.1, 125.2, 78.3, 74.1, 73.6, 26.8 ppm. HRMS (ESI) exact mass calcd. for $C_{16}H_{18}NaO_2$: *m/z* 265.1199 ([M + Na]$^+$), found *m/z* 265.1201 ([M + Na]$^+$). IR (neat): 3452, 2860, 1447, 1093, 736, 696 cm^{-1}. $[\alpha]_D^{27.5}$ = –12.0 (*c* = 1.16, $CHCl_3$).

(*S*)-1-(Benzyloxy)-2-(*o*-tolyl)propan-2-ol (**13b**)

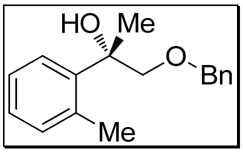

2-(*o*-Tolyl)propane-1,2-diol (**12b**) (16.6 mg, 100 µmol), benzyl bromide (68 mg, 400 µmol), borinic acid **8** (2.1 mg, 10 µmol), PTC (*S,S*)-**7** (6.0 mg, 4 µmol), NaI (75 mg, 500 µmol) and Cs_2CO_3 (65 mg, 200 µmol) were reacted as described in **GP-2** for 120 h. The crude product mixture was purified by column chromatography on silica gel (hexane:EtOAc = 6:1 → 1:1) to obtain the title compound **13b** (10.8 mg, 42%, 82% ee) as colorless oil and substrate **12b** (7.0 mg, 42%, 67% ee) as white solid.

The enantiomeric purity of the product was determined by HPLC analysis (Daicel CHIRALPAK AD3, hexane/*i*PrOH = 60:1, flow rate = 1.0 mL/min, retention time; 11.1 min (major) and 14.3 min (minor)).

The enantiomeric purity of the substrate was determined by HPLC analysis (Daicel CHIRALPAK ADH, hexane/*i*PrOH = 20:1, flow rate = 1.0 mL/min, retention time; 15.6 min (minor) and 17.7 min (major)).

^{1}H NMR (400 MHz, $CDCl_3$) δ = 7.38 (dt, *J* = 4.4, 2.7, 1 H), 7.36 – 7.25 (m, 5 H), 7.16 – 7.10 (m, 3 H), 4.58 (s, 2 H), 3.86 (d, *J* = 9.3, 1 H), 3.58 (d, *J* = 9.3, 1 H), 2.93 (s, 1 H), 2.51 (s, 3 H), 1.58 (d, *J* = 6.0, 3 H) ppm. ^{13}C NMR (100 MHz, $CDCl_3$) δ = 142.5, 138.1, 136.3, 132.8, 128.6, 128.0, 127.9, 127.3, 126.5, 125.8, 76.8, 75.2, 73.6, 26.1, 22.3 ppm. HRMS (ESI) exact mass calcd. for $C_{17}H_{20}NaO_2$: *m/z* 279.1356 ([M + Na]$^+$), found *m/z* 279.1359 ([M + Na]$^+$). IR (neat): 3552, 2861, 1453, 1091, 726 cm^{-1}. $[\alpha]_D^{27.5}$ = –11.3 (*c* = 1.08, $CHCl_3$).

(*S*)-1-(Benzyloxy)-2-(*m*-tolyl)propan-2-ol (**13c**)

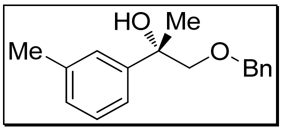

2-(*m*-Tolyl)propane-1,2-diol (**12c**) (16.6 mg, 100 µmol), benzyl bromide (68 mg, 400 µmol), borinic acid **8** (2.1 mg, 10 µmol), PTC (*S,S*)-**7** (6.0 mg, 4 µmol), NaI (75 mg, 500 µmol) and Cs_2CO_3 (65 mg, 200 µmol) were reacted as described in **GP-2** for 120 h. The crude product mixture was purified by column chromatography on silica gel (hexane:EtOAc = 6:1 → 1:1) to obtain the title compound **13c** (89.8 mg, 38%, 85% ee) as colorless oil and substrate **12c** (9.0 mg, 54%, 48% ee) as white solid.

The enantiomeric purity of the product was determined by HPLC analysis (Daicel CHIRALPAK AD3, hexane/*i*PrOH = 60:1, flow rate = 1.0 mL/min, retention time; 13.6 min (major) and 15.1 min (minor)).

The enantiomeric purity of the substrate was determined by HPLC analysis (Daicel CHIRALPAK ADH, hexane/*i*PrOH = 20:1, flow rate = 1.0 mL/min, retention time; 16.8 min (minor) and 24.4 min (major)).

^{1}H NMR (400 MHz, $CDCl_3$) δ = 7.36 – 7.20 (m, 8 H), 7.06 (t, *J* = 3.5, 1 H), 4.54 (s, 2 H), 3.61 (d, *J* = 9.2, 1 H), 3.54 (d, *J* = 9.2, 1 H), 2.88 (s, 1 H), 2.35 (s, 3 H), 1.51 (s, 3 H) ppm. ^{13}C NMR (100 MHz, $CDCl_3$) δ = 145.6, 138.2, 137.9, 128.6, 128.2, 127.9, 127.8, 125.9, 122.2, 78.3, 74.0, 73.6, 26.9, 21.7 ppm. HRMS (ESI) exact mass calcd. for $C_{17}H_{20}NaO_2$: *m/z* 279.1356 ([M + Na]$^+$), found *m/z* 279.1357 ([M + Na]$^+$). IR (neat): 3453, 2860, 1453, 1091, 697 cm^{-1}. $[\alpha]_D^{27.5}$ = +30.7 (*c* = 0.98, $CHCl_3$).

(*S*)-1-(Benzyloxy)-2-(*p*-tolyl)propan-2-ol (**13d**)

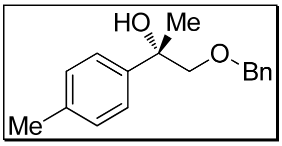

2-(*p*-Tolyl)propane-1,2-diol (**12d**) (16.6 mg, 100 µmol), benzyl bromide (68 mg, 400 µmol), borinic acid **8** (3.2 mg, 15 µmol), PTC (*S,S*)-**7** (6.0 mg, 4 µmol), NaI (75 mg, 500 µmol) and Cs_2CO_3 (65 mg, 200 µmol) were reacted as described in **GP-2** for 120 h. The crude product mixture was purified by column chromatography on silica gel (hexane:EtOAc = 6:1 → 1:1) to obtain the title compound **13d** (10.5 mg, 41%, 83% ee) as colorless oil and substrate **12d** (7.6 mg, 46 µmol, 60% ee) as white solid.

The enantiomeric purity of the product was determined by HPLC analysis (Daicel CHIRALPAK AD3, hexane/*i*PrOH = 60:1, flow rate = 1.0 mL/min, retention time; 15.4 min (major) and 17.3 min (minor)).

The enantiomeric purity of the substrate was determined by HPLC analysis (Daicel CHIRALPAK ADH, hexane/*i*PrOH = 20:1, flow rate = 1.0 mL/min, retention time; 18.0 min (minor) and 21.9 min (major)).

^{1}H NMR (400 MHz, $CDCl_3$) δ = 7.38 – 7.24 (m, 7 H), 7.14 (d, *J* = 8.0, 2 H), 4.54 (s, 2 H), 3.60 (d, *J* = 9.2, 1 H), 3.53 (d, *J* = 9.2, 1 H), 2.86 (s, 1 H), 2.33 (s, 3 H), 1.51 (s, 3 H) ppm. ^{13}C NMR (100 MHz, $CDCl_3$) δ = 142.7, 138.2, 136.6, 129.0, 128.6, 127.9, 127.8, 125.1, 78.3, 74.0, 73.6, 26.9, 21.1 ppm. HRMS (ESI) exact mass calcd. for $C_{17}H_{20}NaO_2$:

m/z 279.1356 ([M + Na]$^+$), found m/z 279.1358 ([M + Na]$^+$). IR (neat): 3447, 2860, 1454, 1095, 817 cm^{-1}. $[\alpha]_D^{27.6}$ = –14.4 (c = 0.76, $CHCl_3$).

(*S*)-1-(benzyloxy)-2-(4-fluorophenyl)propan-2-ol (**13e**)

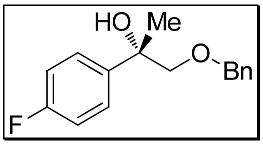

2-(4-Fluorophenyl)propane-1,2-diol (**12e**) (18.2 mg, 100 µmol), benzyl bromide (68 mg, 400 µmol), borinic acid **8** (2.1 mg, 10 µmol), PTC (*S,S*)-**7** (6.0 mg, 4 µmol), NaI (75 mg, 500 µmol) and Cs_2CO_3 (65 mg, 200 µmol) were reacted as described in **GP-2** for 120 h. The crude product mixture was purified by column chromatography on silica gel (hexane:EtOAc = 4:1 → 1:1) to obtain the title compound **13e** (7.8 mg, 30%, 91% ee) as colorless oil and substrate **12e** (6.8 mg, 38%, 43% ee) as white solid.

The enantiomeric purity of the product was determined by HPLC analysis (Daicel CHIRALPAK AD3, hexane/*i*PrOH = 60:1, flow rate = 0.5 mL/min, retention time; 31.7 min (major) and 33.0 min (minor)).

The enantiomeric purity of the substrate was determined by HPLC analysis (Daicel CHIRALPAK AD3, hexane/*i*PrOH = 20:1, flow rate = 0.1 mL/min, retention time; 37.4 min (minor) and 41.2 min (major)).

^{1}H NMR (400 MHz, $CDCl_3$) δ = 7.41 (dd, *J* = 8.6, 5.4, 2 H), 7.37 – 7.22 (m, 5 H), 7.01 (t, *J* = 8.7, 2 H), 4.55 (s, 2 H), 3.59 (d, *J* = 9.2, 1 H), 3.53 (d, *J* = 9.2, 1 H), 2.89 (s, 1 H), 1.51 (s, 3 H) ppm. ^{13}C NMR (125 MHz, $CDCl_3$) δ = 161.9 (d, *J* = 244.9), 141.3 (d, *J* = 3.0), 137.9, 128.5, 127.9, 127.7, 126.9 (d, *J* = 8.6), 114.9 (d, *J* = 21.2), 78.1, 73.8, 73.5, 26.9 ppm. ^{19}F NMR (466 MHz, $CDCl_3$) δ = -116.4 (tt, *J* = 8.5, 5.4) ppm. HRMS (ESI) exact mass calcd. for $C_{16}H_{17}FNaO_2$: m/z 283.1105 ([M + Na]$^+$), found m/z 283.1106 ([M + Na]$^+$). IR (neat): 3442, 2859, 1602, 1509, 1095, 836 cm^{-1}. $[\alpha]_D^{27.7}$ = –10.3 (c = 0.78, $CHCl_3$).

(*S*)-1-(benzyloxy)-2-(4-methoxyphenyl)propan-2-ol (**13f**)

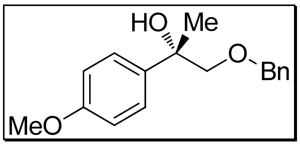

2-(4-Methoxyphenyl)propane-1,2-diol (**12f**) (18.2 mg, 100 µmol), benzyl bromide (68 mg, 400 µmol), borinic acid **8** (2.1 mg, 10 µmol), PTC (*S,S*)-**7** (6.0 mg, 4 µmol), NaI (75 mg, 500 µmol) and Cs_2CO_3 (65 mg, 200 µmol) were reacted as described in **GP-2** for 120 h. The crude product mixture was purified by column chromatography on silica gel (hexane:EtOAc = 4:1 → 1:1) to obtain the title compound **13f** (12.4 mg, 46%, 87% ee) as colorless oil and substrate **12f** (8.3 mg, 46, 80% ee) as white solid.

The enantiomeric purity of the product was determined by HPLC analysis (Daicel CHIRALPAK AD3, hexane/*i*PrOH = 60:1, flow rate = 1.0 mL/min, retention time; 28.7 min (major) and 30.4 min (minor)).

The enantiomeric purity of the substrate was determined by HPLC analysis (Daicel CHIRALPAK ADH, hexane/EtOH = 15:1, flow rate = 1.0 mL/min, retention time; 20.0 min (minor) and 24.7 min (major)).

^{1}H NMR (400 MHz, $CDCl_3$) δ = 7.40 – 7.24 (m, 7 H), 6.90 – 6.84 (m, 2 H), 4.54 (s, 2 H), 3.79 (s, 3 H), 3.59 (d, *J* = 9.2, 1 H), 3.51 (d, *J* = 9.2, 1 H), 2.85 (s, 1 H), 1.51 (s, 3 H) ppm. ^{13}C NMR (100 MHz, $CDCl_3$) δ = 158.8, 138.2, 137.8, 128.6, 127.9, 127.8, 126.4, 113.7, 78.4, 73.8, 73.6, 55.4, 26.8 ppm. HRMS (ESI) exact mass calcd. for $C_{17}H_{20}NaO_3$: *m/z* 295.1305 ([M + Na]$^+$), found *m/z* 295.1307 ([M + Na]$^+$). IR (neat): 3462, 2859, 1611, 1511, 1246, 1093, 735 cm^{-1}. $[\alpha]_D^{27.7}$ = –11.1 (*c* = 1.24, $CHCl_3$).

(*S*)-1-(Benzyloxy)-2-(naphthalen-2-yl)propan-2-ol (**13g**)

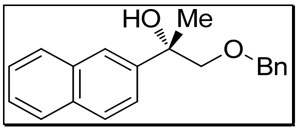

2-(4-Fluorophenyl)propane-1,2-diol (**12g**) (20.2 mg, 100 µmol), benzyl bromide (68 mg, 400 µmol), borinic acid **8** (2.1 mg, 10 µmol), PTC (*S,S*)-**7** (6.0 mg, 4 µmol), NaI (75 mg, 500 µmol) and Cs_2CO_3 (65 mg, 200 µmol) were reacted as described in **GP-2** for 120 h. The crude product mixture was purified by column chromatography on silica gel (hexane:EtOAc = 8:1 → 1:1) to obtain the title compound **13g** (9.7 mg, 33%, 78% ee) as orange solid and substrate **12g** (13.2 mg, 65%, 26% ee) as white solid.
The enantiomeric purity of the product was determined by HPLC analysis (Daicel CHIRALPAK AD3, hexane/*i*PrOH = 60:1, flow rate = 1.0 mL/min, retention time; 32.6 min (major) and 43.8 min (minor)).
The enantiomeric purity of the substrate was determined by HPLC analysis (Daicel CHIRALPAK OD3, hexane/EtOH = 15:1, flow rate = 1.0 mL/min, retention time; 15.8 min (major) and 24.4 min (minor)).

^{1}H NMR (400 MHz, $CDCl_3$) δ = 7.94 (s, 1 H), 7.82 (dd, *J* = 9.1, 6.0, 3 H), 7.56 – 7.51 (m, 1 H), 7.46 (p, *J* = 6.8, 2 H), 7.28 (dt, *J* = 13.9, 6.7, 5 H), 4.55 (s, 2 H), 3.74 (d, *J* = 9.2, 1 H), 3.64 (d, *J* = 9.2, 1 H), 3.03 (s, 1 H), 1.60 (s, 3 H) ppm. ^{13}C NMR (100 MHz, $CDC3_3$) δ = 143.1, 138.1, 133.4, 132.7, 128.6, 128.4, 128.0, 128.0, 127.9, 127.7, 126.2, 125.9, 124.0, 123.7, 78.1, 74.3, 73.7, 26.9 ppm. HRMS (ESI) exact mass calcd. for $C_{20}H_{20}NaO_2$: *m/z* 315.1356 ([M + Na]$^+$), found *m/z* 315.1363 ([M + Na]$^+$). IR (neat): 3446, 2859, 2360, 1340, 1094, 747 cm^{-1}. $[\alpha]_D^{32.8}$ = –19.8 (*c* = 0.97, $CHCl_3$).

(*S*)-1-(Benzyloxy)-2-(thiophen-3-yl)propan-2-ol (**13h**)

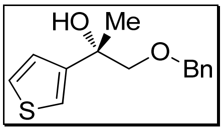

(*S*)-2-(Thiophen-3-yl)propane-1,2-diol (**12h**) (15.8 mg, 100 µmol), benzyl bromide (68 mg, 400 µmol), borinic acid **8** (2.1 mg, 10 µmol), PTC (*S,S*)-**7** (6.0 mg, 4 µmol), NaI (75 mg, 500 µmol) and Cs_2CO_3 (65 mg, 200 µmol) were reacted as described in **GP-2** for 80 h. The crude product mixture was purified by column chromatography on silica gel (hexane:EtOAc = 10:1 → 1:1) to obtain the title compound **13h** (10.9 mg, 44%, 72% ee) as pale organe oil and substrate **12h** (8.6 mg, 54%, 37% ee) as pale orange oil.
The enantiomeric purity of the product was determined by HPLC analysis (Daicel CHIRALPAK IC3, hexane/*i*PrOH = 60:1, flow rate = 1.0 mL/min, retention time; 15.8 min (minor) and 17.9 min (major)).

The enantiomeric purity of the substrate was determined by HPLC analysis (Daicel CHIRALPAK OD3, hexane/*i*PrOH = 20:1, flow rate = 0.5 mL/min, retention time; 37.8 min (minor) and 41.1 min (major)).

^{1}H NMR (400 MHz, $CDCl_3$) δ = 7.38 – 7.25 (m, 5 H), 7.20 (dd, *J* = 4.6, 1.5, 1 H), 6.98 – 6.92 (m, 2 H), 4.58 (d, *J* = 1.7, 2 H), 3.65 (d, *J* = 9.2, 1 H), 3.56 (d, *J* = 9.2, 1 H), 3.07 (s, 1 H), 1.62 (s, *J* = 7.1, 3 H) ppm. ^{13}C NMR (100 MHz, $CDCl_3$) δ = 150.5, 138.0, 128.6, 128.0, 127.9, 126.8, 124.3, 123.0, 78.7, 73.7, 73.3, 27.5 ppm. HRMS (ESI) exact mass calcd. for $C_{14}H_{16}NaO_2S$: *m/z* 271.0763 ([M + Na]$^+$), found *m/z* 271.0766 ([M + Na]$^+$). IR (neat): 3447, 2859, 1453, 1093, 695 cm^{-1}. $[\alpha]_D^{27.8}$ = –6.5 (*c* = 1.06, $CHCl_3$).

(*S*)-1-(Benzyloxy)-2-phenylpent-4-en-2-ol (**13i**)

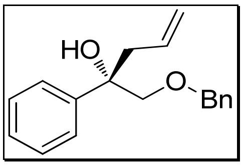

(*S*)-2-Phenylpent-4-ene-1,2-diol (**12i**) (17.8 mg, 100 µmol), benzyl bromide (68 mg, 400 µmol), borinic acid **8** (2.1 mg, 10 µmol), PTC (*S,S*)-**7** (6.0 mg, 4 µmol), NaI (75 mg, 500 µmol) and Cs_2CO_3 (65 mg, 200 µmol) were reacted as described in **GP-2** for 120 h. The crude product mixture was purified by column chromatography on silica gel (hexane:EtOAc = 10:1 → 1:1) to obtain the title compound **13i** (7.8 mg, 25%, 69% ee) as colorless oil and substrate **12i** (9.5 mg, 53%, 24% ee) as colorless oil.
The enantiomeric purity of the product was determined by HPLC analysis (Daicel CHIRALPAK AD3, hexane/*i*PrOH = 60:1, flow rate = 0.5 mL/min, retention time; 26.0 min (major) and 30.6 min (minor)).
The enantiomeric purity of the substrate was determined by HPLC analysis (Daicel CHIRALPAK ADH, hexane/EtOH = 20:1, flow rate = 1.0 mL/min, retention time; 19.7 min (minor) and 26.9 min (major)).

^{1}H NMR (400 MHz, $CDCl_3$) δ = 7.42 (d, *J* = 7.4, 2 H), 7.38 – 7.21 (m, 8 H), 5.63 (ddt, *J* = 17.4, 10.1, 7.2, 1 H), 5.10 – 4.96 (m, 2 H), 4.53 (s, 2 H), 3.63 (s, 2 H), 2.83 (s, 1 H), 2.74 – 2.55 (m, 2 H) ppm. ^{13}C NMR (100 MHz, $CDCl_3$) δ = 144.0, 138.1, 133.5, 128.6, 128.3, 127.9, 127.8, 127.1, 125.7, 118.7, 77.1, 75.7, 73.6, 44.0 ppm. HRMS (ESI) exact mass calcd. for $C_{18}H_{20}NaO_2$: *m/z* 291.1356 ([M + Na]$^+$), found *m/z* 291.1360 ([M + Na]$^+$). IR (neat): 3566, 2860, 1559, 1448, 1094, 913, 736 cm^{-1}. $[\alpha]_D^{28.3}$ = –10.4 (*c* = 0.78, $CHCl_3$).

1 mmol Reaction

(*S*)-2-((4-Methylbenzyl)oxy)-1-phenylethanol **2ad**

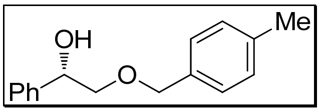

1-Phenylethane-1,2-diol (**1a**) (138 mg, 1.00 mmol), 1-(bromomethyl)-4-methylbenzene (740 mg, 4.00 mmol), borinic acid **8** (10.5 mg, 50 µmol), PTC (*S,S*)-**7** (29.6 mg, 20 µmol), NaI (750 mg, 5.00 mmol) and Cs_2CO_3 (650 mg, 2.00 mmol) in CH_2Cl_2/toluene/H_2O (1.5 ml/2.0 mL/ 6.5 mL) were reacted as described in **GP-1** for 90 h. The crude product mixture was purified by column chromatography on silica gel (hexane:EtOAc = 10:1 → 1:1) to obtain the title compound **2ad** (115 mg, 48%, 86% ee) as colorless oil along with the corresponding side-product **3ad** (27.7 mg, 11%) as colorless oil and substrate **1a** (48.6 mg, 35%, 89% ee) as a white solid.

The enantiomeric purity of the product was determined by HPLC analysis (Daicel CHIRALPAK AD3, hexane/*i*PrOH = 60:1, flow rate = 0.5 mL/min, retention time; 60.9 min (major) and 65.1 min (minor)).

The enantiomeric purity of the substrate was determined by HPLC analysis (Daicel CHIRALPAK ADH, hexane/EtOH = 20:1, flow rate = 1.0 mL/min, retention time; 29.5 min (minor) and 32.7 min (major)).

The analytical data were in agreement with the previous experiment.

Alkylation of Secondary Alcohol and Desymmetrization (Scheme 2)

(*R*)-2-(Benzyloxy)-2-phenylethanol **3aa**

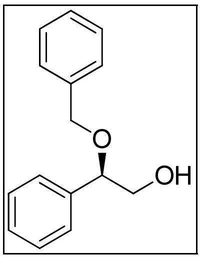

(*R*)-1-Phenylethane-1,2-diol (**1a**) (13.8 mg, 100 µmol, 87% ee), benzyl bromide (51 mg, 300 µmol) [after 48 h additional 300 µmol of benzyl bromide were added], borinic acid **8** (1.1 mg, 5 µmol), PTC (*S*)-**14** (4.5 mg, 4 µmol) and Cs_2CO_3 (65 mg, 200 µmol) were reacted as described in **GP-1** at ambient temperature for 93 h. The crude product mixture was purified by column chromatography on silica gel (hexane:EtOAc = 10:1 → 1:1) to obtain the title compound **3aa** (17.5 mg, 77%, 91% ee) as colorless oil along with the corresponding side-product **2aa** (5.2 mg, 23%, 75% ee) as colorless oil.

The enantiomeric purity of the product was determined by HPLC analysis (Daicel CHIRALPAK AD3, hexane/*i*PrOH = 60:1, flow rate = 1.0 mL/min, retention time; 22.4 min (major) and 25.8 min (minor)).

The enantiomeric purity of the side-product was determined by HPLC analysis (Daicel CHIRALPAK AD3, hexane/*i*PrOH = 60:1, flow rate = 1.0 mL/min, retention time; 31.1 min (major) and 32.8 min (minor)).

^{1}H NMR (500 MHz, $CDCl_3$) δ = 7.44 – 7.27 (m, 10 H), 4.61 – 4.50 (m, 2 H), 4.35 (d, *J* = 11.5, 1 H), 3.79 – 3.69 (m, 1 H), 3.69 – 3.59 (m, 1 H), 2.35 – 2.22 (m, 1 H) ppm. ^{13}C NMR (125 MHz, $CDCl_3$) δ = 138.5, 138.1, 128.8, 128.6, 128.4, 128.0, 127.9, 127.1, 82.4, 70.9, 67.5 ppm. HRMS (ESI) exact mass calcd. for $C_{15}H_{16}NaO_2$: *m/z* 251.1043 ([M + Na]$^+$), found *m/z* 251.1043 ([M + Na]$^+$). IR (neat): 3414, 2868, 1453, 1348, 1100, 698 cm^{-1}. $[\alpha]_D^{28.3}$ = –85.3 (*c* = 1.77, $CHCl_3$).

(1*S*,2*R*)-2-((2-methylbenzyl)oxy)-1,2-diphenylethanol **16**

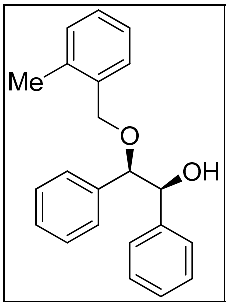

meso-1,2-diphenylethane-1,2-diol (**15**) (21.4 mg, 100 µmol), 1-(bromomethyl)-4-methylbenzene (68 mg, 400 µmol), borinic acid **8** (2.1 mg, 10 µmol), PTC (*S,S*)-**7** (6.0 mg, 4 µmol), and Cs_2CO_3 (65 mg, 200 µmol) were reacted as described in **GP-2** for 50 h. The crude product mixture was purified by column chromatography on silica gel (hexane:EtOAc = 10:1 → 6:1) to obtain the title compound **16** (30.6 mg, 96%, 52% ee) as a white solid.

The enantiomeric purity of the product was determined by HPLC analysis (Daicel CHIRALPAK IC3, hexane/*i*PrOH = 60:1, flow rate = 0.5 mL/min, retention time; 26.4 min (minor) and 28.0 min (major)).

^{1}H NMR (500 MHz, $CDCl_3$) δ = 7.35 – 7.28 (m, 3 H), 7.27 – 7.14 (m, 8 H), 7.09 (m, 3 H), 4.84 (d, *J* = 6.1, 1 H), 4.45 (dd, *J* = 10.5, 9.0, 2 H), 4.21 (d, *J* = 11.8, 1 H) ppm. ^{13}C NMR (125 MHz, $CDCl_3$) δ = 140.8, 138.1, 137.1, 135.9, 130.3, 129.0, 128.4, 128.3, 128.3, 128.1, 128.0, 127.8, 127.3, 125.8, 85.3, 77.3, 69.3, 18.6 ppm. HRMS (ESI) exact mass calcd. for $C_{22}H_{22}NaO_2$: *m/z* 341.1512 ($[M + Na]^+$), found *m/z* 341.1502 ($[M + Na]^+$). IR (neat): 3447, 2870, 1453, 1085, 1060, 1028 cm^{-1}. $[\alpha]_D^{26.0}$ = –20.7 (*c* = 0.8, $CHCl_3$).

The absolute configuration was determined by comparing the benzyl bromide analog of compound **13**, which was obtained in 50% ee with a specific rotation of $[\alpha]_D^{29.0}$ = –25.1 (*c* = 0.5, $CHCl_3$), with the reported one in literature.[5]

NMR Experiment (Figure 2)

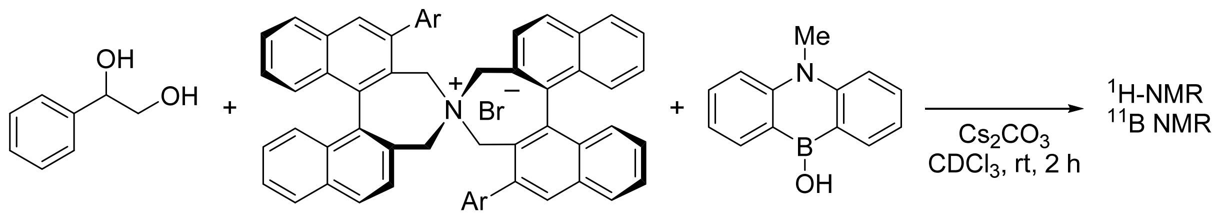

1a Ar = 3,5-(iC_3F_7)$_2$$C_6H_3$ (**7**) **8**

Experiment 1: (*rac*)-**1a** (2 eq.)
Experiment 2: (*R*)-**1a** (2 eq.) *mismatched*
Experiment 3: (*S*)-**1a** (2 eq.) *matched*
Experiment 4: (*rac*)-**1a** (7 eq.)

Experiment 1 *rac*-Diol

A solution of 1-phenylethane-1,2-diol (**1a**) (1.38 mg, 10 µmol), borinic acid **8** (1.1 mg, 5 µmol), PTC (*S,S*)-**7** (7.4 mg, 5 µmol) and Cs_2CO_3 (32.5 mg, 100 µmol) in $CDCl_3$ (0.8 mL) was stirred at room temperature for 2 h. The base was filtered off and the NMR was measured immediately from the crude reaction mixture.

Experiment 2 (*R*)-Diol (mismatched case)

A solution of (*R*)-1-phenylethane-1,2-diol (**1a**) (1.38 mg, 10 µmol), borinic acid **8** (1.1 mg, 5 µmol), PTC (*S,S*)-**7** (7.4 mg, 5 µmol) and Cs_2CO_3 (32.5 mg, 100 µmol) in $CDCl_3$ (0.8 mL) was stirred at room temperature for 2 h. The base was filtered off and the NMR was measured immediately from the crude reaction mixture.

Experiment 3 (*S*)-Diol (matched case)

A solution of (*S*)-1-phenylethane-1,2-diol (**1a**) (1.38 mg, 10 µmol), borinic acid **8** (1.1 mg, 5 µmol), PTC (*S,S*)-**7** (7.4 mg, 5 µmol) and Cs_2CO_3 (32.5 mg, 100 µmol) in $CDCl_3$ (0.8 mL) was stirred at room temperature for 2 h. The base was filtered off and the NMR was measured immediately from the crude reaction mixture.

Experiment 4 *rac*-Diol excess

To the resulting solution obtained from **Experiment 1**, 1-phenylethane-1,2-diol (**1a**) (6.90 mg, 50 µmol) was added.

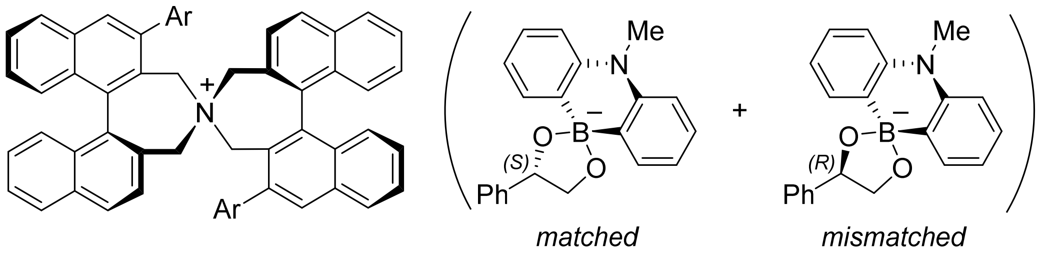

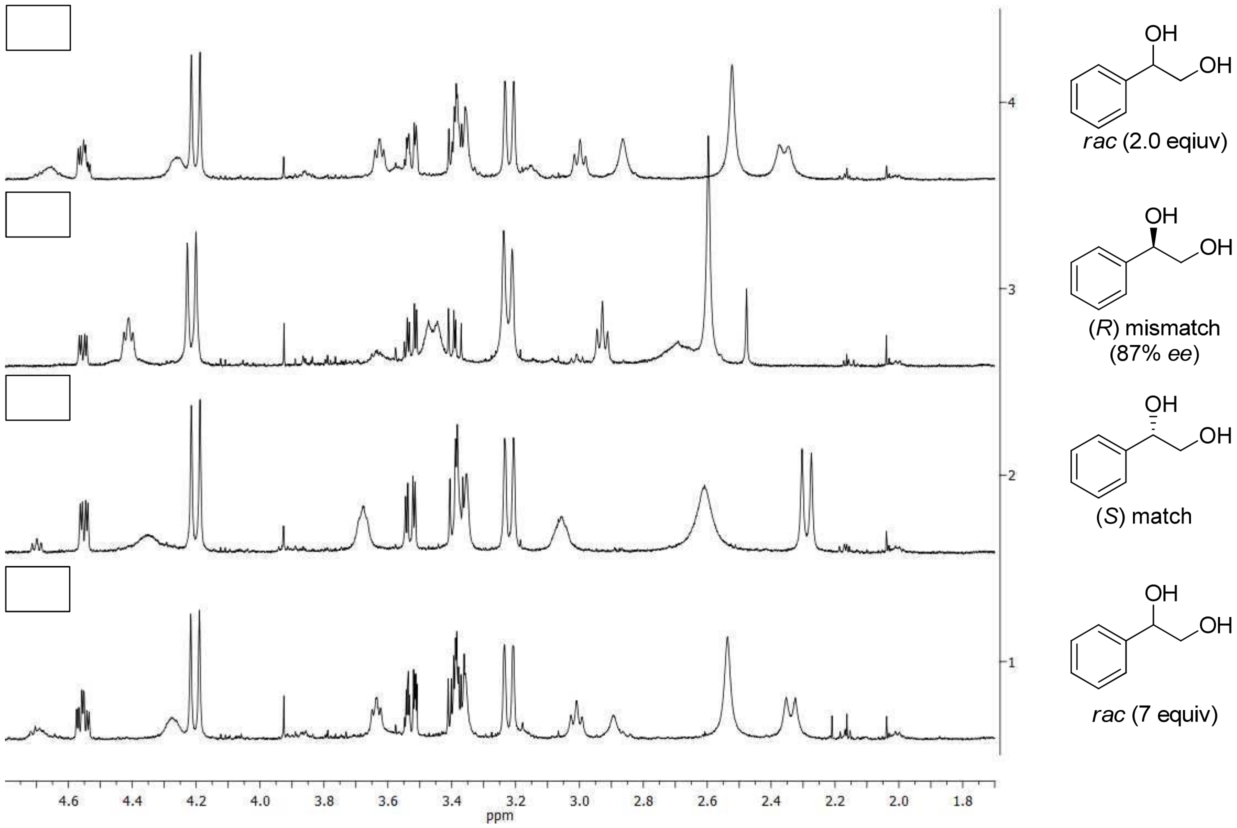

^{11}B-NMR of **Experiment 1** displays the formation of the borinate.

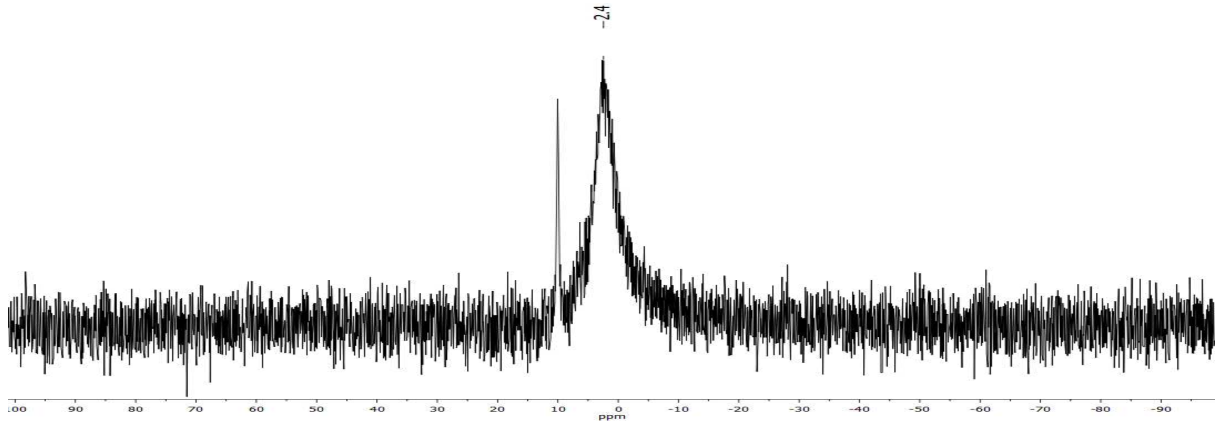

11B NMR (160 MHz, $CDCl_3$)

References

[1] T. Furuta, M. Nikaido, J. Yamamoto, T. Kuribayashi, T. Kawabata, *Synthsis*, **2013**, *45*, 1312.

[2] A. Arlt, H. Toyama, K. Takada, T. Hashimoto, K. Maruoka, *Chem. Commun*., **2017,** *53*, 4779.

[3] B. Balagam, R. Mitra, D. E. Richardson, *Tetrahedron Lett*., **2008**, *49*, 1071.

[4] J. H. Kim, I. Čorić, C. Palumbo, B. List, *J. Am. Chem. Soc.*, **2015**, *137*, 1778.

[5] F. A. Davis, M.S. Haque, R. M. Przeslawski, *J. Org. Chem.*, **1989**, *54*, 2021.

NMR Spectra

PTC (*S*)-Precursor **10**

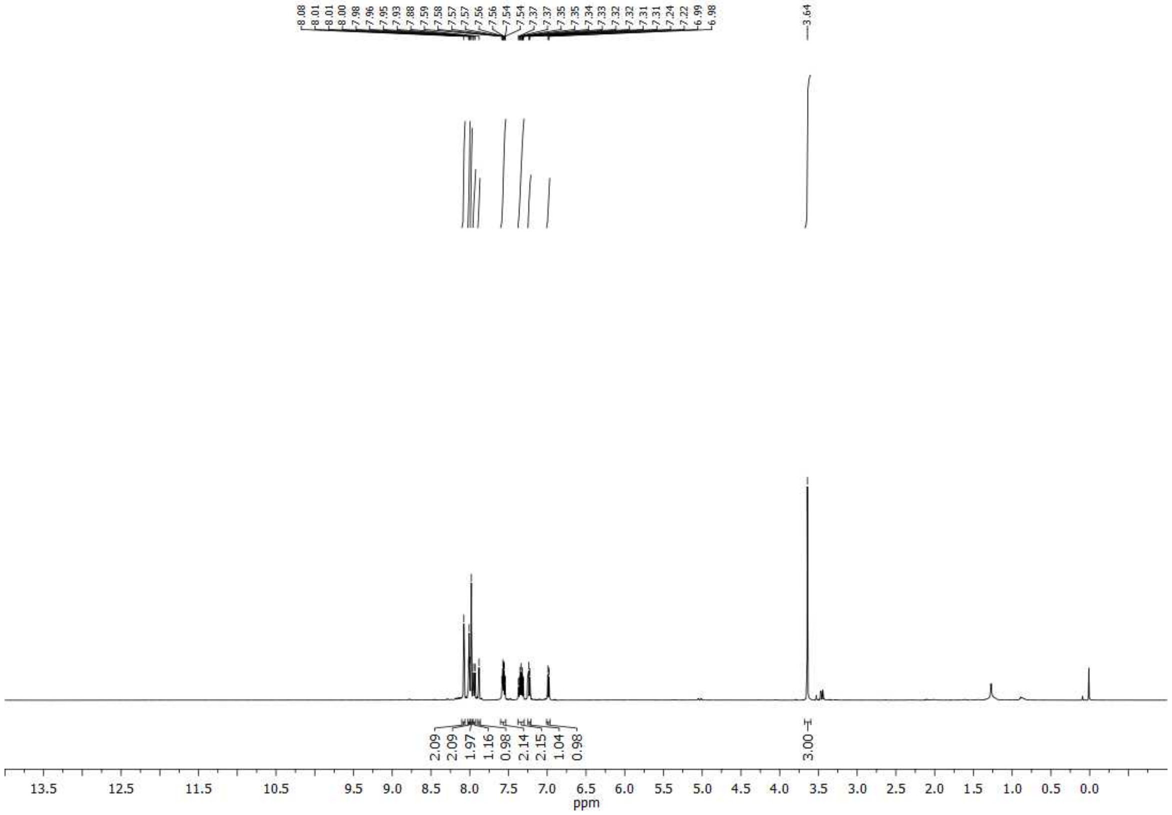

^{1}H NMR (500 MHz, $CDCl_3$)

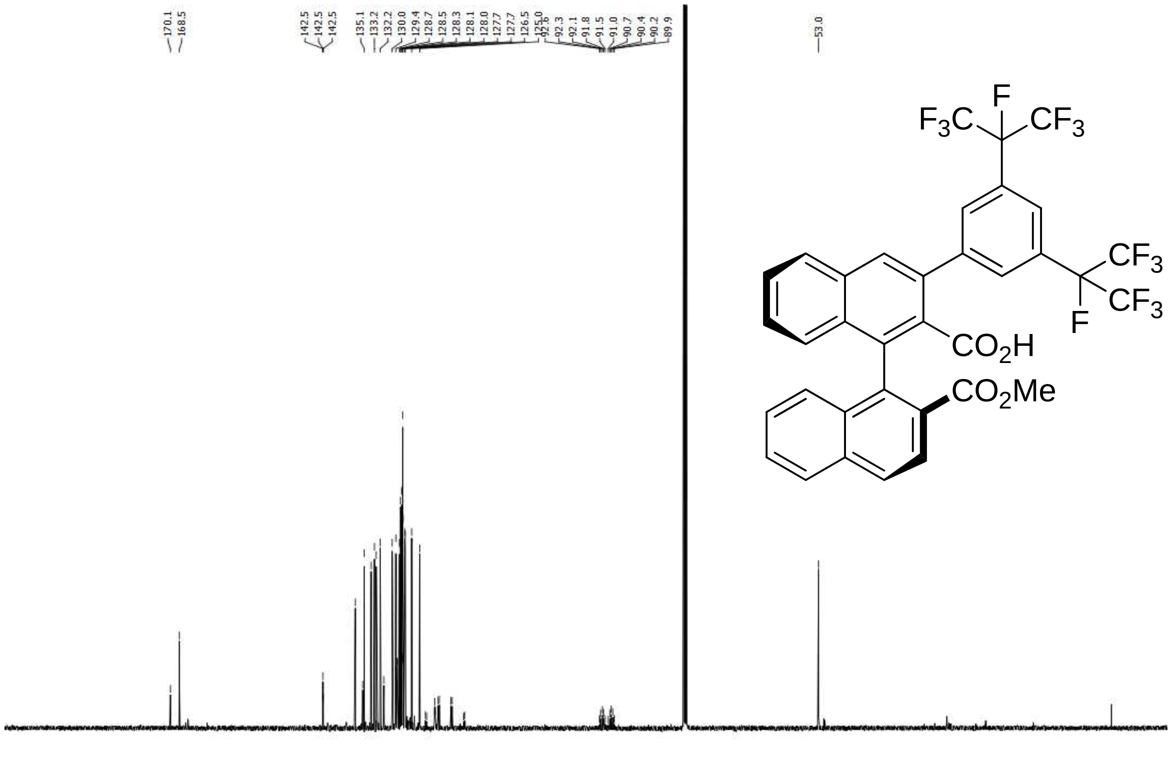

^{13}C NMR (125 MHz, $CDCl_3$)

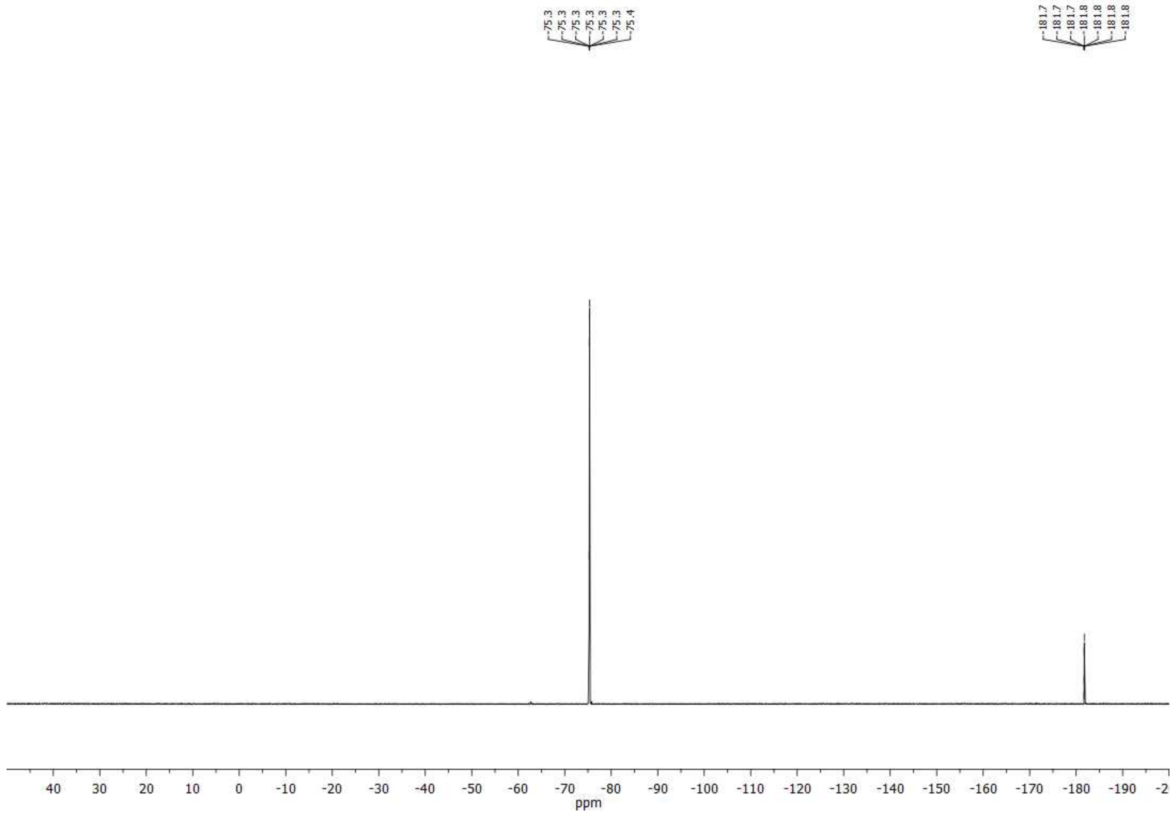

^{19}F NMR (466 MHz, $CDCl_3$)

PTC (*S*)-Precursor **18**

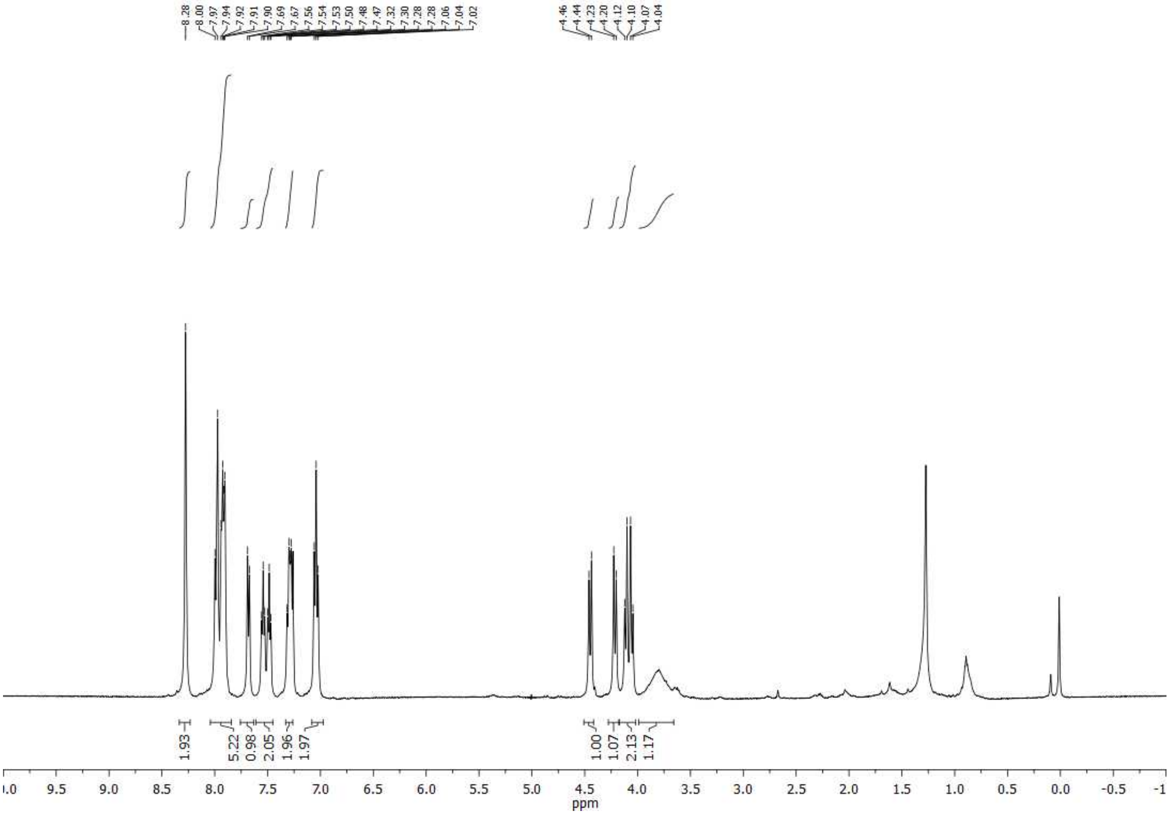

^{1}H NMR (500 MHz, $CDCl_3$)

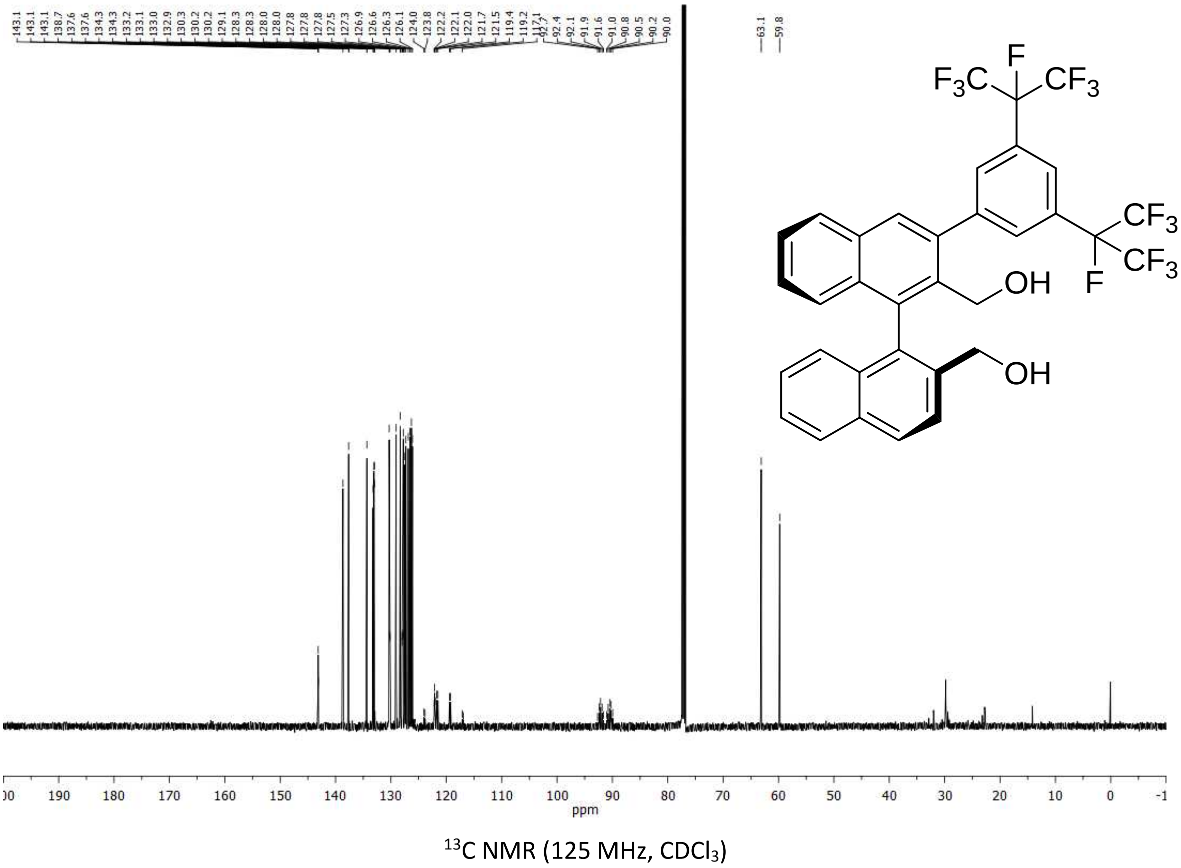

^{13}C NMR (125 MHz, $CDCl_3$)

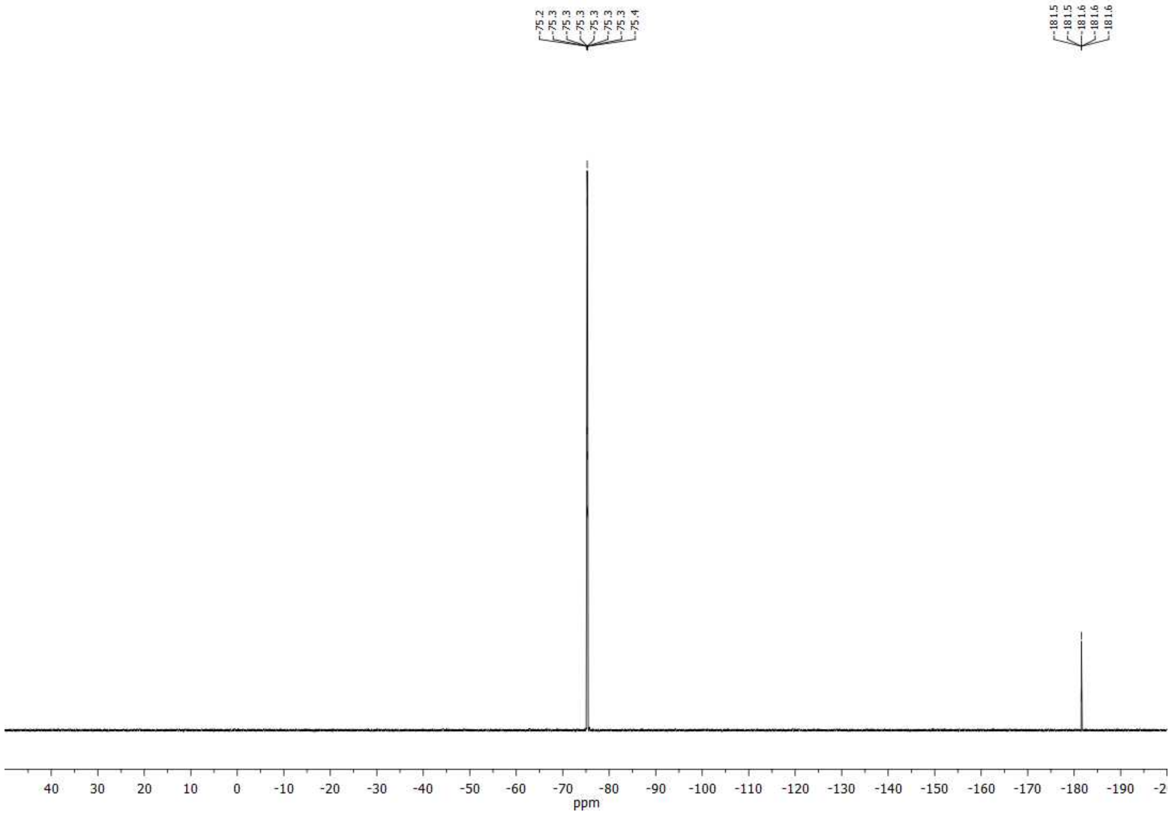

^{19}F NMR (466 MHz, $CDCl_3$)

PTC (*S*)-Precursor **11**

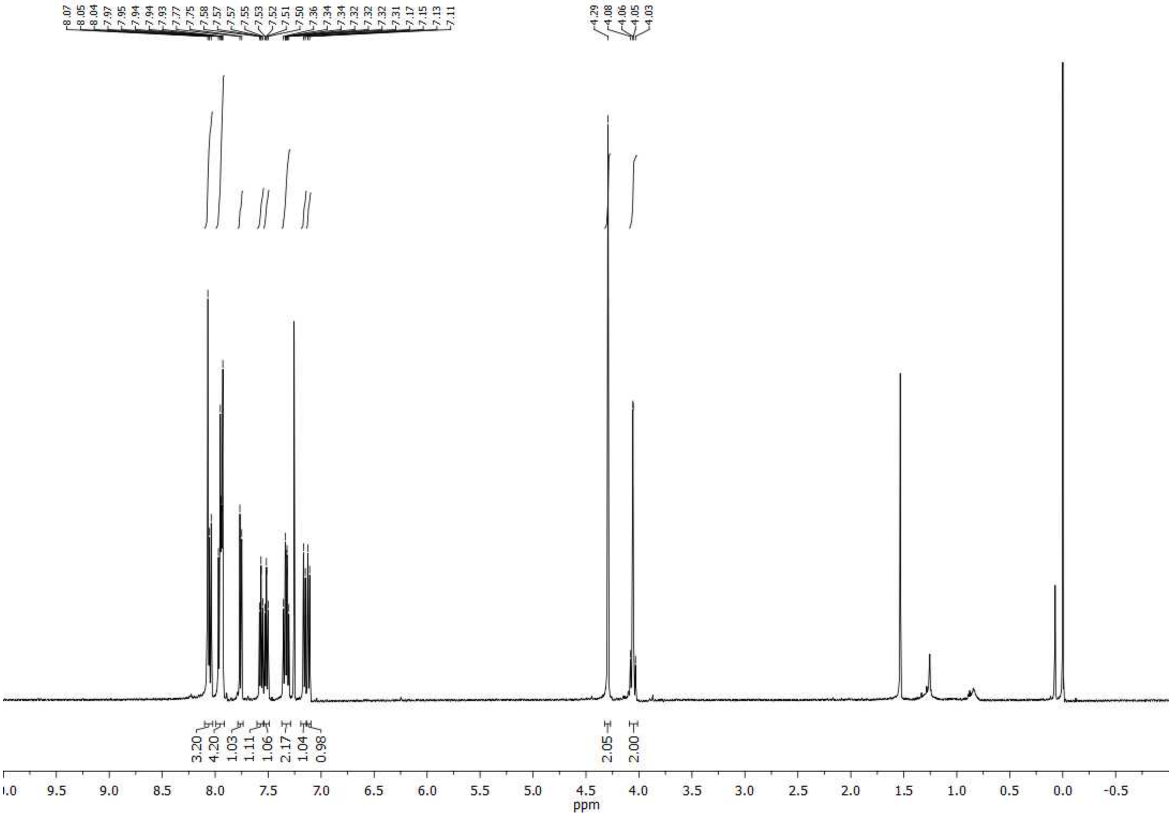

^{1}H NMR (500 MHz, $CDCl_3$)

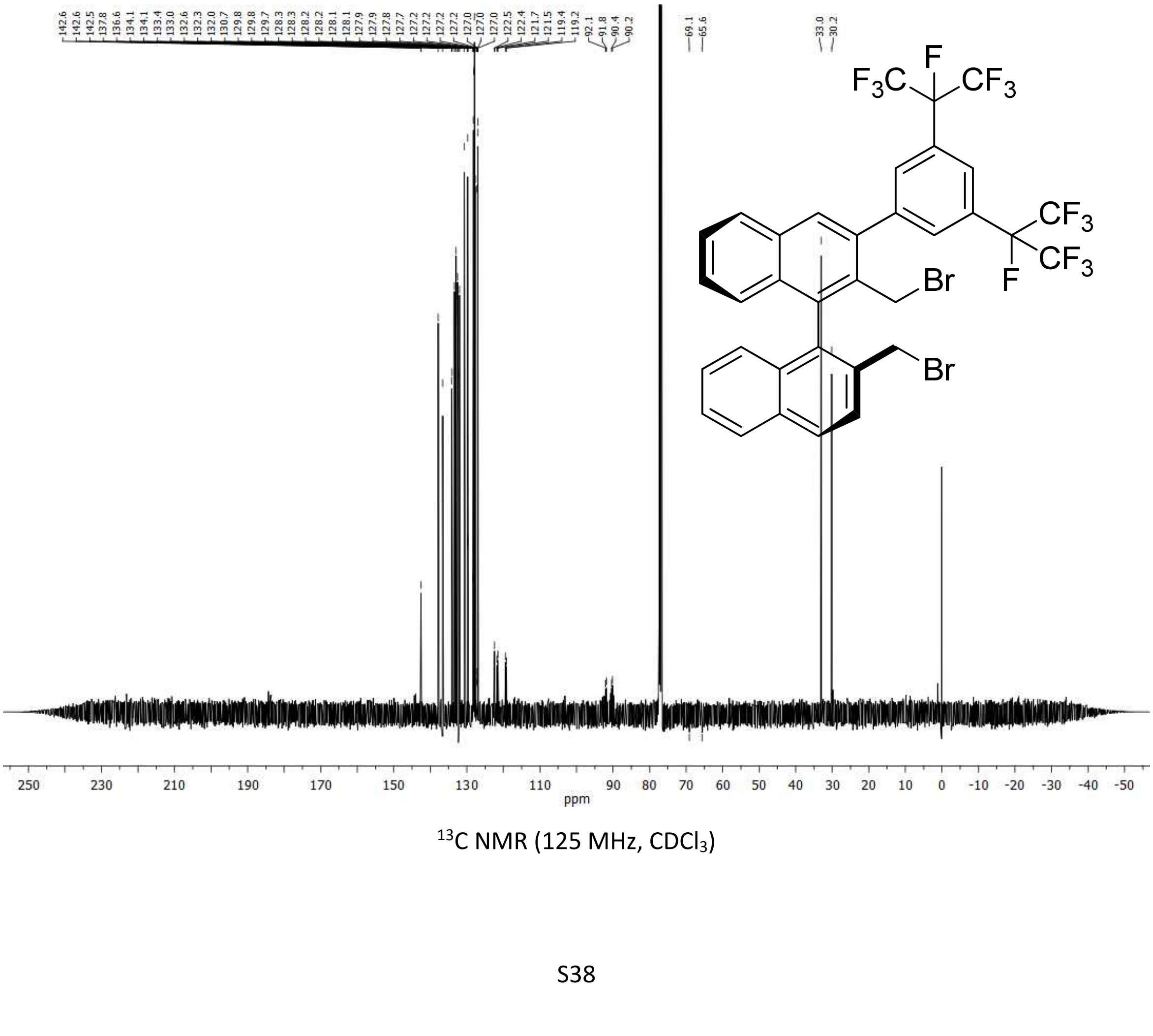

^{13}C NMR (125 MHz, $CDCl_3$)

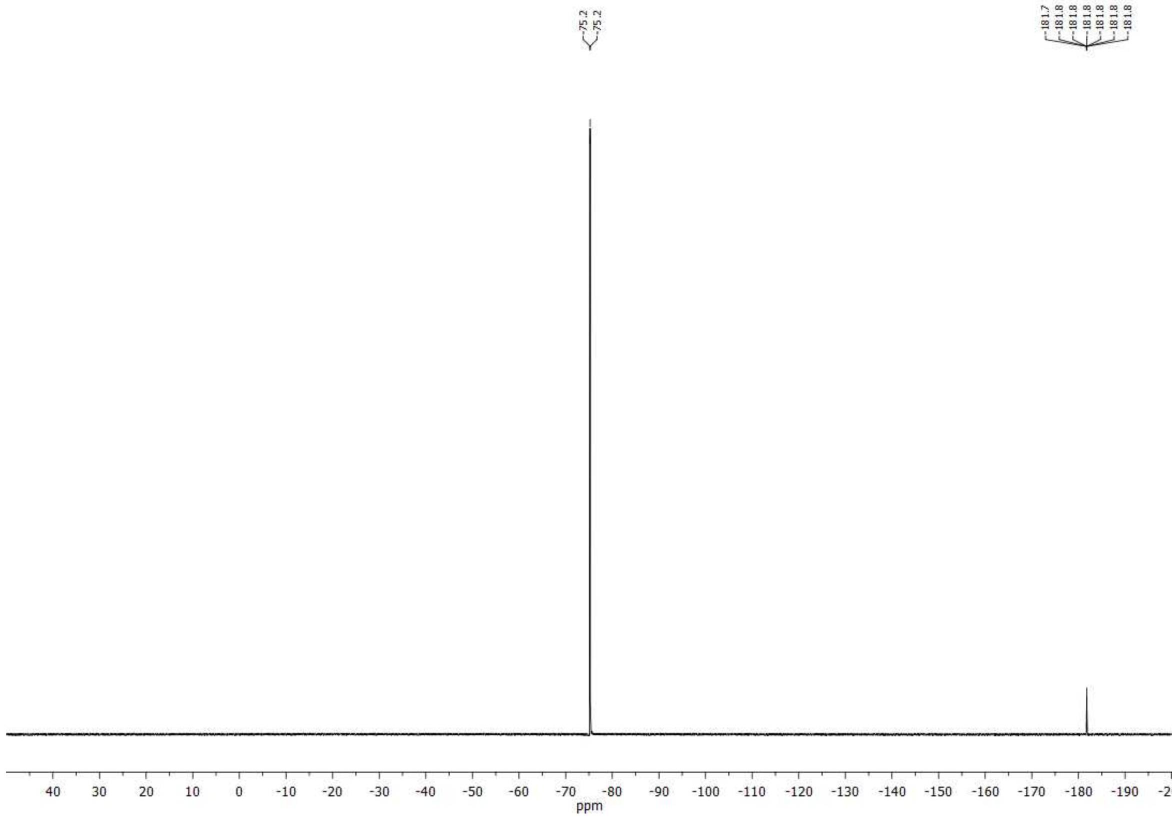

^{19}F NMR (466 MHz, $CDCl_3$)

PTC (*S*,*S*)-**7**

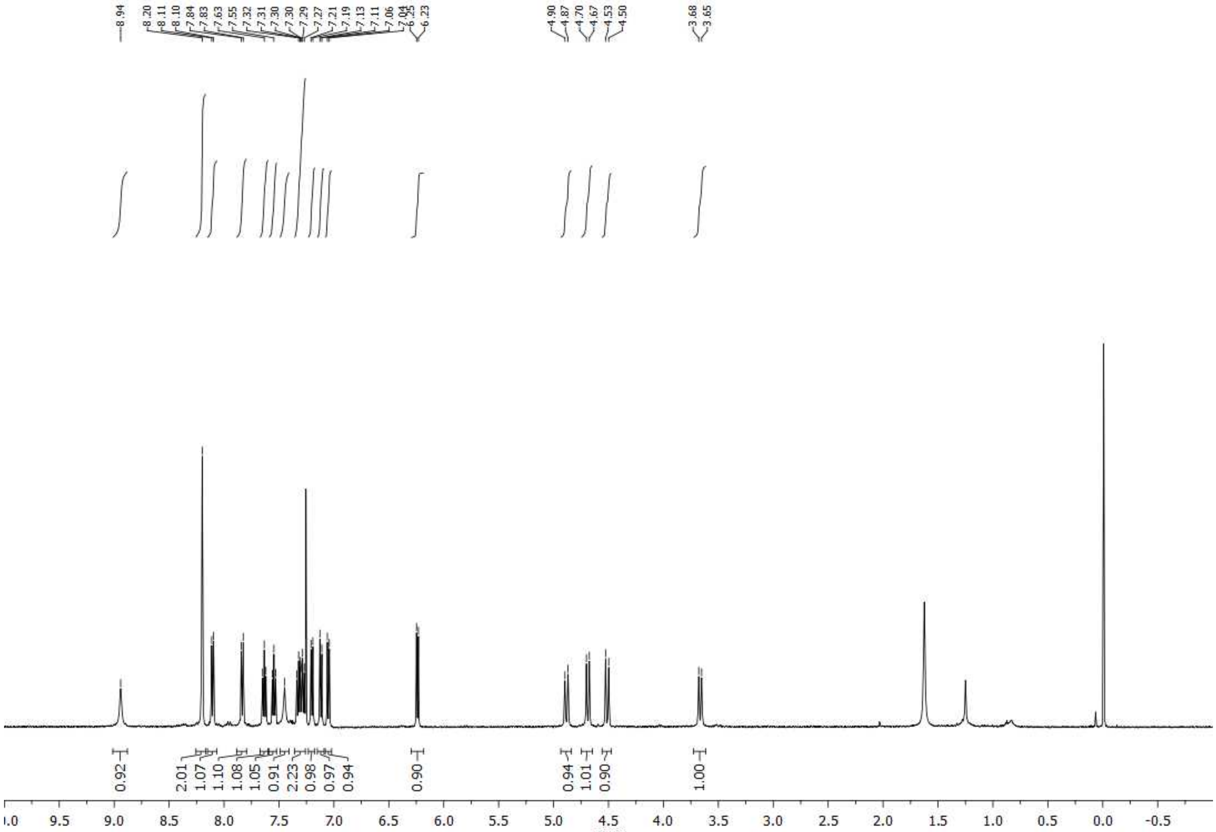

^{1}H NMR (500 MHz, CDCl$_3$)

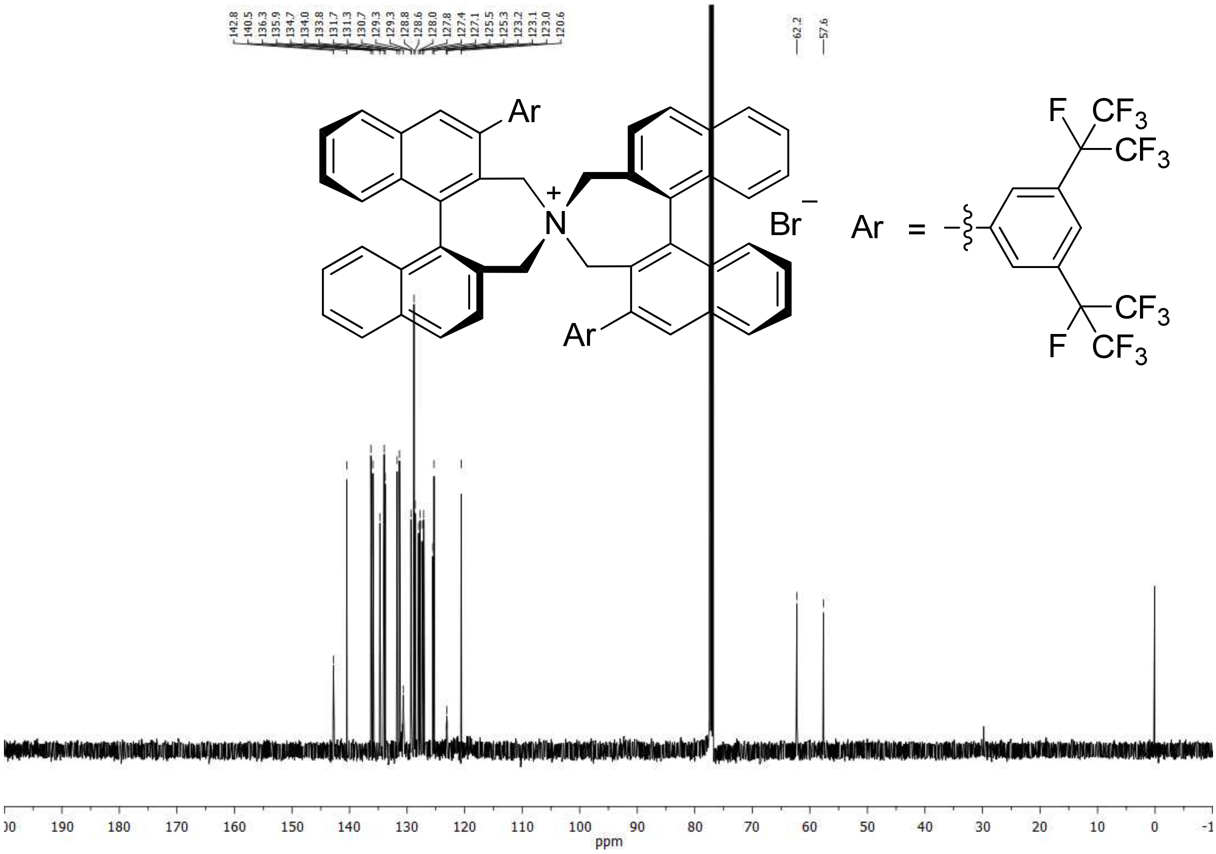

^{13}C NMR (125 MHz, CDCl$_3$)

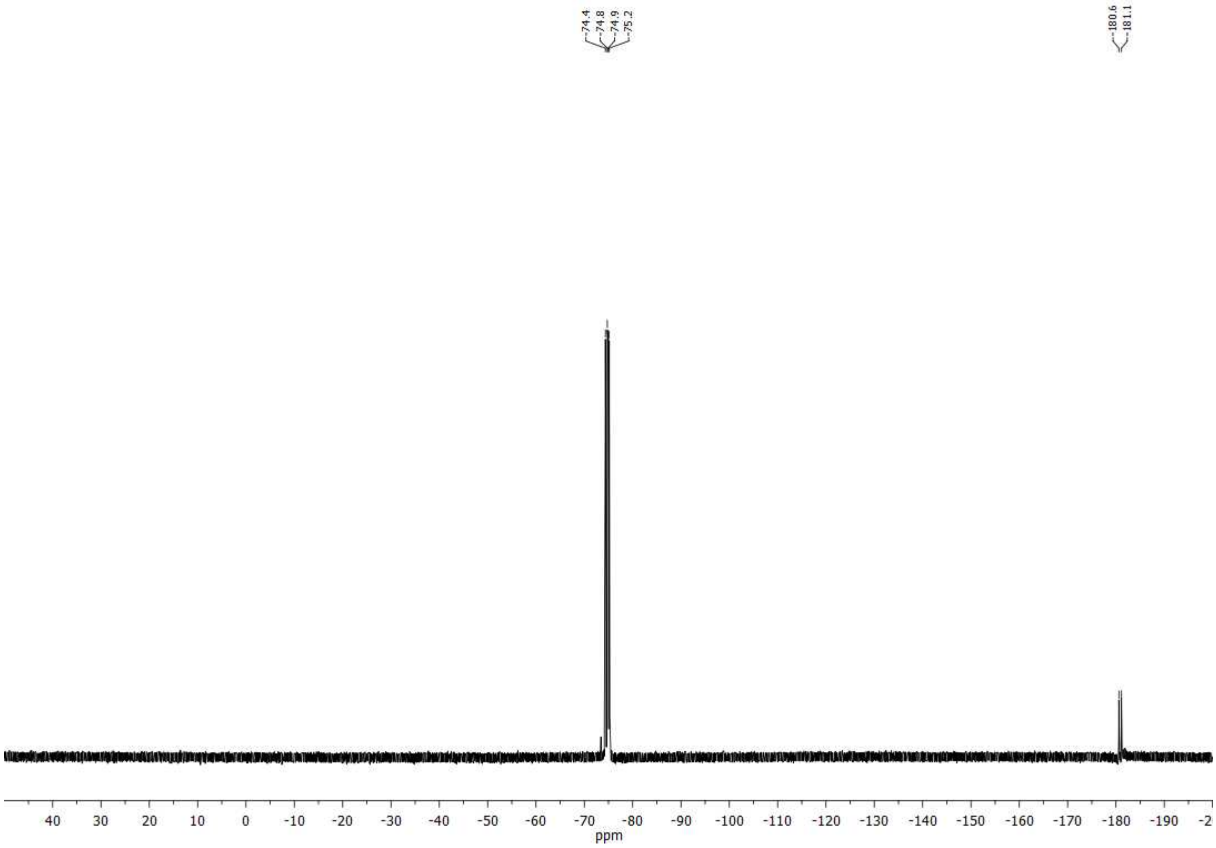

^{19}F NMR (466 MHz, $CDCl_3$)

(*S*)-2-(Benzyloxy)-1-phenylethanol (**2aa**)

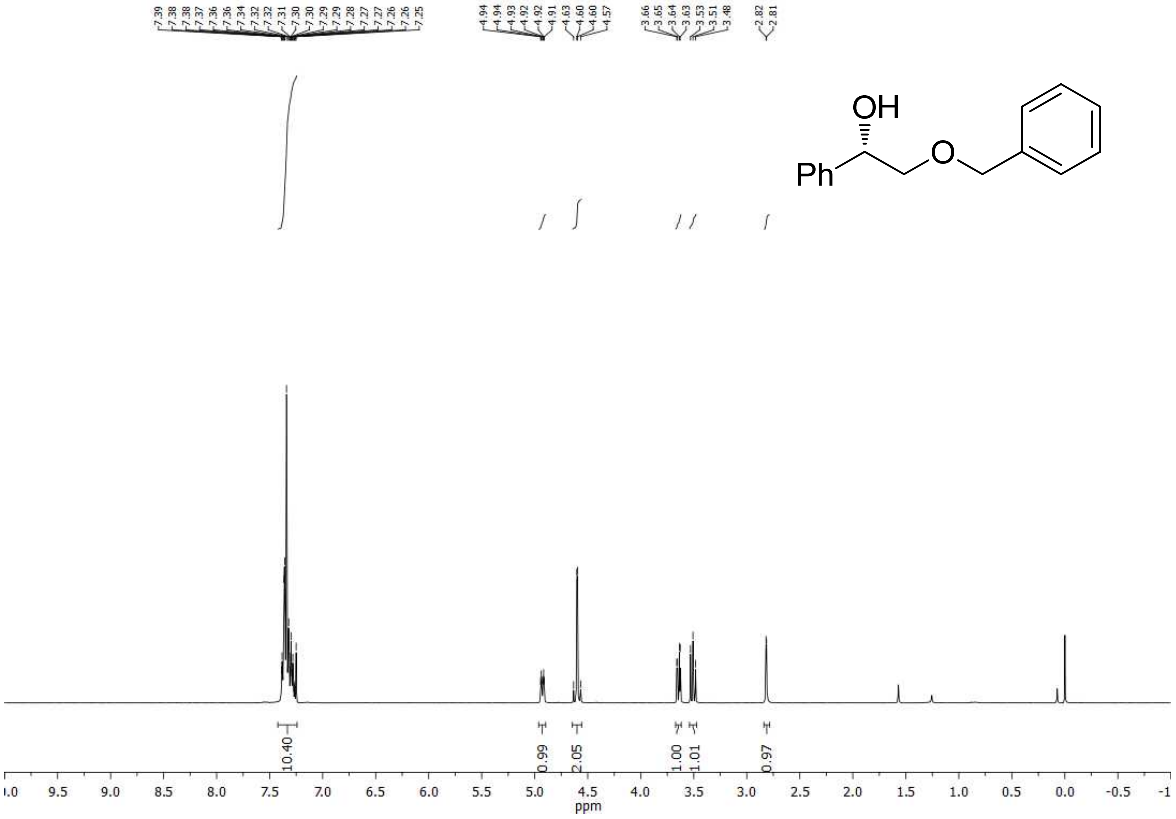

^{1}H-NMR (400 MHz, $CDCl_3$)

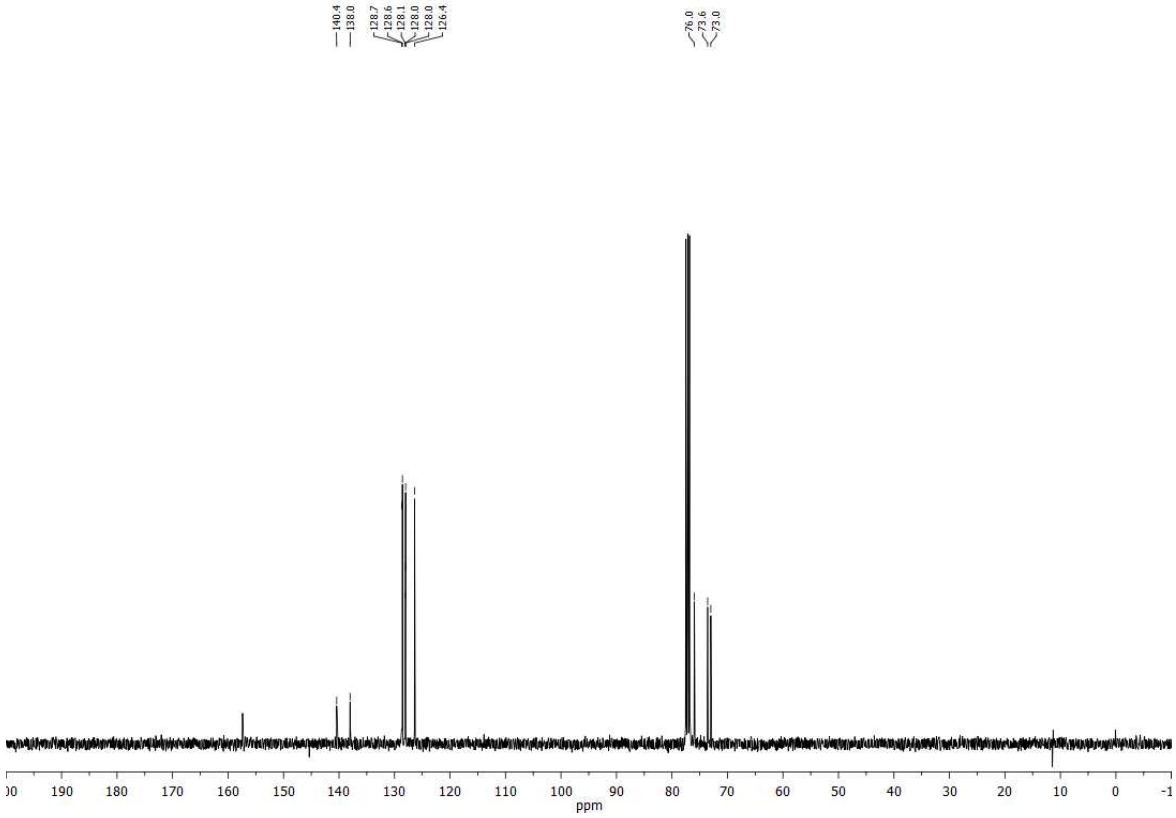

^{13}C NMR (100 MHz, $CDCl_3$)

(*S*)-2-((2-Methylbenzyl)oxy)-1-phenylethanol (**2ab**)

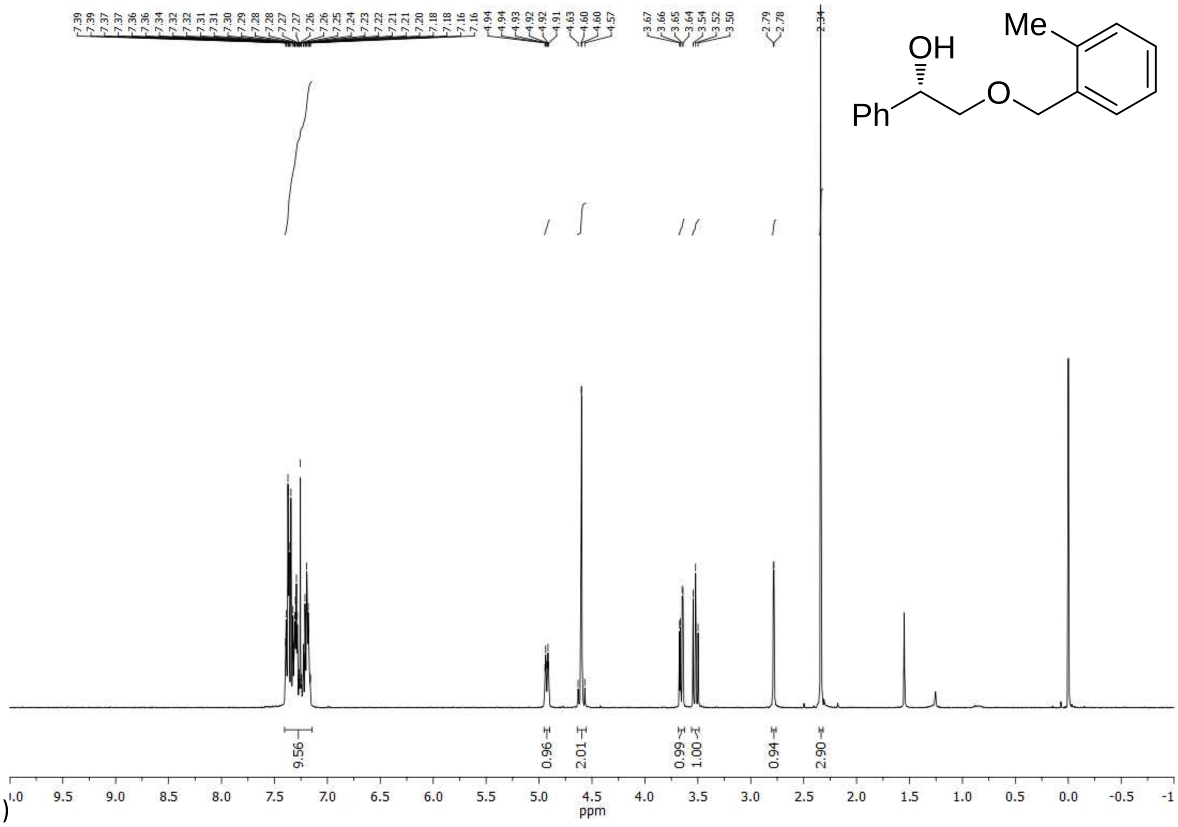

^{1}H NMR (400 MHz, $CDCl_3$)

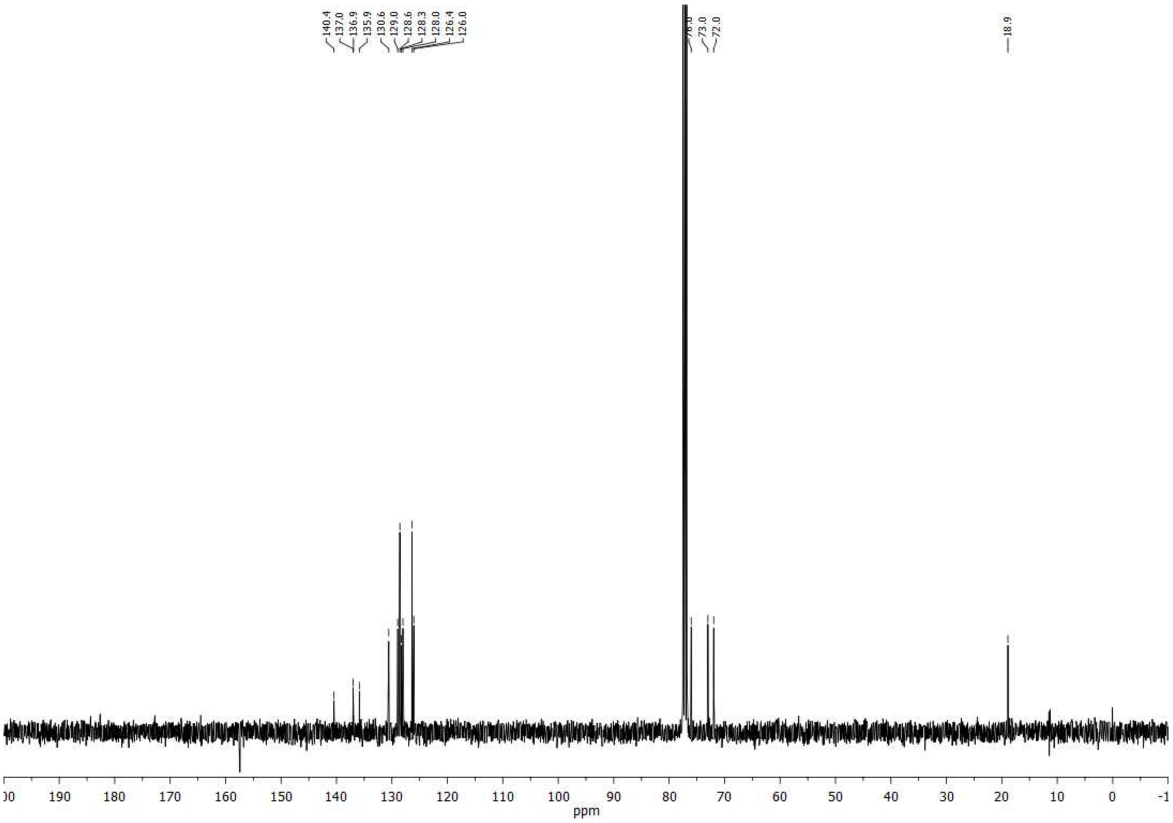

^{13}C NMR (100 MHz, $CDCl_3$)

(*S*)-2-((3-Methylbenzyl)oxy)-1-phenylethanol (2**ac**)

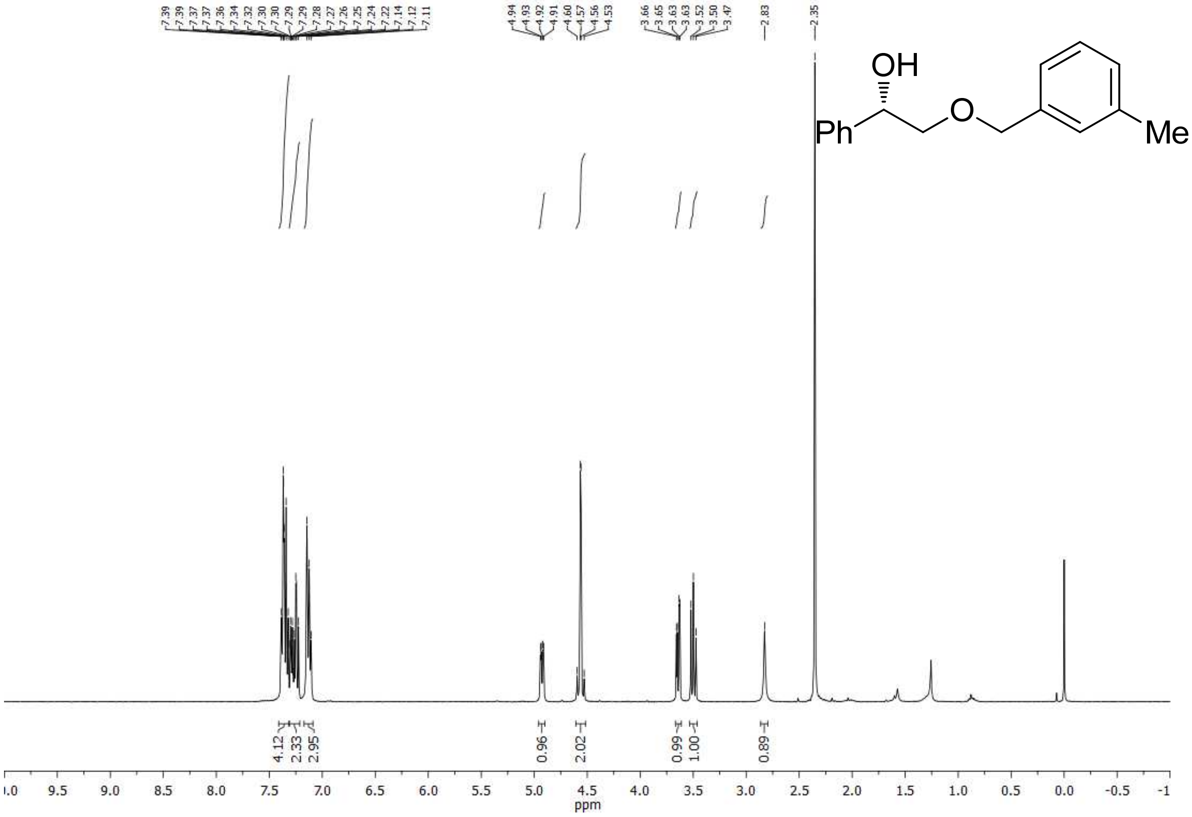

^{1}H NMR (400 MHz, $CDCl_3$)

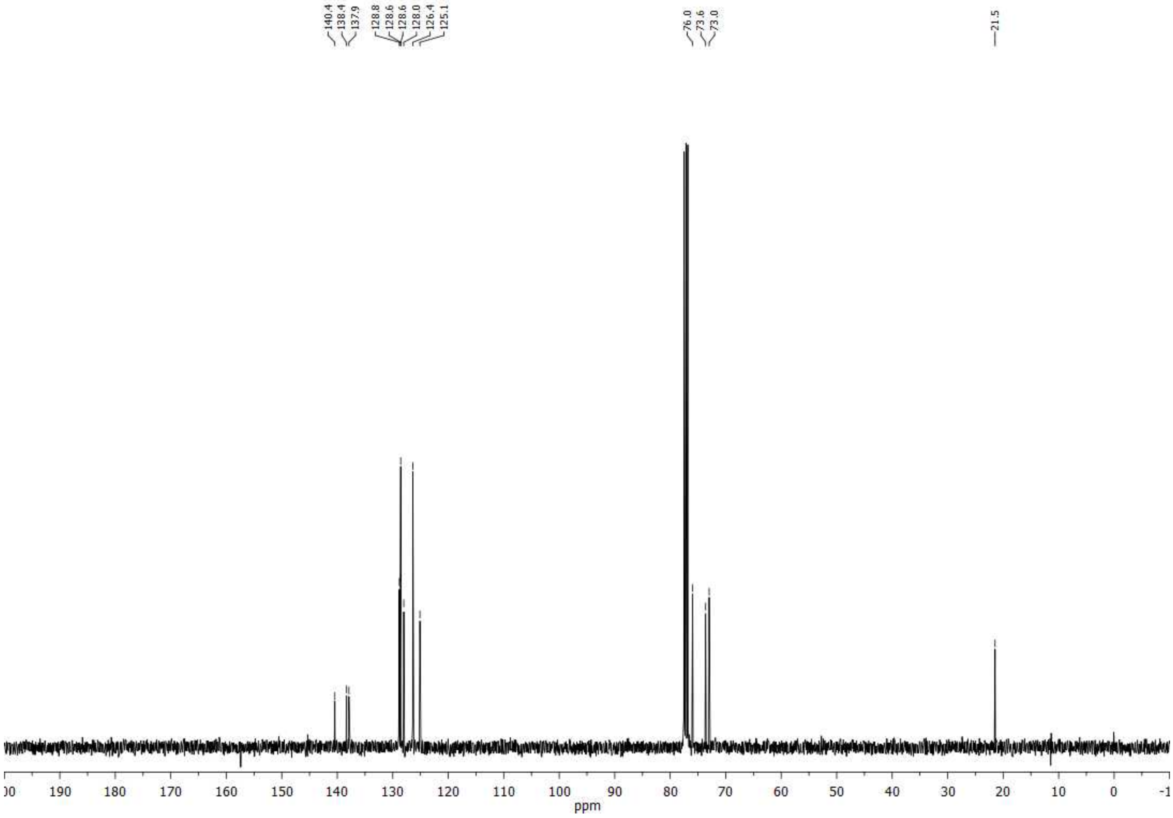

NMR (100 MHz, $CDCl_3$)

(*S*)-2-((4-Methylbenzyl)oxy)-1-phenylethanol (**2ad**)

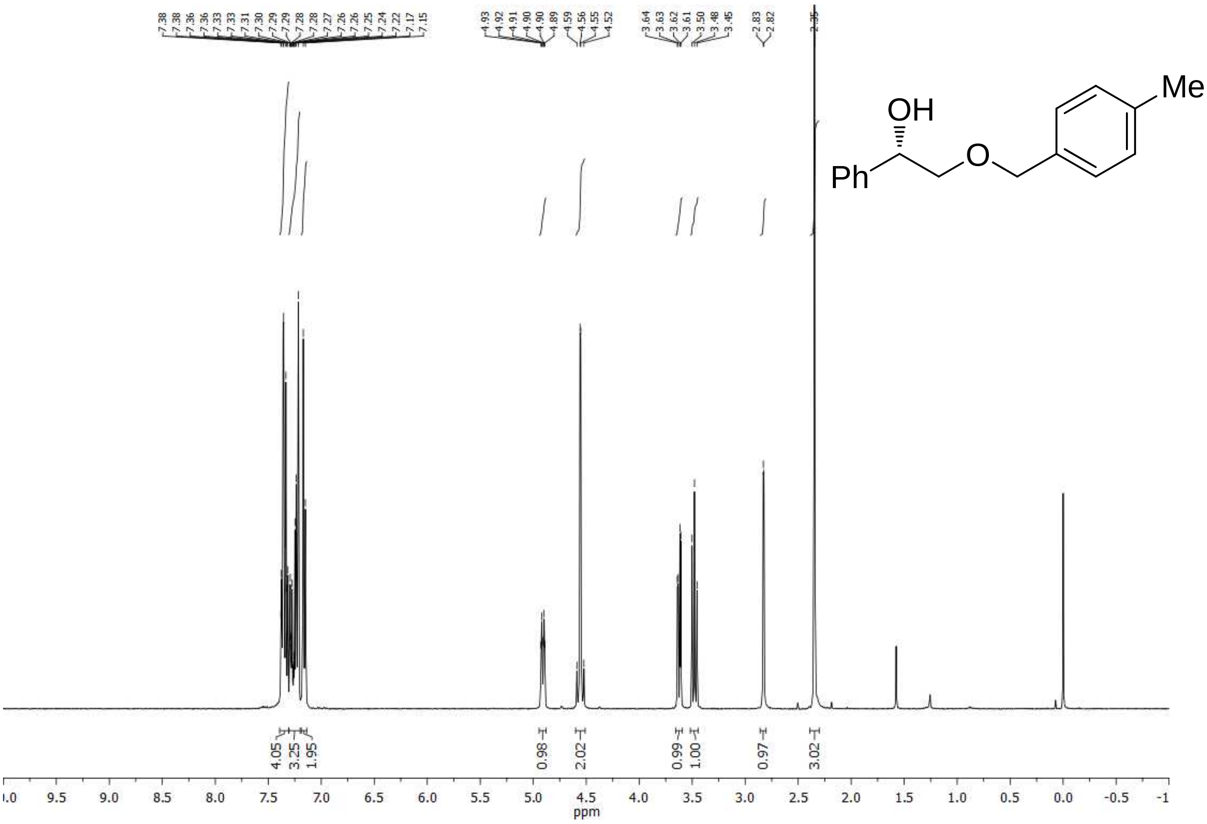

^{1}H NMR (400 MHz, $CDCl_3$)

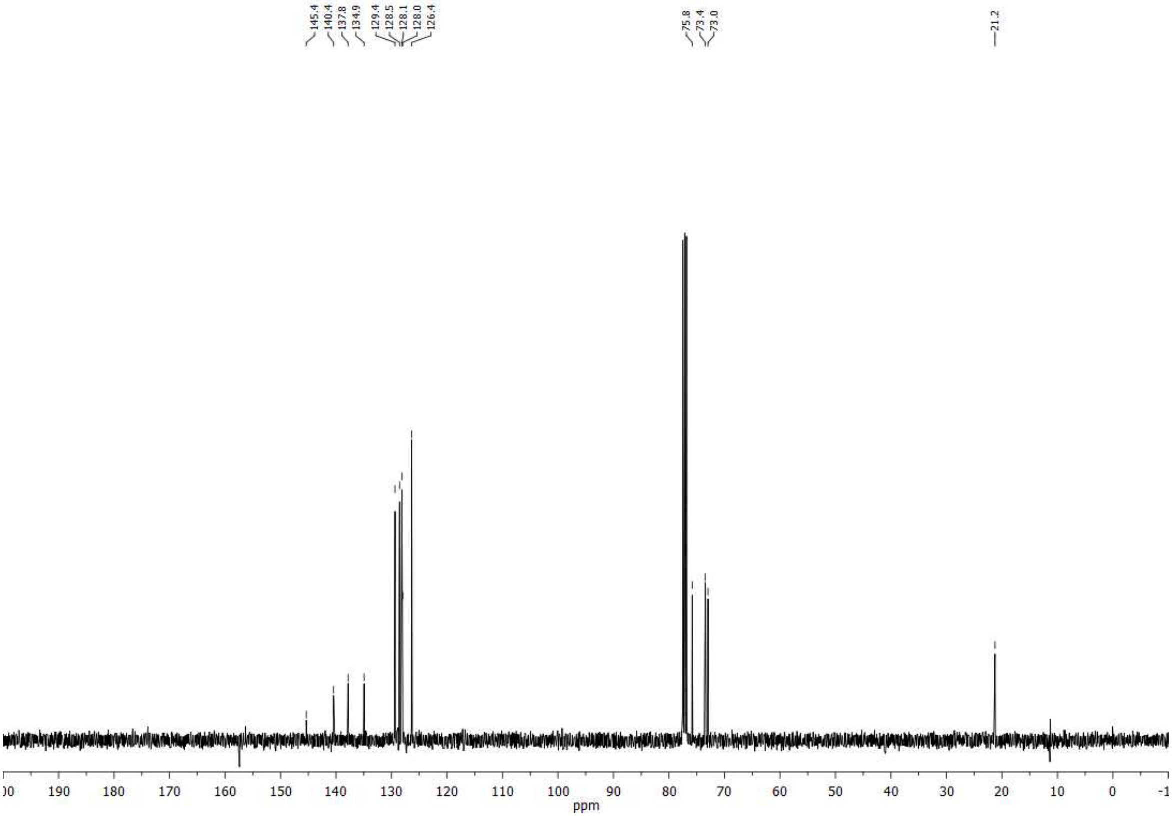

^{13}C NMR (100 MHz, $CDCl_3$)

(*S*)-2-((2-Fluorobenzyl)oxy)-1-phenylethanol (**2ae**)

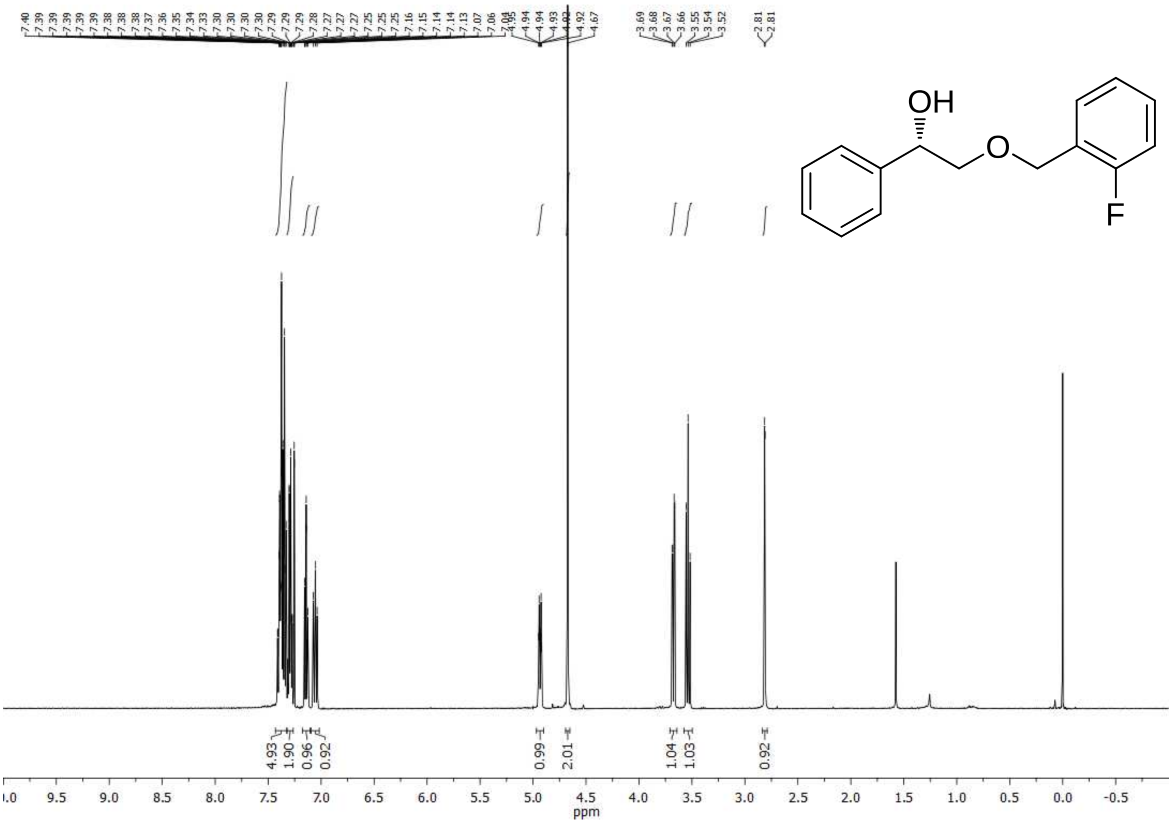

^{1}H-NMR (500 MHz, CDCl$_3$)

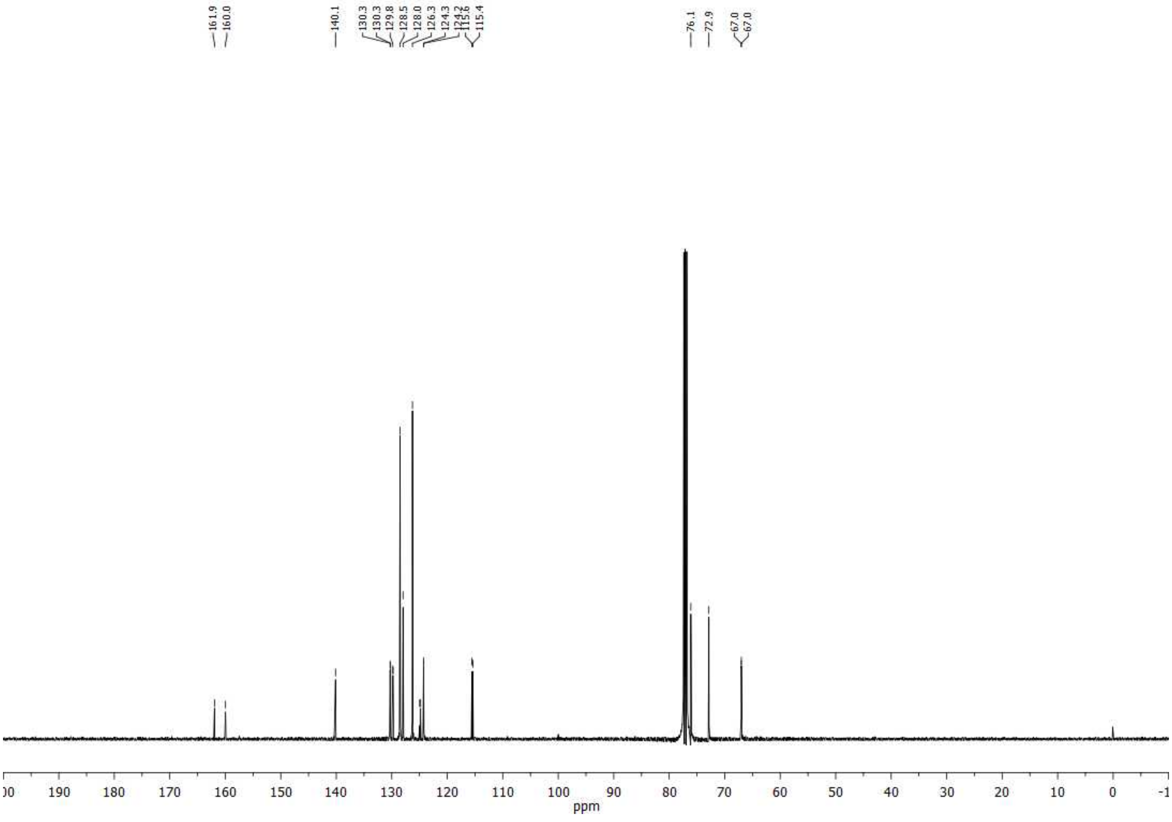

^{13}C NMR (125 MHz, CDCl$_3$)

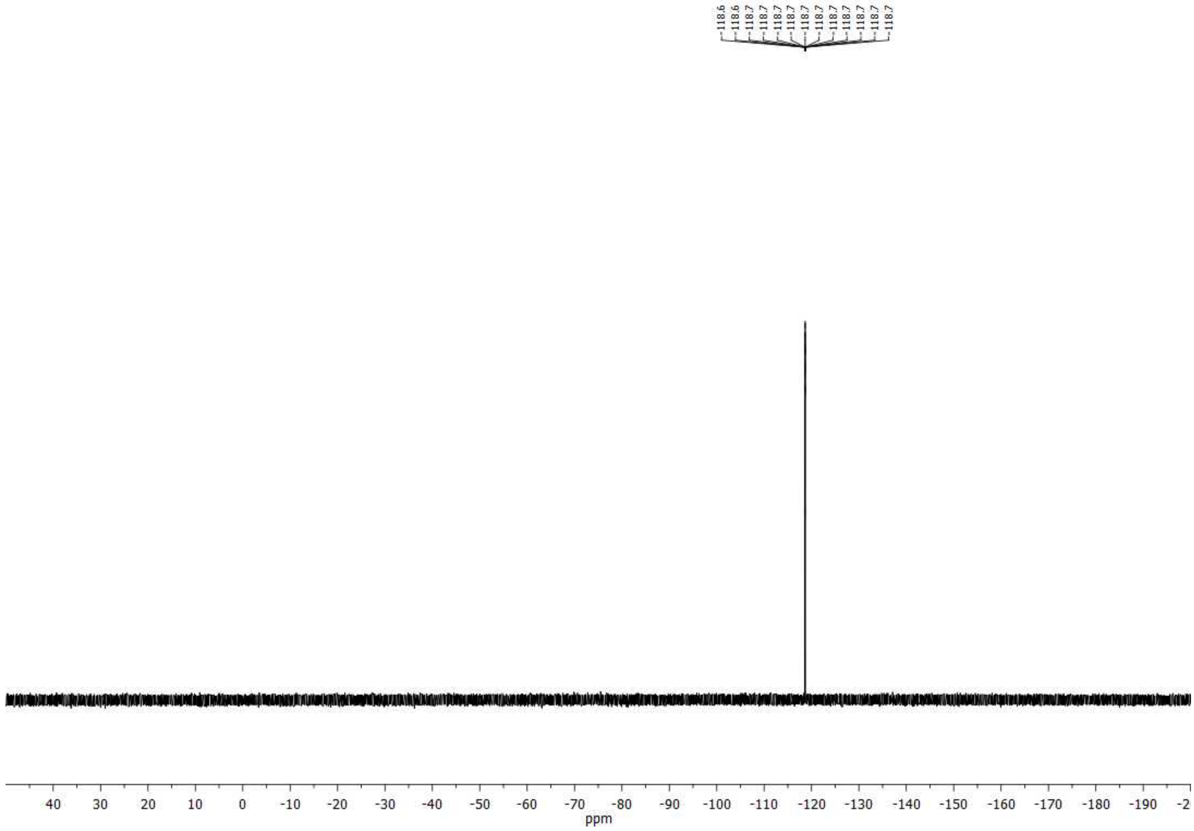

^{19}F NMR (466 MHz, $CDCl_3$

(*S*)-2-((2-Bromobenzyl)oxy)-1-phenylethanol (**2af**)

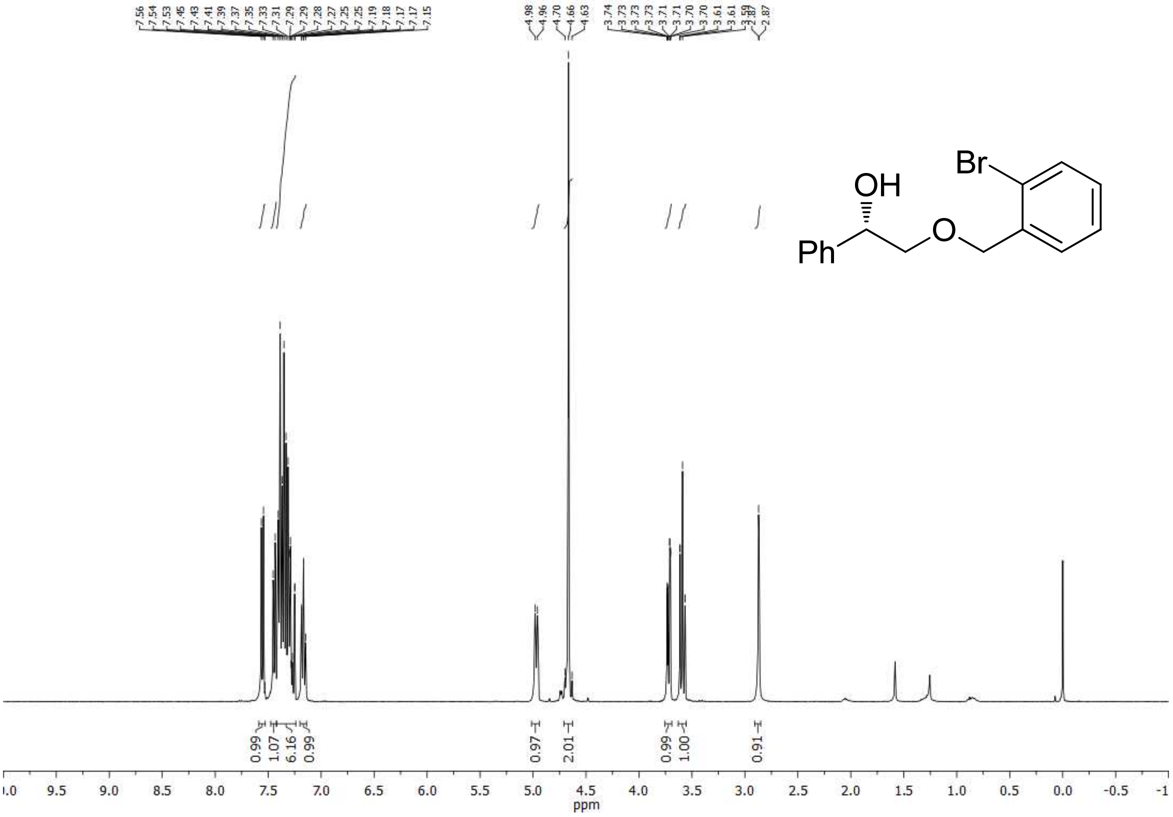

^{1}H NMR (400 MHz, $CDCl_3$)

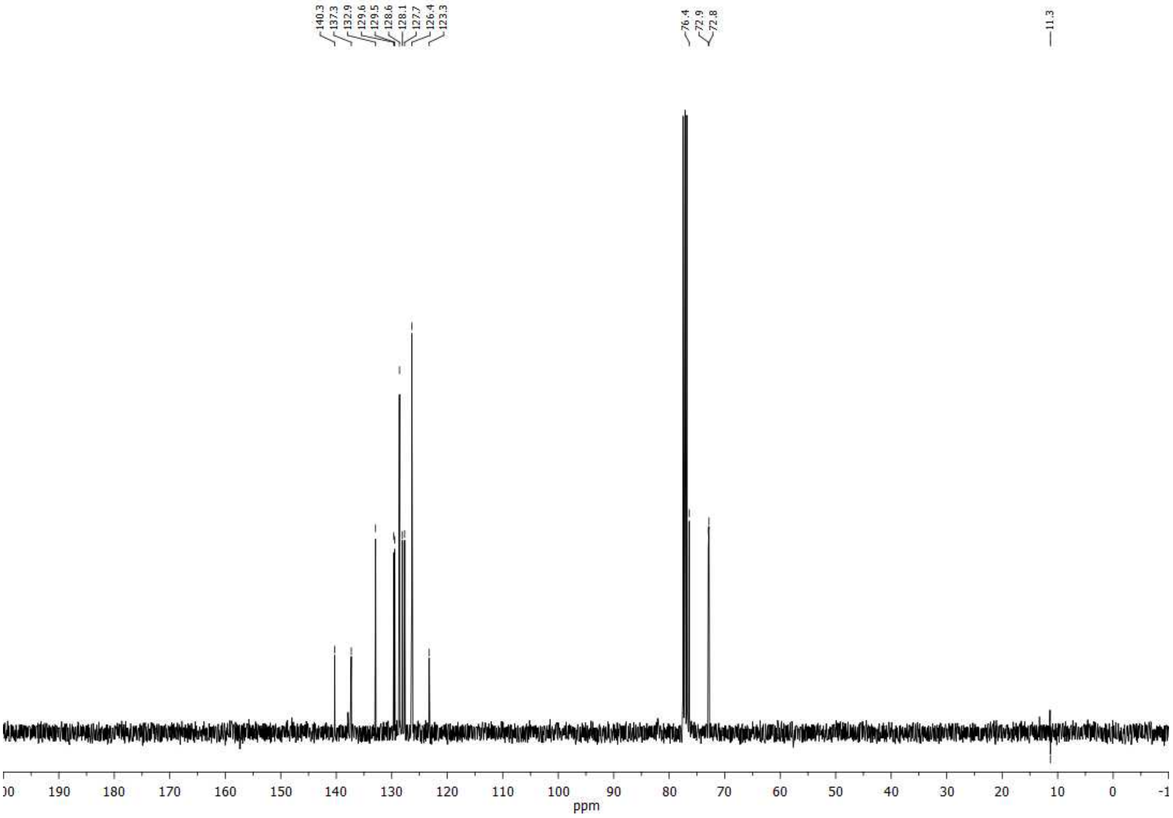

^{13}C NMR (100 MHz, $CDCl_3$)

(*S*)-2-((2-Hydroxy-2-phenylethoxy)methyl)benzonitrile (**2ag**)

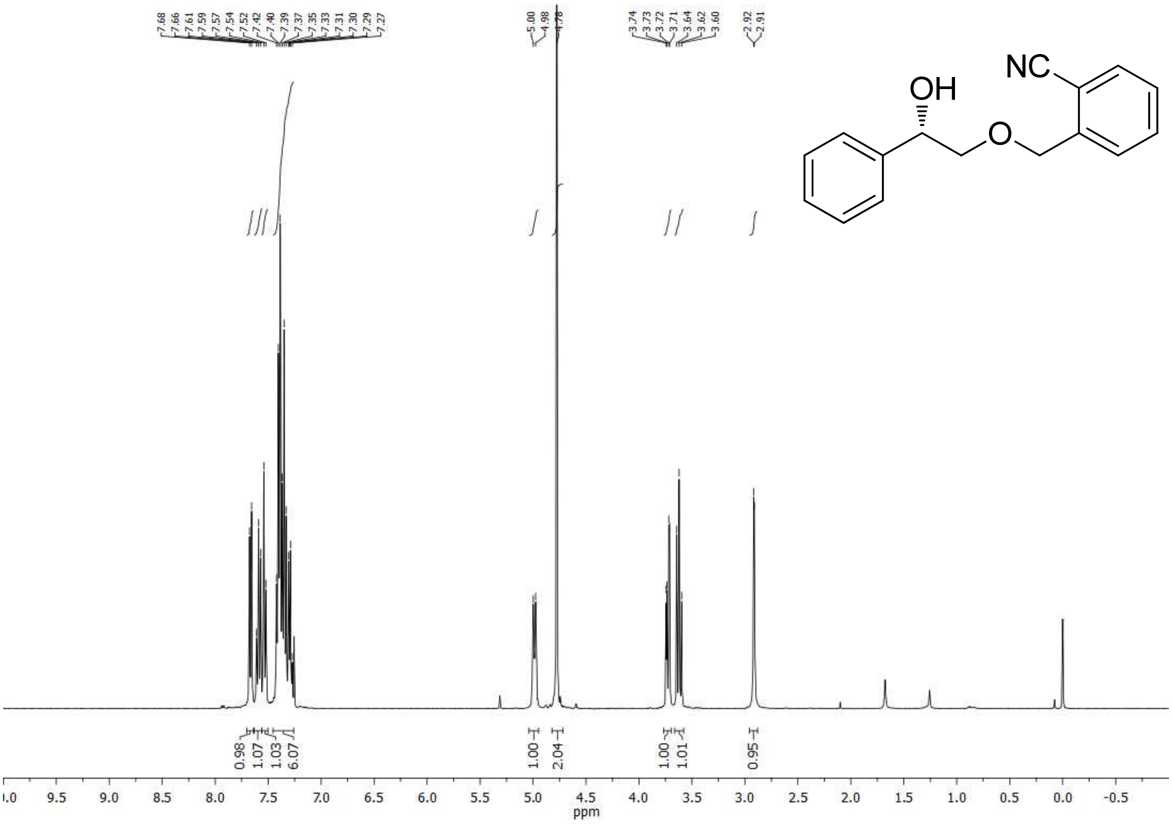

^{1}H NMR (400 MHz, $CDCl_3$)

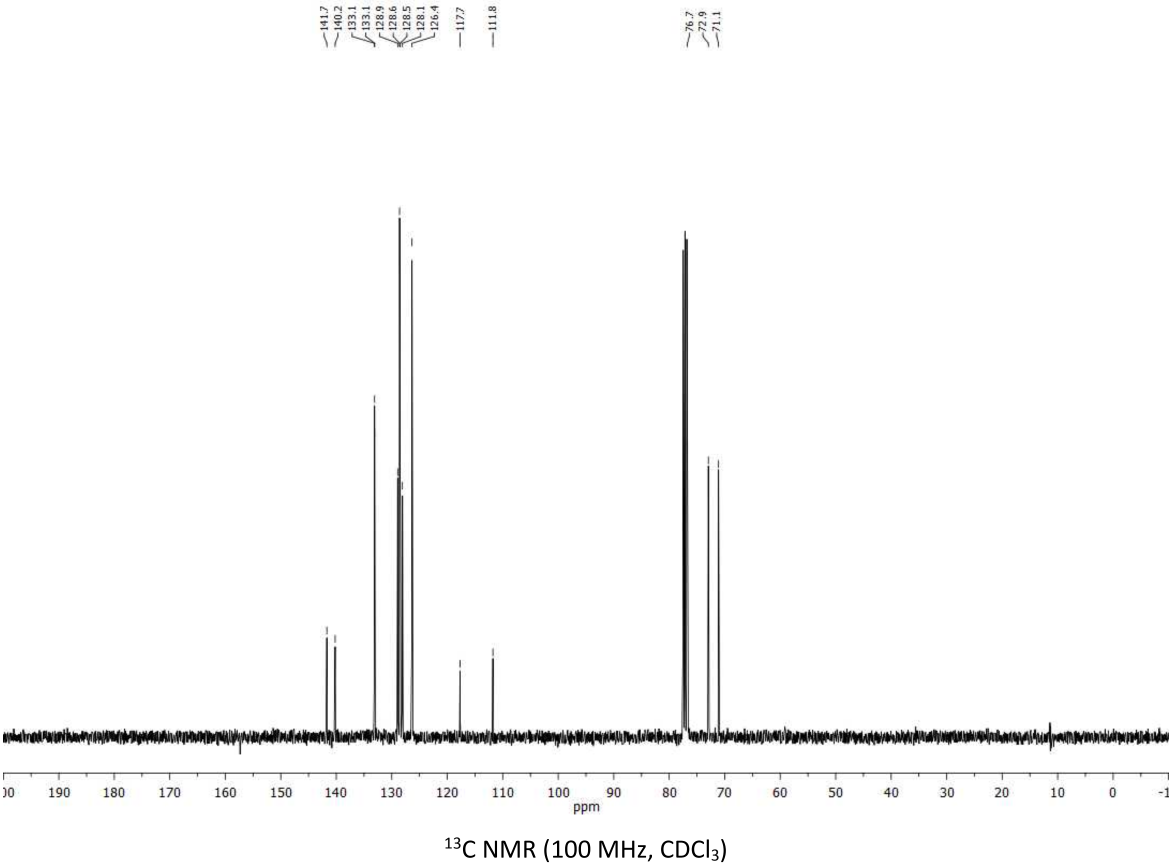

^{13}C NMR (100 MHz, $CDCl_3$)

(*S*)-2-([1,1'-Biphenyl]-4-ylmethoxy)-1-phenylethanol (**2ah**)

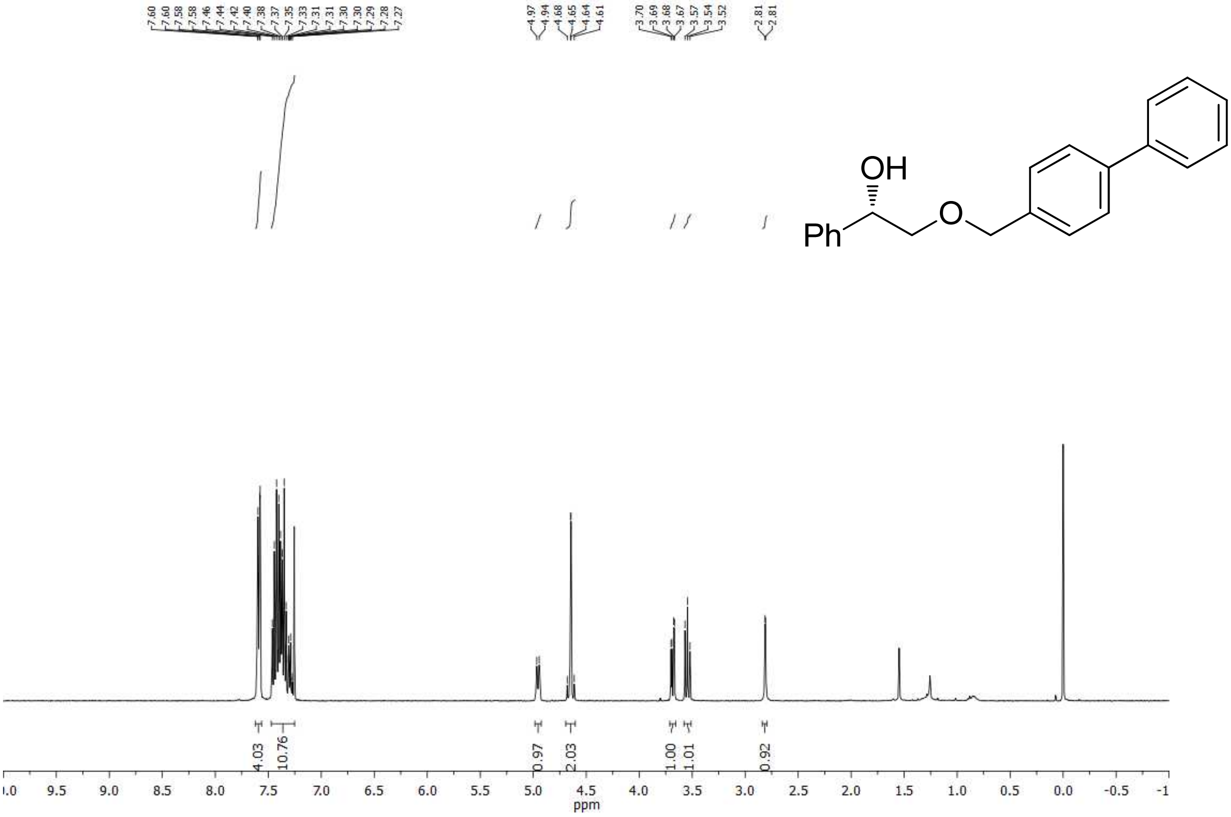

^{1}H NMR (400 MHz, $CDCl_3$)

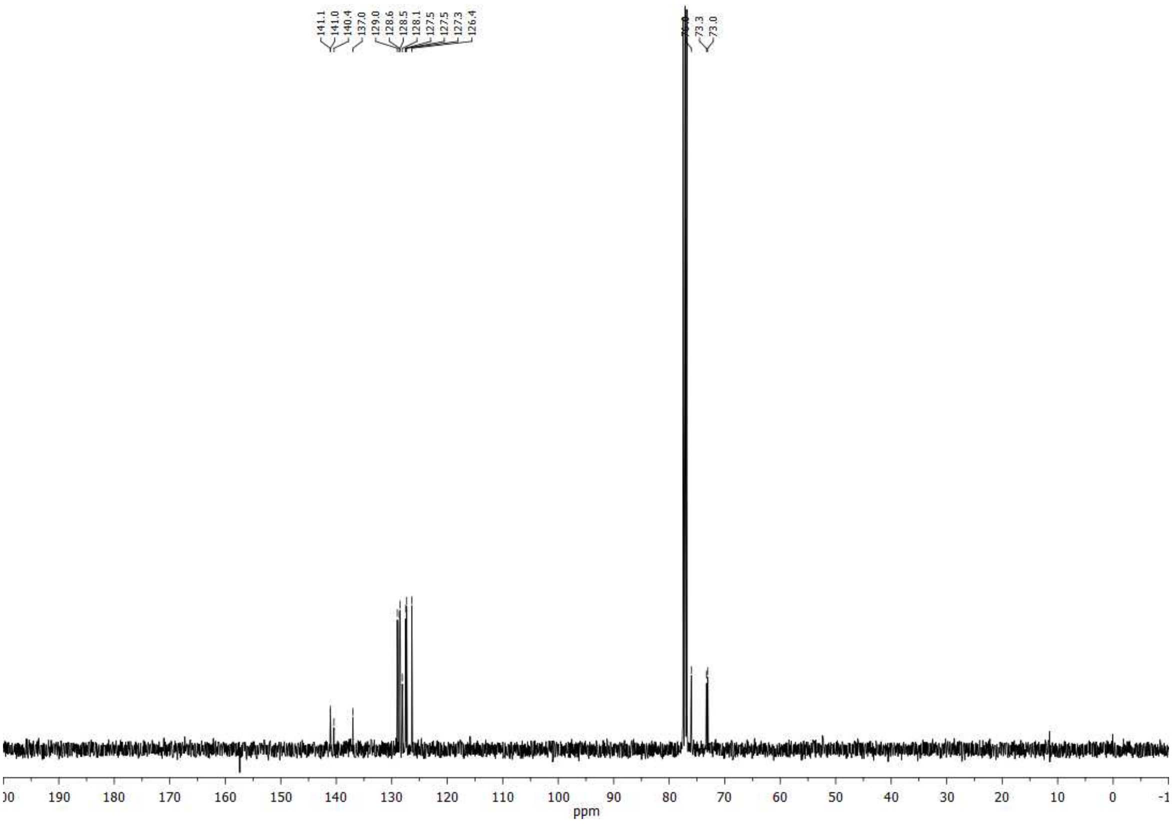

^{13}C NMR (100 MHz, $CDCl_3$)

(*S*)-2-((3,5-Bis(trifluoromethyl)benzyl)oxy)-1-phenylethanol (**2ai**)

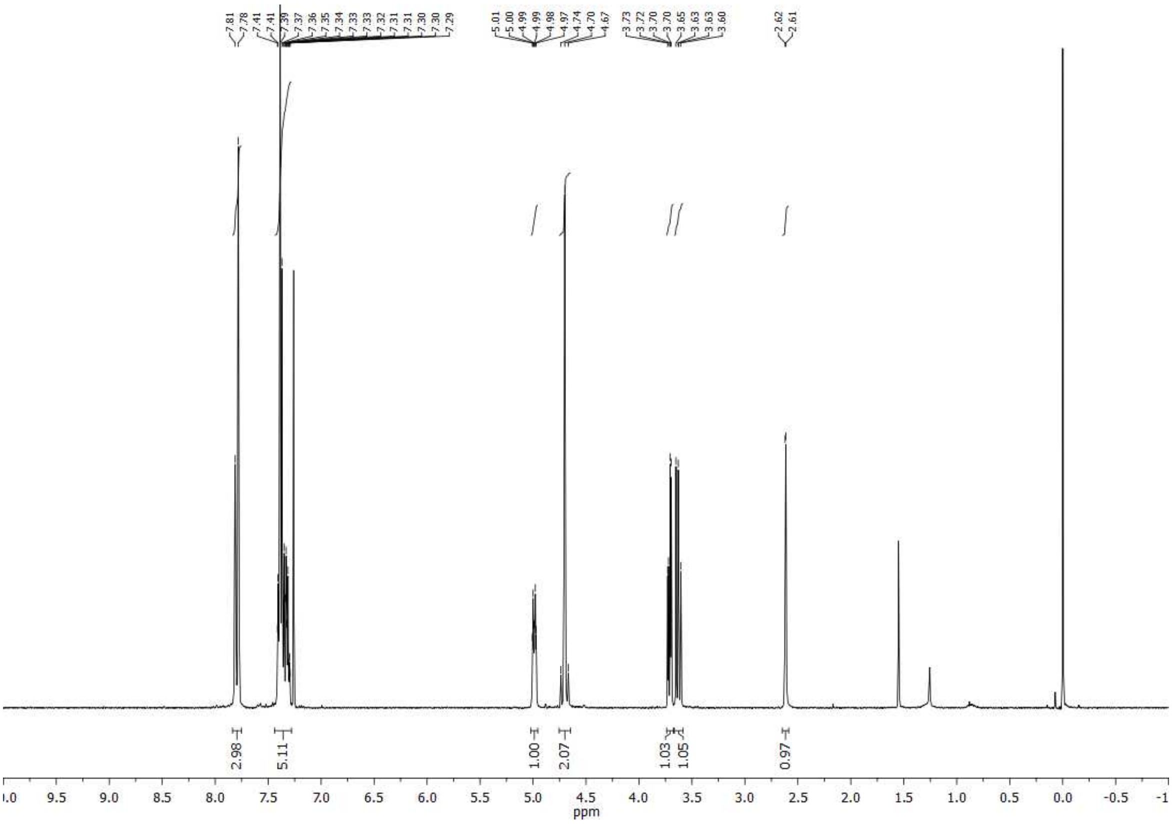

^{1}H NMR (400 MHz, $CDCl_3$)

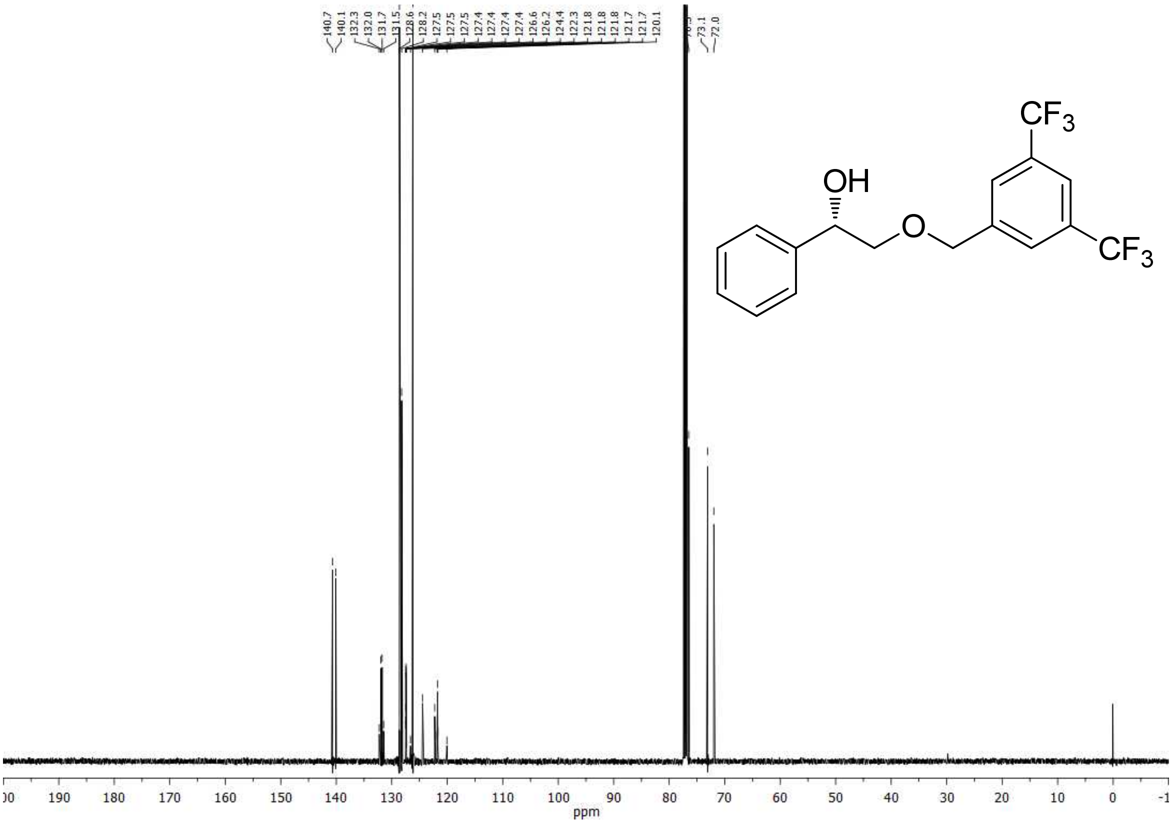

^{13}C NMR (125 MHz, $CDCl_3$)

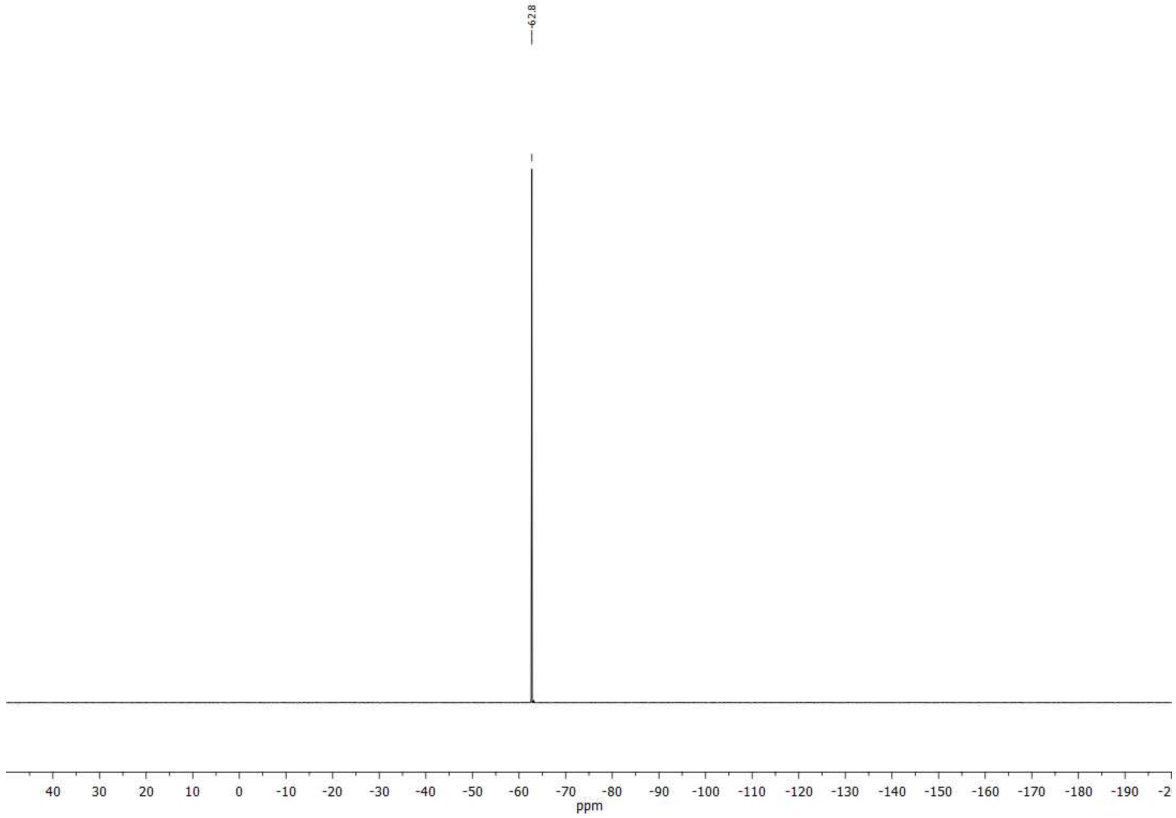

^{19}F NMR (466 MHz, $CDCl_3$)

(*S*)-2-(Naphthalen-2-ylmethoxy)-1-phenylethanol (**2aj**)

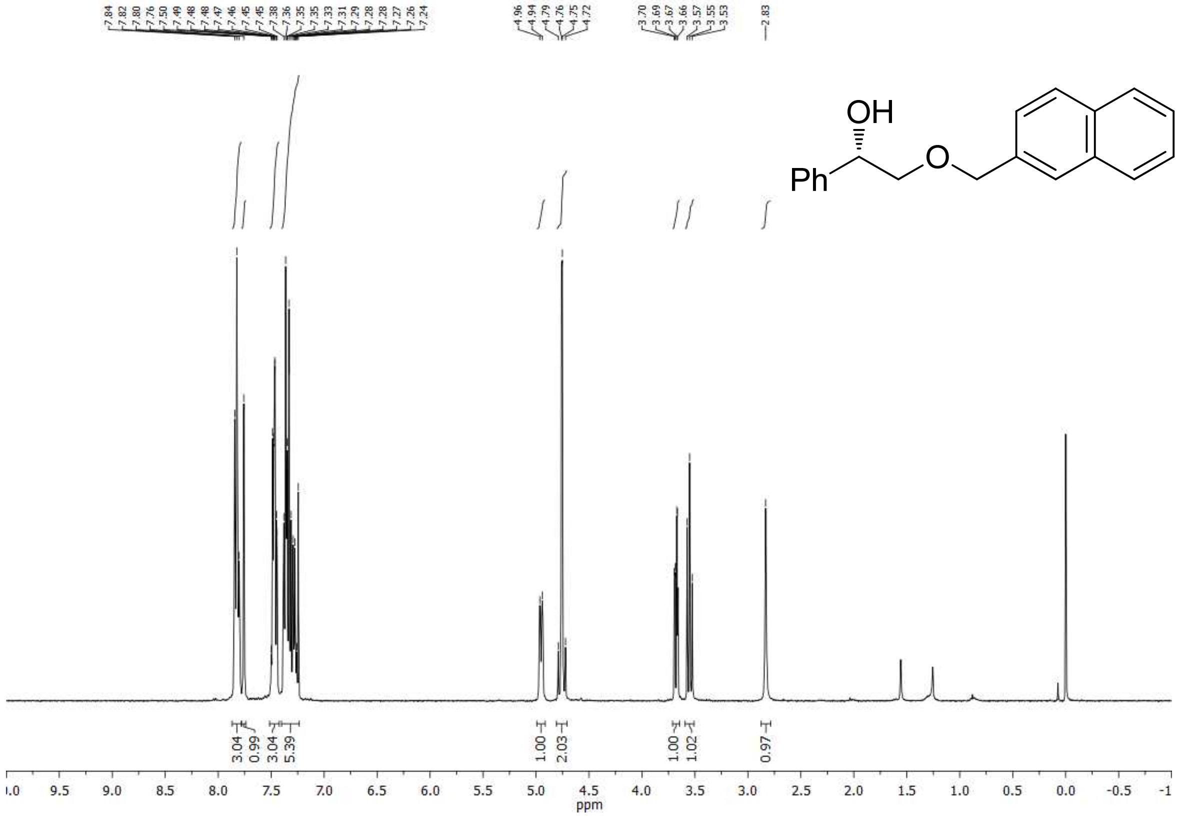

^{1}H NMR (400 MHz, $CDCl_3$)

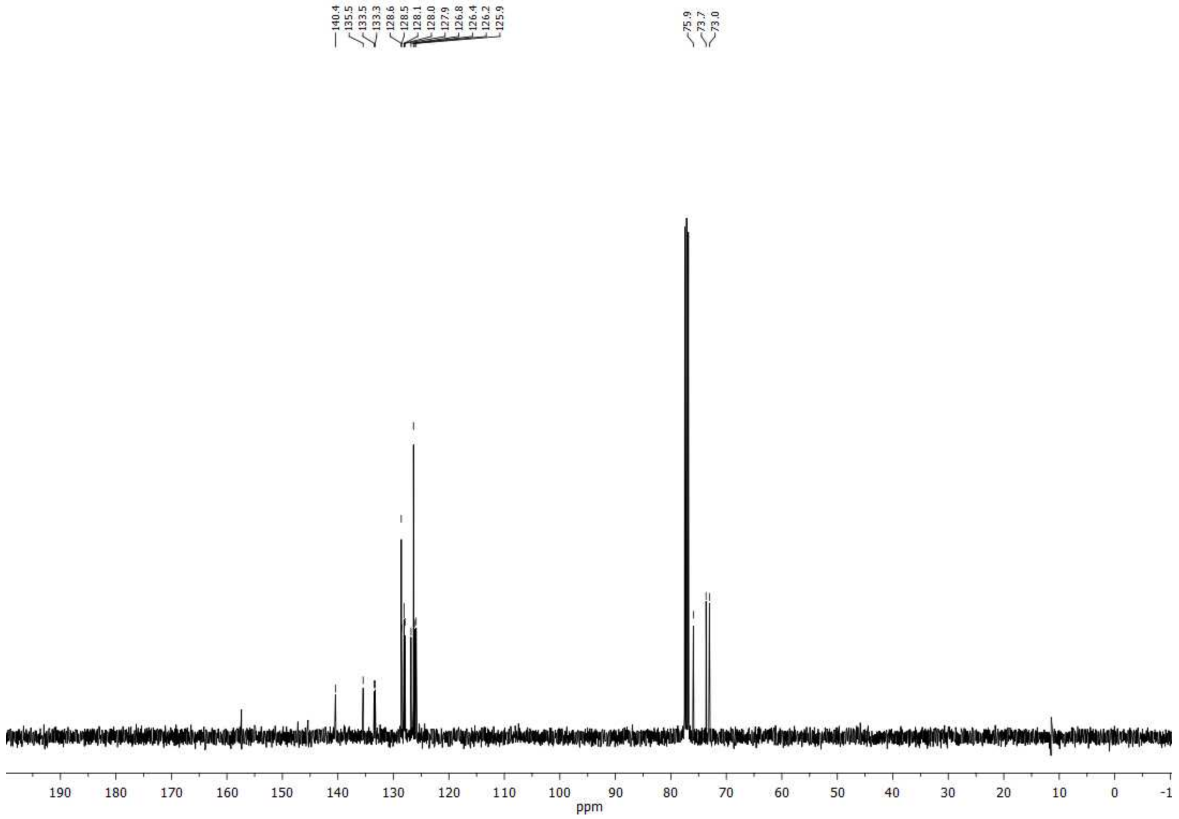

(100 MHz, $CDCl_3$)

(*S*)-2-((3-Methylbut-2-en-1-yl)oxy)-1-phenylethanol (**2ak)**

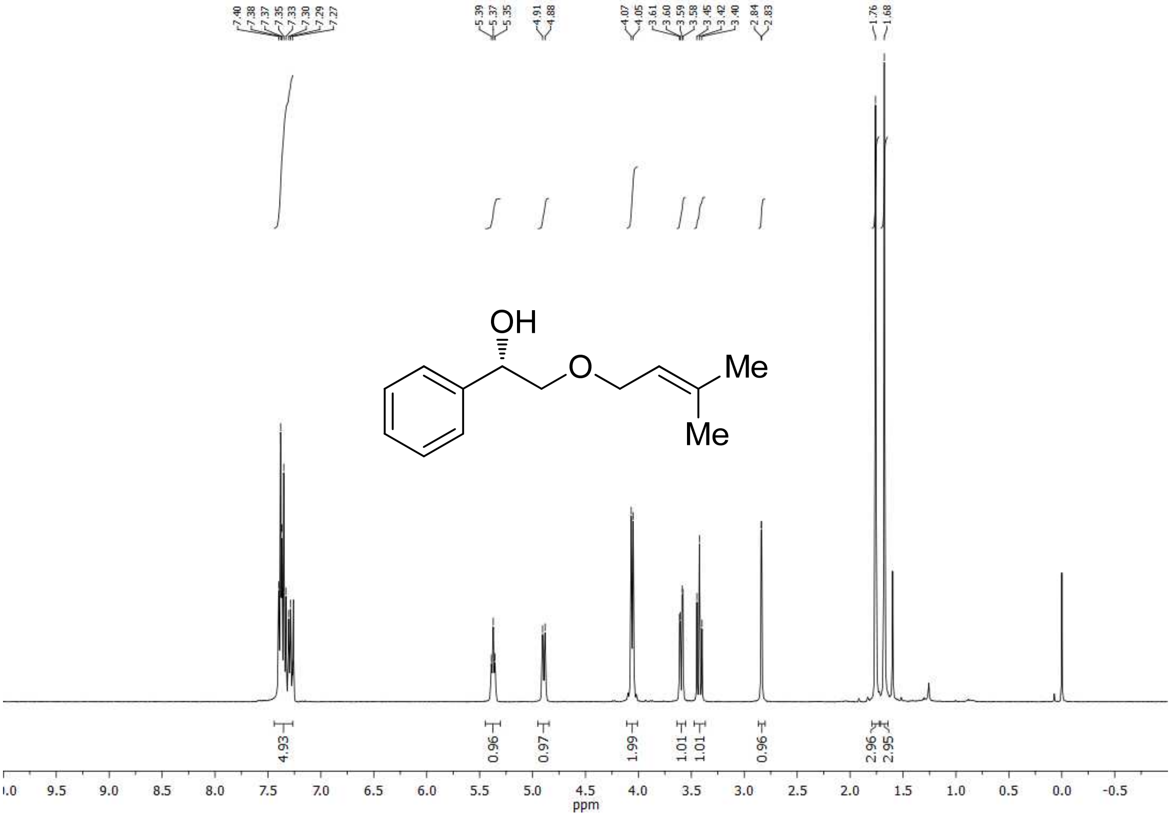

^{1}H NMR (400 MHz, $CDCl_3$)

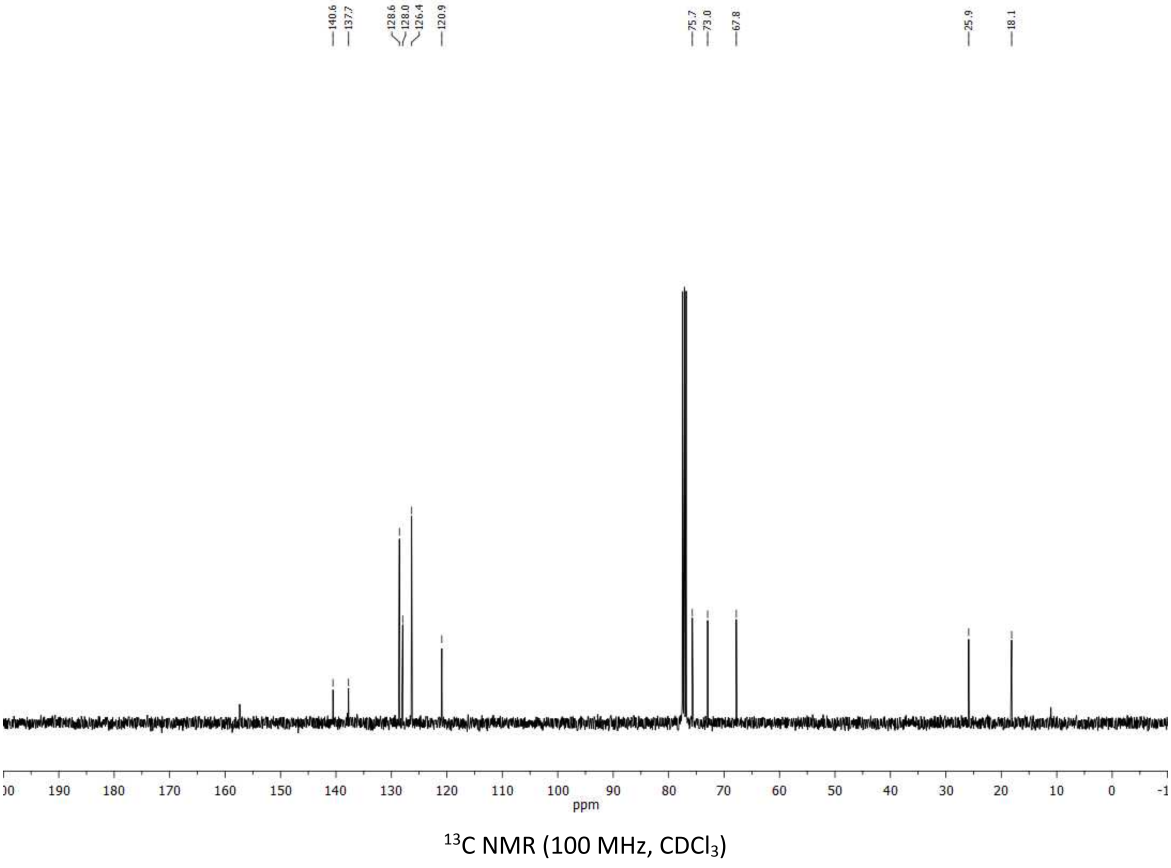

^{13}C NMR (100 MHz, $CDCl_3$)

(*S*)-2-(Cinnamyloxy)-1-phenylethanol (**2al**)

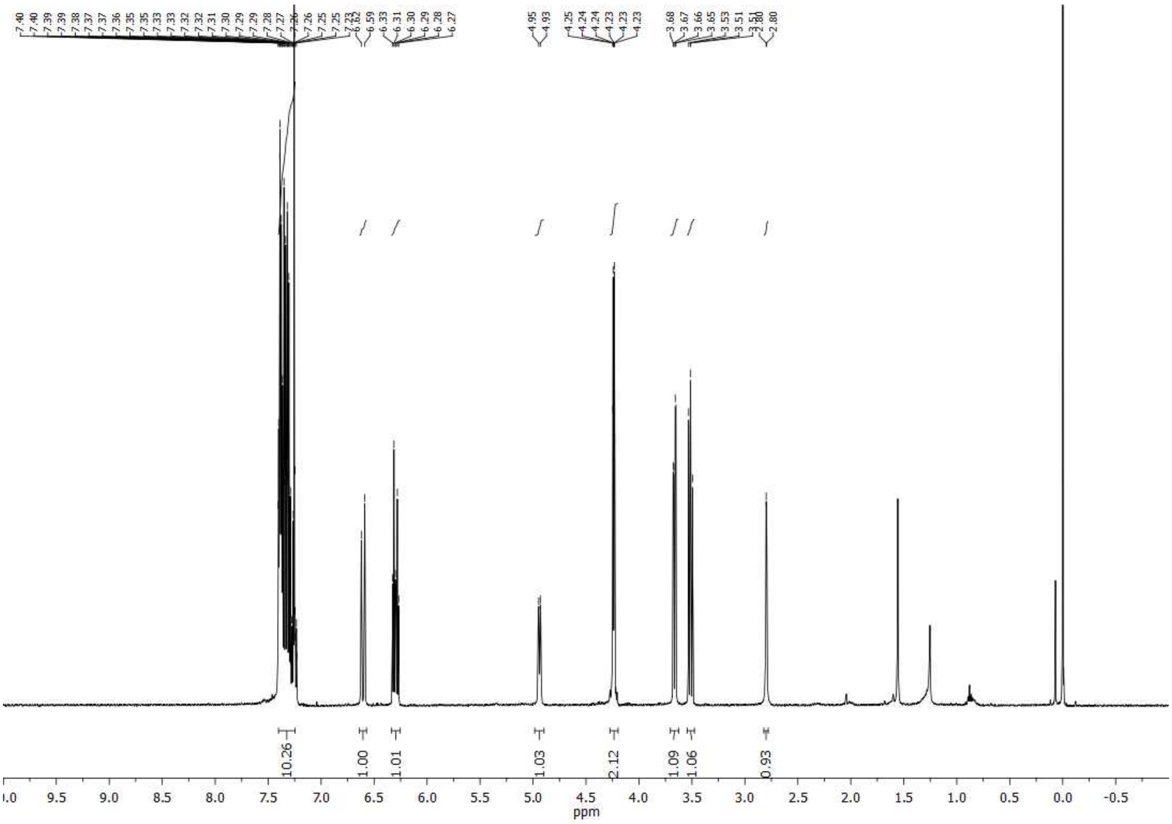

^{1}H NMR (500 MHz, $CDCl_3$)

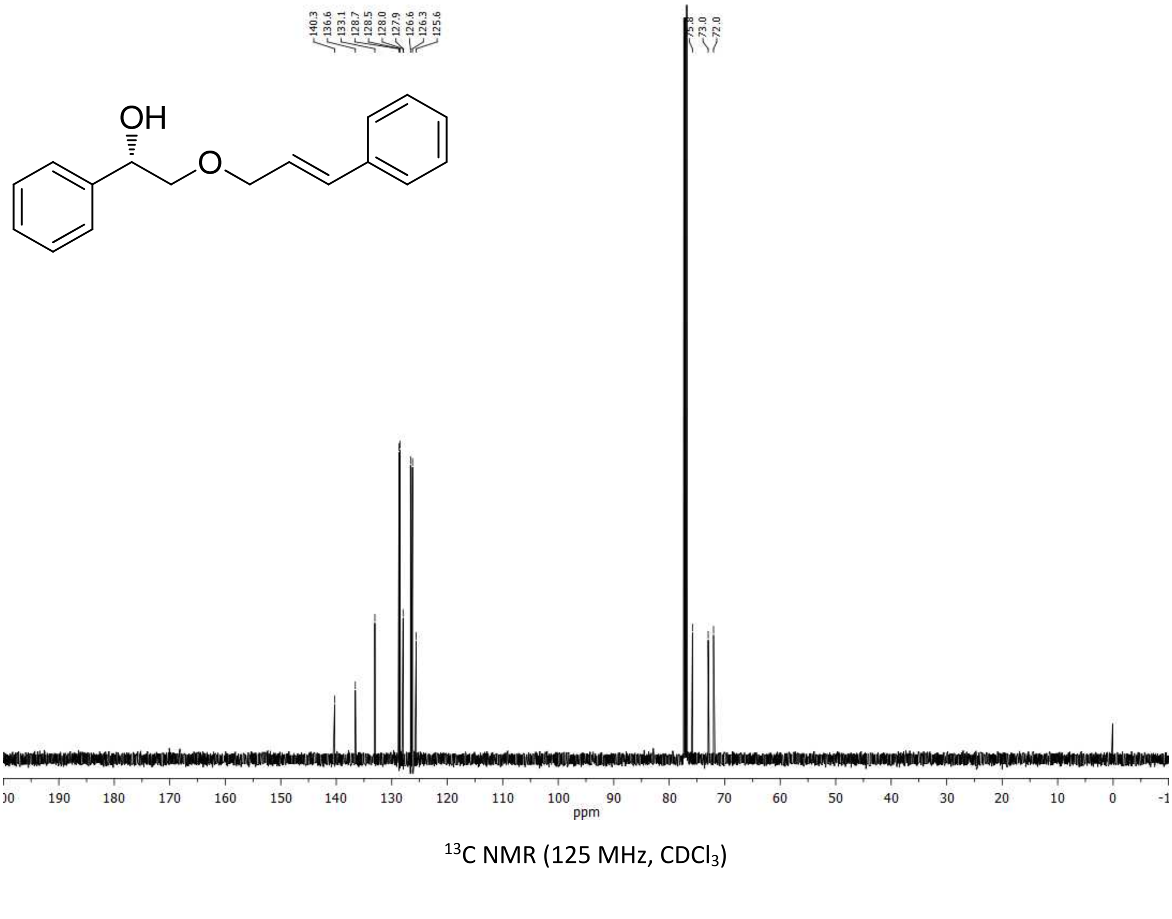

^{13}C NMR (125 MHz, $CDCl_3$)

(*S*,*E*)-Methyl 4-(2-hydroxy-2-phenylethoxy)but-2-enoate (**2am**)

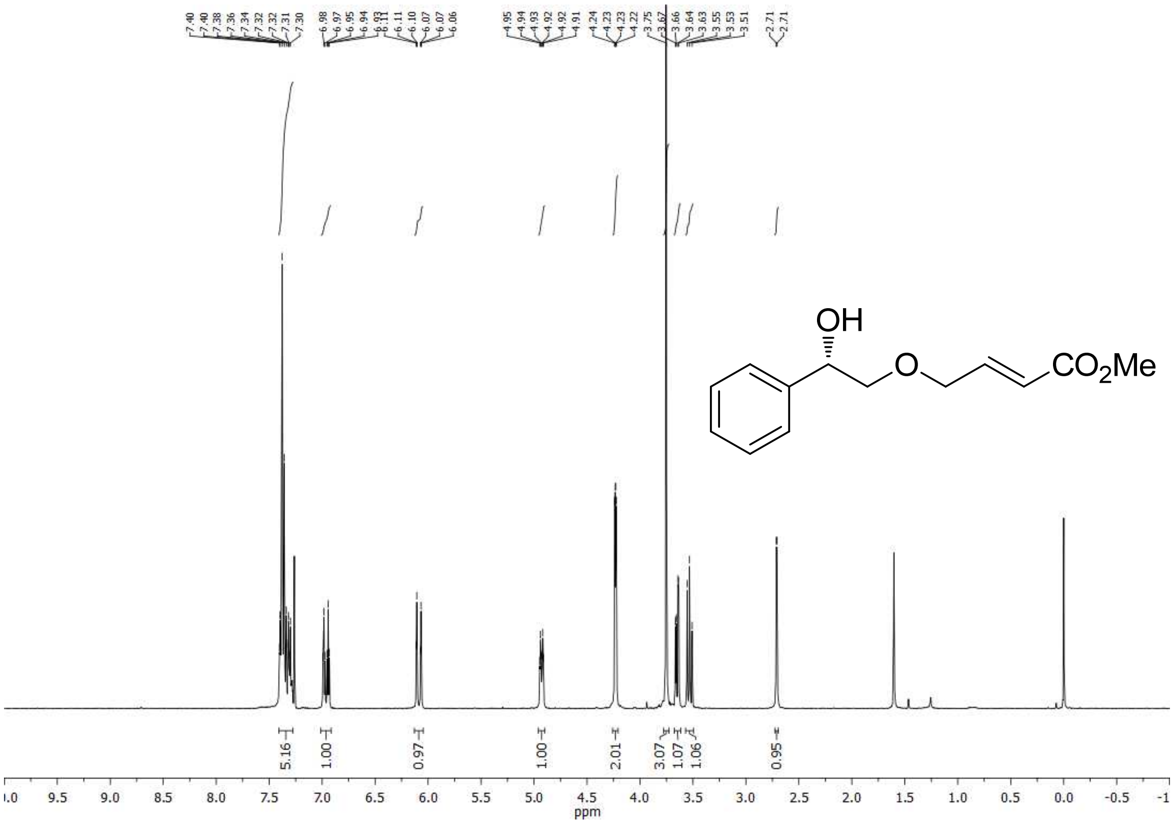

^{1}H NMR (400 MHz, $CDCl_3$)

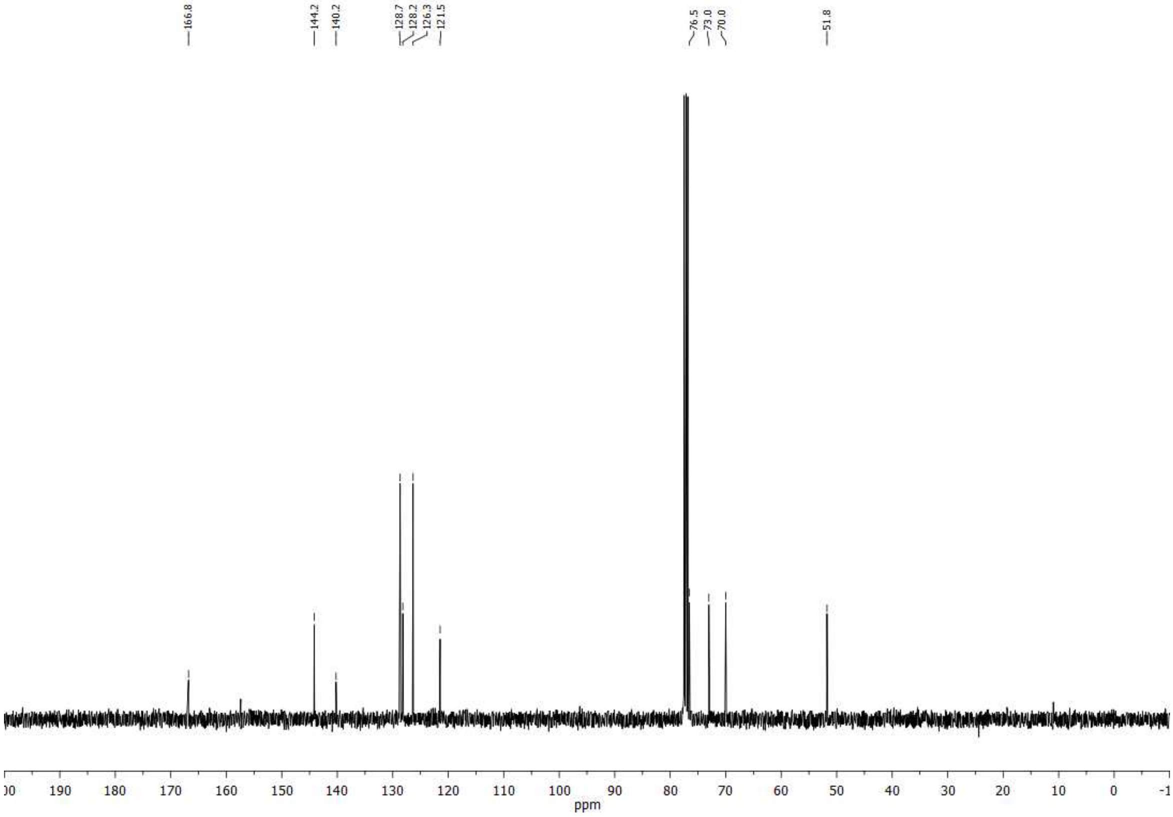

^{13}C NMR (100 MHz, $CDCl_3$)

(*S*)-1-Phenyl-2-((3-phenylprop-2-yn-1-yl)oxy)ethanol (**2an**)

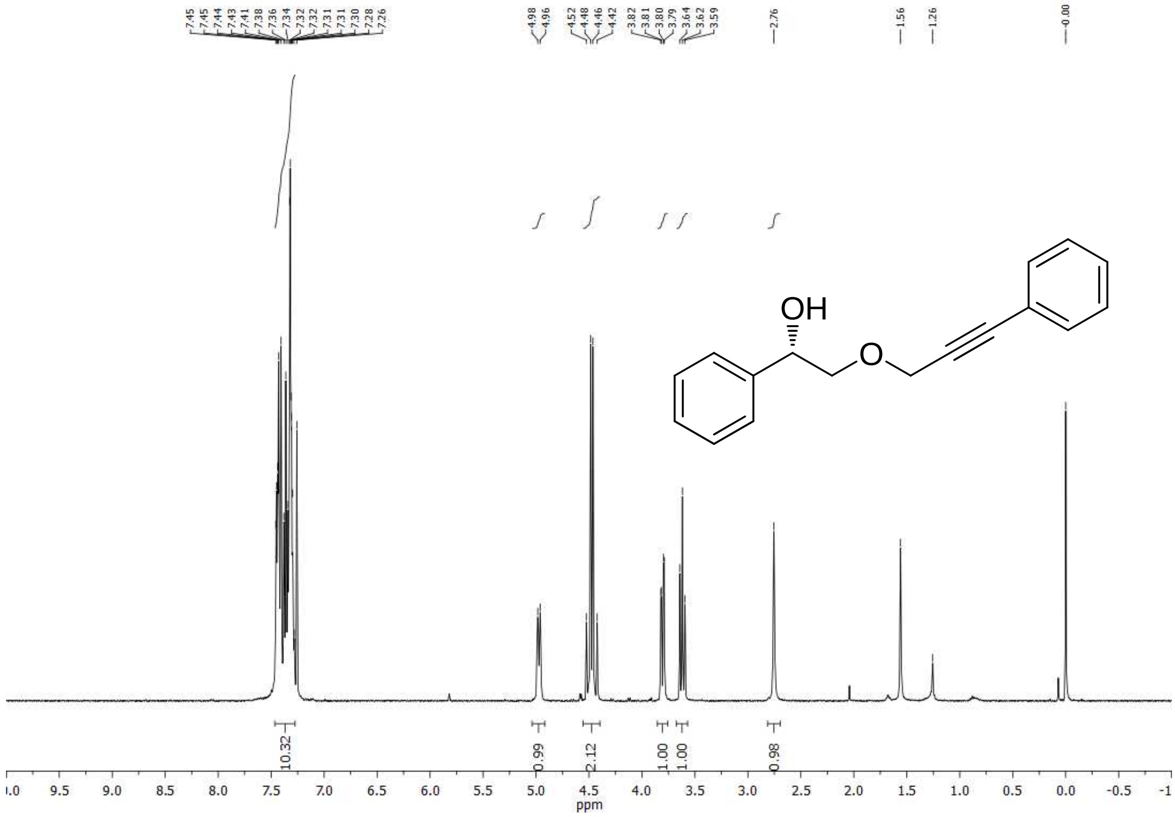

^{1}H NMR (400 MHz, $CDCl_3$)

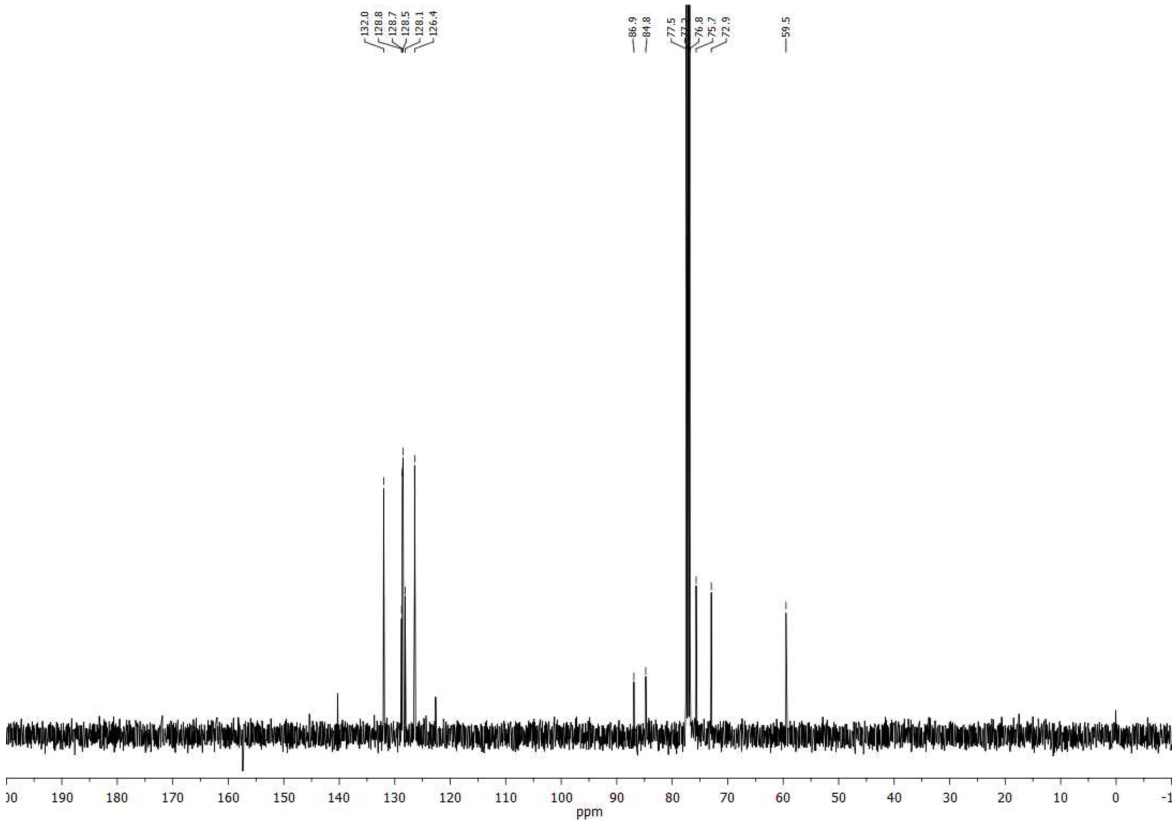

^{13}C NMR (100 MHz, $CDCl_3$)

(*S*)-2-(benzyloxy)-1-(*o*-tolyl)ethanol (**2ba**)

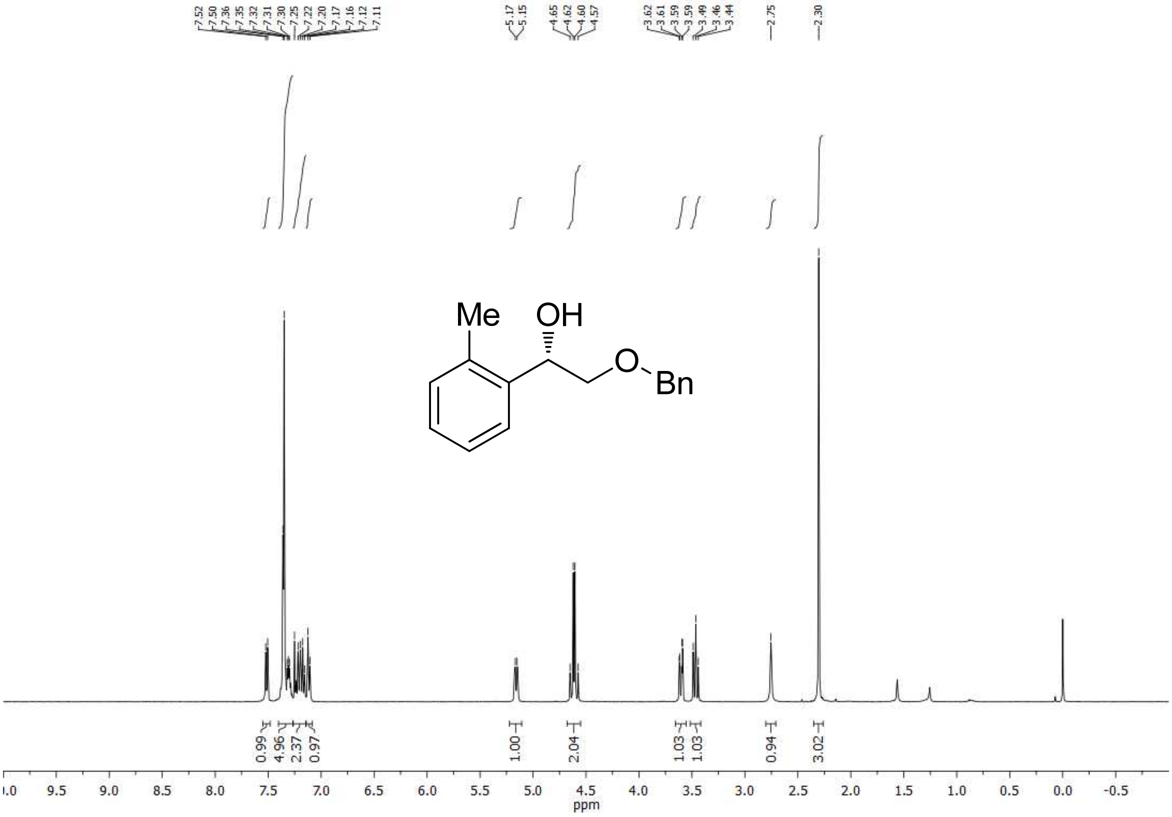

^{1}H NMR (400 MHz, $CDCl_3$)

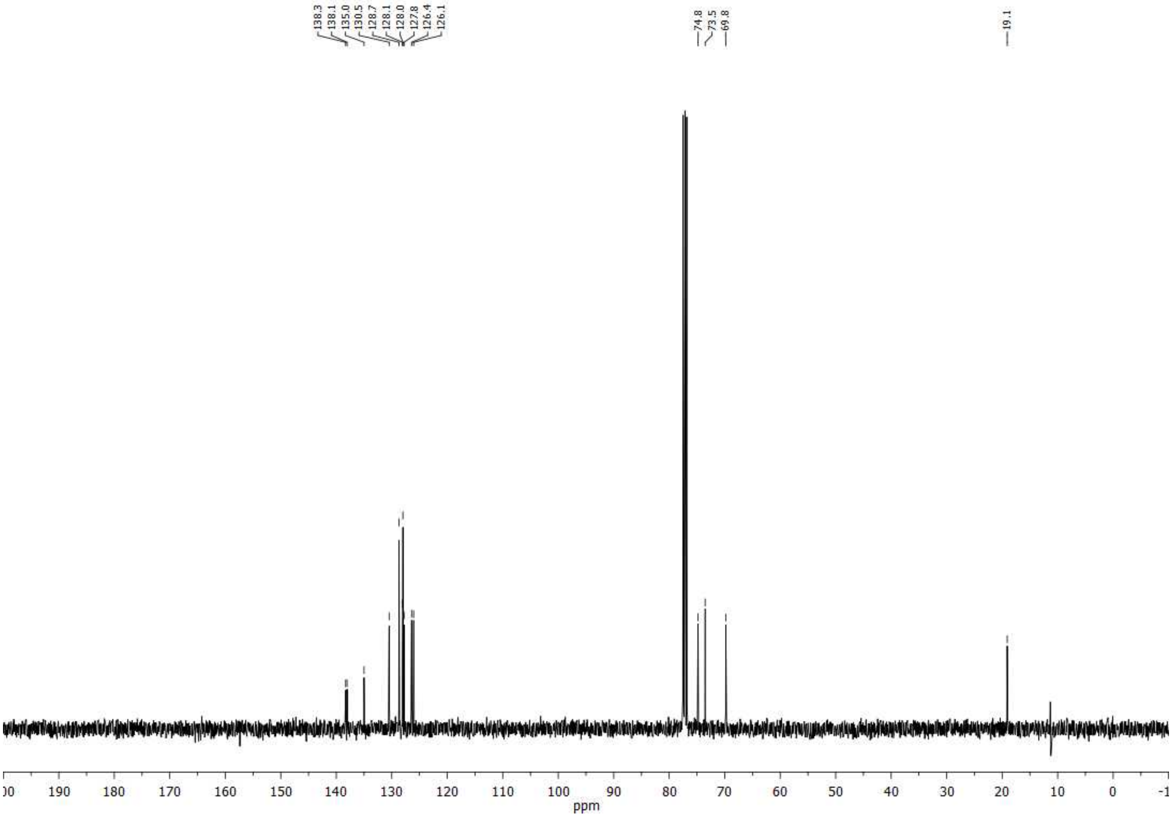

^{13}C NMR (100 MHz, $CDCl_3$)

(*S*)-2-(Benzyloxy)-1-(*m*-tolyl)ethanol (**2ca**)

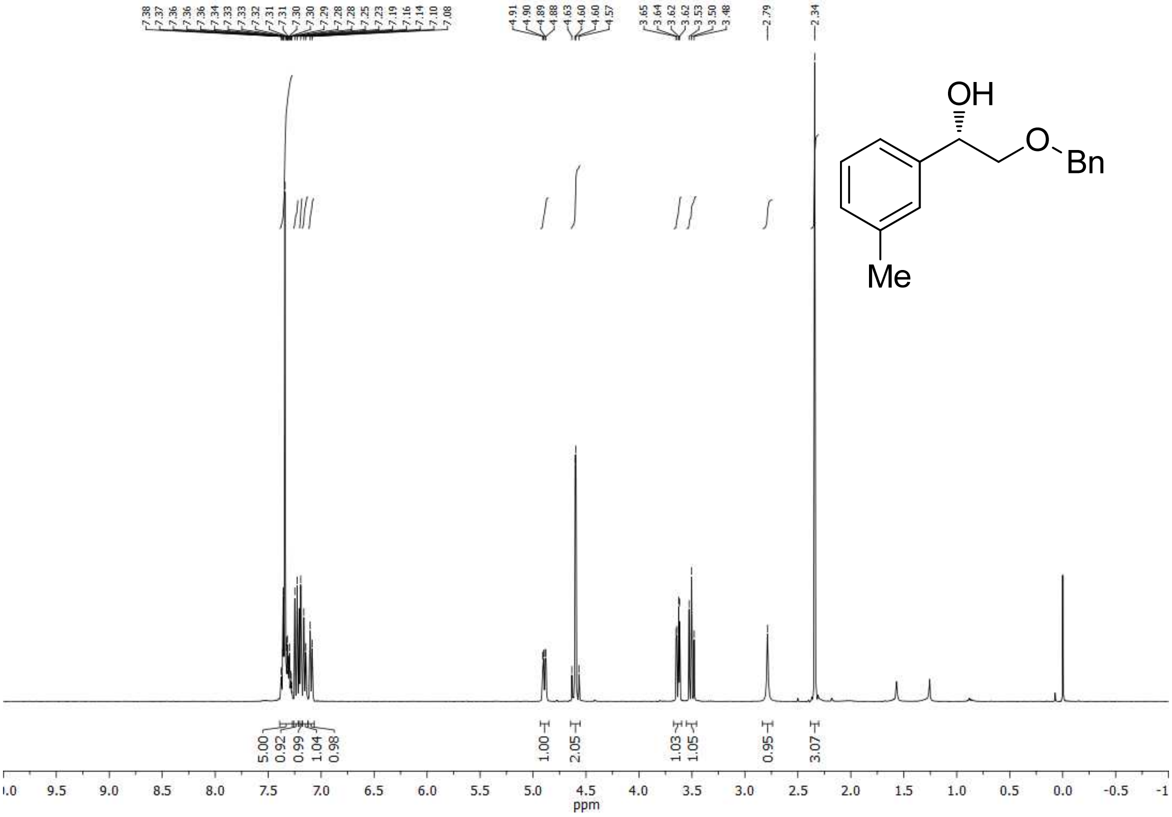

^{1}H NMR (400 MHz, $CDCl_3$)

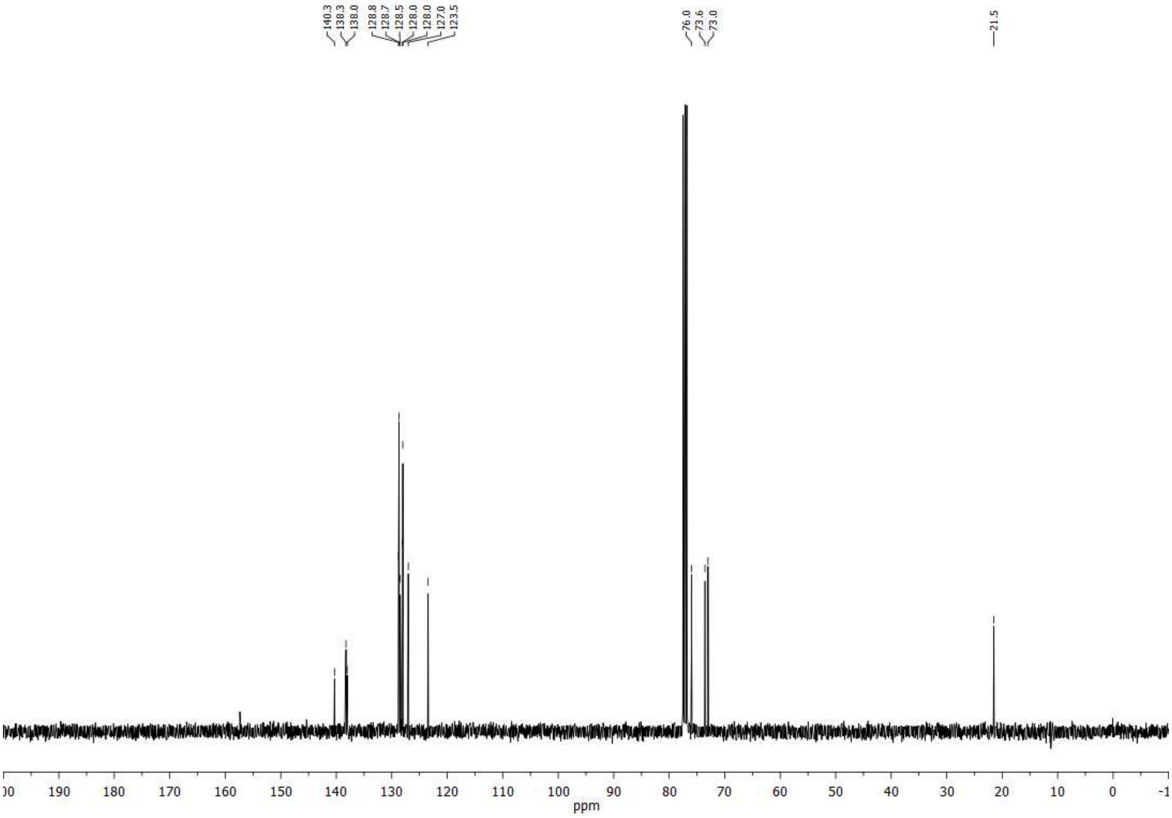

^{13}C NMR (100 MHz, $CDCl_3$)

(*S*)-2-(Benzyloxy)-1-(*p*-tolyl)ethanol (**2da**)

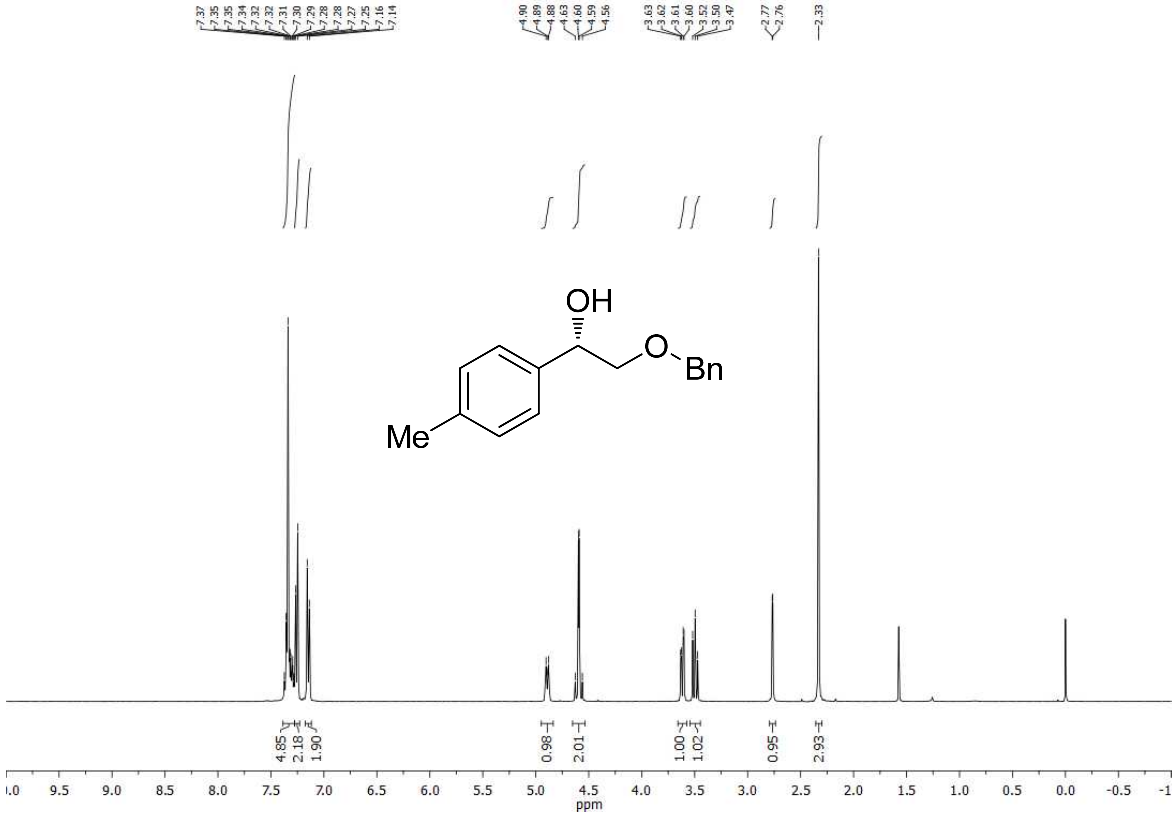

^{1}H NMR (400 MHz, CDCl$_3$)

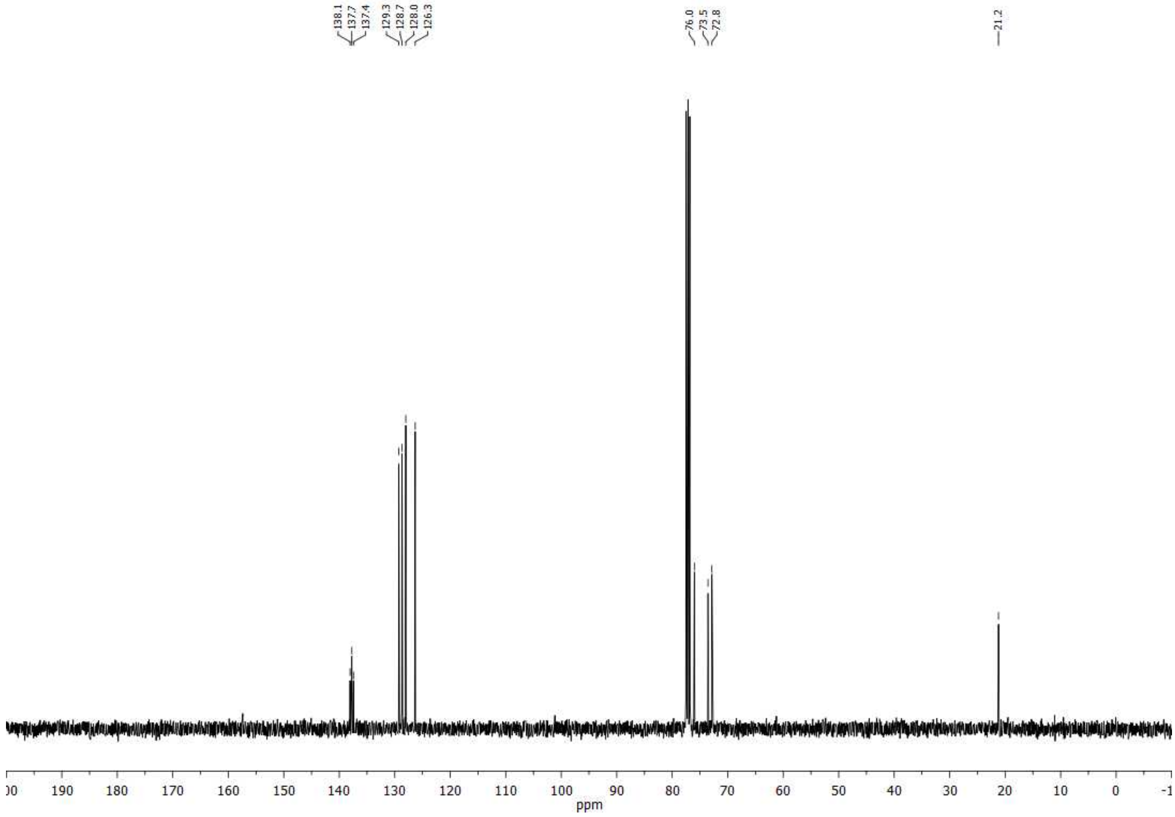

^{13}C NMR (100 MHz, CDCl$_3$)

(*S*)-2-(Benzyloxy)-1-(2-fluorophenyl)ethanol (**2ea**)

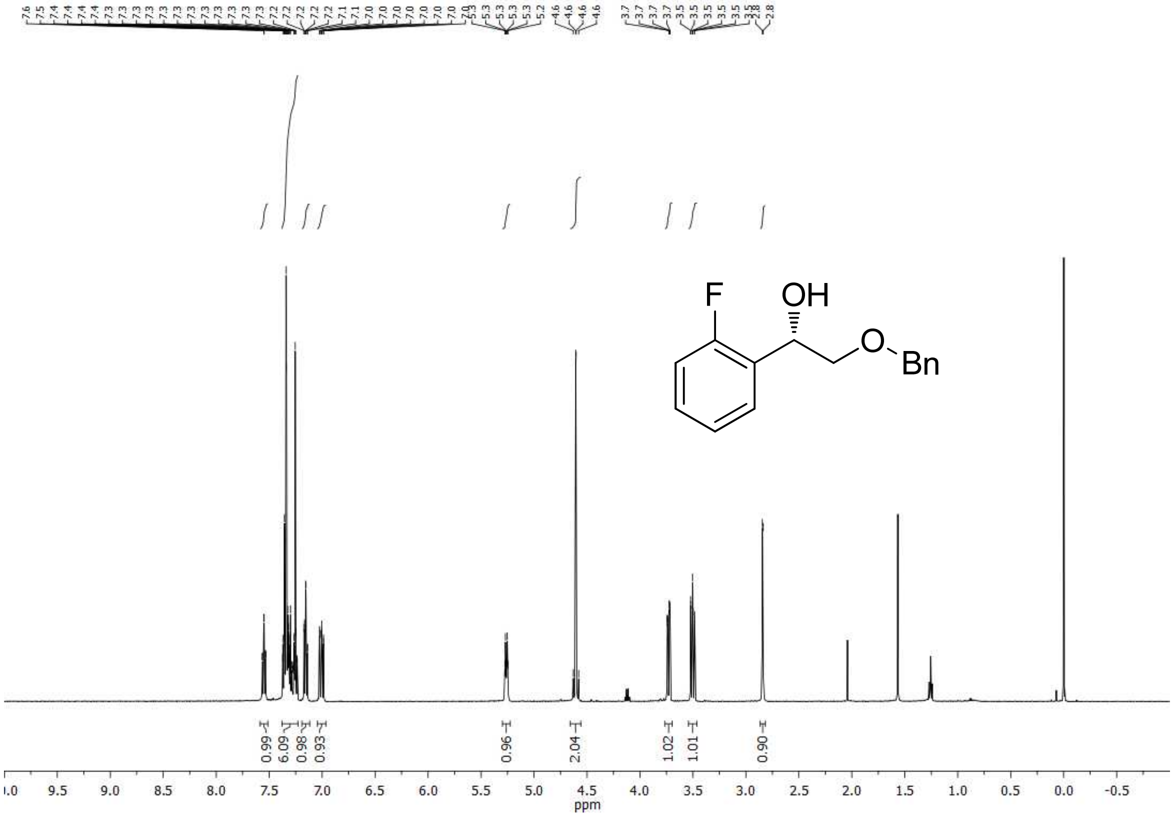

^{1}H NMR (500 MHz, CDCl$_3$)

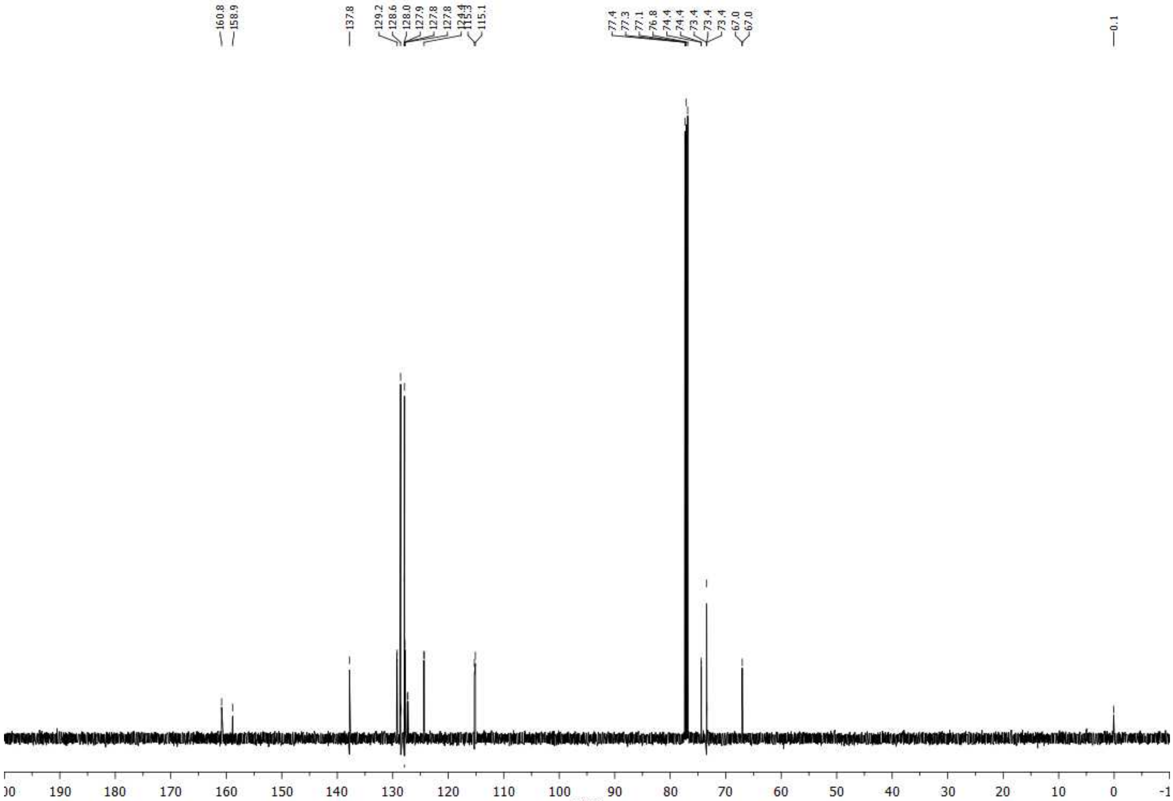

^{13}C NMR (125 MHz, CDCl$_3$)

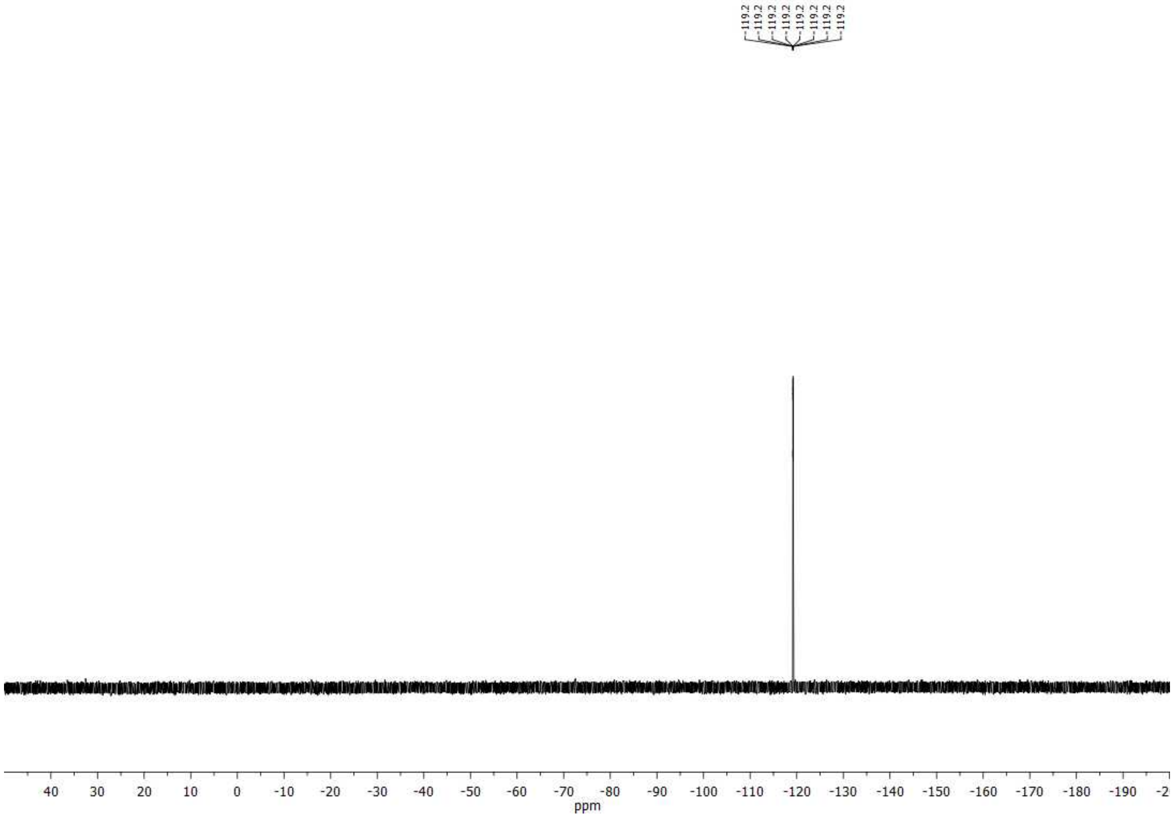

^{19}F NMR (466 MHz, $CDCl_3$)

(*S*)-2-(Benzyloxy)-1-(3-fluorophenyl)ethanol (**2fa**)

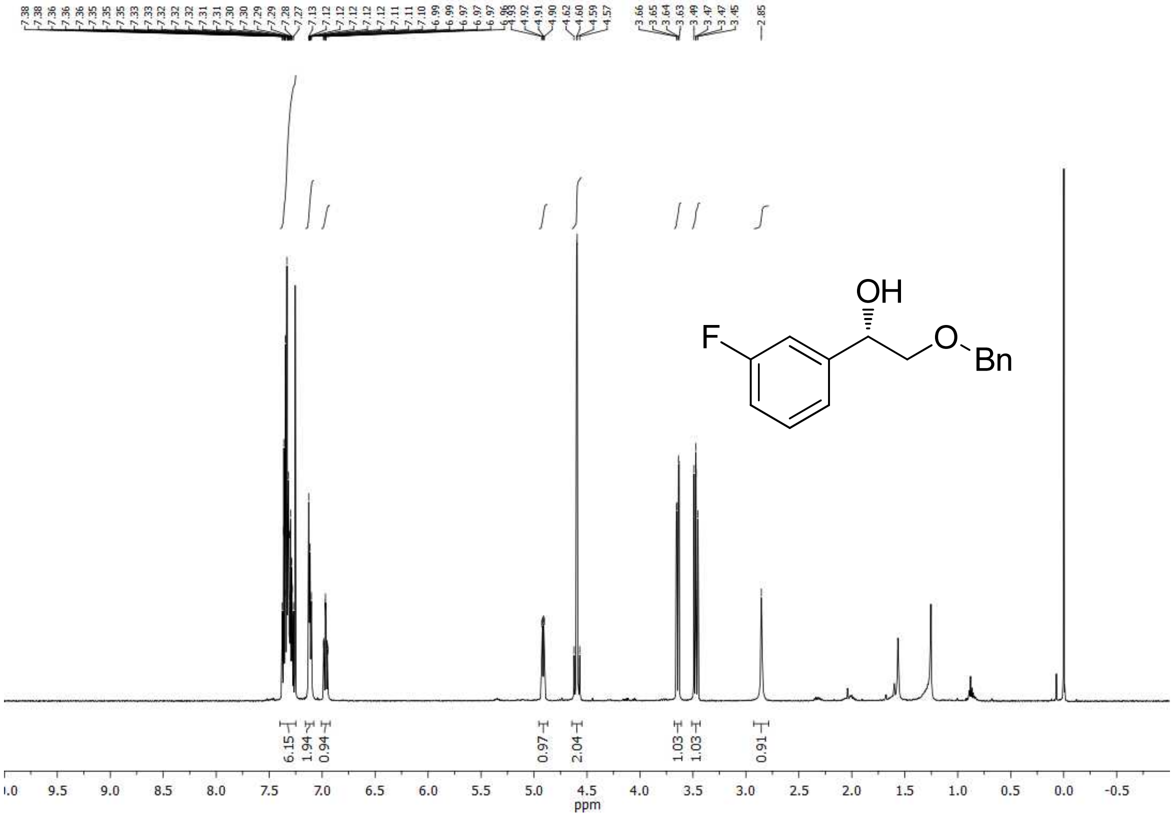

^{1}H NMR (500 MHz, $CDCl_3$)

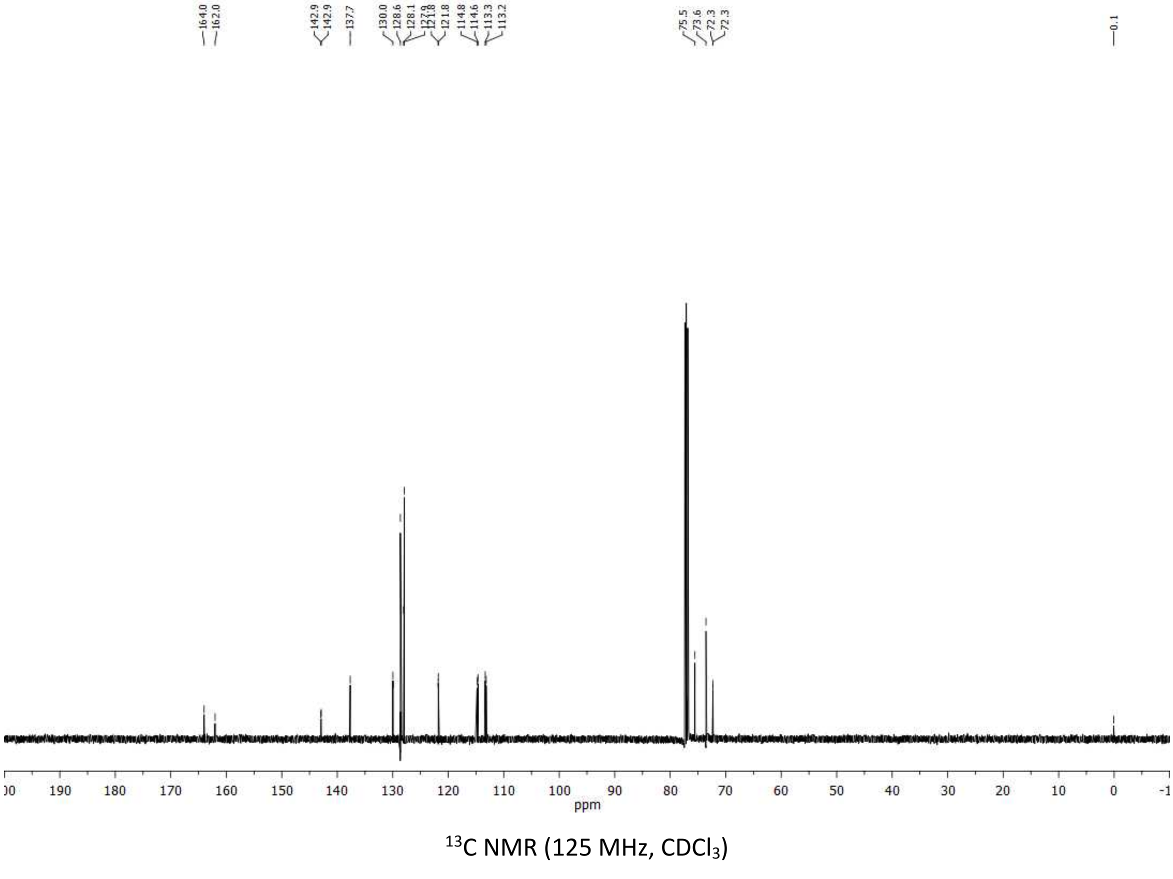

^{13}C NMR (125 MHz, $CDCl_3$)

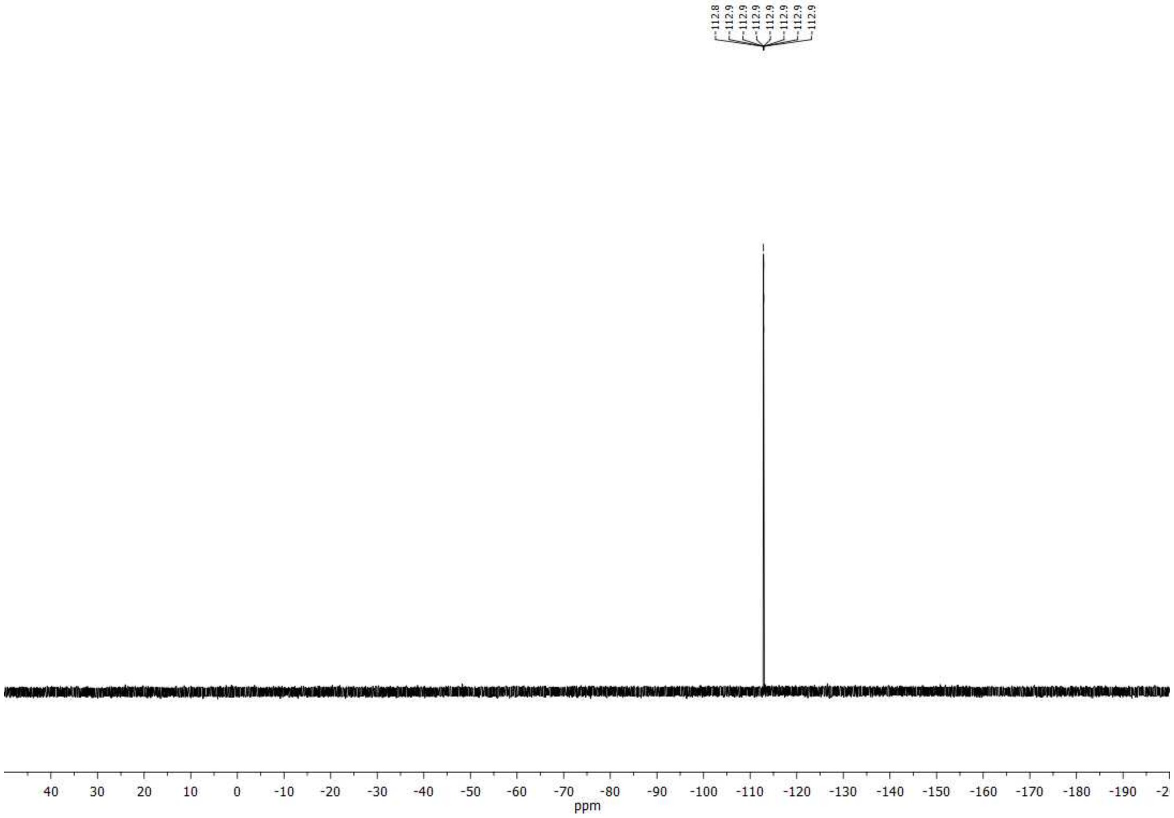

^{19}F NMR (466 MHz, $CDCl_3$)

(*S*)-2-(Benzyloxy)-1-(4-fluorophenyl)ethanol (**2ga**)

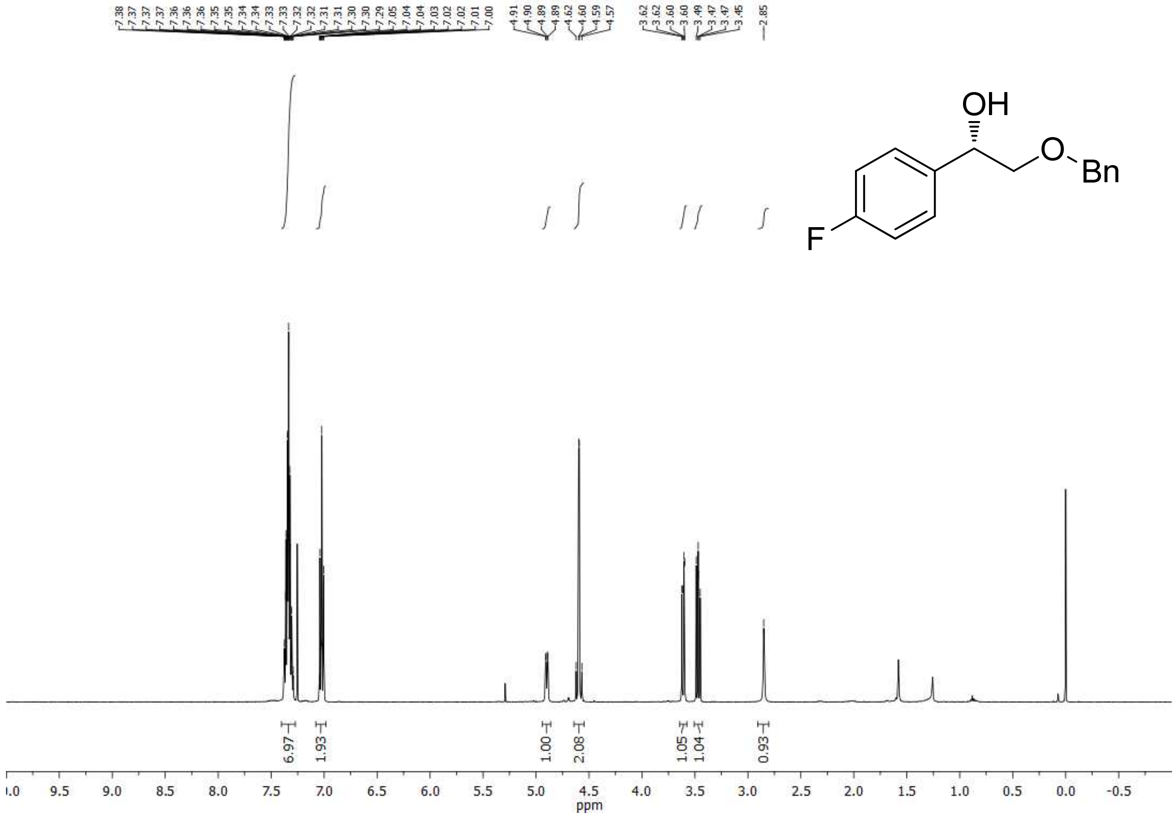

^{1}H NMR (500 MHz, $CDCl_3$)

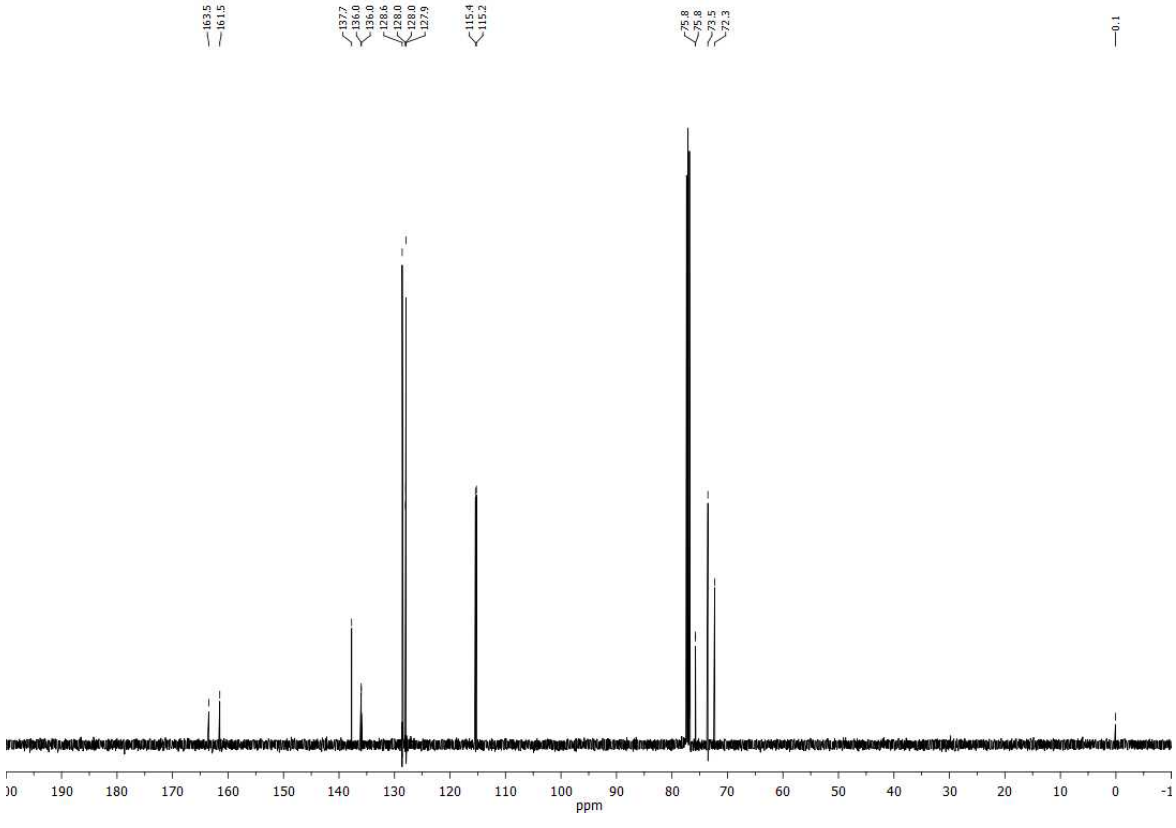

^{13}C NMR (125 MHz, $CDCl_3$)

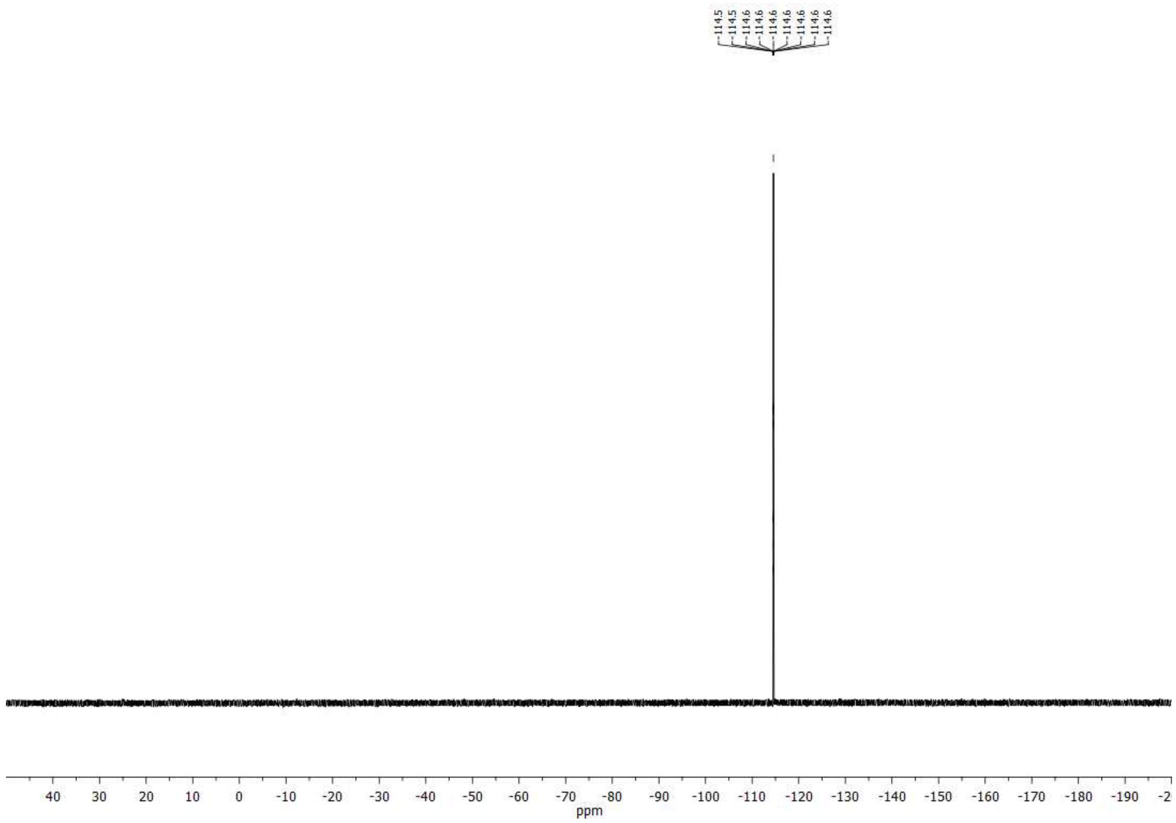

^{19}F NMR (466 MHz, $CDCl_3$)

(*S*)-2-(Benzyloxy)-1-(4-bromophenyl)ethanol (**2ha**)

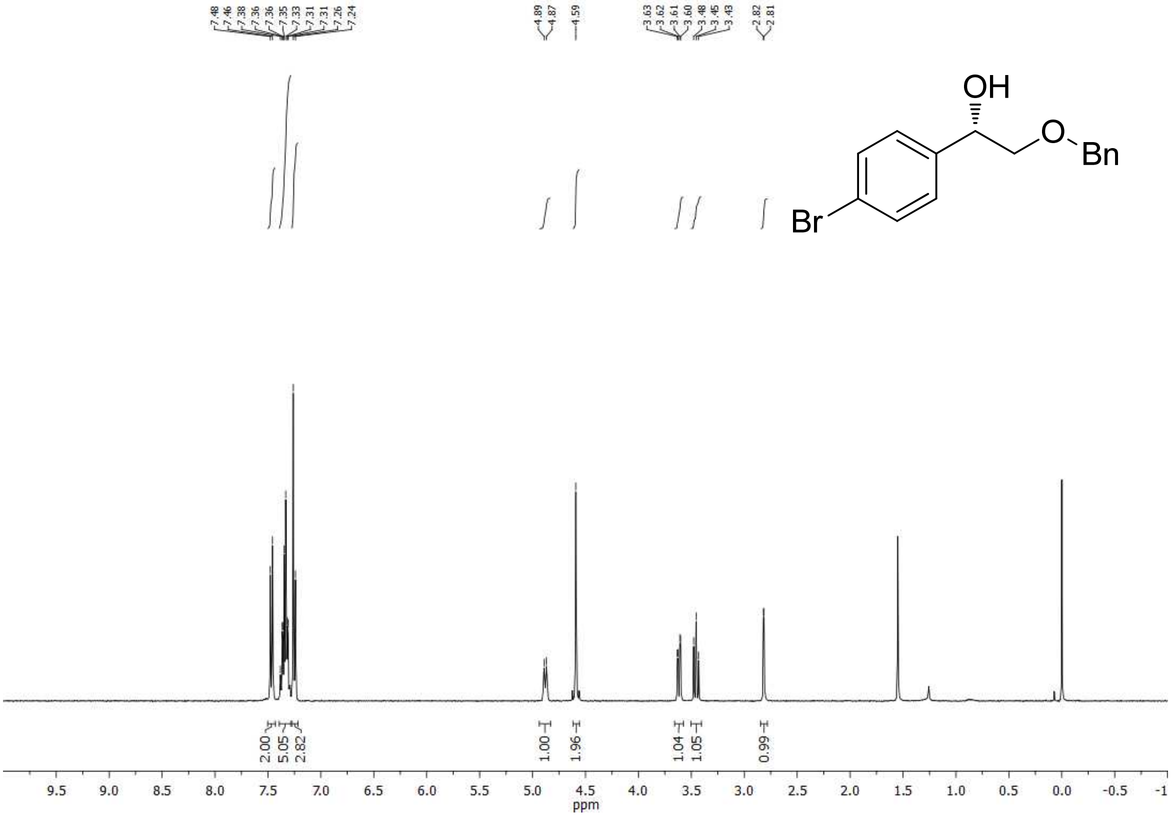

^{1}H NMR (400 MHz, $CDCl_3$)

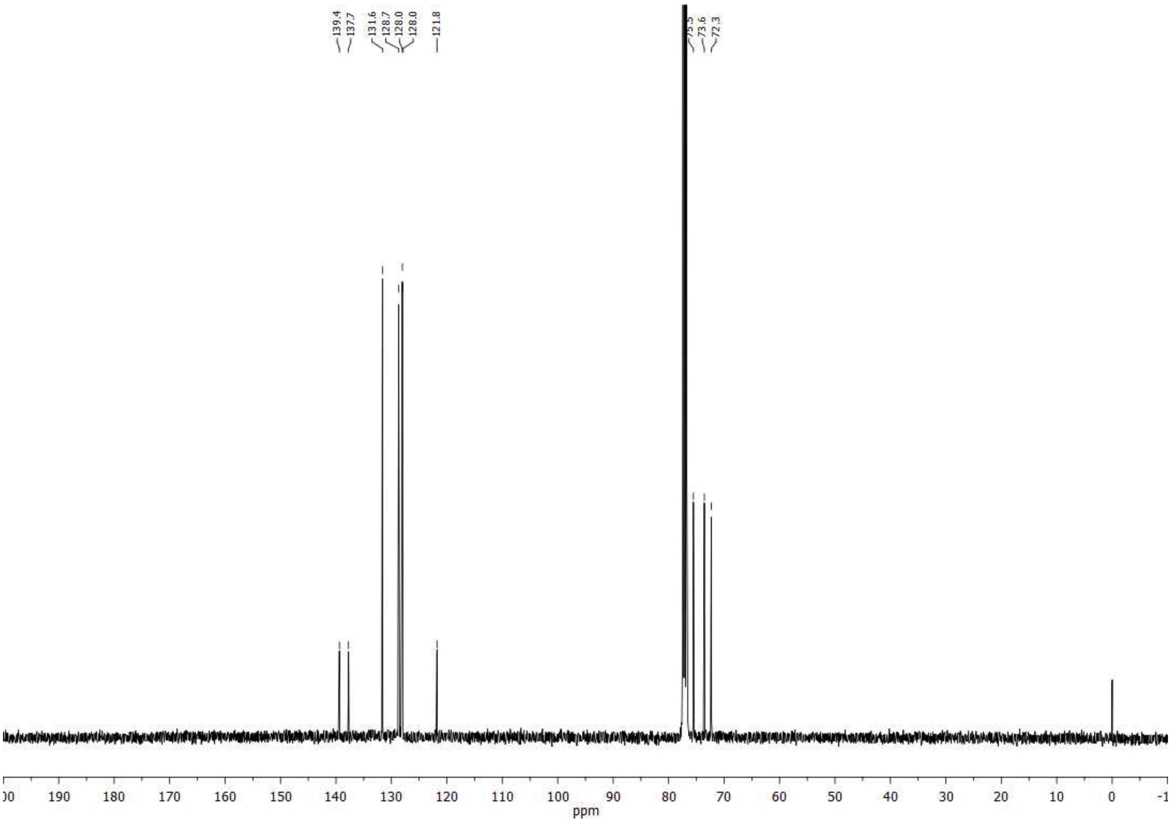

^{13}C NMR (100 MHz, $CDCl_3$)

(*S*)-2-(Benzyloxy)-1-(4-methoxyphenyl)ethanol (**2ia**)

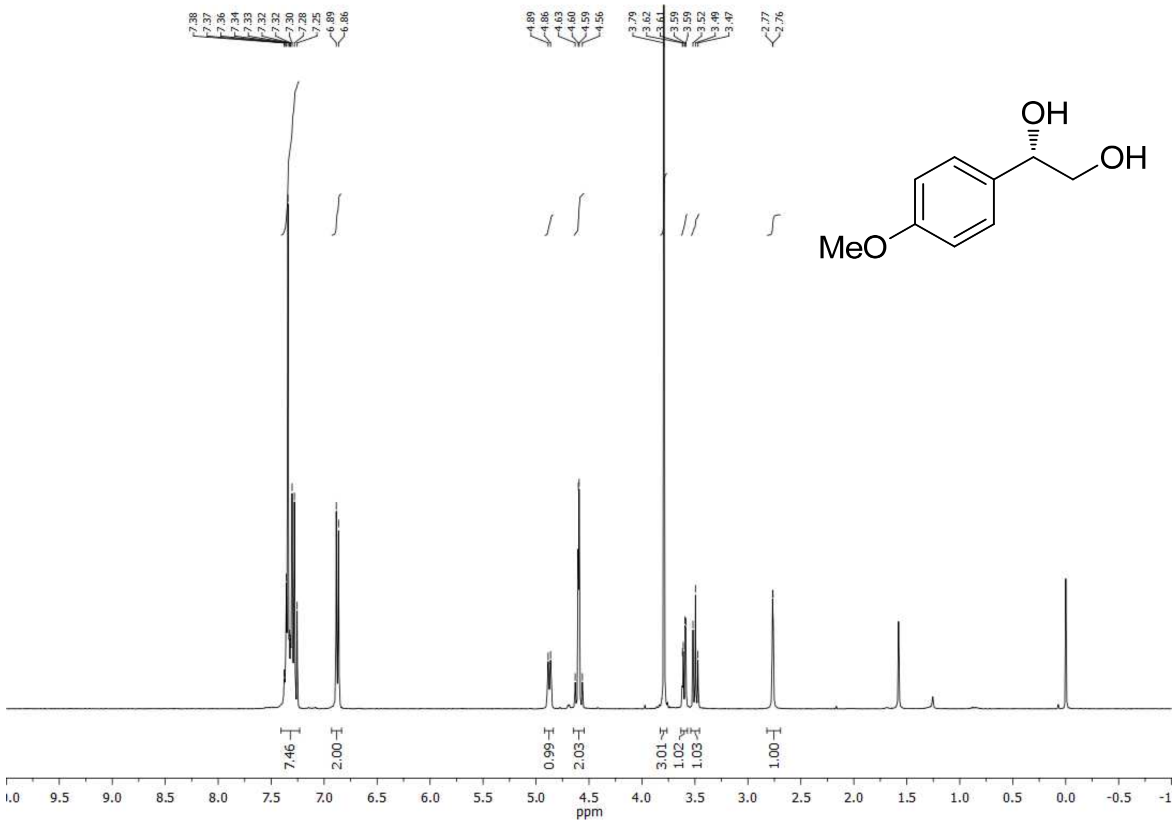

^{1}H NMR (400 MHz, $CDCl_3$)

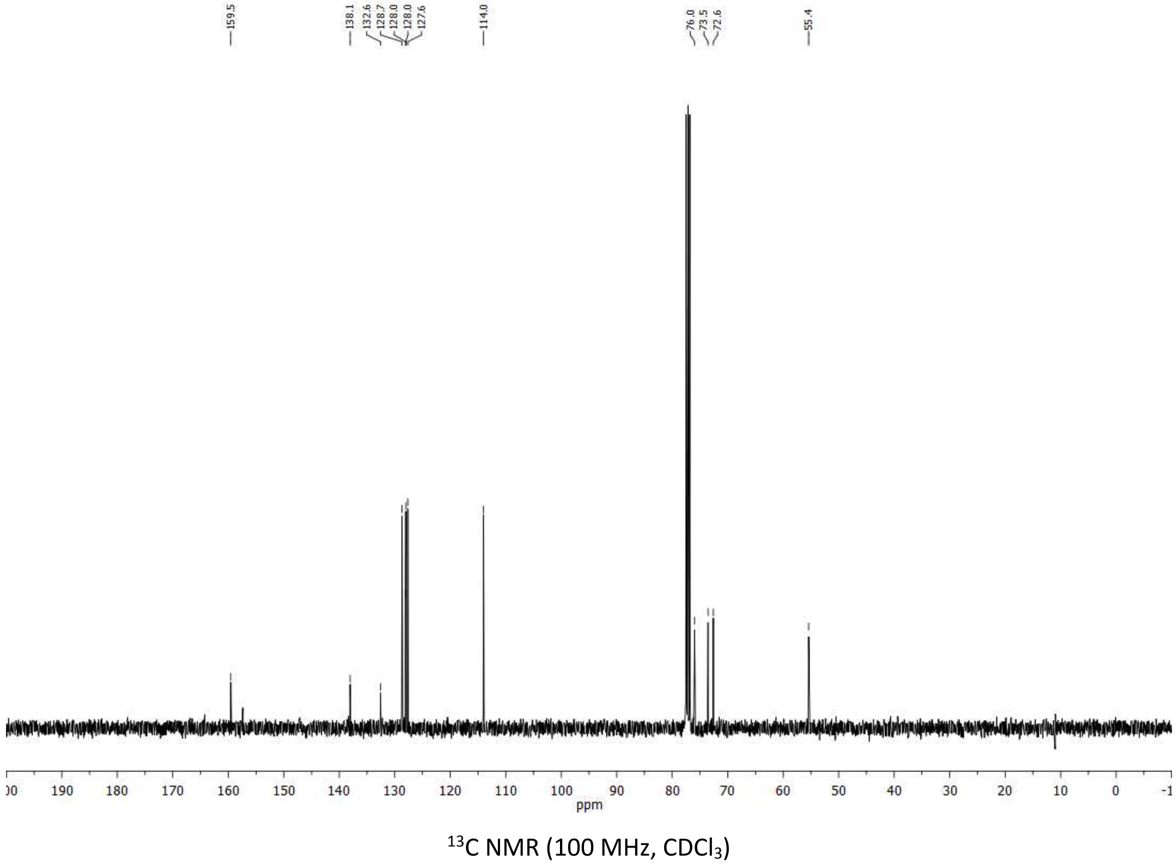

^{13}C NMR (100 MHz, $CDCl_3$)

(*S*)-2-(Benzyloxy)-1-cyclohexylethanol (**2ja**)

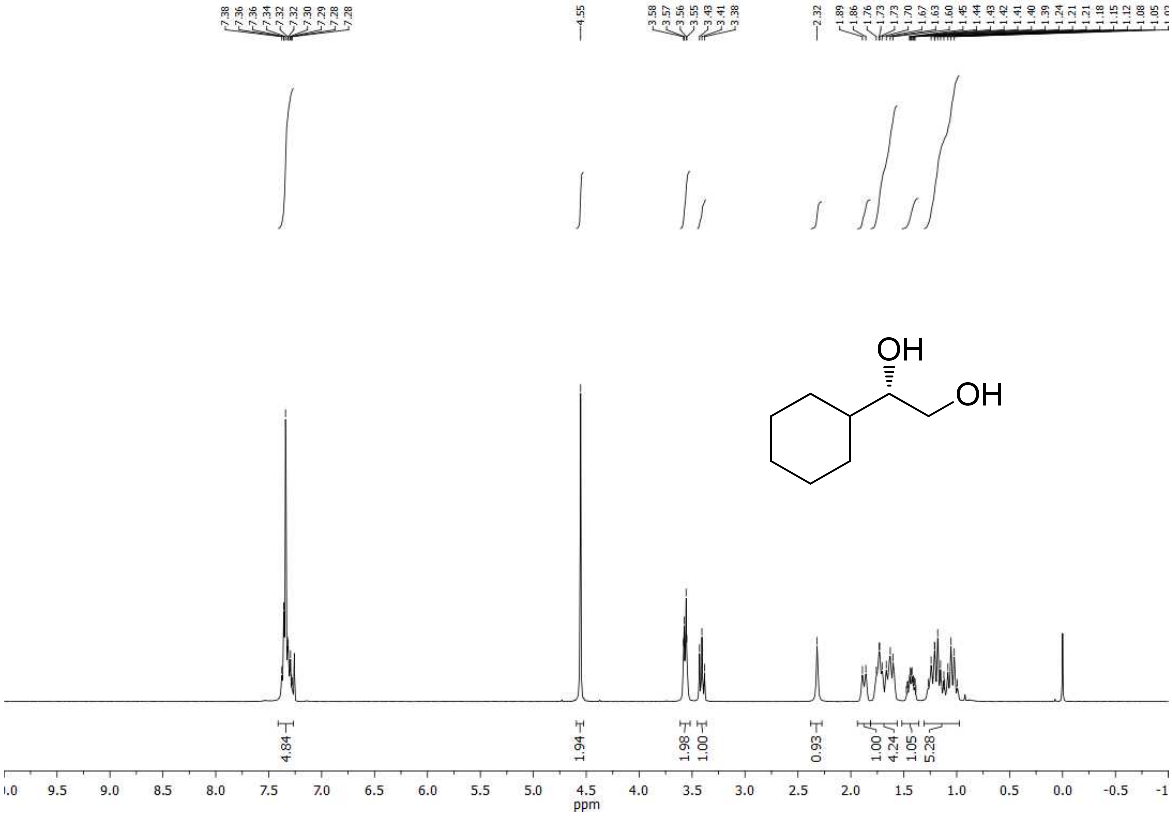

^{1}H NMR (400 MHz, $CDCl_3$)

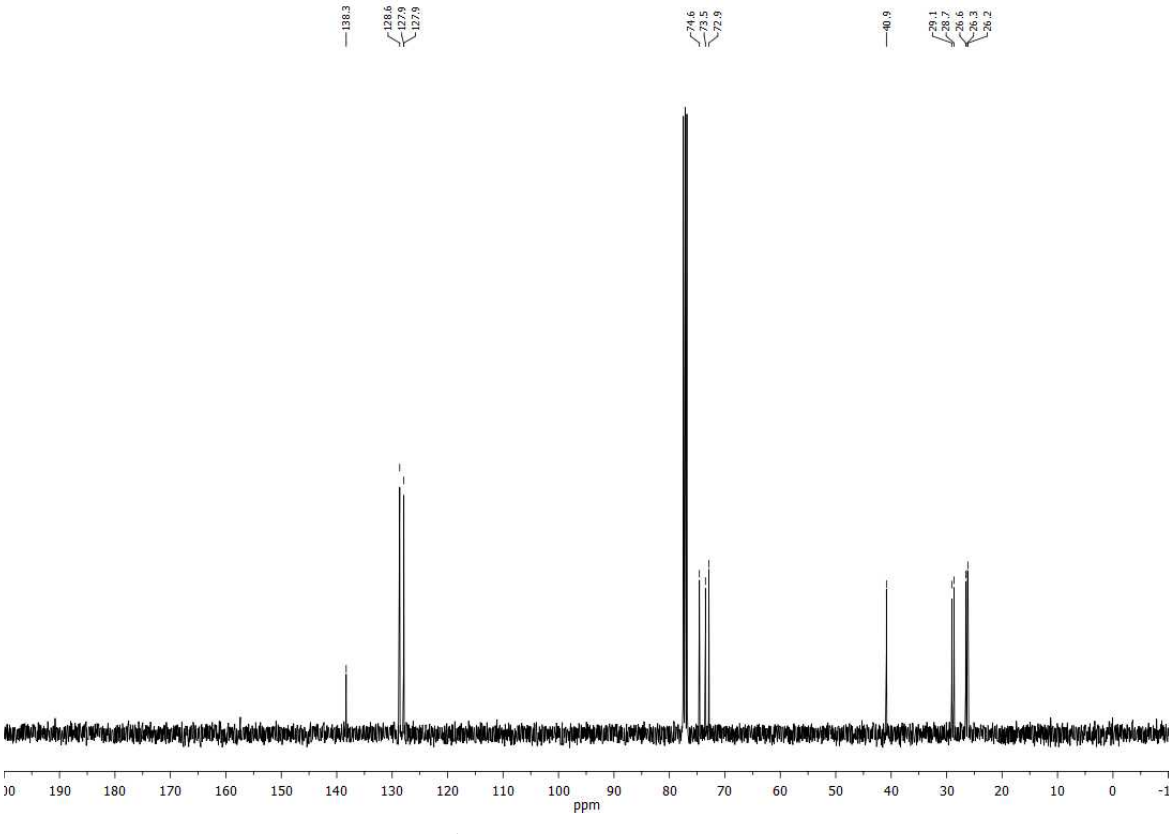

^{13}C NMR (100 MHz, $CDCl_3$)

(*S*)-1-(benzyloxy)-2-phenylpropan-2-ol (**13a**)

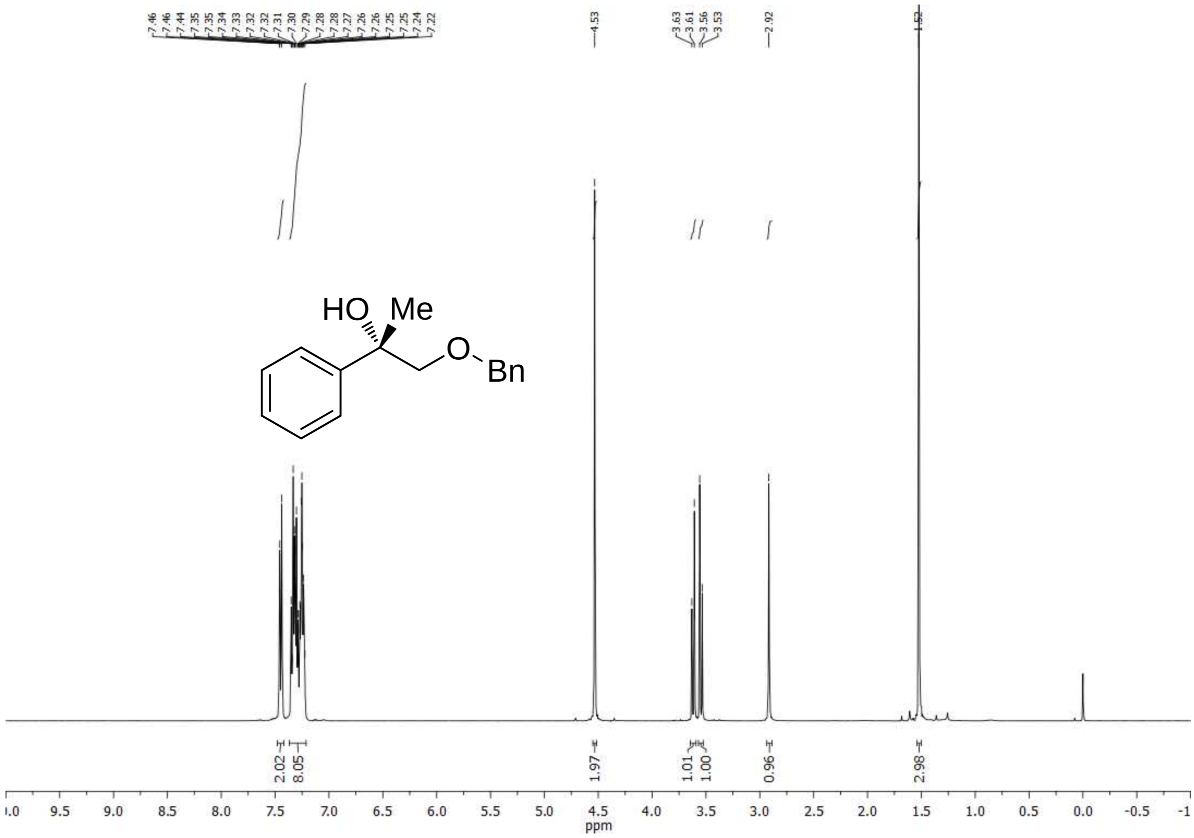

^{1}H NMR (400 MHz, $CDCl_3$)

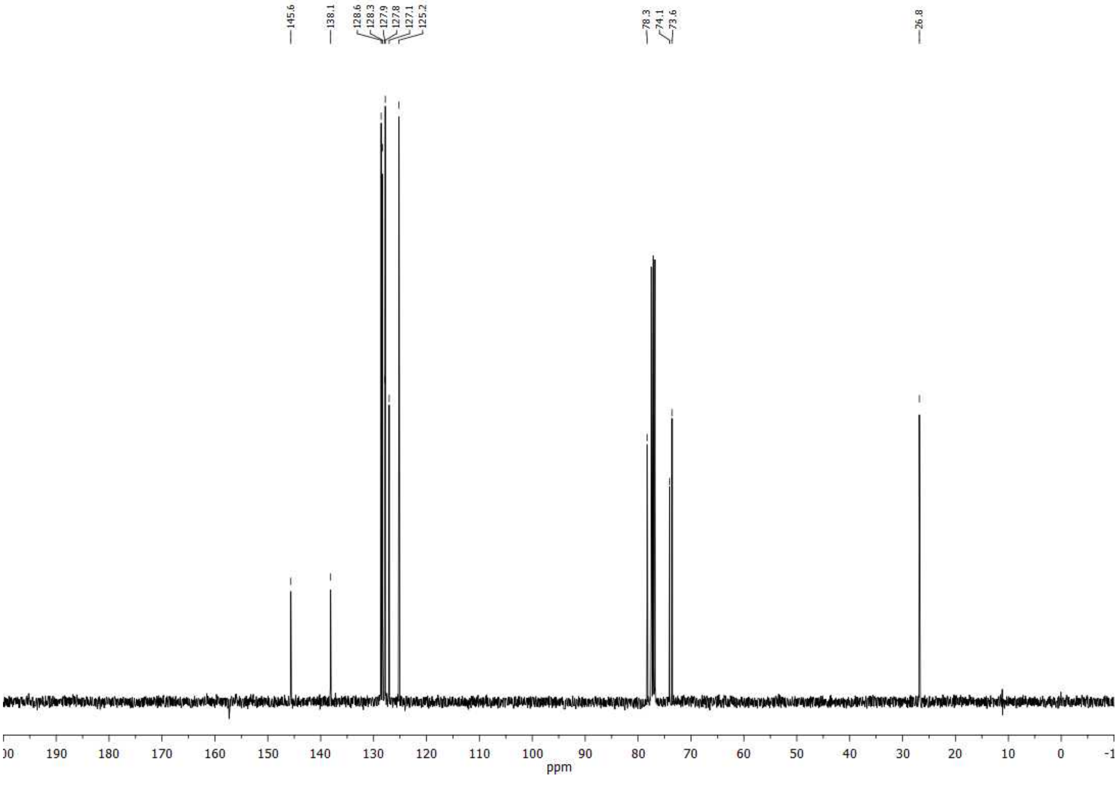

^{13}C NMR (100 MHz, $CDCl_3$)

(*S*)-1-(benzyloxy)-2-(*o*-tolyl)propan-2-ol (**13b**)

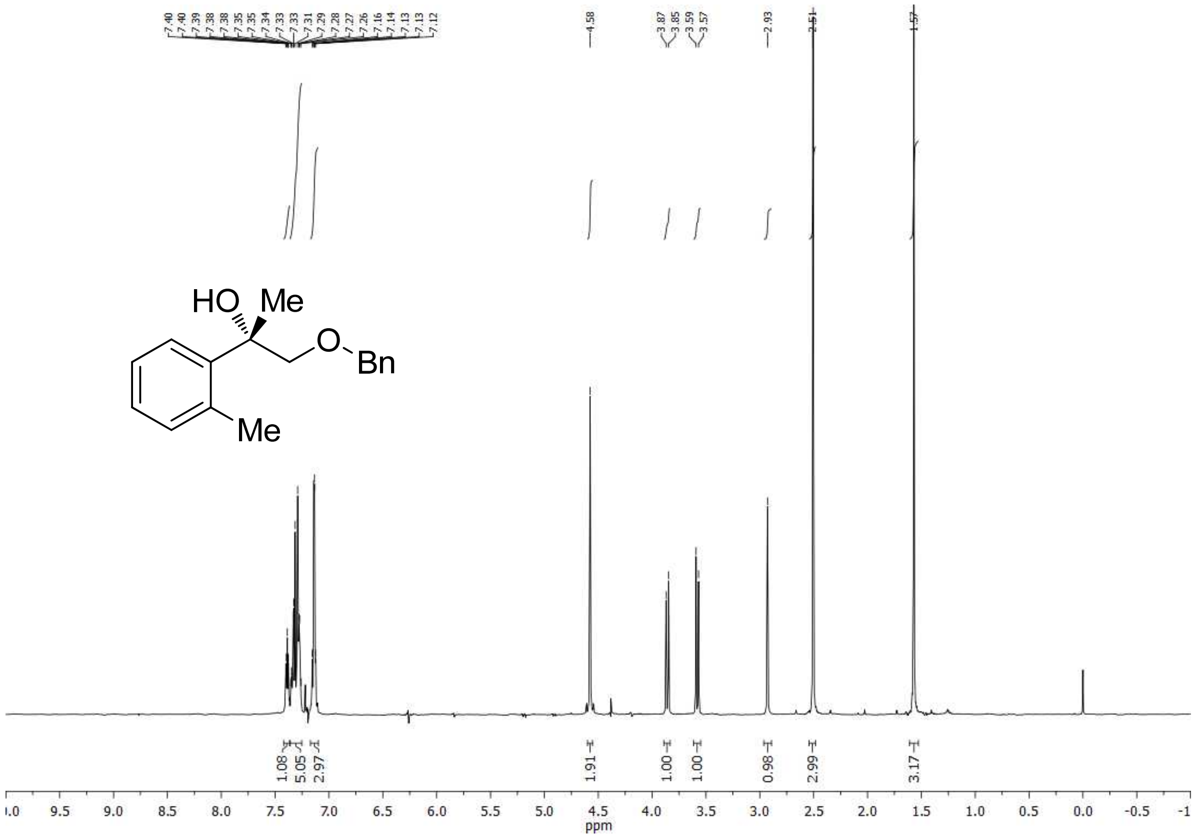

^{1}H NMR (400 MHz, $CDCl_3$)

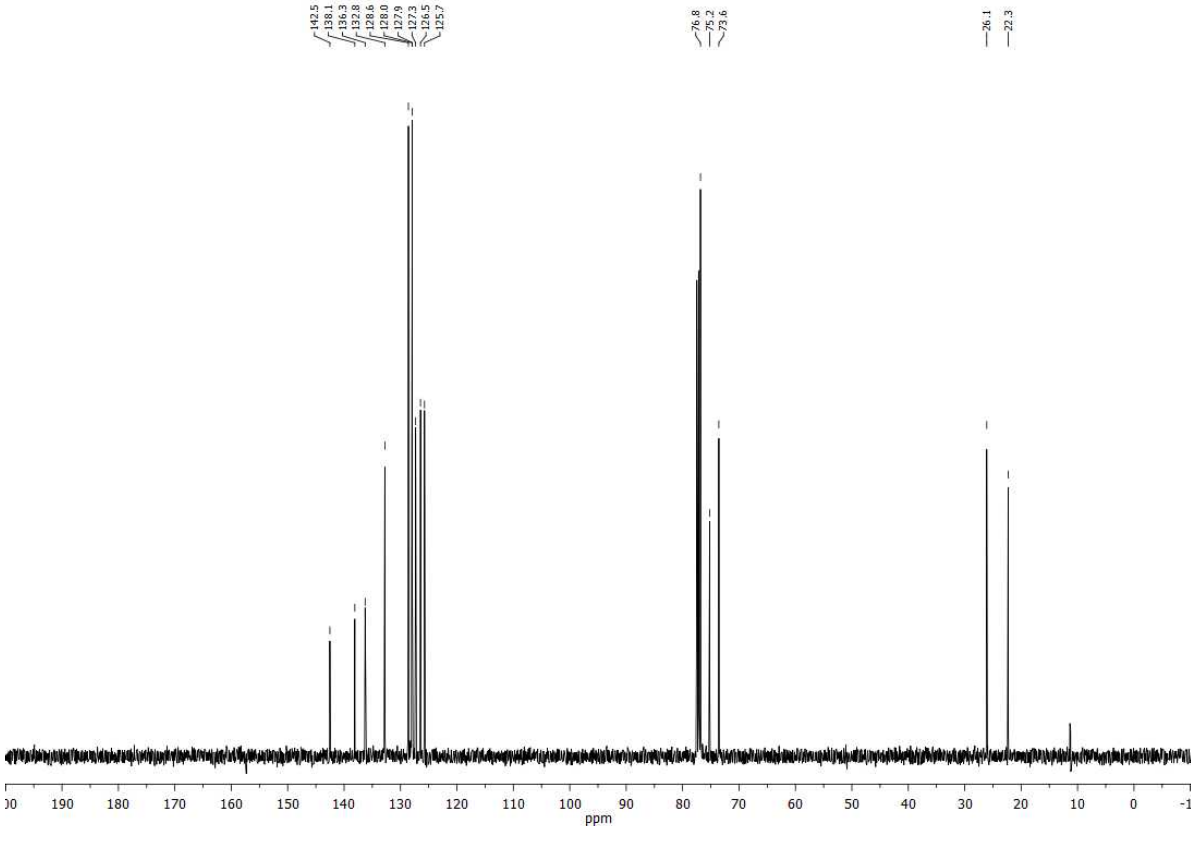

^{13}C NMR (100 MHz, $CDCl_3$)

(*S*)-1-(Benzyloxy)-2-(*m*-tolyl)propan-2-ol (**13c**)

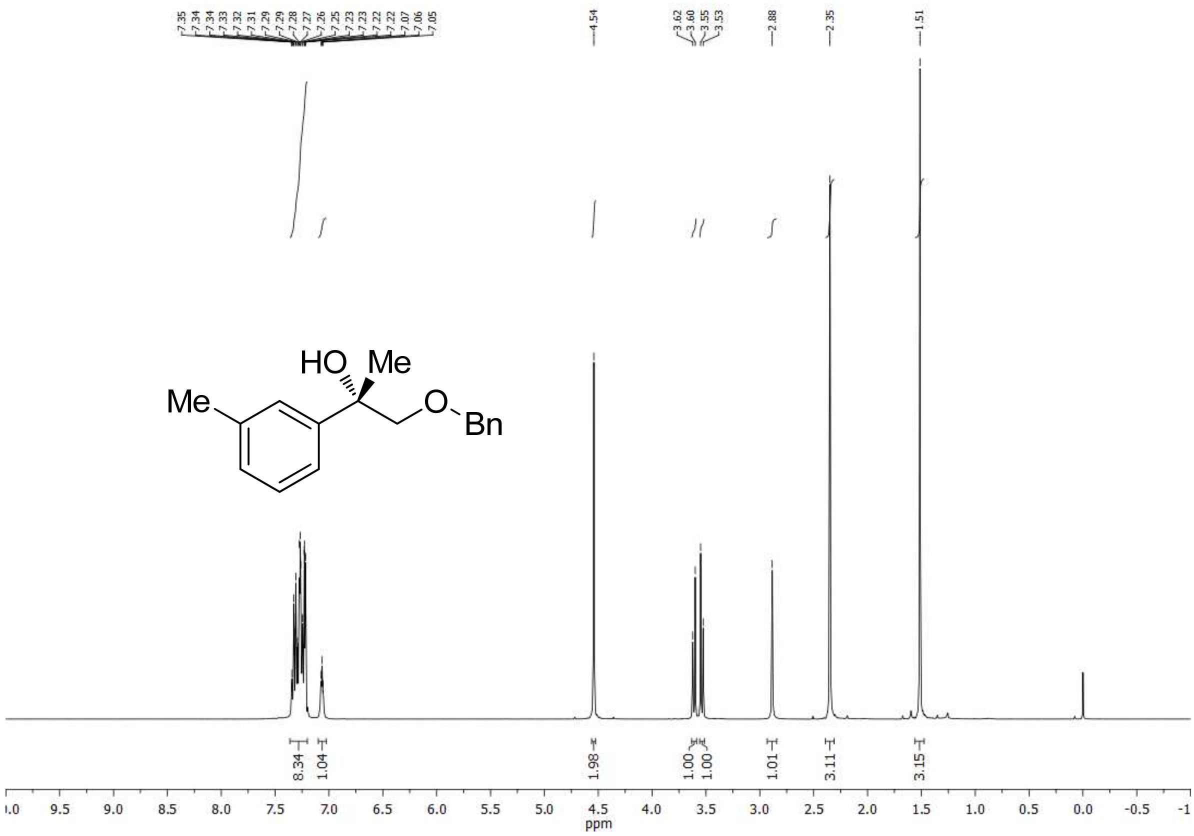

^{1}H NMR (400 MHz, $CDCl_3$)

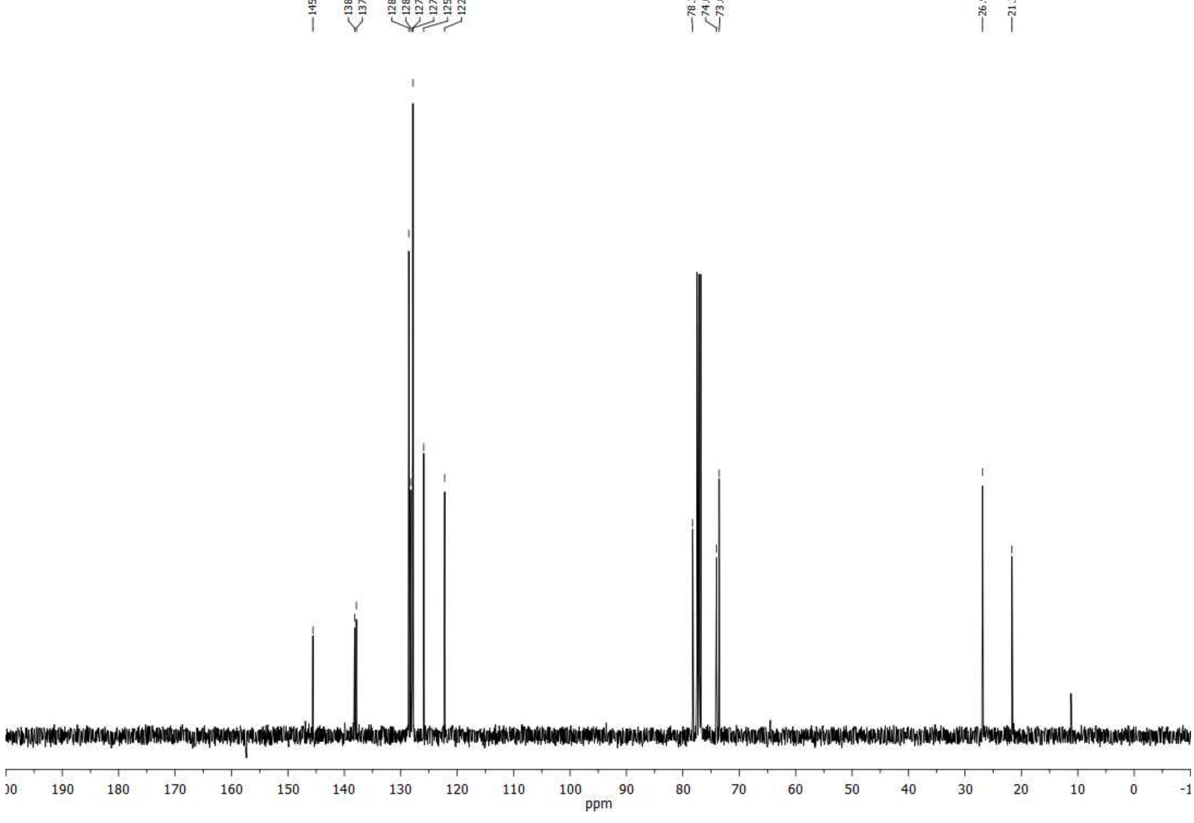

^{13}C NMR (100 MHz, $CDCl_3$)

(*S*)-1-(Benzyloxy)-2-(*p*-tolyl)propan-2-ol (**13d**)

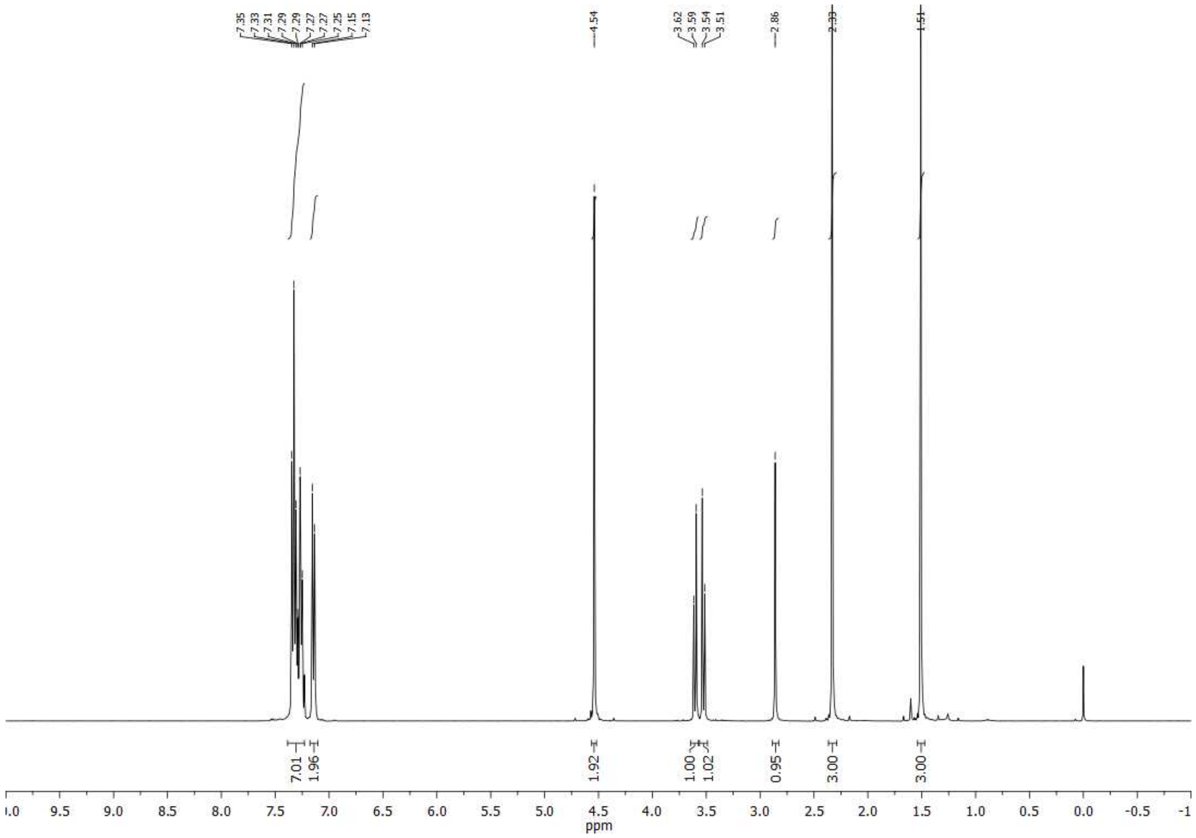

^{1}H NMR (400 MHz, $CDCl_3$)

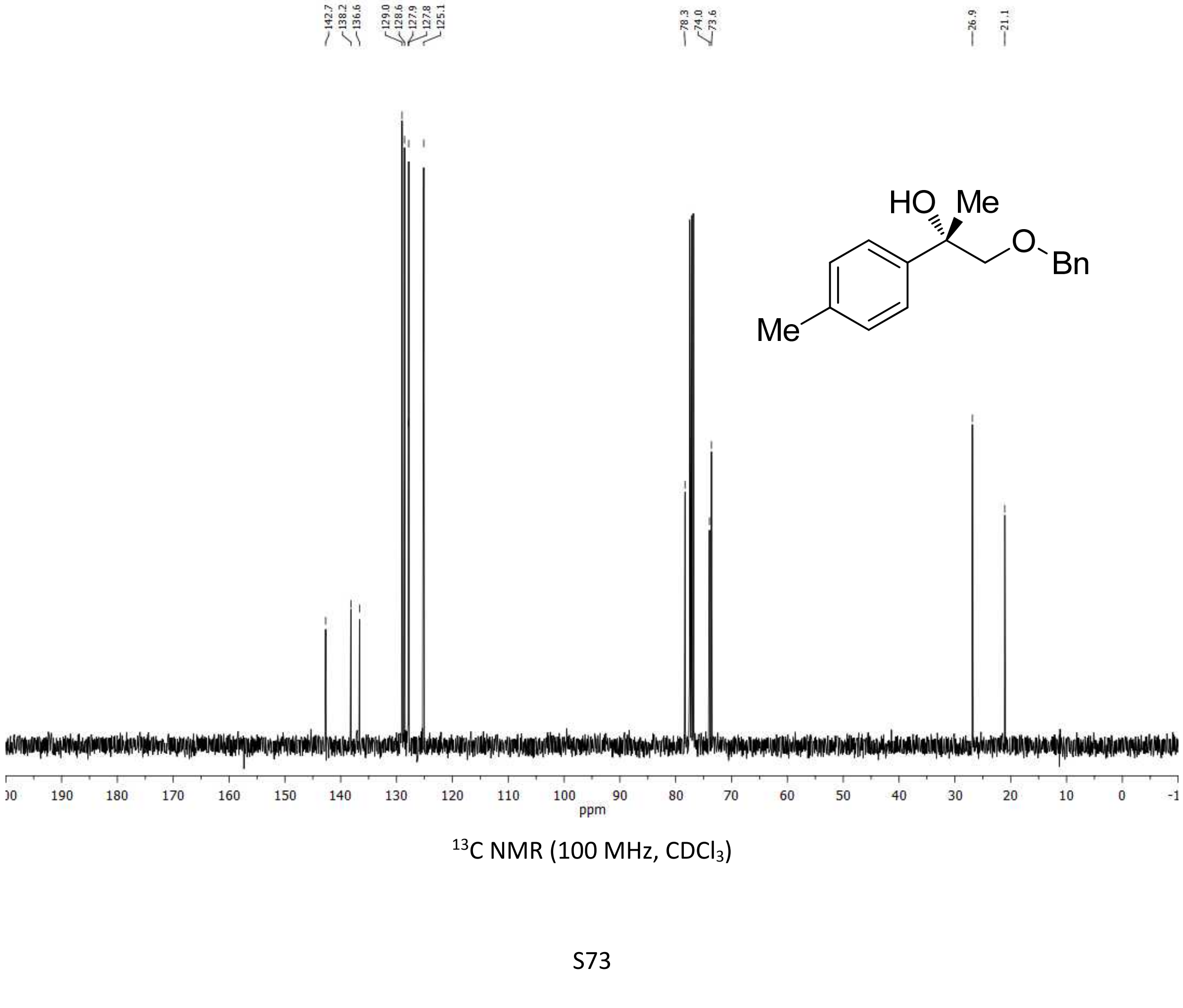

^{13}C NMR (100 MHz, $CDCl_3$)

(*S*)-1-(Benzyloxy)-2-(4-fluorophenyl)propan-2-ol (**13e**)

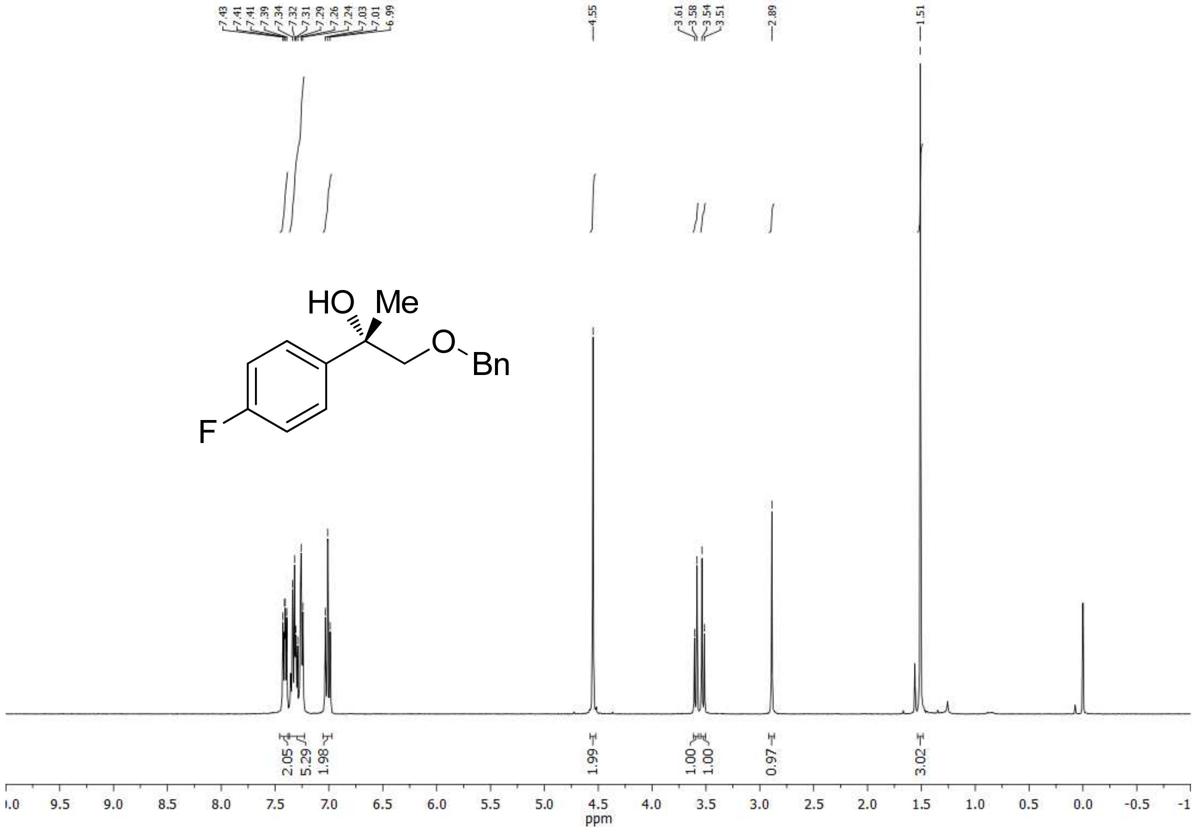

^{1}H NMR (400 MHz, CDCl$_3$)

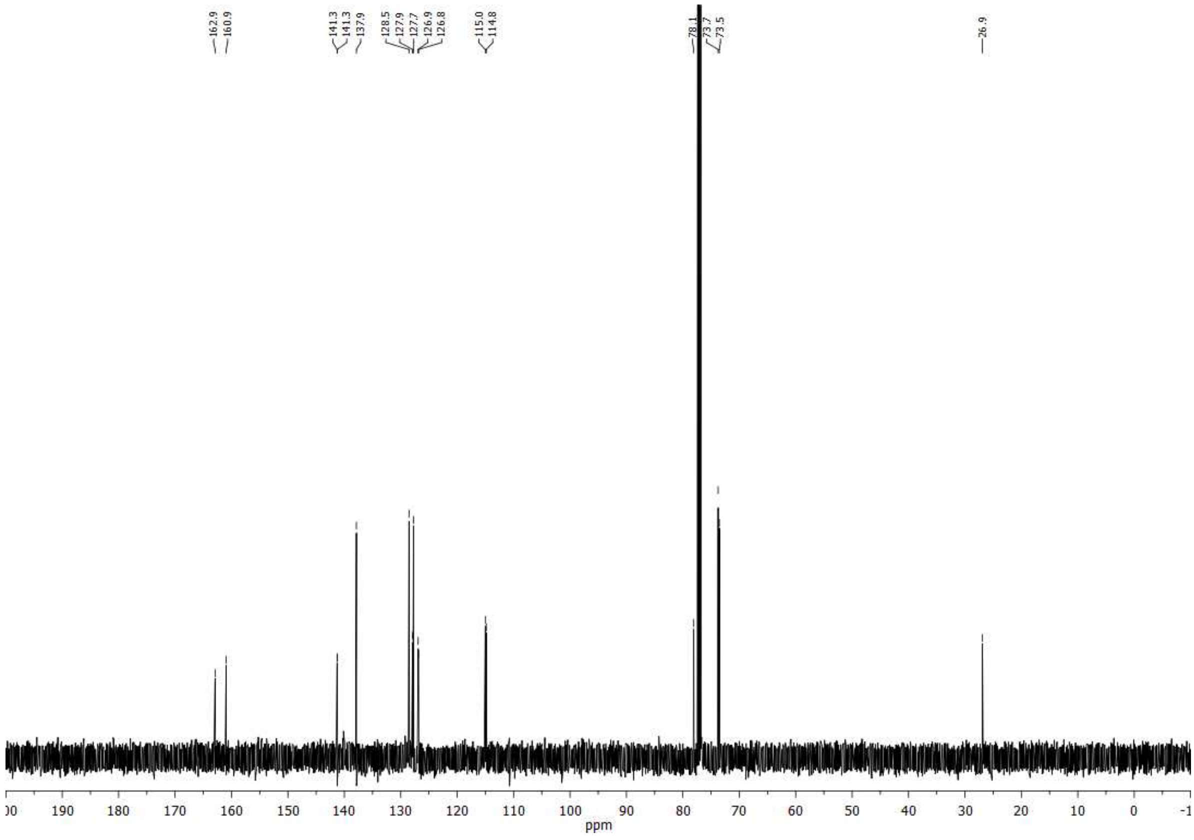

^{13}C NMR (125 MHz, CDCl$_3$)

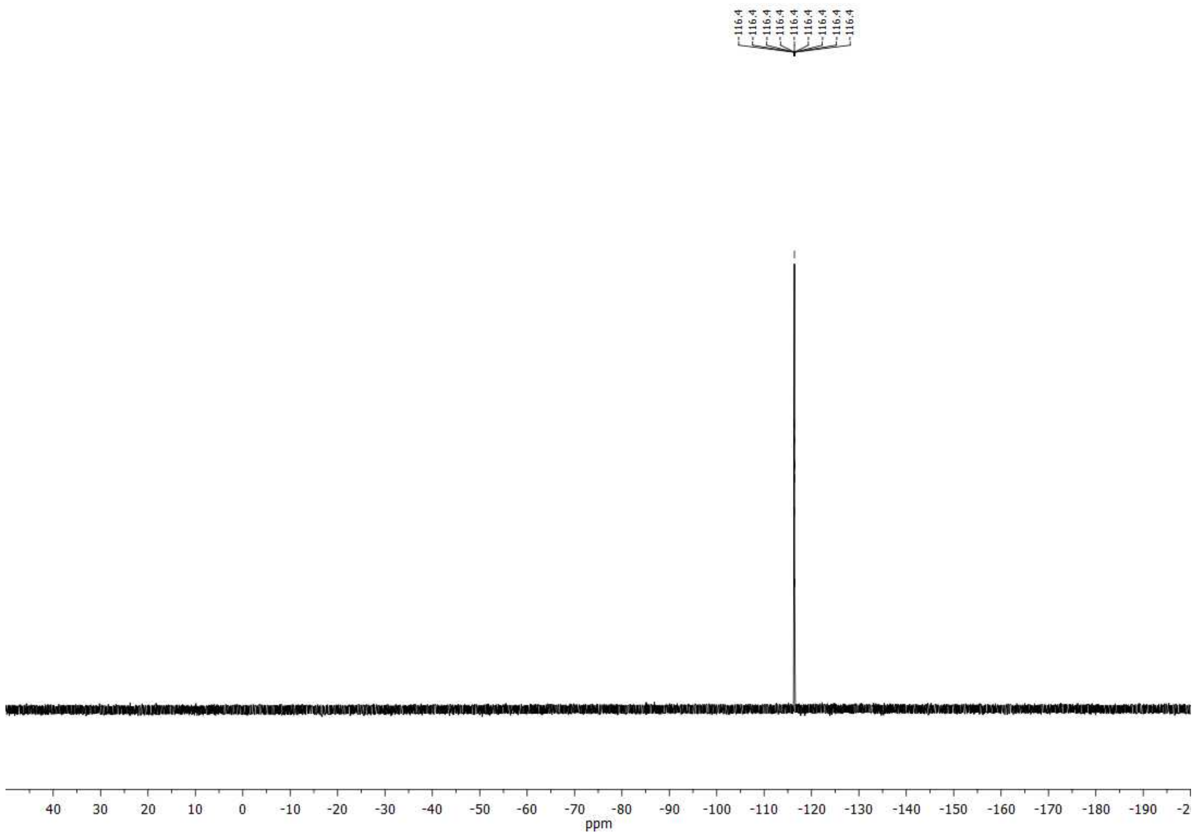

^{19}F NMR (466 MHz, $CDCl_3$)

(*S*)-1-(Benzyloxy)-2-(4-methoxyphenyl)propan-2-ol (**13f**)

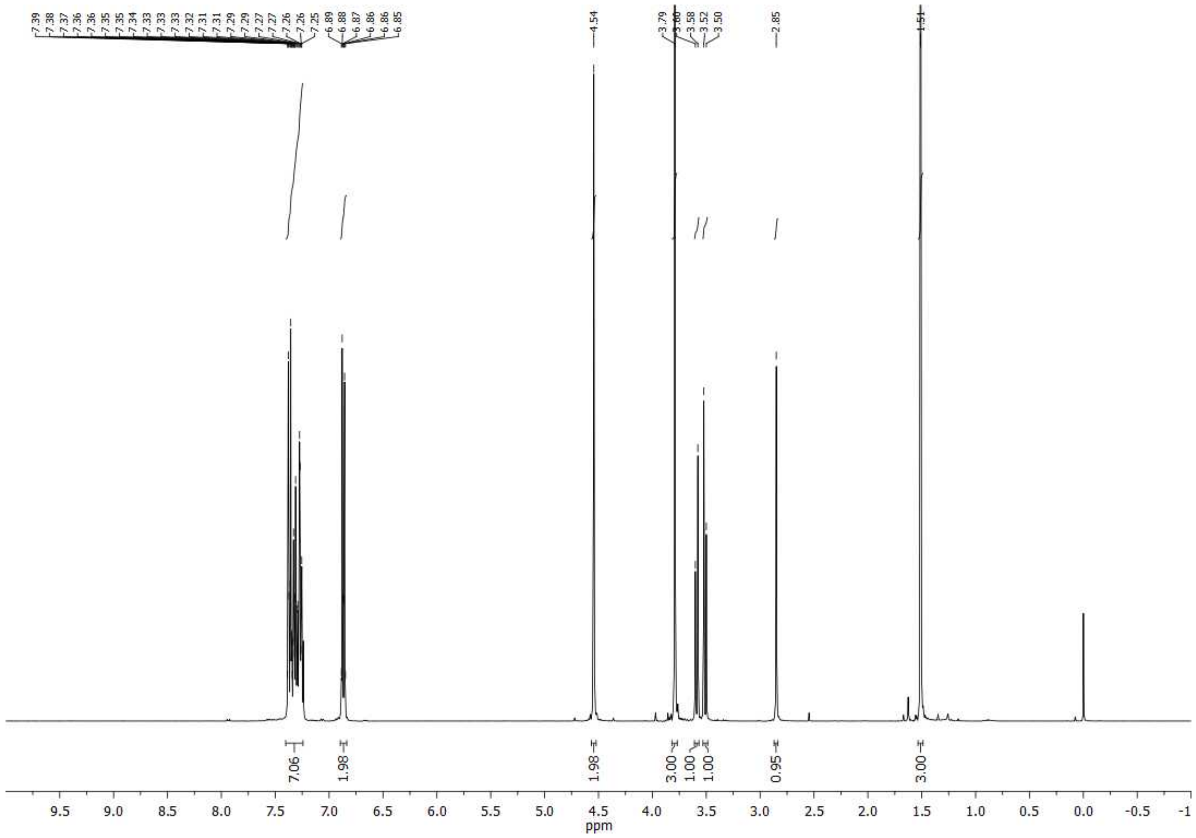

^{1}H NMR (400 MHz, CDCl$_3$)

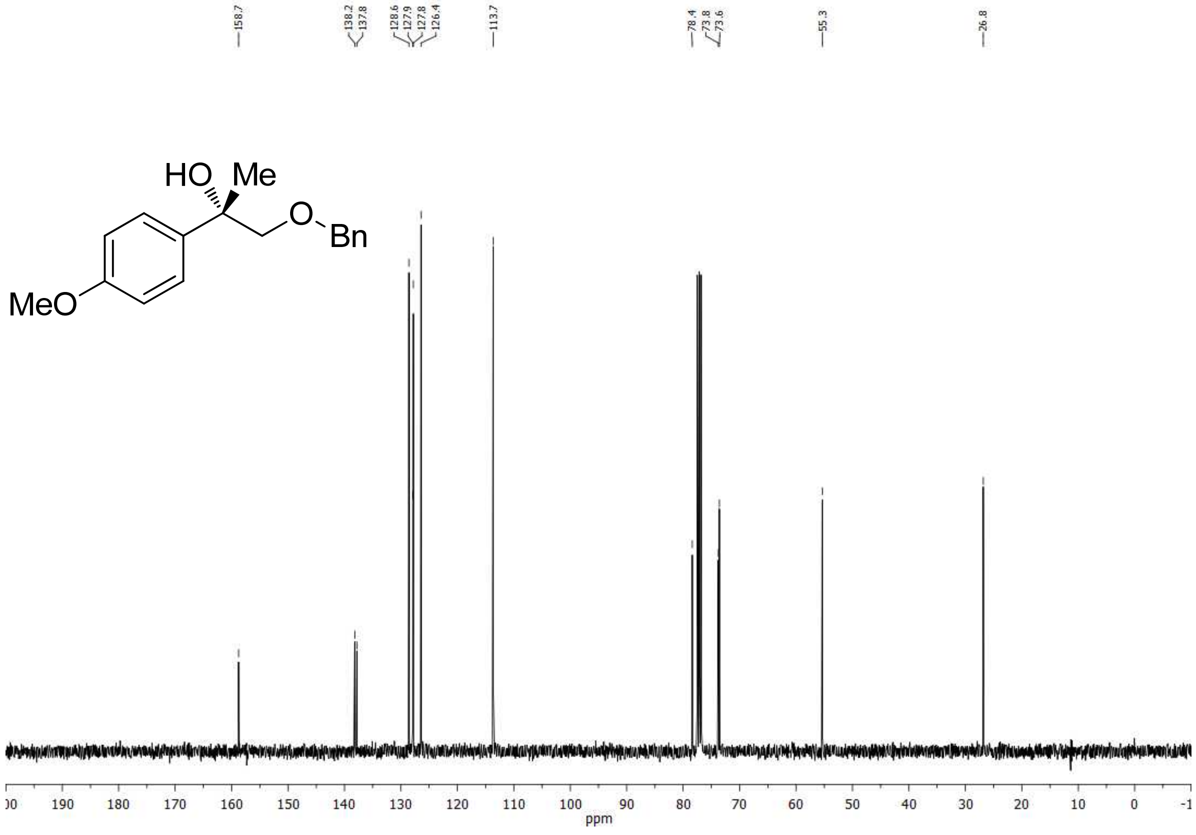

^{13}C NMR (100 MHz, CDCl$_3$)

(*S*)-1-(Benzyloxy)-2-(naphthalen-2-yl)propan-2-ol (**13g**)

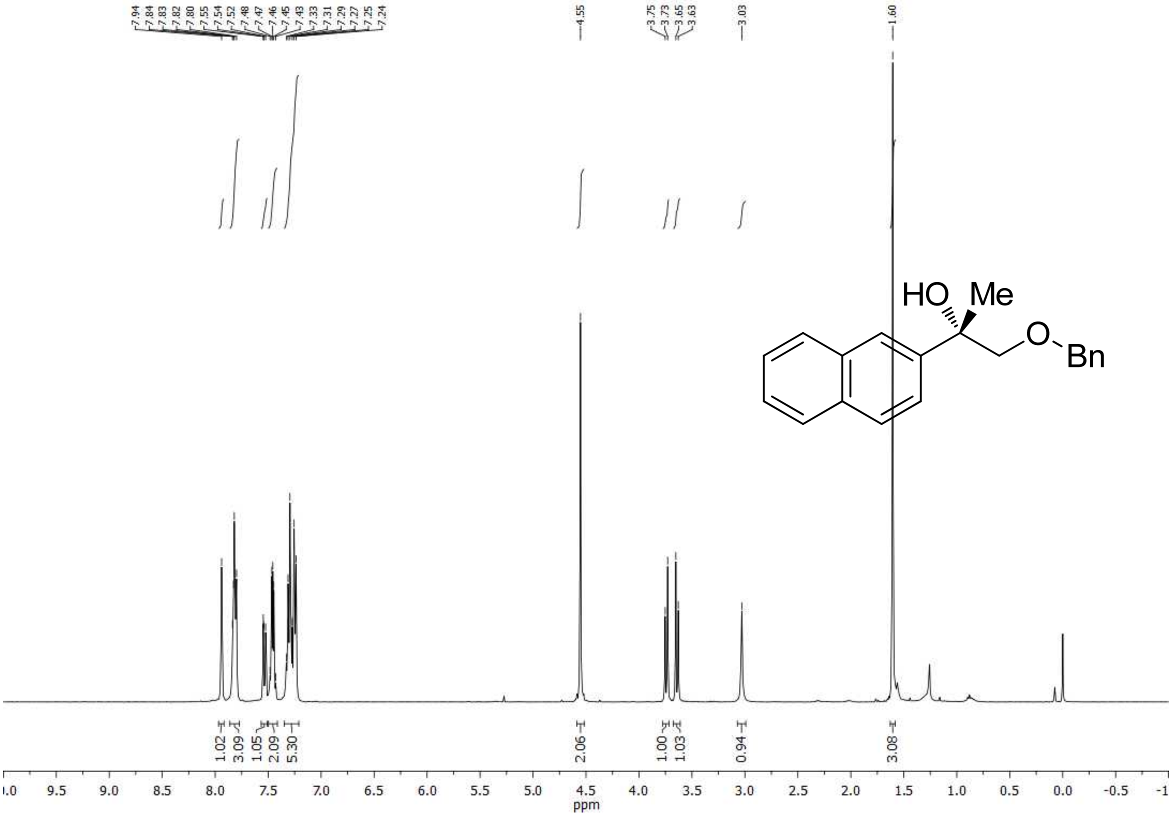

^{1}H NMR (400 MHz, $CDCl_3$)

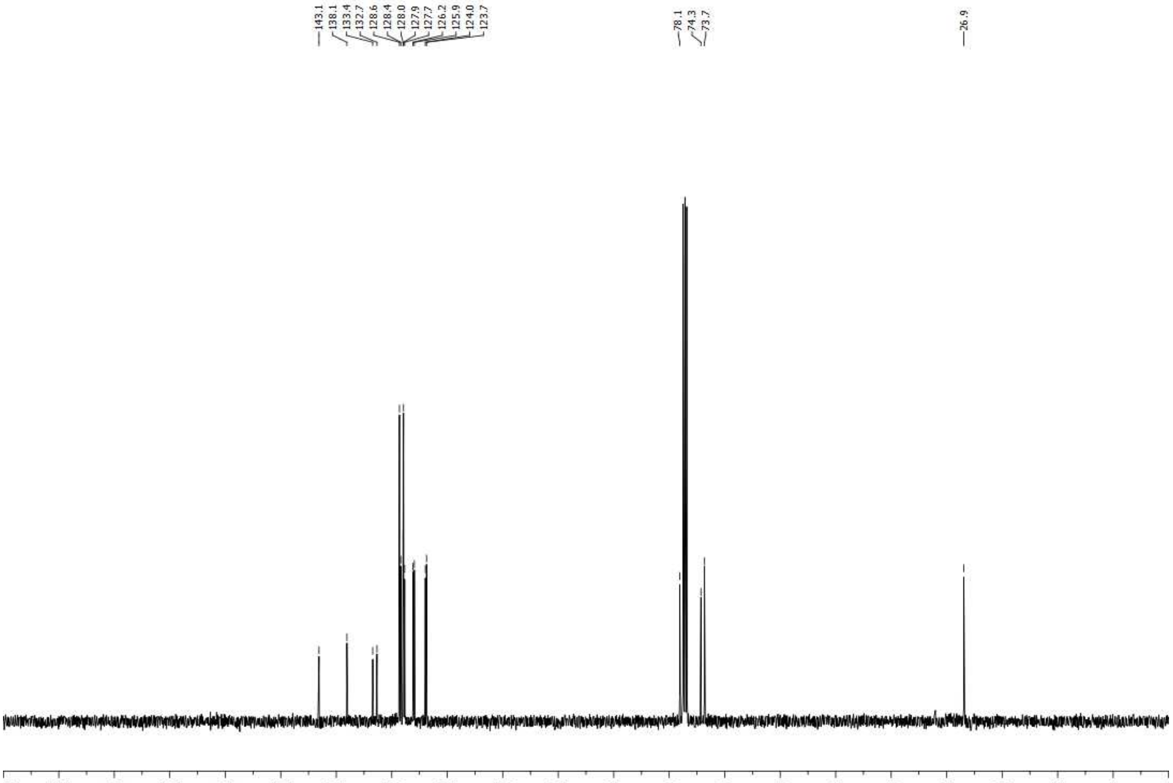

^{13}C NMR (100 MHz, $CDCl_3$)

(*S*)-1-(Benzyloxy)-2-(thiophen-3-yl)propan-2-ol (**13h**)

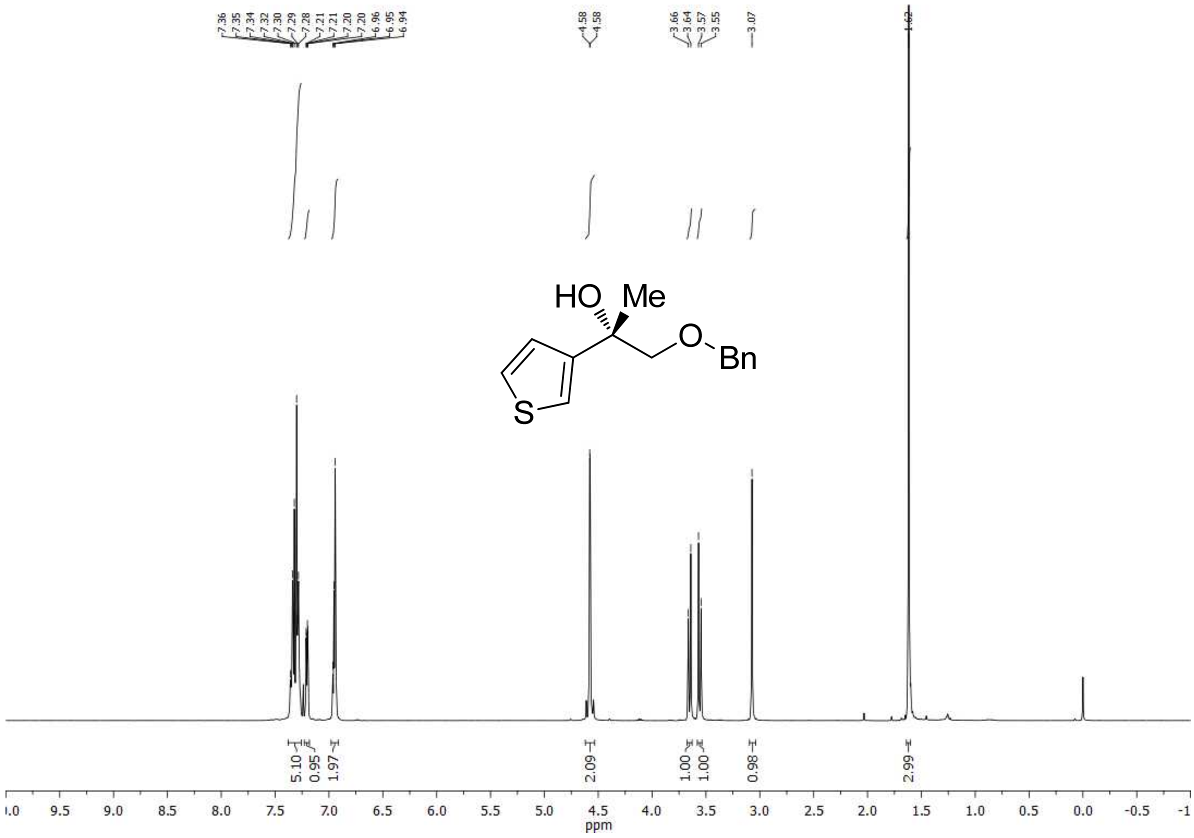

^{1}H NMR (400 MHz, $CDCl_3$)

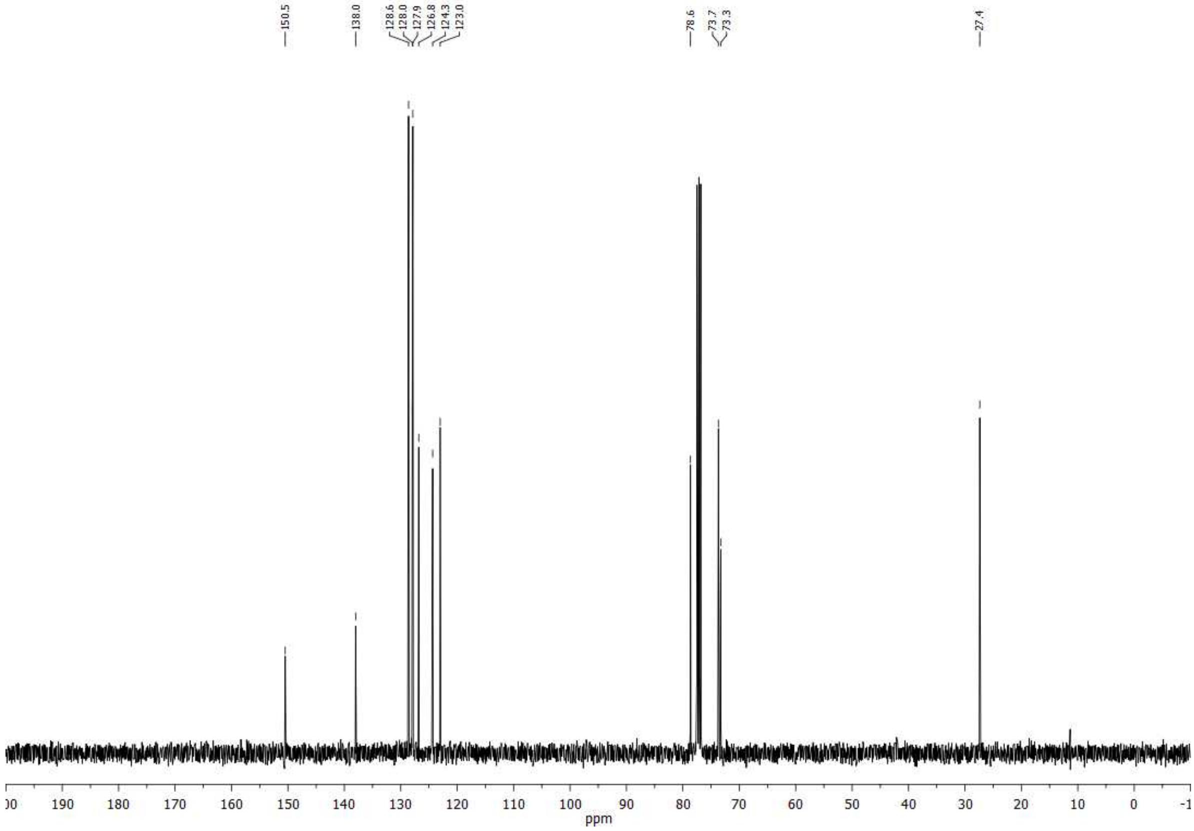

^{13}C NMR (100 MHz, $CDCl_3$)

(*S*)-1-(Benzyloxy)-2-phenylbut-3-en-2-ol (**13i**)

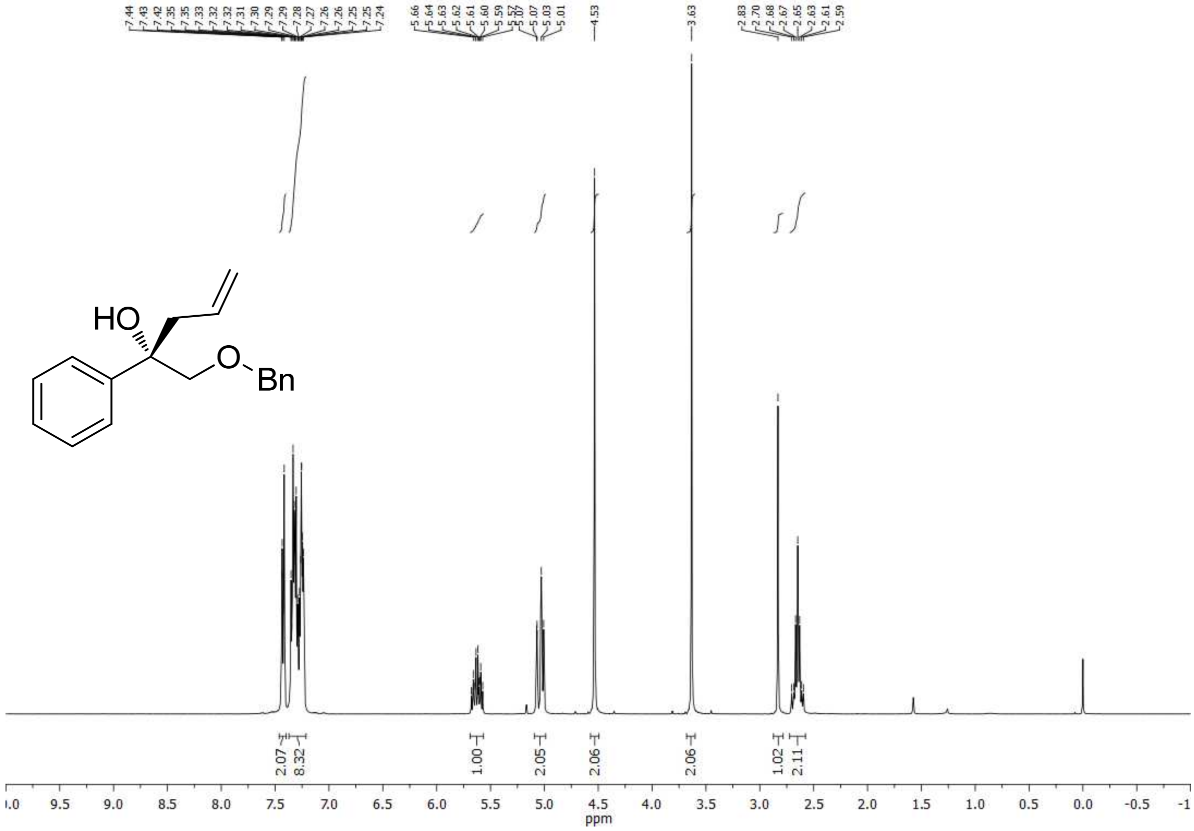

^{1}H NMR (400 MHz, $CDCl_3$)

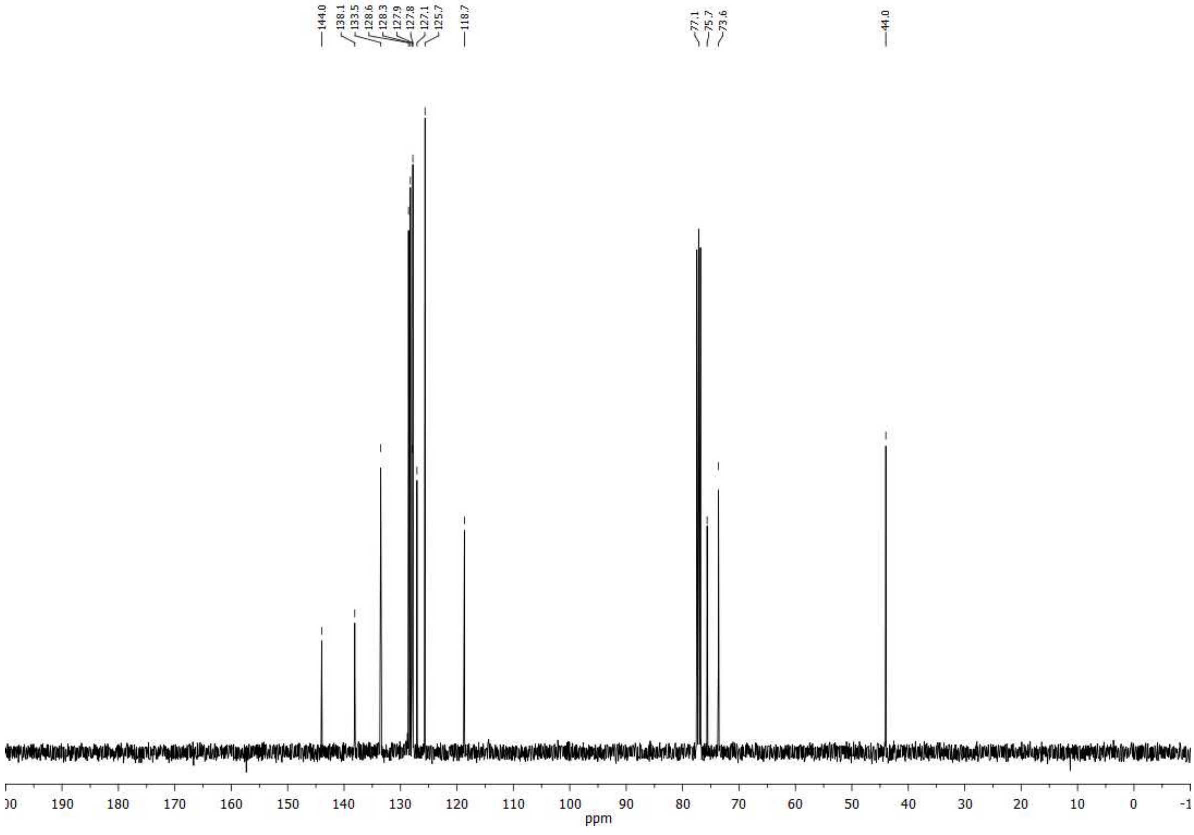

^{13}C NMR (100 MHz, $CDCl_3$)

(*R*)-2-(benzyloxy)-2-phenylethanol (**3aa**)

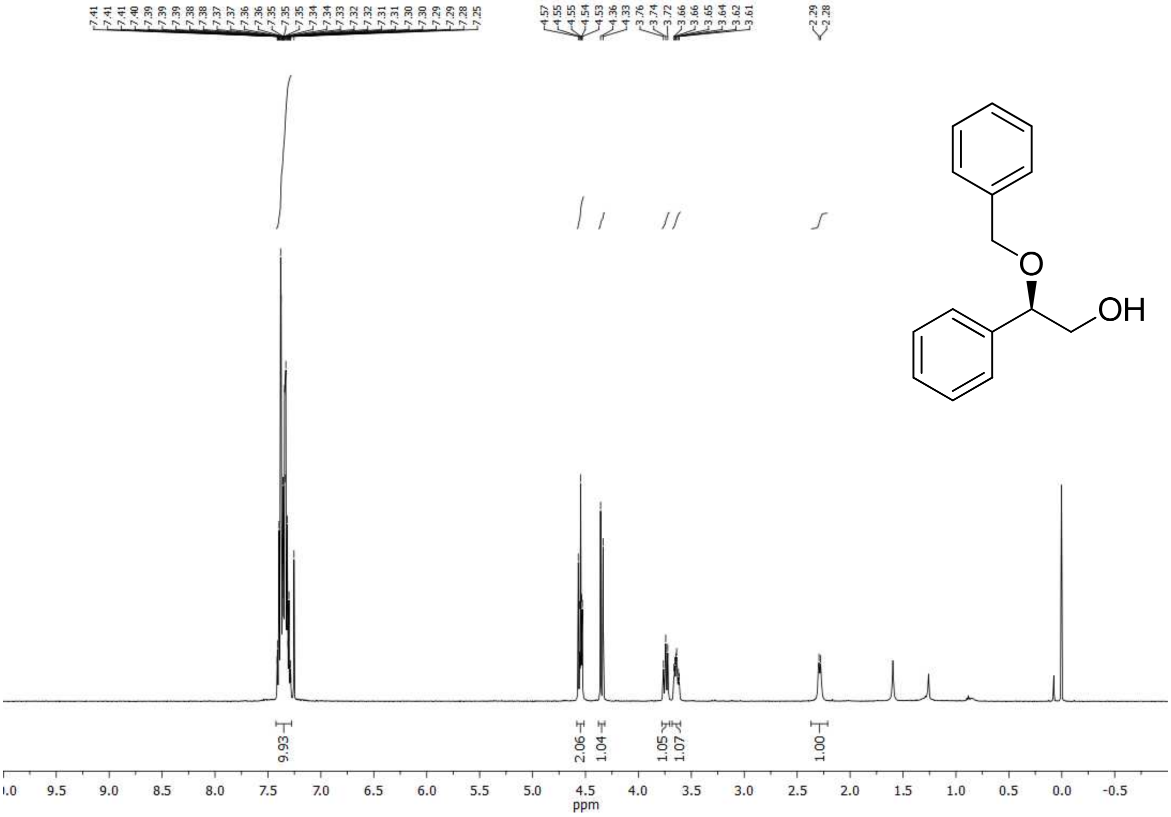

^{1}H NMR (500 MHz, CDCl$_3$)

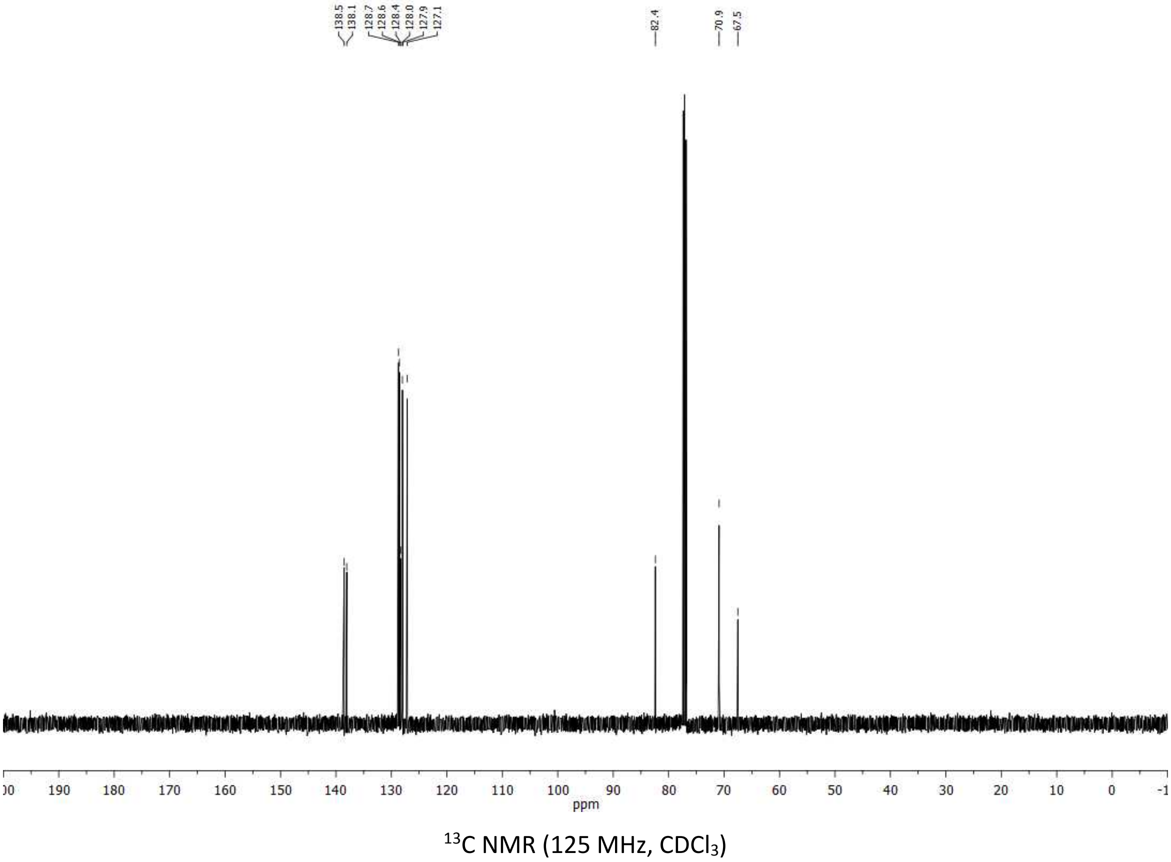

^{13}C NMR (125 MHz, CDCl$_3$)

(1*S*,2*R*)-2-((2-methylbenzyl)oxy)-1,2-diphenylethanol (**13**)

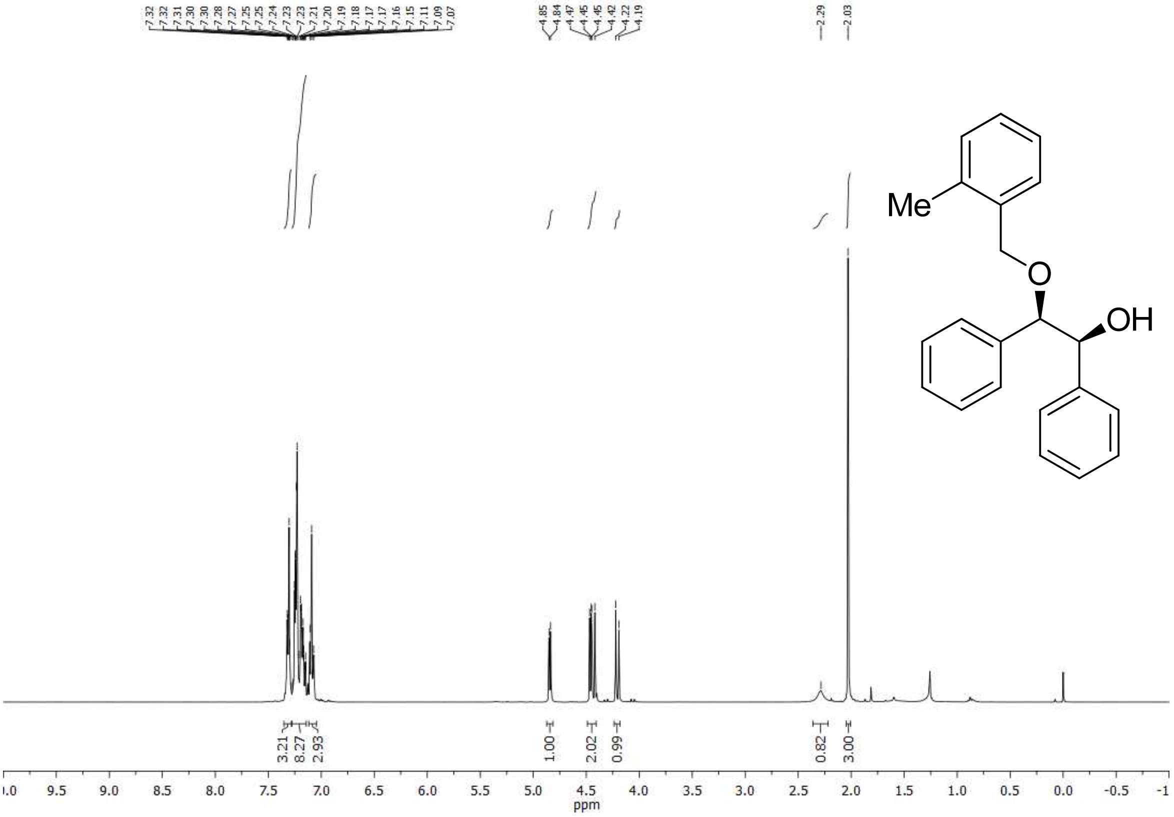

^{1}H NMR (400 MHz, $CDCl_3$)

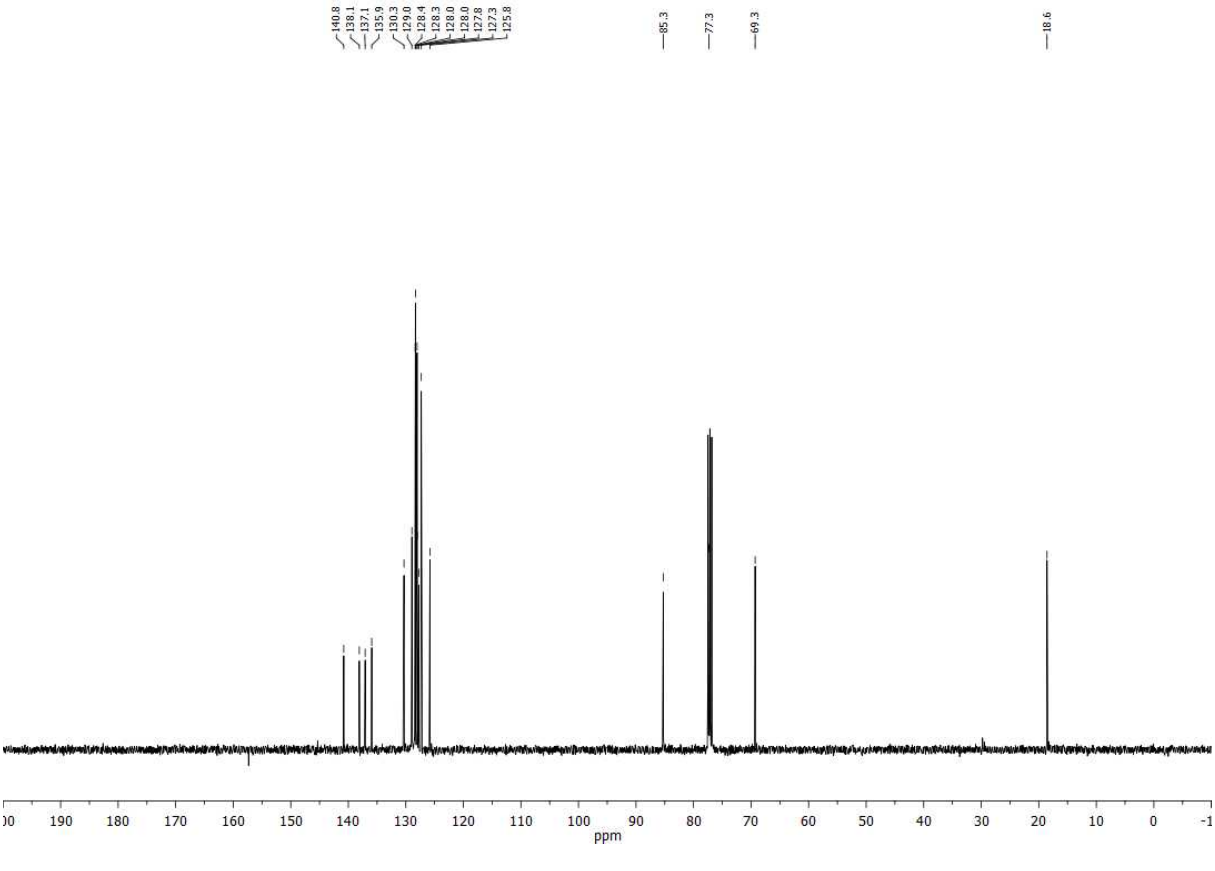

^{13}C NMR (100 MHz, $CDCl_3$)

HPLC

(*S*)-2-(Benzyloxy)-1-phenylethanol (**2aa**)

ﾍﾟｰｼﾞ 1/1

面積%レポート

ﾃﾞｰﾀﾌｧｲﾙ名 : C:¥Data(HPLC)¥Martin¥Project2¥Rac¥4_63_1_Pro_rac AD3 P60 0.5 210.dat
ﾒｿｯﾄﾞﾌｧｲﾙ名: C:¥Data(HPLC)¥Sasagawa¥Method 120 min.met
ﾕｰｻﾞｰ名: System
分析日時: 2017/03/23 9:38:09
印刷日時: 2017/04/12 11:04:05

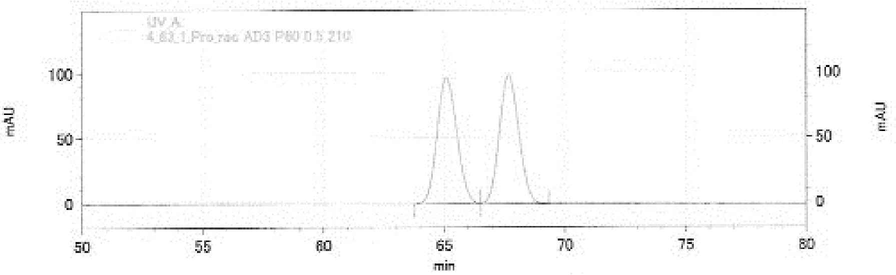

UV_A結果

Pk #	名前	保持時間	面積	面積%	ﾍﾞｰｽﾗｲﾝｺｰﾄﾞ
1		65.09	5522512	49.970	MM
2		67.67	5529148	50.030	MM
ﾄｰﾀﾙ			11051660	100.000	

ﾍﾟｰｼﾞ 1/1

面積%レポート

ﾃﾞｰﾀﾌｧｲﾙ名 : C:¥Data(HPLC)¥Martin¥MP-2-150_Pro_1 AD3 P60 0.5 210.dat
ﾒｿｯﾄﾞﾌｧｲﾙ名: C:¥Data(HPLC)¥Sasagawa¥Method 120 min.met
ﾕｰｻﾞｰ名: System
分析日時: 2016/07/07 10:34:48
印刷日時: 2017/04/11 15:37:48

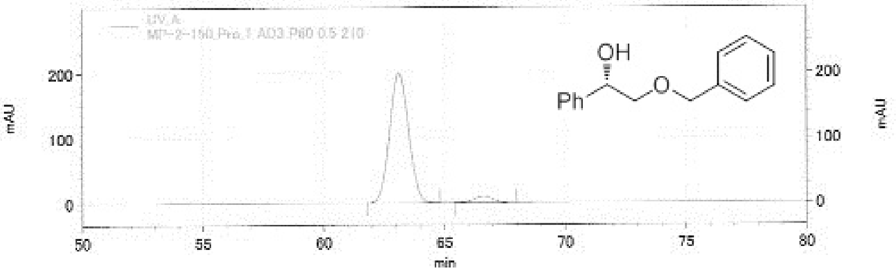

UV_A結果

Pk #	名前	保持時間	面積	面積%	ﾍﾞｰｽﾗｲﾝｺｰﾄﾞ
1		63.12	10980352	95.491	MM
2		66.61	518481	4.509	MM
ﾄｰﾀﾙ			11498833	100.000	

(*S*)-2-((2-Methylbenzyl)oxy)-1-phenylethanol (**2ab**)

面積%レポート

デ－タファイル名： C:¥Data(HPLC)¥Martin¥Project2¥Rac¥4_63_3_Pro_rac2 AD3 P60 0.5 210.dat
メソッド ファイル名： C:¥Data(HPLC)¥Sasagawa¥Method 120 min.met
ユ－ザ－名： System
分析日時： 2017/03/24 13:42:58
印刷日時： 2017/04/12 11:09:31

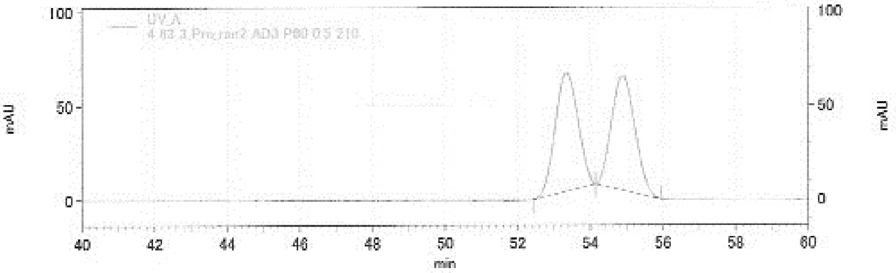

UV_A結果

Pk #	名前	保持時間	面積	面積%	ベ－スラインコ－ド
1		53.34	2760667	50.041	MM
2		54.90	2756089	49.959	MM
ト－タル			5516756	100.000	

ペ－ジ 1/1

面積%レポート

デ－タファイル名： C:¥Data(HPLC)¥Martin¥MP-2-148_Pro_1 AD3 P60 0.5 210.dat
メソッド ファイル名： C:¥Data(HPLC)¥Sasagawa¥Method 120 min.met
ユ－ザ－名： System
分析日時： 2016/07/06 14:38:41
印刷日時： 2017/04/11 15:44:05

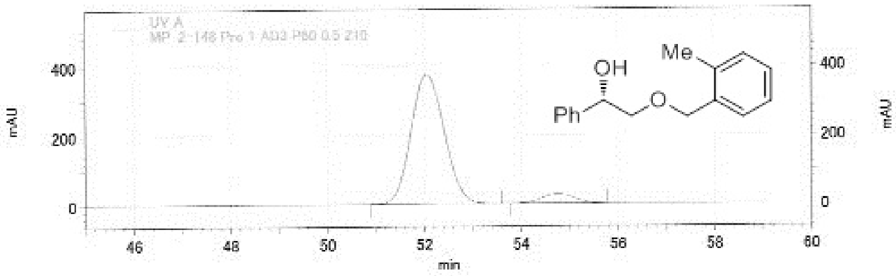

UV_A結果

Pk #	名前	保持時間	面積	面積%	ベ－スラインコ－ド
1		52.07	17742499	93.443	MM
2		54.75	1245078	6.557	MM
ト－タル			18987577	100.000	

(*S*)-2-((3-Methylbenzyl)oxy)-1-phenylethanol (**2ac**)

面積%レポート

デ-タファイル名 : C:¥Data(HPLC)¥Martin¥Project2¥Rac¥4_63_9_rac IE P60 0.5 210.dat
メソッドファイル名 : C:¥Data(HPLC)¥Sasagawa¥Method 120 min.met
ユ-ザ-名 : System
分析日時 : 2017/04/03 18:12:48
印刷日時 : 2017/04/12 11:16:32

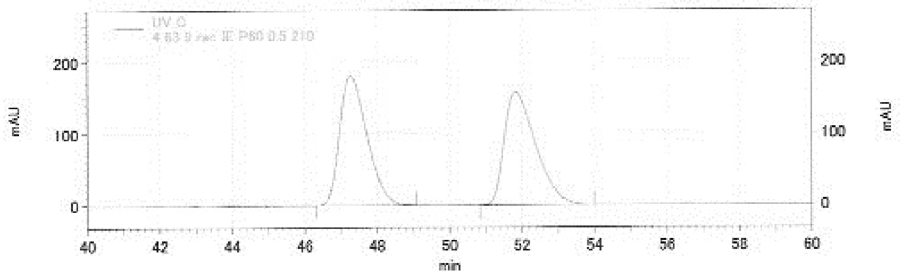

UV_C結果

Pk #	名前	保持時間	面積	面積%	ベ-スラインコ-ド
1		47.27	9526167	49.944	MM
2		51.83	9547545	50.056	MM
ト-タル			19073712	100.000	

ペ-ジ 1/1

面積%レポート

デ-タファイル名 : C:¥Data(HPLC)¥Martin¥2_194_pro IE P60 0.5 210.dat
メソッドファイル名 : C:¥Data(HPLC)¥Sasagawa¥Method 120 min.met
ユ-ザ-名 : System
分析日時 : 2017/04/13 17:44:25
印刷日時 : 2017/04/13 18:45:28

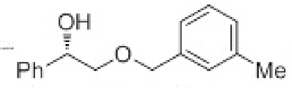

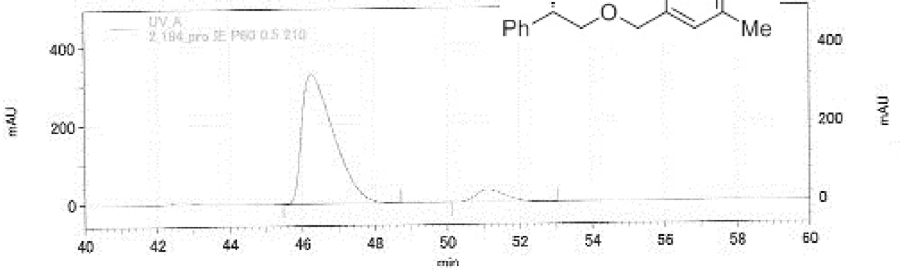

UV_A結果

Pk #	名前	保持時間	面積	面積%	ベ-スラインコ-ド
1		46.26	20331949	91.501	MM
2		51.12	1888434	8.499	MM
ト-タル			22220383	100.000	

(*S*)-2-((4-Methylbenzyl)oxy)-1-phenylethanol (**2ad**)

面積%レポート

データファイル名 : C:¥Data(HPLC)¥Martin¥Project2¥Rac¥4_63_5 rac AD3 P60 0.5 210.dat
メソッドファイル名 : C:¥Data(HPLC)¥Sasagawa¥Method 120 min.met
ユーザー名 : System
分析日時 : 2017/04/04 9:53:26
印刷日時 : 2017/04/12 11:12:12

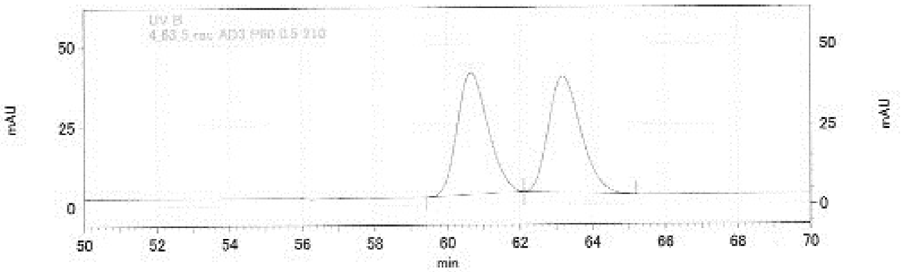

UV_B結果

Pk #	名前	保持時間	面積	面積%	ベースラインコード
1		60.66	2254800	49.925	MM
2		63.19	2261582	50.075	MM
トータル			4516382	100.000	

ページ 1/1

面積%レポート

データファイル名 : C:¥Data(HPLC)¥Martin¥MP-2-123_Product1 AD3 P60 0.5 210.dat
メソッドファイル名 : C:¥Data(HPLC)¥Sasagawa¥Method 120 min.met
ユーザー名 : System
分析日時 : 2016/06/29 15:00:29
印刷日時 : 2017/04/11 15:45:43

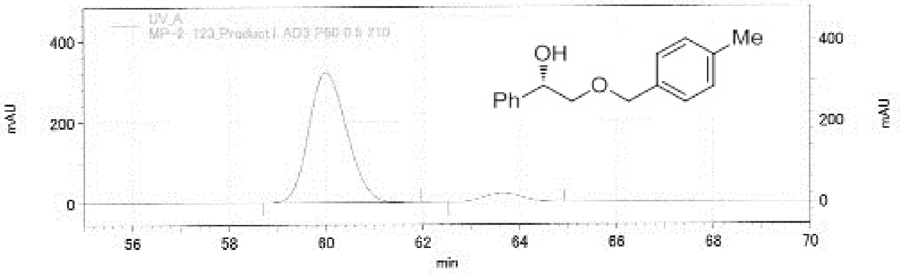

UV_A結果

Pk #	名前	保持時間	面積	面積%	ベースラインコード
1		60.01	17539843	93.640	MM
2		63.63	1191340	6.360	MM
トータル			18731183	100.000	

(*S*)-2-((2-Fluorobenzyl)oxy)-1-phenylethanol (**2ae**)

ページ 1/1

面積%レポート

データファイル名 : C:¥Data(HPLC)¥Martin¥MP-2-149_rac_2 AD3 P60 0.5 210.dat
メソッドファイル名 : C:¥Data(HPLC)¥Sasagawa¥Method 120 min.met
ユーザー名 : System
分析日時 : 2016/07/04 17:04:23
印刷日時 : 2017/04/11 15:43:04

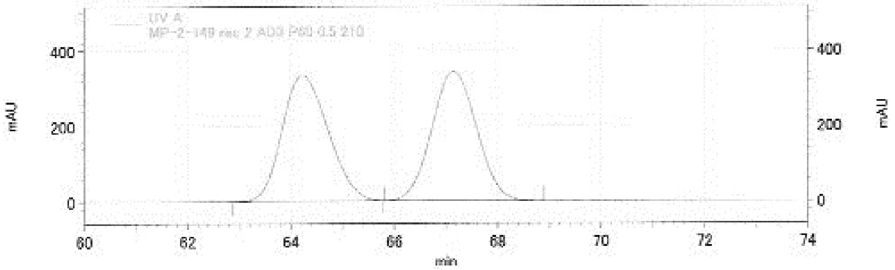

UV_A結果

Pk #	名前	保持時間	面積	面積%	ベースラインコード
1		64.22	20494657	50.030	MM
2		67.16	20469806	49.970	MM
トータル			40964463	100.000	

ページ 1/1

面積%レポート

データファイル名 : C:¥Data(HPLC)¥Martin¥MP-2-149b_1 AD3 P60 0.5 210.dat
メソッドファイル名 : C:¥Data(HPLC)¥Sasagawa¥Method 120 min.met
ユーザー名 : System
分析日時 : 2016/07/13 17:38:20
印刷日時 : 2017/04/11 15:43:08

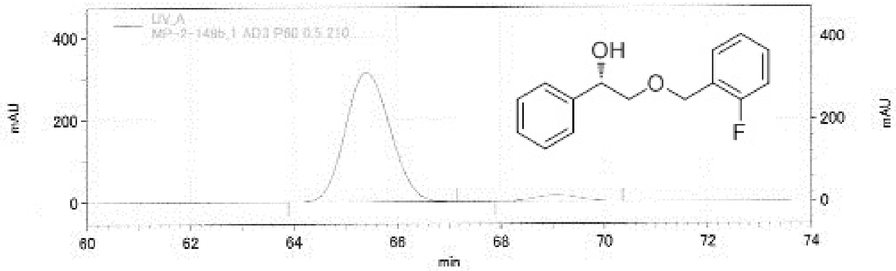

UV_A結果

Pk #	名前	保持時間	面積	面積%	ベースラインコード
1		65.41	19084293	95.178	MM
2		69.06	966943	4.822	MM
トータル			20051236	100.000	

(*S*)-2-((2-Bromobenzyl)oxy)-1-phenylethanol (**2af**)

ページ 1/1

面積%レポート

データファイル名 : C:¥Data(HPLC)¥Martin¥Project2¥Rac¥4_63_12_rac IC3 P60 1.0 210.dat
メソッドファイル名 : C:¥Data(HPLC)¥Sasagawa¥Method 120 min.met
ユーザー名 : System
分析日時 : 2017/04/13 14:30:27
印刷日時 : 2017/04/20 16:43:11

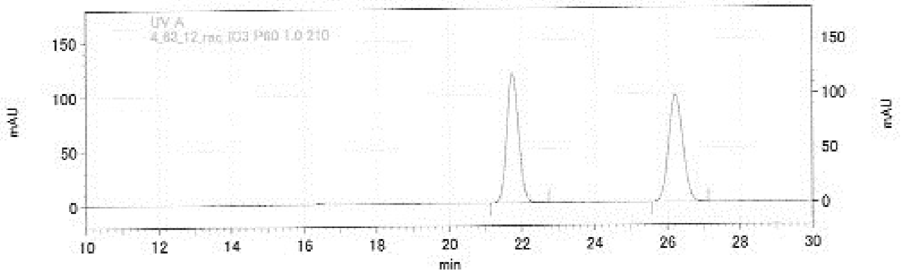

UV_A結果

Pk #	rt	area	area%
1	21.75	2691631	50.012
2	26.23	2690332	49.988
トータル		5381963	100.000

ページ 1/1

面積%レポート

データファイル名 : C:¥Data(HPLC)¥Martin¥MP-4-36_Pro IC3 P60 1.0 210.dat
メソッドファイル名 : C:¥Data(HPLC)¥Sasagawa¥Method 120 min.met
ユーザー名 : System
分析日時 : 2017/01/18 15:03:08
印刷日時 : 2017/04/11 15:57:08

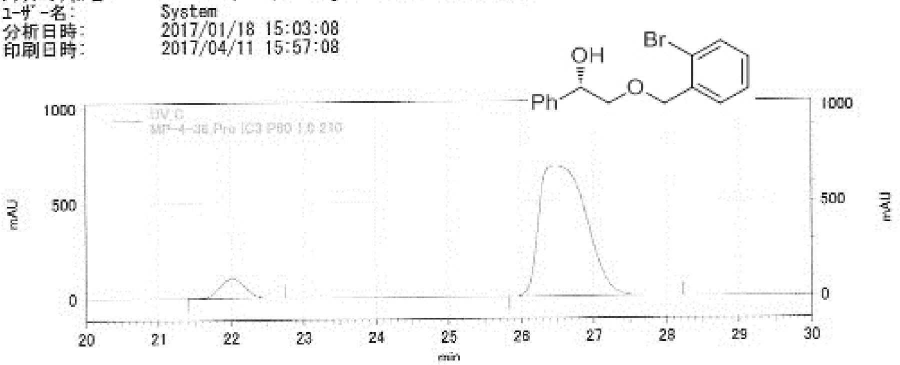

UV_C結果

Pk #	名前	保持時間	面積	面積%	ベースラインコード
1		22.02	2529488	7.601	MM
2		26.48	30749950	92.399	MM
トータル			33279438	100.000	

(*S*)-2-((2-Hydroxy-2-phenylethoxy)methyl)benzonitrile (**2ag**)

面積%レポート

ﾃﾞｰﾀﾌｧｲﾙ名 : C:¥Data(HPLC)¥Martin¥Project2¥Rac¥4_63_11_rac ADH E15 1.0 210.dat
ﾒｿｯﾄﾞﾌｧｲﾙ名 : C:¥Data(HPLC)¥Sasagawa¥Method 120 min.met
ﾕｰｻﾞｰ名 : System
分析日時 : 2017/04/14 12:01:17
印刷日時 : 2017/04/14 13:05:24

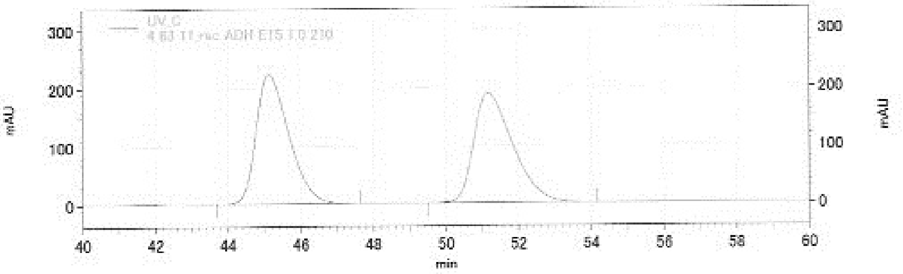

UV_C結果

Pk #	名前	保持時間	面積	面積%	ﾍﾞｰｽﾗｲﾝｺｰﾄﾞ
1		45.14	13935156	49.870	MM
2		51.18	14007674	50.130	MM
ﾄｰﾀﾙ			27942830	100.000	

ﾍﾟｰｼﾞ 1/1

面積%レポート

ﾃﾞｰﾀﾌｧｲﾙ名 : C:¥Data(HPLC)¥Martin¥MP-3-23_Pro ADH E15 1.0 210.dat
ﾒｿｯﾄﾞﾌｧｲﾙ名 : C:¥Data(HPLC)¥Sasagawa¥Method 120 min.met
ﾕｰｻﾞｰ名 : System
分析日時 : 2016/09/16 10:04:49
印刷日時 : 2017/04/11 15:55:41

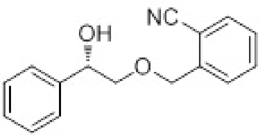

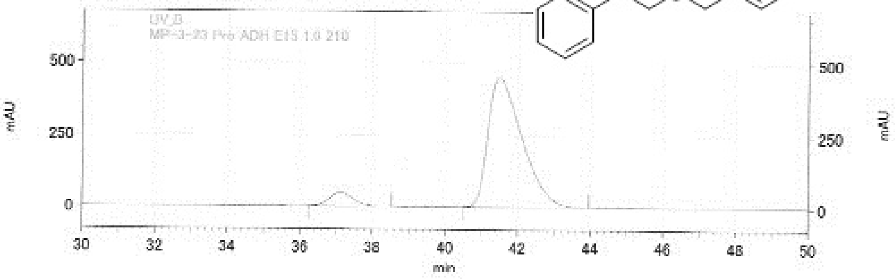

UV_B結果

Pk #	名前	保持時間	面積	面積%	ﾍﾞｰｽﾗｲﾝｺｰﾄﾞ
1		37.12	2115622	6.771	MM
2		41.48	29127622	93.229	MM
ﾄｰﾀﾙ			31243244	100.000	

(*S*)-2-([1,1'-Biphenyl]-4-ylmethoxy)-1-phenylethanol (**2ah**)

面積%レポート

デ゛ータファイル名： C:¥Data(HPLC)¥Martin¥Project2¥Rac¥4_63_7 rac ADH E15 1.5 210.dat
メソッド゛ファイル名： C:¥Data(HPLC)¥Sasagawa¥Method 120 min.met
ユーザ゛ー名： System
分析日時： 2017/04/04 9:50:05
印刷日時： 2017/04/12 11:13:15

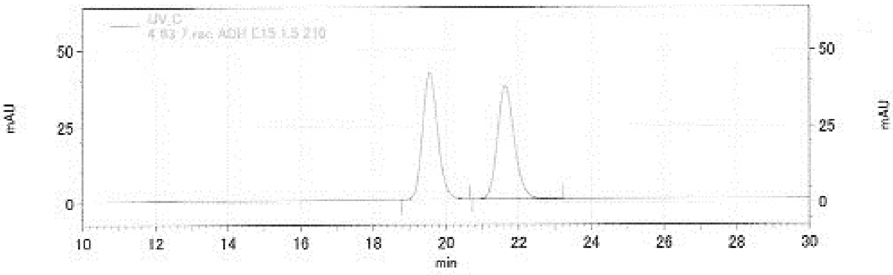

UV_C結果

Pk #	名前	保持時間	面積	面積%	ベ゛ースラインコード゛
1		19.55	1188798	49.809	MM
2		21.63	1197930	50.191	MM
トータル			2386728	100.000	

ペ゛ージ゛ 1/1

面積%レポート

デ゛ータファイル名： C:¥Data(HPLC)¥Martin¥MP-2-156_Pro ADH E15 1.5 210.dat
メソッド゛ファイル名： C:¥Data(HPLC)¥Sasagawa¥Method 120 min.met
ユーザ゛ー名： System
分析日時： 2016/07/12 14:20:37
印刷日時： 2017/04/11 15:49:11

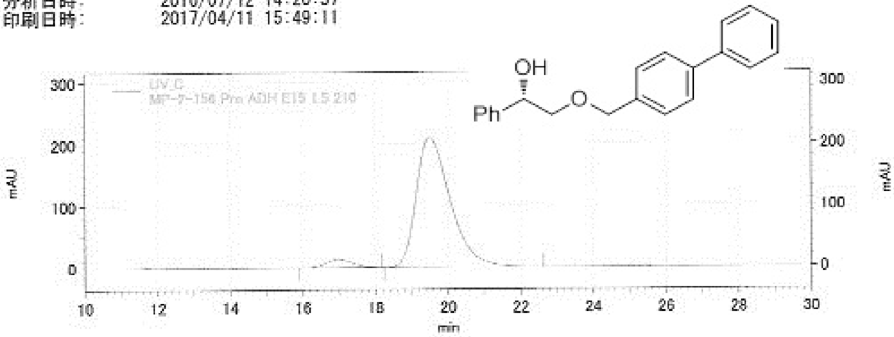

UV_C結果

Pk #	名前	保持時間	面積	面積%	ベ゛ースラインコード゛
1		16.98	656998	4.606	MM
2		19.51	13605868	95.394	MM
トータル			14262866	100.000	

(*S*)-2-((3,5-Bis(trifluoromethyl)benzyl)oxy)-1-phenylethanol (**2ai**)

面積%レポート

ﾃﾞｰﾀﾌｧｲﾙ名 : C:¥Data(HPLC)¥Martin¥Project2¥Rac¥4_63_8_rac AD3 P60 1.0 210.dat
ﾒｿｯﾄﾞﾌｧｲﾙ名 : C:¥Data(HPLC)¥Sasagawa¥Method 120 min.met
ﾕｰｻﾞｰ名 : System
分析日時 : 2017/03/23 11:03:57
印刷日時 : 2017/04/12 11:14:03

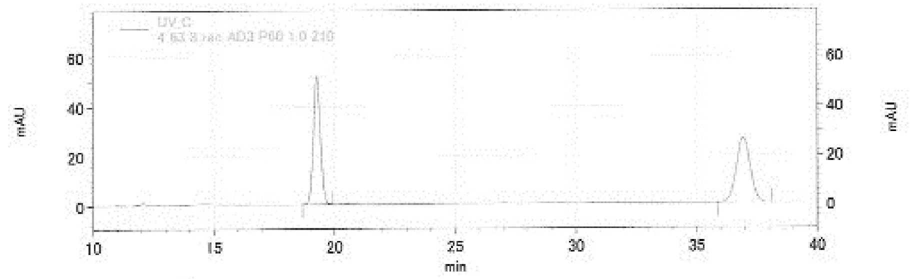

UV_C結果

Pk #	名前	保持時間	面積	面積%	ﾍﾞｰｽﾗｲﾝｺｰﾄﾞ
1		19.27	1046155	49.931	MM
2		36.93	1049065	50.069	MM
ﾄｰﾀﾙ			2095220	100.000	

面積%レポート

ﾃﾞｰﾀﾌｧｲﾙ名 : C:¥Data(HPLC)¥Martin¥MP-2-160_PRO_1 AD3 P60 1.0 210.dat
ﾒｿｯﾄﾞﾌｧｲﾙ名 : C:¥Data(HPLC)¥Sasagawa¥Method 120 min.met
ﾕｰｻﾞｰ名 : System
分析日時 : 2016/07/14 11:12:47
印刷日時 : 2017/04/11 15:50:14

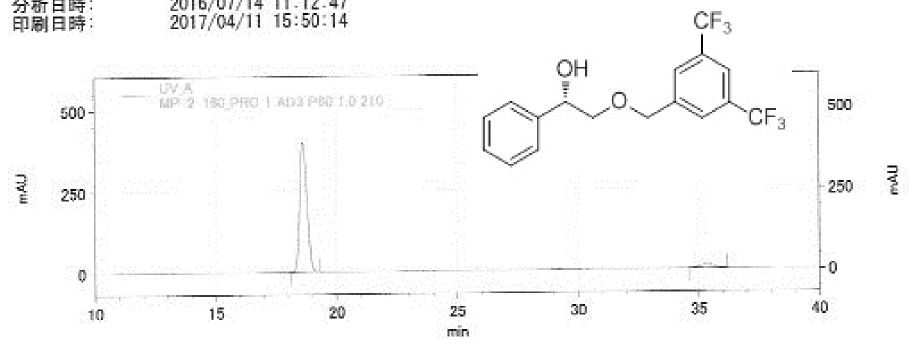

UV_A結果

Pk #	名前	保持時間	面積	面積%	ﾍﾞｰｽﾗｲﾝｺｰﾄﾞ
1		18.62	8388950	94.459	MM
2		35.35	492104	5.541	MM
ﾄｰﾀﾙ			8881054	100.000	

(*S*)-2-(Naphthalen-2-ylmethoxy)-1-phenylethanol (**2aj**)

面積%レポート

データファイル名 : C:¥Data(HPLC)¥Martin¥Project2¥Rac¥4_63_4_rac ADH E50 1.5 210.dat
メソッドファイル名 : C:¥Data(HPLC)¥Sasagawa¥Method 120 min.met
ユーザー名 : System
分析日時 : 2017/04/04 12:44:45
印刷日時 : 2017/04/12 11:10:51

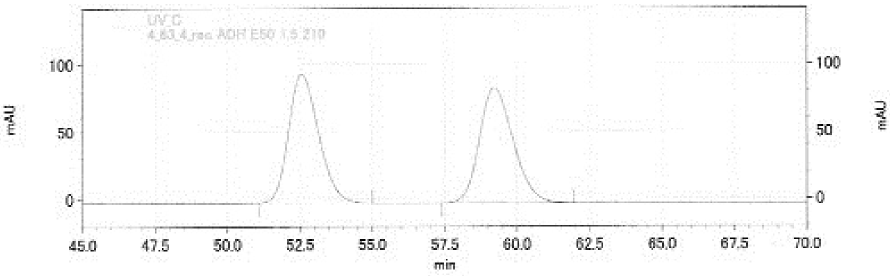

UV_C結果

Pk #	名前	保持時間	面積	面積%	ベースラインコード
1		52.57	6911334	49.882	MM
2		59.21	6944168	50.118	MM
トータル			13855502	100.000	

ページ 1/1

面積%レポート

データファイル名 : C:¥Data(HPLC)¥Martin¥MP-2-154_Pro ADH E50 1.5 210.dat
メソッドファイル名 : C:¥Data(HPLC)¥Sasagawa¥Method 120 min.met
ユーザー名 : System
分析日時 : 2016/07/15 13:04:20
印刷日時 : 2017/04/11 15:44:38

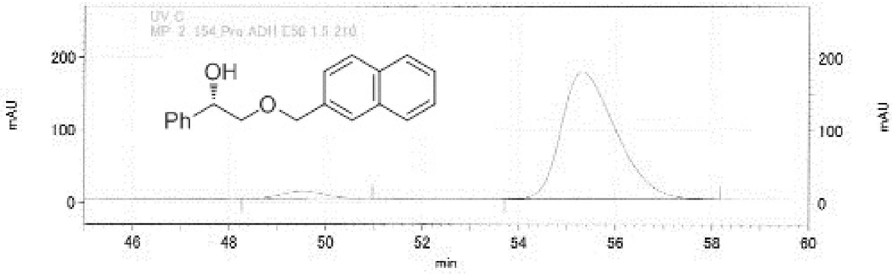

UV_C結果

Pk #	名前	保持時間	面積	面積%	ベースラインコード
1		49.53	642167	4.469	MM
2		55.32	13728255	95.531	MM
トータル			14370422	100.000	

(*S*)-2-((3-Methylbut-2-en-1-yl)oxy)-1-phenylethanol (**2ak**)

面積%レポート

ﾃﾞｰﾀﾌｧｲﾙ名 : C:¥Data(HPLC)¥Martin¥Project2¥Rac¥4_63_13_rac AD3 P60 1.0 210.dat
ﾒｿｯﾄﾞﾌｧｲﾙ名 : C:¥Data(HPLC)¥Sasagawa¥Method 120 min.met
ﾕｰｻﾞｰ名 : System
分析日時 : 2017/03/24 10:15:53
印刷日時 : 2017/04/12 11:22:35

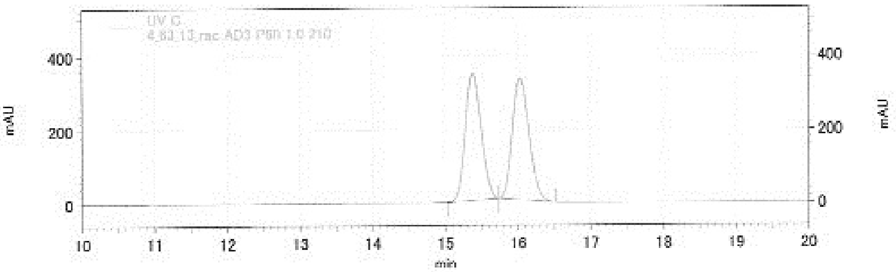

UV_C結果

Pk #	名前	保持時間	面積	面積%	ﾍﾞｰｽﾗｲﾝｺｰﾄﾞ
1		15.38	5258531	49.917	MM
2		16.03	5275937	50.083	MM
ﾄｰﾀﾙ			10534468	100.000	

面積%レポート

ﾍﾟｰｼﾞ 1/1

ﾃﾞｰﾀﾌｧｲﾙ名 : C:¥Data(HPLC)¥Martin¥MP-4-34_Pro AD3 P60 1.0 210.dat
ﾒｿｯﾄﾞﾌｧｲﾙ名 : C:¥Data(HPLC)¥Sasagawa¥Method 120 min.met
ﾕｰｻﾞｰ名 : System
分析日時 : 2017/01/18 17:08:28
印刷日時 : 2017/04/11 15:58:22

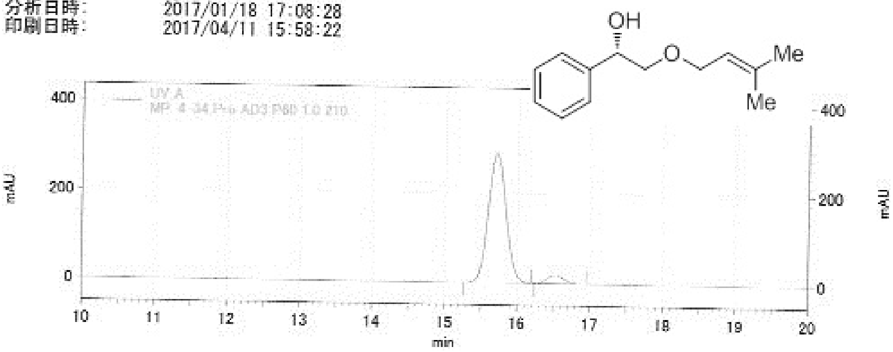

UV_A結果

Pk #	名前	保持時間	面積	面積%	ﾍﾞｰｽﾗｲﾝｺｰﾄﾞ
1		15.70	4906448	94.283	MM
2		16.52	297531	5.717	MM
ﾄｰﾀﾙ			5203979	100.000	

(*S*)-2-(Cinnamyloxy)-1-phenylethanol (**2al**)

ﾃﾞｰﾀﾌｧｲﾙ名 : C:¥Data(HPLC)¥Martin¥Project2¥Rac¥4_63_14_rac IC3 P60 1.0 210.dat
ﾒｿｯﾄﾞﾌｧｲﾙ名 : C:¥Data(HPLC)¥Sasagawa¥Method 120 min.met
ﾕｰｻﾞｰ名 : System
分析日時 : 2017/04/03 18:25:09
印刷日時 : 2017/04/12 11:24:05

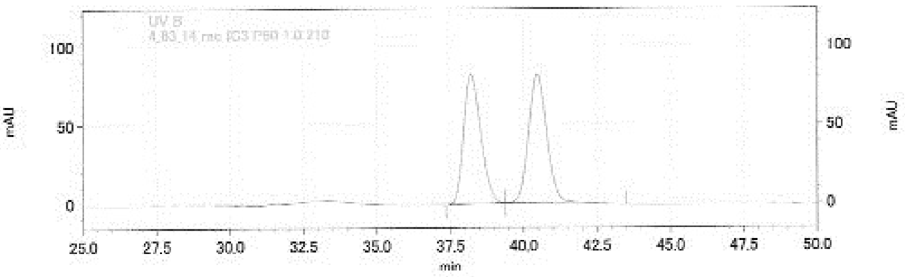

UV_B結果

Pk #	名前	保持時間	面積	面積%	ﾍﾞｰｽﾗｲﾝｺｰﾄﾞ
1		38.22	3349755	48.296	MM
2		40.47	3586148	51.704	MM
ﾄｰﾀﾙ			6935903	100.000	

ﾍﾟｰｼﾞ 1/1

面積%レポート

ﾃﾞｰﾀﾌｧｲﾙ名 : C:¥Data(HPLC)¥Martin¥4_46B_pro IC3 P60 1.0 210.dat
ﾒｿｯﾄﾞﾌｧｲﾙ名 : C:¥Data(HPLC)¥Sasagawa¥Method 120 min.met
ﾕｰｻﾞｰ名 : System
分析日時 : 2017/04/13 15:22:48
印刷日時 : 2017/04/13 16:14:28

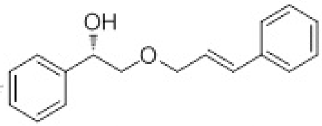

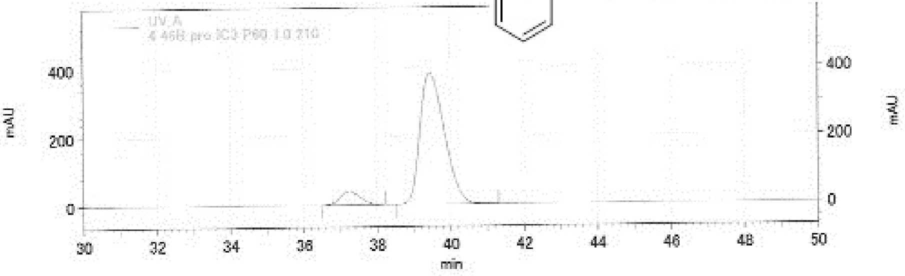

UV_A結果

Pk #	名前	保持時間	面積	面積%	ﾍﾞｰｽﾗｲﾝｺｰﾄﾞ
1		37.25	1533102	7.974	MM
2		39.44	17692775	92.026	MM
ﾄｰﾀﾙ			19225877	100.000	

(*S*,*E*)-Methyl 4-(2-hydroxy-2-phenylethoxy)but-2-enoate (**2am**)

データファイル名 : C:¥Data(HPLC)¥Martin¥Project2¥Rac¥3_63_15_rac2 OD3 P20 1.0 210.dat
メソッドファイル名: C:¥Data(HPLC)¥Sasagawa¥Method 120 min.met
ユーザー名: System
分析日時: 2017/04/14 12:18:54
印刷日時: 2017/04/14 13:32:25

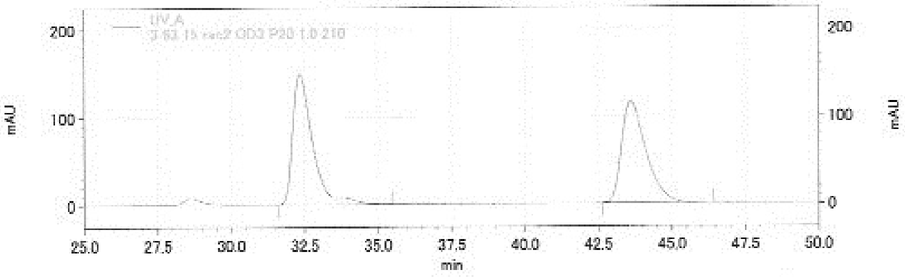

UV_A結果

Pk #	名前	保持時間	面積	面積%	ベースラインコード
1		32.34	6994526	51.003	MM
2		43.61	6719343	48.997	MM
トータル			13713869	100.000	

ページ 1/1

面積%レポート

データファイル名 : C:¥Data(HPLC)¥Martin¥MP-4-45_Pro OD3 P20 1.0 210.dat
メソッドファイル名: C:¥Data(HPLC)¥Sasagawa¥Method 120 min.met
ユーザー名: System
分析日時: 2017/01/28 11:54:13
印刷日時: 2017/04/14 13:32:51

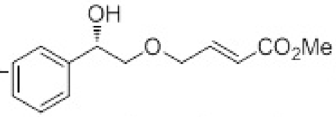

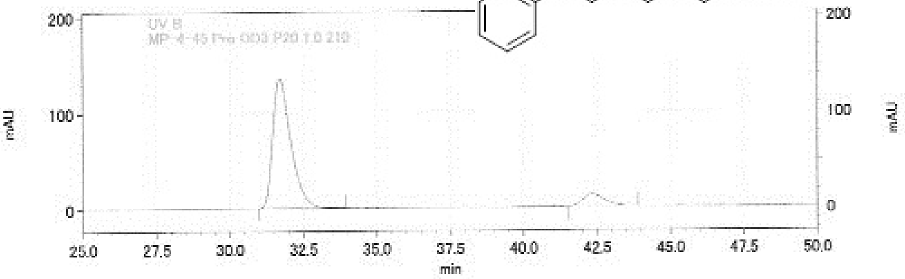

UV_B結果

Pk #	名前	保持時間	面積	面積%	ベースラインコード
1		31.71	5794714	90.043	MM
2		42.35	640764	9.957	MM
トータル			6435478	100.000	

(*S*)-1-Phenyl-2-((3-phenylprop-2-yn-1-yl)oxy)ethanol (**2an**)

面積%レポート

デ－タファイル名： C:¥Data(HPLC)¥Martin¥Project2¥Rac¥4_63_16_Pro_rac2 AD3 P60 1.0 210.dat
メソッドファイル名： C:¥Data(HPLC)¥Sasagawa¥Method 120 min.met
ユ－ザ－名： System
分析日時： 2017/03/24 10:14:09
印刷日時： 2017/04/12 11:32:33

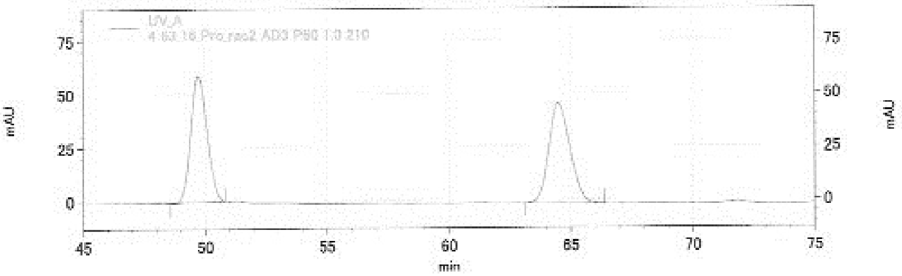

UV_A結果

Pk #	名前	保持時間	面積	面積%	ベ－スラインコ－ド
1		49.73	2741163	49.244	MM
2		64.49	2825272	50.756	MM
ト－タル			5566435	100.000	

ペ－ジ 1/1

面積%レポート

デ－タファイル名： C:¥Data(HPLC)¥Martin¥4_43_Pro_ AD3 P60 1.0 210.dat
メソッドファイル名： C:¥Data(HPLC)¥Sasagawa¥Method 120 min.met
ユ－ザ－名： System
分析日時： 2017/04/13 13:43:28
印刷日時： 2017/04/13 15:04:39

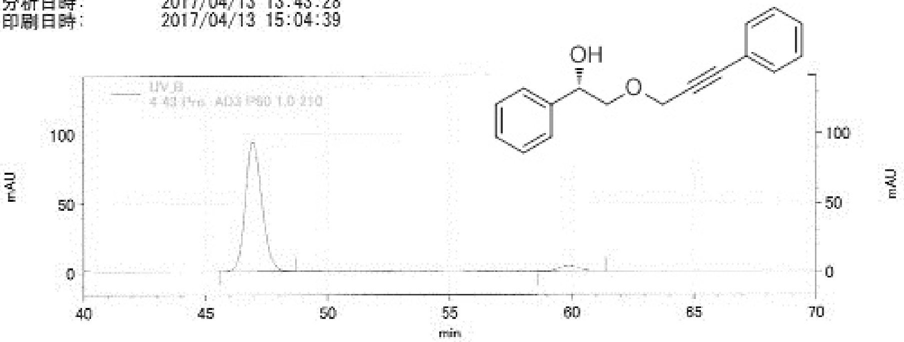

UV_B結果

Pk #	名前	保持時間	面積	面積%	ベ－スラインコ－ド
1		46.94	4378801	94.951	MM
2		59.89	232845	5.049	MM
ト－タル			4611646	100.000	

(*S*)-2-(Benzyloxy)-1-(*o*-tolyl)ethanol (**2ba**)

面積%レポート

データファイル名 : C:¥Data(HPLC)¥Martin¥Project2¥Rac¥4_64_1_rac AD3 P60 1.0 210.dat
メソッドファイル名 : C:¥Data(HPLC)¥Sasagawa¥Method 120 min.met
ユーザー名 : System
分析日時 : 2017/04/12 16:01:30
印刷日時 : 2017/04/18 18:51:28

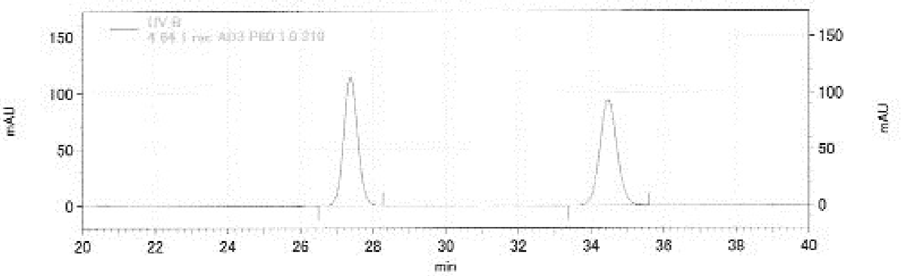

UV_B結果

Pk #	名前	保持時間	面積	面積%	ベースラインコード
1		27.39	3067873	49.636	MM
2		34.48	3112821	50.364	MM
トータル			6180694	100.000	

ページ 1/1

面積%レポート

データファイル名 : C:¥Data(HPLC)¥Martin¥MP-3-119_Pro AD3 P60 1.0 210.dat
メソッドファイル名 : C:¥Data(HPLC)¥Sasagawa¥Method 120 min.met
ユーザー名 : System
分析日時 : 2016/11/16 13:08:26
印刷日時 : 2017/04/12 11:41:29

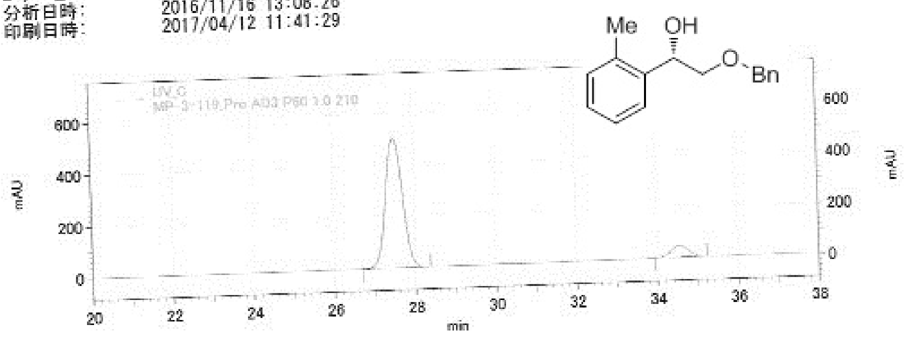

UV_C結果

Pk #	名前	保持時間	面積	面積%	ベースラインコード
1		27.51	14706756	91.501	MM
2		34.54	1365989	8.499	MM
トータル			16072745	100.000	

(*S*)-2-(Benzyloxy)-1-(*m*-tolyl)ethanol (**2ca**)

[illegible]

デ－タファイル名： C:¥Data(HPLC)¥Martin¥Project2¥Rac¥4_64_2_rac IE P60 1.5 210.dat
メソッドファイル名： C:¥Data(HPLC)¥Sasagawa¥Method 120 min.met
ユ－ザ－名： System
分析日時： 2017/04/03 16:42:56
印刷日時： 2017/04/12 11:43:11

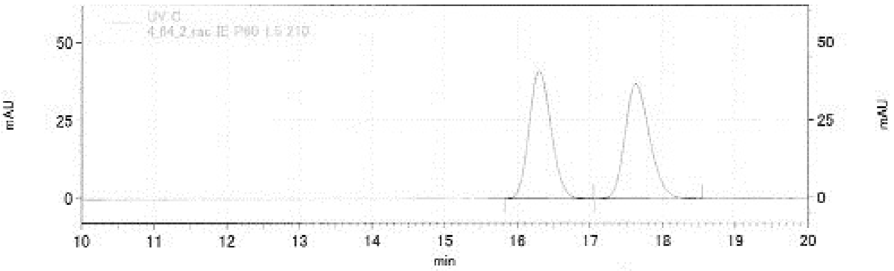

UV_C結果

Pk #	名前	保持時間	面積	面積%	ベースラインコード
1		16.30	866720	49.835	MM
2		17.63	872467	50.165	MM
トータル			1739187	100.000	

面積%レポート

ページ 1/1

デ－タファイル名： C:¥Data(HPLC)¥Martin¥MP-3-118_Pro IE P60 1.5 210.dat
メソッドファイル名： C:¥Data(HPLC)¥Sasagawa¥Method 120 min.met
ユ－ザ－名： System
分析日時： 2016/11/16 16:35:21
印刷日時： 2017/04/11 16:09:39

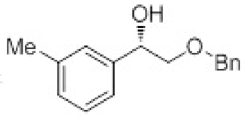

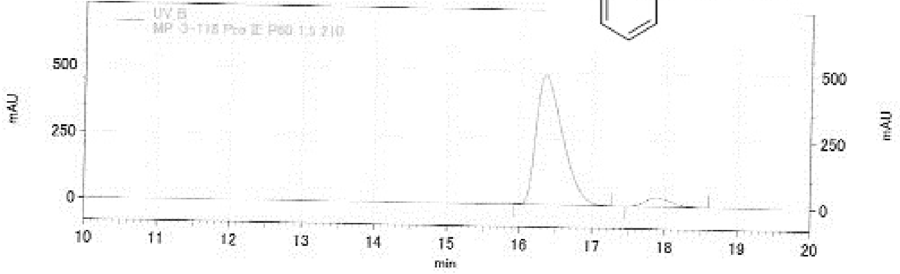

UV_B結果

Pk #	名前	保持時間	面積	面積%	ベースラインコード
1		16.34	12335509	93.638	MM
2		17.89	838109	6.362	MM
トータル			13173618	100.000	

(*S*)-2-(Benzyloxy)-1-(*p*-tolyl)ethanol (**2da**)

面積%レポート

データファイル名 : C:¥Data(HPLC)¥Martin¥Project2¥Rac¥4_64_3_rac AD3 P60 0.5 210.dat
メソッドファイル名: C:¥Data(HPLC)¥Sasagawa¥Method 120 min.met
ユーザー名: System
分析日時: 2017/03/23 17:54:16
印刷日時: 2017/04/12 11:46:38

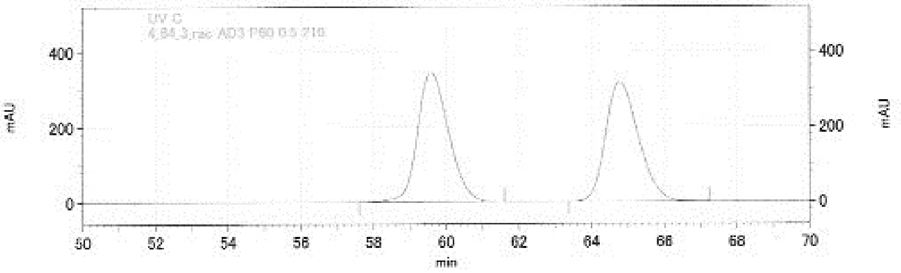

UV C結果

Pk #	名前	保持時間	面積	面積%	ベースラインコード
1		59.59	20496026	50.622	MM
2		64.78	19992457	49.378	MM
トータル			40488483	100.000	

ページ 1/1

面積%レポート

データファイル名 : C:¥Data(HPLC)¥Martin¥MP-3-71_PorA AD3 P60 0.50 210.dat
メソッドファイル名: C:¥Data(HPLC)¥Sasagawa¥Method 120 min.met
ユーザー名: System
分析日時: 2016/10/19 14:21:59
印刷日時: 2017/04/12 11:46:42

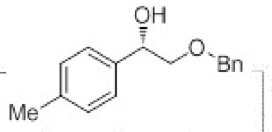

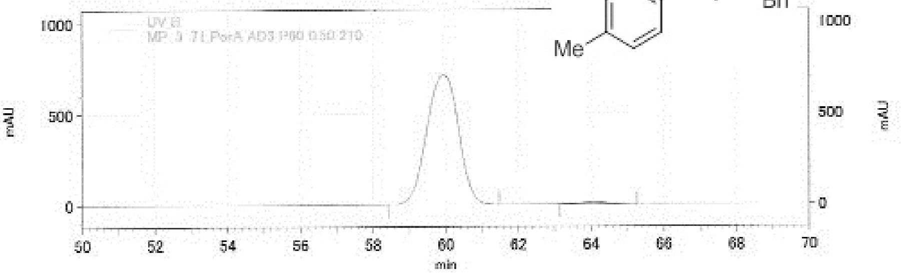

UV B結果

Pk #	名前	保持時間	面積	面積%	ベースラインコード
1		59.96	43156160	98.693	MM
2		64.09	571423	1.307	MM
トータル			43727583	100.000	

(*S*)-2-(Benzyloxy)-1-(2-fluorophenyl)ethanol (**2ea**)

ﾍﾟｰｼﾞ 1/1

面積%レポート

ﾃﾞｰﾀﾌｧｲﾙ名 : C:¥Data(HPLC)¥Martin¥Project2¥Rac¥4_64_4 rac AD3 P60 1.0 210.dat
ﾒｿｯﾄﾞﾌｧｲﾙ名 : C:¥Data(HPLC)¥Sasagawa¥Method 120 min.met
ﾕｰｻﾞｰ名 : System
分析日時 : 2017/04/12 16:50:10
印刷日時 : 2017/04/12 17:32:43

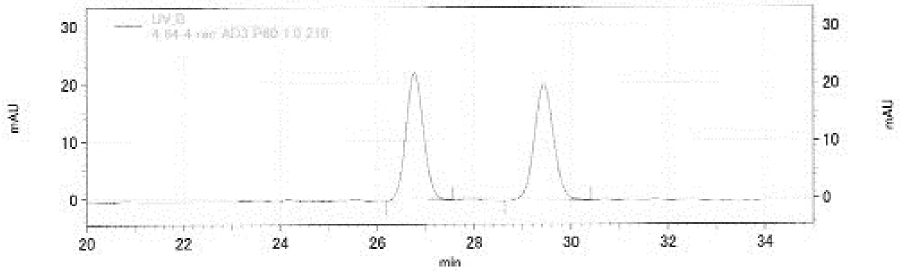

UV_B結果

Pk #	名前	保持時間	面積	面積%	ﾍﾞｰｽﾗｲﾝｺｰﾄﾞ
1		26.77	561138	49.680	MM
2		29.44	568376	50.320	MM
ﾄｰﾀﾙ			1129514	100.000	

ﾍﾟｰｼﾞ 1/1

面積%レポート

ﾃﾞｰﾀﾌｧｲﾙ名 : C:¥Data(HPLC)¥Martin¥MP-4-4_Pro AD3 P60 1.0 210.dat
ﾒｿｯﾄﾞﾌｧｲﾙ名 : C:¥Data(HPLC)¥Sasagawa¥Method 120 min.met
ﾕｰｻﾞｰ名 : System
分析日時 : 2016/12/23 10:35:38
印刷日時 : 2017/04/11 16:10:56

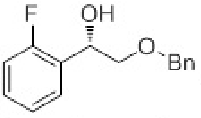

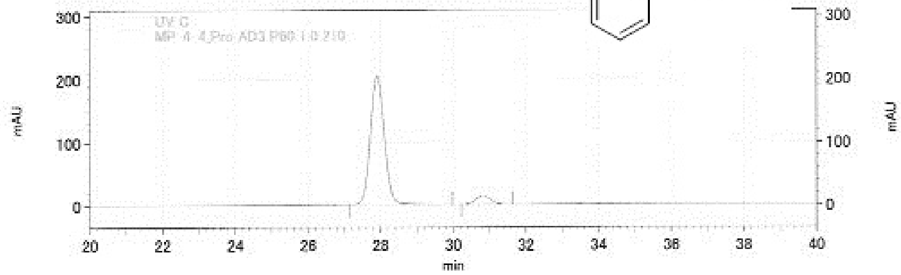

UV_C結果

Pk #	名前	保持時間	面積	面積%	ﾍﾞｰｽﾗｲﾝｺｰﾄﾞ
1		27.91	5391382	93.320	MM
2		30.81	385941	6.680	MM
ﾄｰﾀﾙ			5777323	100.000	

(*S*)-2-(Benzyloxy)-1-(3-fluorophenyl)ethanol (**2fa**)

面積%レポート

データファイル名： C:¥Data(HPLC)¥Martin¥Project2¥Rac¥4_64_5_rac AD3 P60 1.0 210.dat
メソッドファイル名： C:¥Data(HPLC)¥Sasagawa¥Method 120 min.met
ユーザー名： System
分析日時： 2017/03/22 16:25:10
印刷日時： 2017/04/12 11:51:13

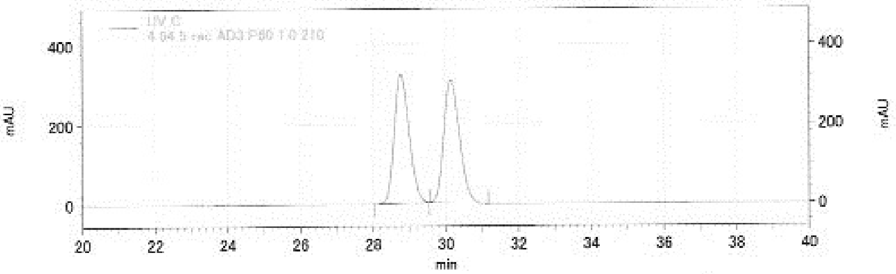

UV_C結果

Pk #	名前	保持時間	面積	面積%	ベースラインコード
1		28.77	9266887	49.939	MM
2		30.14	9289667	50.061	MM
トータル			18556554	100.000	

ページ 1/1

面積%レポート

データファイル名： C:¥Data(HPLC)¥Martin¥MP-3-194_Pro AD3 P60 1.0 210.dat
メソッドファイル名： C:¥Data(HPLC)¥Sasagawa¥Method 120 min.met
ユーザー名： System
分析日時： 2016/12/16 10:40:52
印刷日時： 2017/04/11 16:13:02

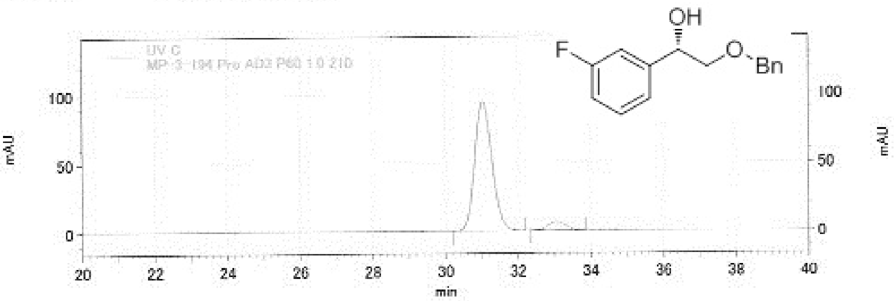

UV_C結果

Pk #	名前	保持時間	面積	面積%	ベースラインコード
1		31.03	3110000	93.461	MM
2		33.06	217599	6.539	MM
トータル			3327599	100.000	

(*S*)-2-(Benzyloxy)-1-(4-fluorophenyl)ethanol (**2ga**)

面積%レポート

デ゛ータファイル名： C:¥Data(HPLC)¥Martin¥Project2¥Rac¥4_64_6_rac AD3 P60 0.5 210.dat
メソット゛ファイル名： C:¥Data(HPLC)¥Sasagawa¥Method 120 min.met
ユーザ゛ー名： System
分析日時： 2017/03/23 12:31:20
印刷日時： 2017/04/12 11:52:34

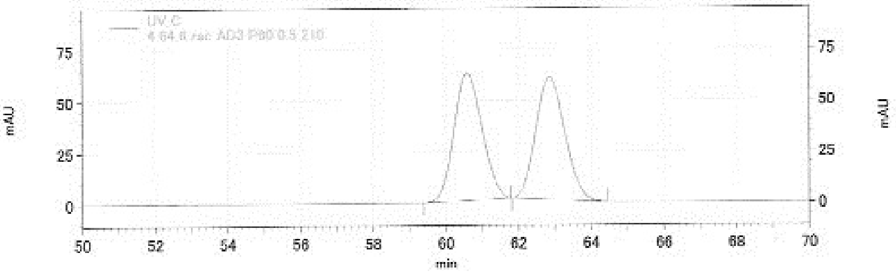

UV_C結果

Pk #	名前	保持時間	面積	面積%	ベ゛ースラインコード゛
1		60.59	3319869	50.008	MM
2		62.85	3318827	49.992	MM
トータル			6638696	100.000	

ペ゛ージ゛ 1/1

面積%レポート

デ゛ータファイル名： C:¥Data(HPLC)¥Martin¥MP-3-176_Pro AD3 P60 0.25 210.dat
メソット゛ファイル名： C:¥Data(HPLC)¥Sasagawa¥Method 120 min.met
ユーザ゛ー名： System
分析日時： 2016/12/13 11:46:23
印刷日時： 2017/04/12 11:54:32

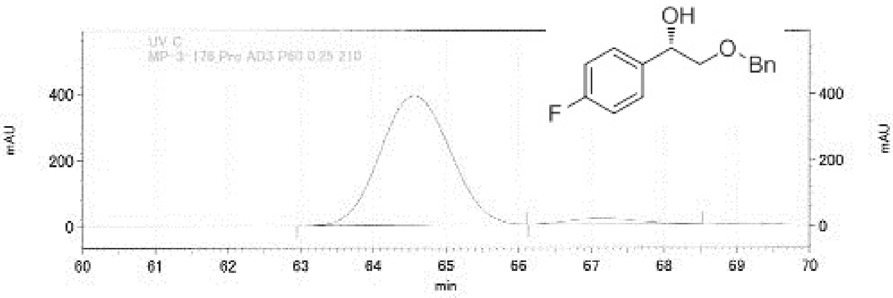

UV_C結果

Pk #	名前	保持時間	面積	面積%	ベ゛ースラインコード゛
1		64.57	26135266	95.592	MM
2		67.12	1205304	4.408	MM
トータル			27340570	100.000	

(*S*)-2-(Benzyloxy)-1-(4-bromophenyl)ethanol (**2ha**)

ﾍﾟｰｼﾞ 1/1

面積%レポート

ﾃﾞｰﾀﾌｧｲﾙ名 : C:¥Data(HPLC)¥Martin¥Project2¥Rac¥4_64_10_rac AD3 P60 1.0 210.dat
ﾒｿｯﾄﾞﾌｧｲﾙ名: C:¥Data(HPLC)¥Sasagawa¥Method 120 min.met
ﾕｰｻﾞｰ名: System
分析日時: 2017/04/12 17:42:30
印刷日時: 2017/04/12 18:30:43

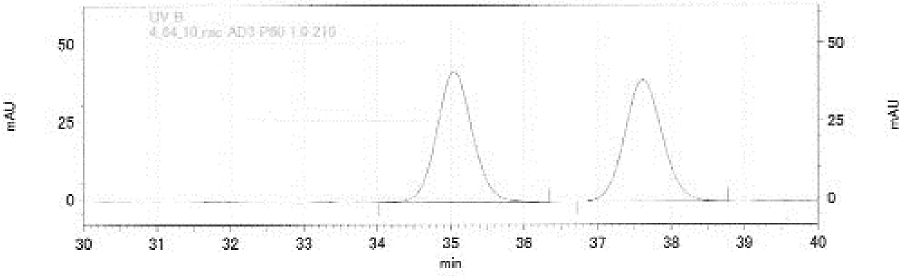

UV_B結果

Pk #	名前	保持時間	面積	面積%	ﾍﾞｰｽﾗｲﾝｺｰﾄﾞ
1		35.04	1418195	50.422	MM
2		37.61	1394431	49.578	MM
ﾄｰﾀﾙ			2812626	100.000	

ﾍﾟｰｼﾞ 1/1

面積%レポート

ﾃﾞｰﾀﾌｧｲﾙ名 : C:¥Data(HPLC)¥Martin¥MP-3-165Pro AD3 P60 1.00 210.dat
ﾒｿｯﾄﾞﾌｧｲﾙ名: C:¥Data(HPLC)¥Sasagawa¥Method 120 min.met
ﾕｰｻﾞｰ名: System
分析日時: 2016/12/14 14:57:38
印刷日時: 2017/04/11 16:18:16

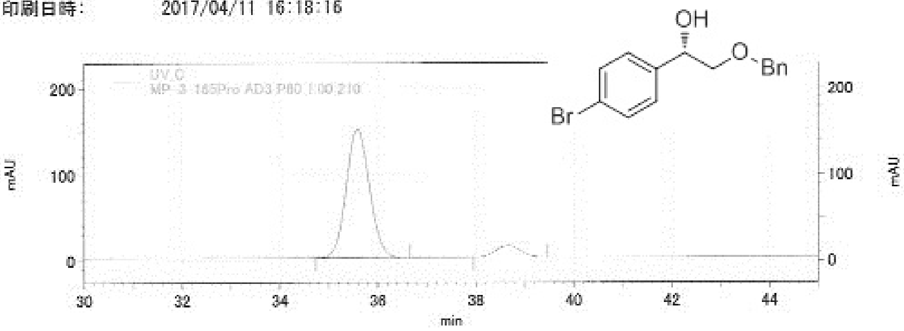

UV_C結果

Pk #	名前	保持時間	面積	面積%	ﾍﾞｰｽﾗｲﾝｺｰﾄﾞ
1		35.58	4934510	91.089	MM
2		38.67	482746	8.911	MM
ﾄｰﾀﾙ			5417256	100.000	

(*S*)-2-(Benzyloxy)-1-(4-methoxyphenyl)ethanol (**2ia**)

面積%レポート

データファイル名 : C:¥Data(HPLC)¥Martin¥Project2¥Rac¥4_64_7_rac AD3 P60 1.0 210.dat
メソッドファイル名 : C:¥Data(HPLC)¥Sasagawa¥Method 120 min.met
ユーザー名 : System
分析日時 : 2017/03/24 17:45:00
印刷日時 : 2017/04/12 11:57:50

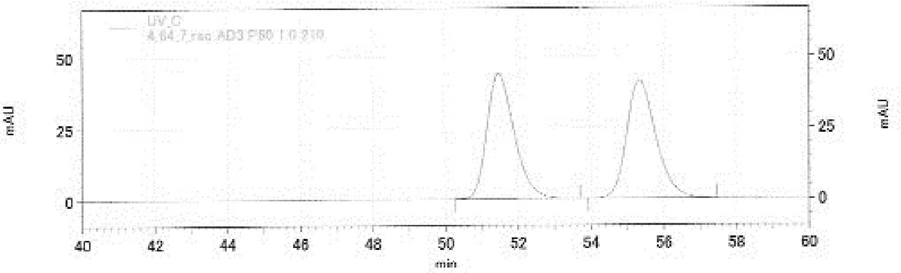

UV_C結果

Pk #	名前	保持時間	面積	面積%	ベースラインコード
1		51.47	2295082	50.039	MM
2		55.34	2291509	49.961	MM
トータル			4586591	100.000	

ページ 1/1

面積%レポート

データファイル名 : C:¥Data(HPLC)¥Martin¥3_112_Pro_ AD3 P60 1.0 210.dat
メソッドファイル名 : C:¥Data(HPLC)¥Sasagawa¥Method 120 min.met
ユーザー名 : System
分析日時 : 2017/04/13 10:33:38
印刷日時 : 2017/04/13 11:54:25

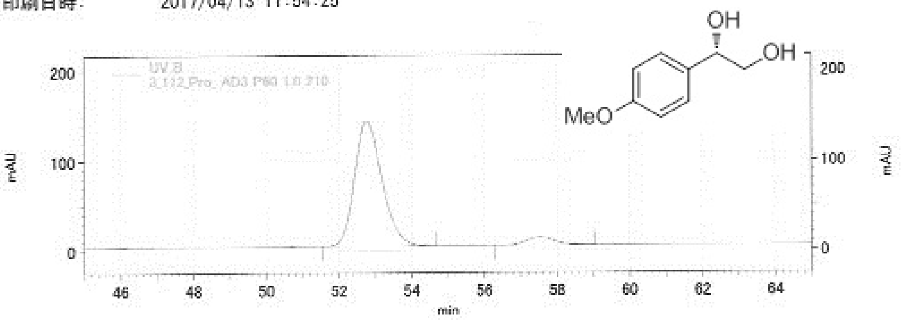

UV_B結果

Pk #	名前	保持時間	面積	面積%	ベースラインコード
1		52.77	7314813	93.835	MM
2		57.52	480559	6.165	MM
トータル			7795372	100.000	

(*S*)-2-(Benzyloxy)-1-cyclohexylethanol (**2ja**)

面積%レポート

デ゛ータファイル名 : C:¥Data(HPLC)¥Martin¥Project2¥Rac¥_64_13_rac AD3 P60 1.0 210.dat
メソット゛ファイル名 : C:¥Data(HPLC)¥Sasagawa¥Method 120 min.met
ユーザ゛ー名 : System
分析日時 : 2017/04/12 14:01:16
印刷日時 : 2017/04/12 15:57:05

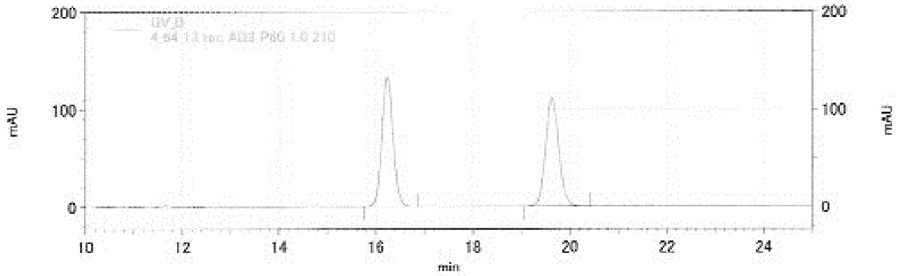

UV_B結果

Pk #	名前	保持時間	面積	面積%	ベ゛ースラインコート゛
1		16.23	2125825	50.115	MM
2		19.63	2116105	49.885	MM
トータル			4241930	100.000	

ペ゛ージ゛ 1/1

面積%レポート

デ゛ータファイル名 : C:¥Data(HPLC)¥Martin¥MP-4-55_Pro AD3 P60 1.0 210.dat
メソット゛ファイル名 : C:¥Data(HPLC)¥Sasagawa¥Method 120 min.met
ユーザ゛ー名 : System
分析日時 : 2017/01/31 15:11:51
印刷日時 : 2017/04/11 16:22:52

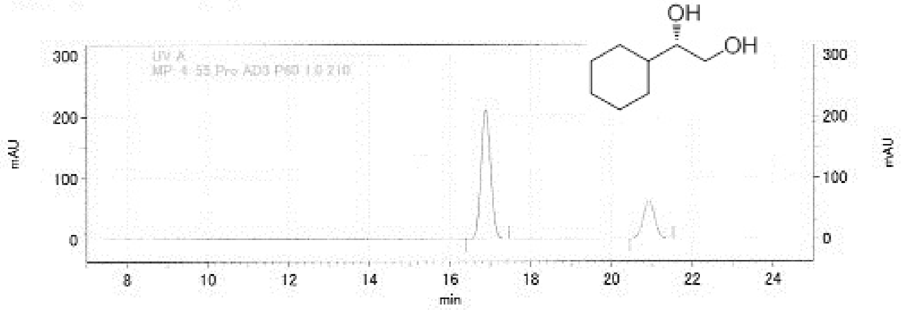

UV_A結果

Pk #	名前	保持時間	面積	面積%	ベ゛ースラインコート゛
1		16.88	3477531	74.033	MM
2		20.93	1219754	25.967	MM
トータル			4697285	100.000	

(*S*)-1-(Benzyloxy)-2-phenylpropan-2-ol (**13a**)

面積%レポート

ﾃﾞｰﾀﾌｧｲﾙ名 : C:¥Data(HPLC)¥Martin¥4_65_1_rac AD3 P60 1.0 210.dat
ﾒｿｯﾄﾞﾌｧｲﾙ名 : C:¥Data(HPLC)¥Sasagawa¥Method 120 min.met
ﾕｰｻﾞｰ名 : System
分析日時 : 2017/04/11 16:31:38
印刷日時 : 2017/04/12 11:38:41

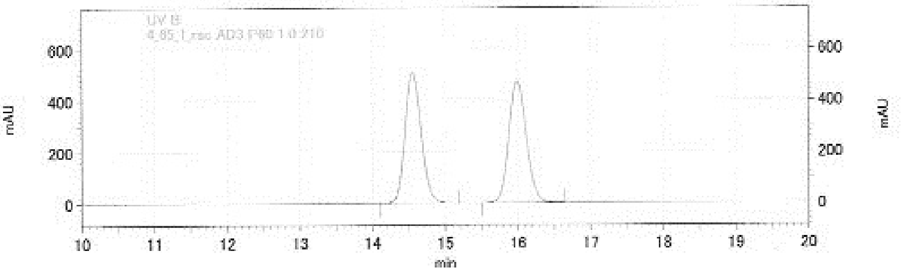

UV_B結果

Pk #	名前	保持時間	面積	面積%	ﾍﾞｰｽﾗｲﾝｺｰﾄﾞ
1		14.55	7840236	49.811	MM
2		15.99	7899611	50.189	MM
ﾄｰﾀﾙ			15739847	100.000	

ﾍﾟｰｼﾞ 1/1

面積%レポート

ﾃﾞｰﾀﾌｧｲﾙ名 : C:¥Data(HPLC)¥Martin¥MP-3-143_Pro AD3 P60 1.0 210.dat
ﾒｿｯﾄﾞﾌｧｲﾙ名 : C:¥Data(HPLC)¥Sasagawa¥Method 120 min.met
ﾕｰｻﾞｰ名 : System
分析日時 : 2016/11/22 9:12:36
印刷日時 : 2017/04/11 16:23:49

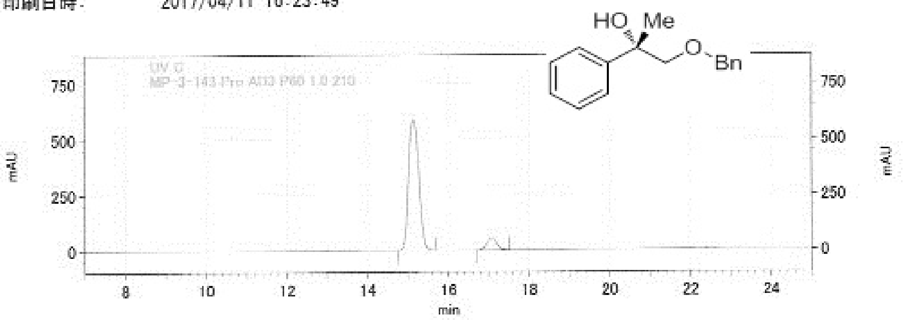

UV_C結果

Pk #	名前	保持時間	面積	面積%	ﾍﾞｰｽﾗｲﾝｺｰﾄﾞ
1		15.14	10370932	92.040	MM
2		17.08	896899	7.960	MM
ﾄｰﾀﾙ			11267831	100.000	

(*S*)-1-(Benzyloxy)-2-(*o*-tolyl)propan-2-ol (**13b**)

面積%レポート

データファイル名 : C:¥Data(HPLC)¥Martin¥Project2¥Rac¥4_65_2_rac AD3 P60 1.0 210.dat
メソッドファイル名 : C:¥Data(HPLC)¥Sasagawa¥Method 120 min.met
ユーザー名 : System
分析日時 : 2017/04/11 16:59:18
印刷日時 : 2017/04/20 16:57:42

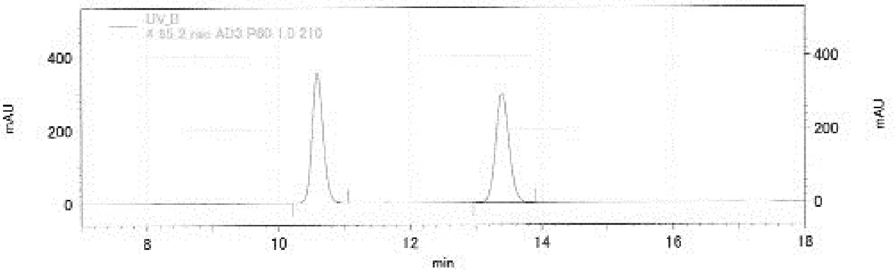

UV_B結果

Pk #	rt	area	area%
1	10.59	4136771	49.725
2	13.40	4182482	50.275
トータル		8319253	100.000

ページ 1/1

面積%レポート

データファイル名 : C:¥Data(HPLC)¥Martin¥MP-3-150_Pro AD3 P60 1.0 210.dat
メソッドファイル名 : C:¥Data(HPLC)¥Sasagawa¥Method 120 min.met
ユーザー名 : System
分析日時 : 2016/11/26 12:23:45
印刷日時 : 2017/04/12 12:14:19

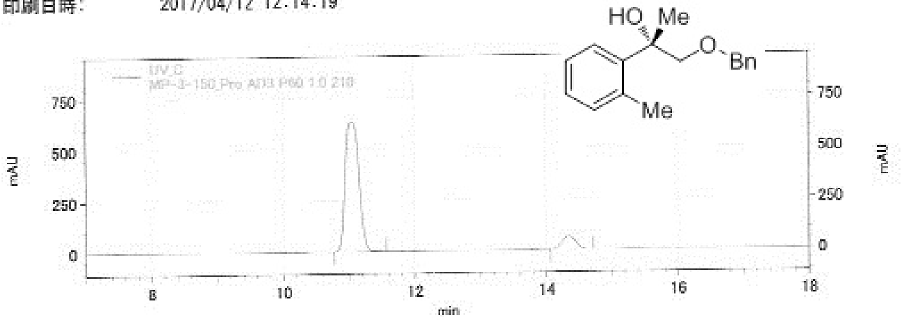

UV_C結果

Pk #	名前	保持時間	面積	面積%	ベースラインコード
1		11.05	9223158	91.007	MM
2		14.34	911414	8.993	MM
トータル			10134572	100.000	

(*S*)-1-(Benzyloxy)-2-(*m*-tolyl)propan-2-ol (**13c**)

ﾃﾞｰﾀﾌｧｲﾙ名 : C:¥Data(HPLC)¥Martin¥Project2¥Rac¥4_65_3_rac AD3 P60 1.0 210.dat
ﾒｿｯﾄﾞﾌｧｲﾙ名 : C:¥Data(HPLC)¥Sasagawa¥Method 120 min.met
ﾕｰｻﾞｰ名 : System
分析日時 : 2017/04/11 17:19:57
印刷日時 : 2017/04/12 12:17:38

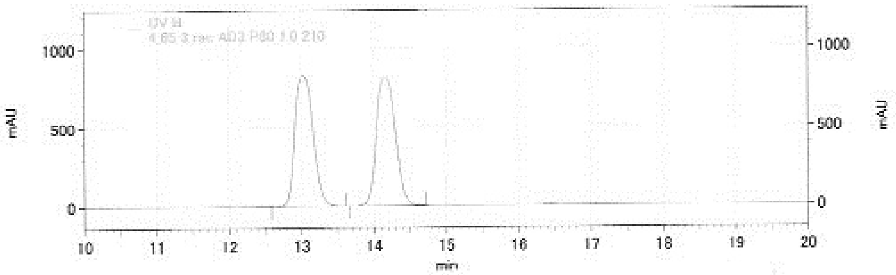

UV_B結果

Pk #	名前	保持時間	面積	面積%	ﾍﾞｰｽﾗｲﾝｺｰﾄﾞ
1		13.03	14344516	49.330	MM
2		14.16	14734175	50.670	MM
ﾄｰﾀﾙ			29078691	100.000	

ﾍﾟｰｼﾞ 1/1

面積%レポート

ﾃﾞｰﾀﾌｧｲﾙ名 : C:¥Data(HPLC)¥Martin¥MP-3-149_Pro AD3 P60 1.0 210.dat
ﾒｿｯﾄﾞﾌｧｲﾙ名 : C:¥Data(HPLC)¥Sasagawa¥Method 120 min.met
ﾕｰｻﾞｰ名 : System
分析日時 : 2016/11/26 9:33:57
印刷日時 : 2017/04/12 12:15:22

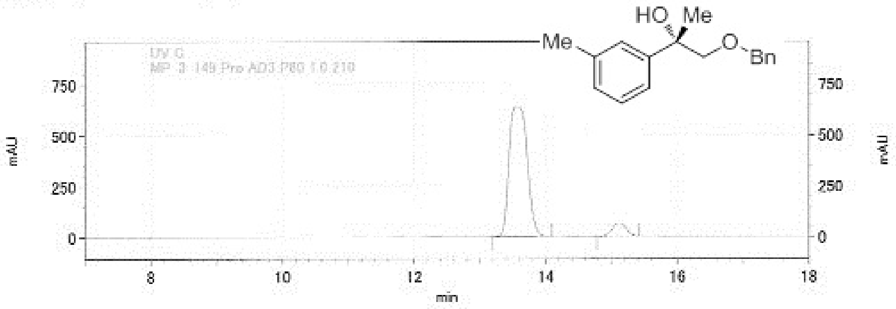

UV_C結果

Pk #	名前	保持時間	面積	面積%	ﾍﾞｰｽﾗｲﾝｺｰﾄﾞ
1		13.57	12148141	92.609	MM
2		15.12	969560	7.391	MM
ﾄｰﾀﾙ			13117701	100.000	

(*S*)-1-(Benzyloxy)-2-(*p*-tolyl)propan-2-ol (**13d**)

ﾍﾟｰｼﾞ 1/1

面積%レポート

ﾃﾞｰﾀﾌｧｲﾙ名 : C:¥Data(HPLC)¥Martin¥Project2¥Rac¥4_65_4_rac AD3 P60 1.0 210.dat
ﾒｿｯﾄﾞﾌｧｲﾙ名: C:¥Data(HPLC)¥Sasagawa¥Method 120 min.met
ﾕｰｻﾞｰ名: System
分析日時: 2017/04/11 17:56:42
印刷日時: 2017/04/12 12:16:57

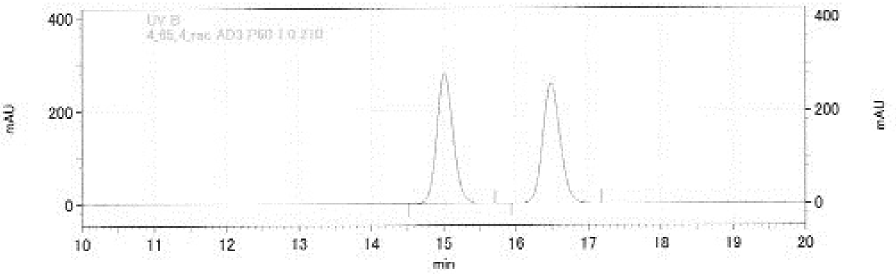

UV B結果

Pk #	名前	保持時間	面積	面積%	ﾍﾞｰｽﾗｲﾝｺｰﾄﾞ
1		15.01	4341439	49.994	MM
2		16.49	4342551	50.006	MM

ﾄｰﾀﾙ			8683990	100.000	

ﾍﾟｰｼﾞ 1/1

面積%レポート

ﾃﾞｰﾀﾌｧｲﾙ名 : C:¥Data(HPLC)¥Martin¥MP-3-175 Pro AD3 P60 1.0 210.dat
ﾒｿｯﾄﾞﾌｧｲﾙ名: C:¥Data(HPLC)¥Sasagawa¥Method 120 min.met
ﾕｰｻﾞｰ名: System
分析日時: 2016/12/13 10:23:40
印刷日時: 2017/04/12 12:16:14

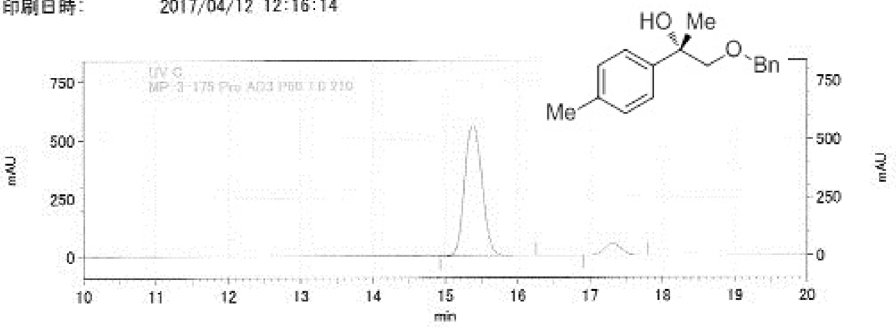

UV_C結果

Pk #	名前	保持時間	面積	面積%	ﾍﾞｰｽﾗｲﾝｺｰﾄﾞ
1		15.38	9431894	91.596	MM
2		17.31	865376	8.404	MM

ﾄｰﾀﾙ			10297270	100.000	

(*S*)-1-(benzyloxy)-2-(4-fluorophenyl)propan-2-ol (**13e**)

ﾃﾞｰﾀﾌｧｲﾙ名 :	C:¥Data(HPLC)¥Martin¥Project2¥Rac¥4_65_6_rac AD3 P60 0.5 210.dat
ﾒｿｯﾄﾞﾌｧｲﾙ名 :	C:¥Data(HPLC)¥Sasagawa¥Method 120 min.met
ﾕｰｻﾞｰ名 :	System
分析日時 :	2017/03/23 15:34:07
印刷日時 :	2017/04/12 12:22:42

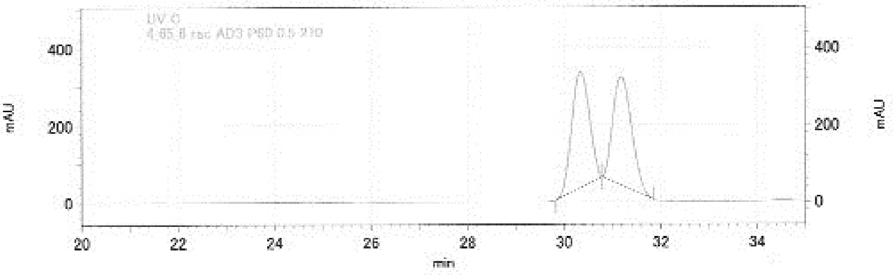

UV_C結果

Pk #	名前	保持時間	面積	面積%	ﾍﾞｰｽﾗｲﾝｺｰﾄﾞ
1		30.34	7653897	50.036	MM
2		31.18	7642880	49.964	MM
ﾄｰﾀﾙ			15296777	100.000	

面積%レポート

ﾃﾞｰﾀﾌｧｲﾙ名 :	C:¥Data(HPLC)¥Martin¥MP-3-147_Pro AD3 P60 0.5 210.dat
ﾒｿｯﾄﾞﾌｧｲﾙ名 :	C:¥Data(HPLC)¥Sasagawa¥Method 120 min.met
ﾕｰｻﾞｰ名 :	System
分析日時 :	2016/11/26 10:14:59
印刷日時 :	2017/04/11 16:26:41

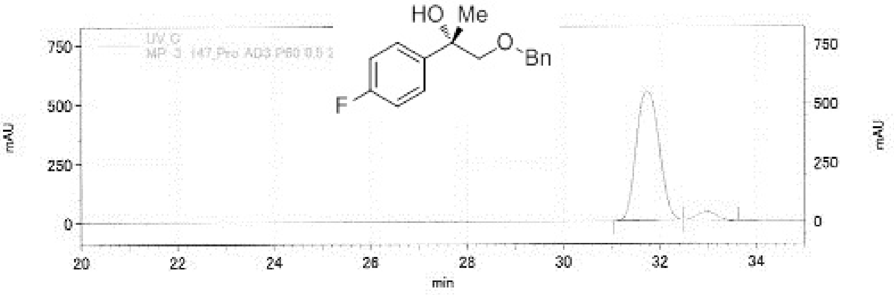

UV_C結果

Pk #	名前	保持時間	面積	面積%	ﾍﾞｰｽﾗｲﾝｺｰﾄﾞ
1		31.74	17894995	94.659	MM
2		32.97	1009662	5.341	MM
ﾄｰﾀﾙ			18904657	100.000	

(*S*)-1-(benzyloxy)-2-(4-methoxyphenyl)propan-2-ol (**13f**)

面積%レポート

ﾃﾞｰﾀﾌｧｲﾙ名 : C:¥Data(HPLC)¥Martin¥Project2¥Rac¥4_65_5_rac AD3 P60 1.0 210.dat
ﾒｿｯﾄﾞﾌｧｲﾙ名: C:¥Data(HPLC)¥Sasagawa¥Method 120 min.met
ﾕｰｻﾞｰ名: System
分析日時: 2017/04/11 18:34:29
印刷日時: 2017/04/12 12:18:41

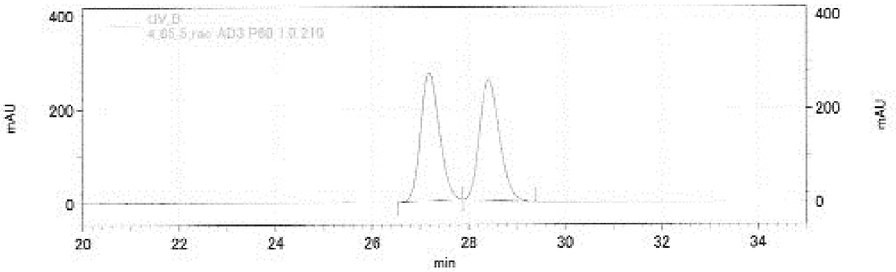

UV_B結果

Pk #	名前	保持時間	面積	面積%	ﾍﾞｰｽﾗｲﾝｺｰﾄﾞ
1		27.19	7344450	49.933	MM
2		28.42	7364201	50.067	MM
ﾄｰﾀﾙ			14708651	100.000	

ﾍﾟｰｼﾞ 1/1

面積%レポート

ﾃﾞｰﾀﾌｧｲﾙ名 : C:¥Data(HPLC)¥Martin¥MP-3-142_Pro AD3 P60 1.0 210.dat
ﾒｿｯﾄﾞﾌｧｲﾙ名: C:¥Data(HPLC)¥Sasagawa¥Method 120 min.met
ﾕｰｻﾞｰ名: System
分析日時: 2016/11/22 15:58:11
印刷日時: 2017/04/11 16:26:02

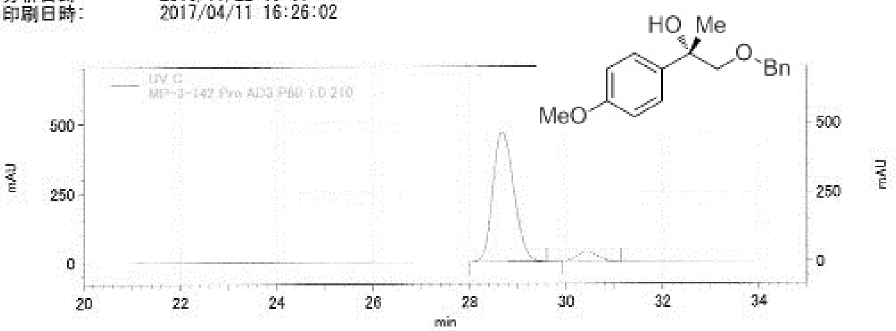

UV_C結果

Pk #	名前	保持時間	面積	面積%	ﾍﾞｰｽﾗｲﾝｺｰﾄﾞ
1		28.68	14161153	93.455	MM
2		30.43	991788	6.545	MM
ﾄｰﾀﾙ			15152941	100.000	

(*S*)-1-(Benzyloxy)-2-(naphthalen-2-yl)propan-2-ol (**13g**)

ページ 1/1

面積%レポート

データファイル名 : C:¥Data(HPLC)¥Martin¥MP-3-141__rac_Pro AD3 P60 1.0 210.dat
メソッドファイル名 : C:¥Data(HPLC)¥Sasagawa¥Method 120 min.met
ユーザー名 : System
分析日時 : 2017/05/09 12:28:33
印刷日時 : 2017/05/09 13:20:52

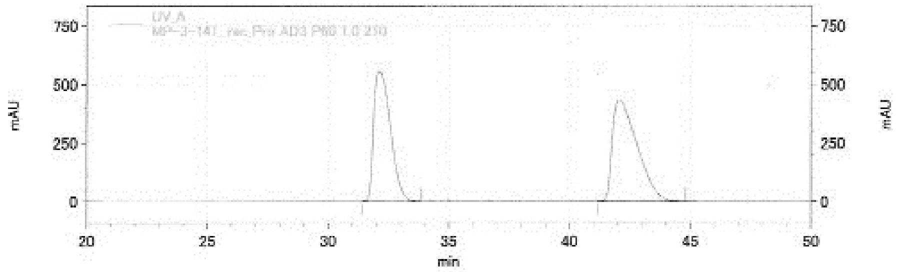

UV_A結果

Pk #	rt	area	area%
1	32.14	26911074	46.535
2	42.07	30918269	53.465
トータル		57829343	100.000

ページ 1/1

面積%レポート

データファイル名 : C:¥Data(HPLC)¥Martin¥MP-3-141_Pro AD3 P60 1.0 210.dat
メソッドファイル名 : C:¥Data(HPLC)¥Sasagawa¥Method 120 min.met
ユーザー名 : System
分析日時 : 2017/05/09 13:23:56
印刷日時 : 2017/05/09 14:24:21

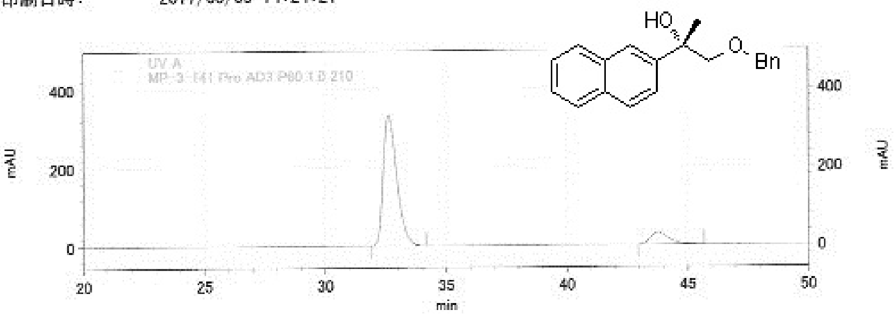

UV_A結果

Pk #	rt	area	area%
1	32.63	12956399	89.162
2	43.77	1574891	10.838
トータル		[illegible]	[illegible]

(*S*)-1-(Benzyloxy)-2-(thiophen-3-yl)propan-2-ol (**13h**)

面積%レポート

データファイル名 : C:¥Data(HPLC)¥Martin¥Project2¥Rac¥4_65_7 rac AD3 P60 1.0 210.dat
メソッドファイル名: C:¥Data(HPLC)¥Sasagawa¥Method 120 min.met
ユーザー名: System
分析日時: 2017/04/12 20:17:38
印刷日時: 2017/04/12 20:56:23

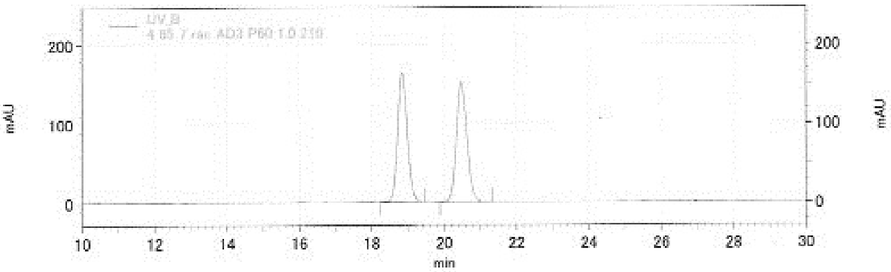

UV_B結果

Pk #	名前	保持時間	面積	面積%	ベースラインコード
1		18.85	3042191	49.928	MM
2		20.48	3050970	50.072	MM
トータル			6093161	100.000	

ページ 1/1

面積%レポート

データファイル名 : C:¥Data(HPLC)¥Martin¥MP-4-33 Pro IC3 P60 1.0 210.dat
メソッドファイル名: C:¥Data(HPLC)¥Sasagawa¥Method 120 min.met
ユーザー名: System
分析日時: 2017/01/18 15:41:07
印刷日時: 2017/04/11 16:27:20

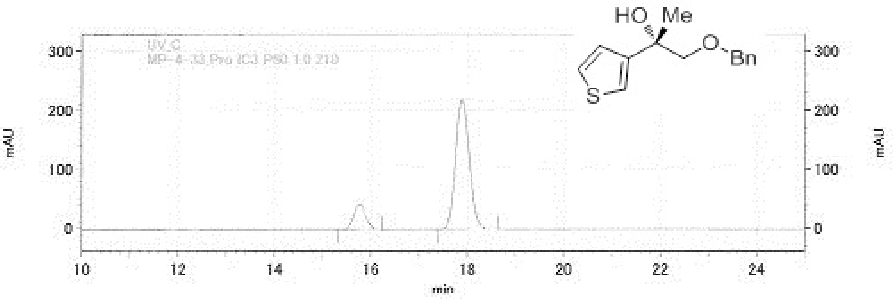

UV_C結果

Pk #	名前	保持時間	面積	面積%	ベースラインコード
1		15.77	721577	14.260	MM
2		17.89	4338517	85.740	MM
トータル			5060094	100.000	

(*S*)-1-(Benzyloxy)-2-phenylpent-4-en-2-ol (**13i**)

ﾍﾟｰｼﾞ 1/1

面積%レポート

ﾃﾞｰﾀﾌｧｲﾙ名 : C:¥Data(HPLC)¥Martin¥Project2¥Rac¥4_65_10_rac AD3 P60 0.5 210.dat
ﾒｿｯﾄﾞﾌｧｲﾙ名 : C:¥Data(HPLC)¥Sasagawa¥Method 120 min.met
ﾕｰｻﾞｰ名 : System
分析日時 : 2017/03/24 12:24:39
印刷日時 : 2017/04/14 9:27:43

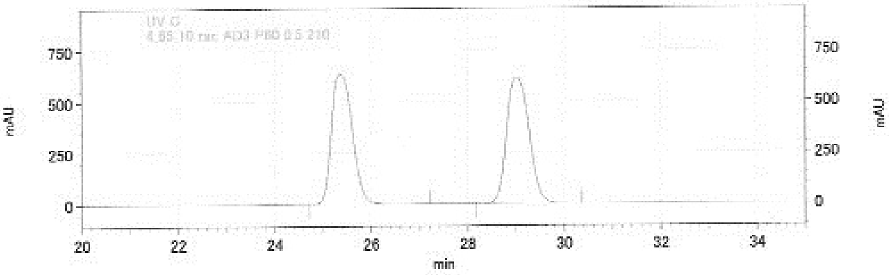

UV_C結果

Pk #	名前	保持時間	面積	面積%	ﾍﾞｰｽﾗｲﾝｺｰﾄﾞ
1		25.37	18977048	49.035	MM
2		29.02	19723591	50.965	MM
ﾄｰﾀﾙ			38700639	100.000	

ﾍﾟｰｼﾞ 1/1

面積%レポート

ﾃﾞｰﾀﾌｧｲﾙ名 : C:¥Data(HPLC)¥Martin¥MP-4-17_Pro AD3 P60 0.5 210.dat
ﾒｿｯﾄﾞﾌｧｲﾙ名 : C:¥Data(HPLC)¥Sasagawa¥Method 120 min.met
ﾕｰｻﾞｰ名 : System
分析日時 : 2017/01/18 14:53:26
印刷日時 : 2017/04/14 11:53:58

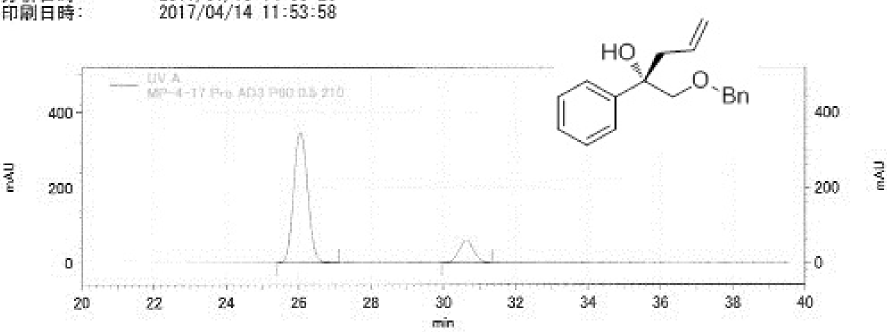

UV_A結果

Pk #	名前	保持時間	面積	面積%	ﾍﾞｰｽﾗｲﾝｺｰﾄﾞ
1		26.04	9314952	84.650	MM
2		30.63	1689063	15.350	MM
ﾄｰﾀﾙ			11004015	100.000	

1 mmol Reaction

(*S*)-2-((4-Methylbenzyl)oxy)-1-phenylethanol **2ad**

ﾃﾞｰﾀﾌｧｲﾙ名 : C:¥Data(HPLC)¥Martin¥MP_91 Pro AD3 P60 0.5 210.dat
ﾒｿｯﾄﾞﾌｧｲﾙ名 : C:¥Data(HPLC)¥Sasagawa¥Method 120 min.met
ﾕｰｻﾞｰ名 : System
分析日時 : 2017/04/20 9:27:42
印刷日時 : 2017/04/20 11:00:47

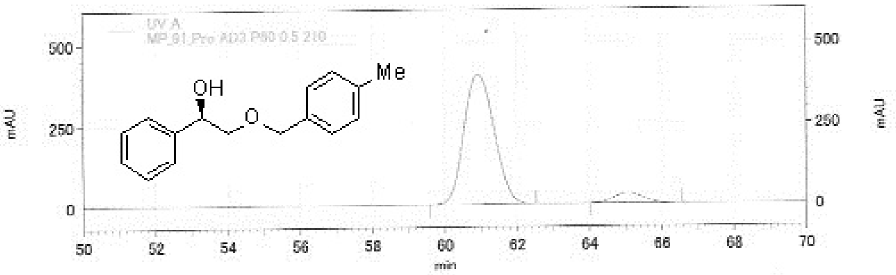

UV_A結果

Pk #	rt	area	area%
1	60.93	23353095	92.946
2	65.08	1772438	7.054
ﾄｰﾀﾙ		25125533	100.000

ﾍﾟｰｼﾞ 1/1

面積%レポート

ﾃﾞｰﾀﾌｧｲﾙ名 : C:¥Data(HPLC)¥Martin¥MP-4-91_Sub ADH E20 1.0 210.dat
ﾒｿｯﾄﾞﾌｧｲﾙ名 : C:¥Data(HPLC)¥Sasagawa¥Method 120 min.met
ﾕｰｻﾞｰ名 : System
分析日時 : 2017/03/15 11:59:45
印刷日時 : 2017/04/20 8:58:52

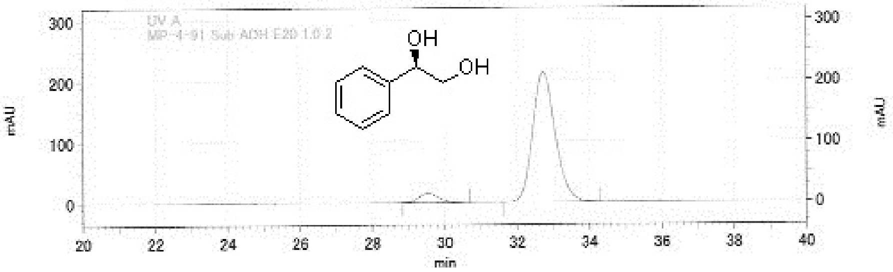

UV_A結果

Pk #	名前	保持時間	面積	面積%	ﾍﾞｰｽﾗｲﾝｺｰﾄﾞ
1		29.54	559313	5.698	MM
2		32.73	9257275	94.302	MM
ﾄｰﾀﾙ			9816588	100.000	

Alkylation of the Secondary Alcohol

(*R*)-2-(Benzyloxy)-2-phenylethanol **3aa**

面積%レポート

データファイル名 : C:¥Data(HPLC)¥Martin¥4_111_pro_secalc AD3 P60 1.0 210.dat
メソッドファイル名: C:¥Data(HPLC)¥Sasagawa¥Method 120 min.met
ユーザー名: System
分析日時: 2017/04/17 17:07:26
印刷日時: 2017/04/17 17:54:07

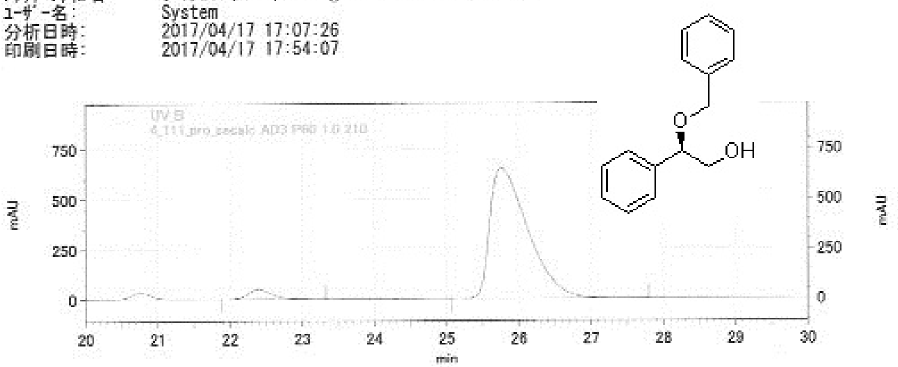

UV_B結果

Pk #	名前	保持時間	面積	面積%	ベースラインコード
1		22.39	1133290	4.553	MM
2		25.76	23758884	95.447	MM
トータル			24892174	100.000	

ページ 1/1

面積%レポート

データファイル名 : C:¥Data(HPLC)¥Martin¥4_111_side_prialc AD3 P60 1.0 210.dat
メソッドファイル名: C:¥Data(HPLC)¥Sasagawa¥Method 120 min.met
ユーザー名: System
分析日時: 2017/04/17 17:55:35
印刷日時: 2017/04/17 18:40:14

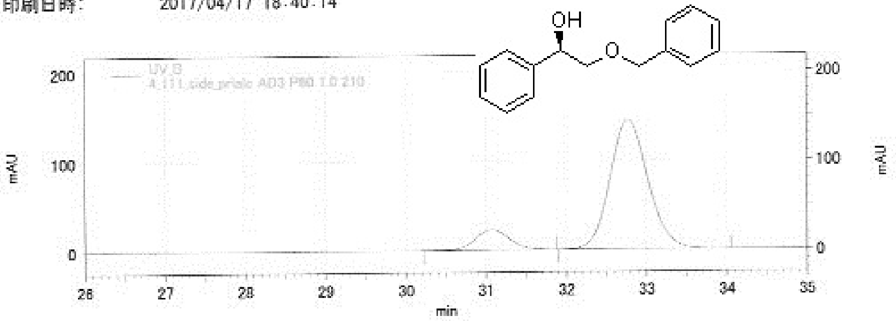

UV_B結果

Pk #	名前	保持時間	面積	面積%	ベースラインコード
1		31.08	674862	12.492	MM
2		32.78	4727408	87.508	MM
トータル			5402270	100.000	

Desymmetrization

(1*S*,2*R*)-2-((2-methylbenzyl)oxy)-1,2-diphenylethanol (**13**)

ﾍﾟｰｼﾞ 1/1

面積%レポート

ﾃﾞｰﾀﾌｧｲﾙ名 : C:¥Data(HPLC)¥Martin¥Project2¥MP-5-77_C IC3 P60 0.5 210.dat
ﾒｿｯﾄﾞﾌｧｲﾙ名: C:¥Data(HPLC)¥Sasagawa¥Method 120 min.met
ﾕｰｻﾞｰ名: System
分析日時: 2017/08/04 9:43:26
印刷日時: 2017/08/04 11:46:40

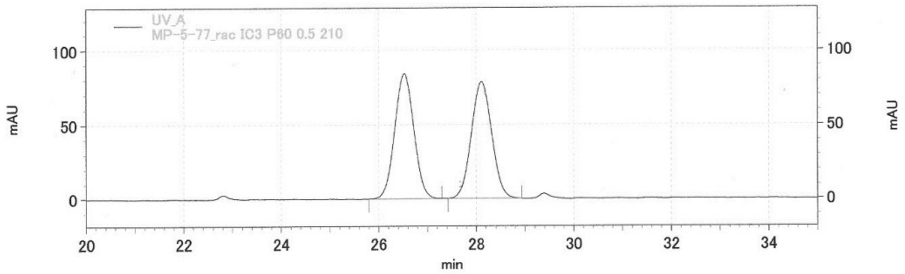

UV_A結果

No	RT	Area	Area%
1	26.53	2330069	50.168
2	28.11	2314437	49.832

ﾍﾟｰｼﾞ 1/1

面積%レポート

ﾃﾞｰﾀﾌｧｲﾙ名 : C:¥Data(HPLC)¥Martin¥Project2¥MP-5-77_D2 IC3 P60 0.5 210.dat
ﾒｿｯﾄﾞﾌｧｲﾙ名: C:¥Data(HPLC)¥Sasagawa¥Method 120 min.met
ﾕｰｻﾞｰ名: System
分析日時: 2017/08/05 13:26:46
印刷日時: 2017/08/05 14:02:17

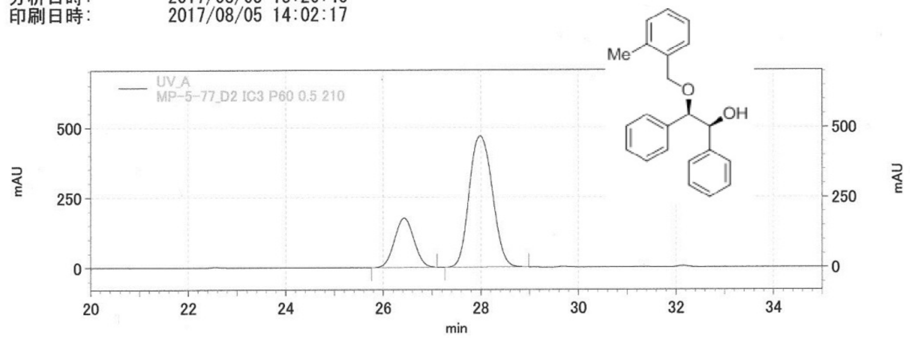

UV_A結果

No	RT	Area	Area%
1	26.43	4865732	23.954
2	27.98	15447453	76.046
ﾄｰﾀﾙ		20313185	100.000
